# Different reactivity of phosphorylallenes under the action of Brønsted or Lewis acids: a crucial role of involvement of the P=O group in intra- or intermolecular interactions at the formation of cationic intermediates

**DOI:** 10.3762/bjoc.15.151

**Published:** 2019-07-08

**Authors:** Stanislav V Lozovskiy, Alexander Yu Ivanov, Aleksander V Vasilyev

**Affiliations:** 1 Department of Organic Chemistry, Institute of Chemistry, Saint Petersburg State University, Universitetskaya nab., 7/9, Saint Petersburg, 199034, Russia; 2 Center for Magnetic Resonance, Research Park, St. Petersburg State University, Universitetskiy pr., 26, Saint Petersburg, Petrodvoretz, 198504, Russia; 3 Department of Chemistry, Saint Petersburg State Forest Technical University, Institutsky per., 5, Saint Petersburg, 194021, Russia

**Keywords:** aluminum chloride, cation, intermediate, oxaphospholium ions, phosphorylallenes, phosphoryl group, triflic acid

## Abstract

3-Methylbuta-1,2-dien-1-ylphosphonic acid derivatives (phosphorylallenes) [X_2_(O=)P–CR=C=CMe_2_, X = Cl, OMe, NR_2_, or SAr] undergo intramolecular cyclization into the corresponding 1,2-oxaphospholium ions in the Brønsted superacid TfOH. These cations have been thoroughly studied by means of NMR spectroscopy. The hydrolysis of superacidic solutions of these species afforded cyclic phosphonic acids and other phosphorus-containing compounds. Contrary to Brønsted acids, 3-methylbuta-1,2-dien-1-ylphosphonic dichloride [Cl_2_(O=)P–HC=C=CMe_2_] reacted with the Lewis acid AlCl_3_ in an intermolecular way forming noncyclic intermediates, which were investigated by NMR spectroscopy and DFT calculations. Hydrolysis of these species resulted in the formation of phosphoryl-substituted allyl alcohols and 1,3-butadienes. A strong coordination of the oxygen of the P=O group with AlCl_3_ prevented the formation of cyclic 1,2-oxaphospholium ions and played a crucial role in the different reactivity of such phosphorylallenes under the action of Brønsted or Lewis acids. Apart from that, the reaction of dichlorophosphorylallenes with arenes and AlCl_3_ led to products of hydroarylation of the allene system, phosphoryl-substituted alkenes and/or indanes. This is the first example of a Lewis acid-promoted intermolecular hydroarylation of allenes bearing electron-withdrawing substituents. Plausible reaction mechanisms have been proposed on the basis of the investigated reactions, and NMR analysis and DFT studies of the intermediate cationic species.

## Introduction

Electrophilic reactions of allenes have been intensely explored in organic synthesis [1–3]. In particular, reports on electrophilic activation of phosphorylallenes are numerous [4–10]. Miscellaneous electrophiles, such as sulfenyl, selenyl, and telluryl chlorides, were used in reactions with these allenes. However, only a few studies have been focused on reactions of phosphorylallenes with Brønsted acids [11–12]. These reactions proceed through an intermediate formation of the corresponding 2,5-dihydro-1,2-oxaphosphol-2-ium ions. The progenitor of the oxaphospholium ion family, 2,2-dichloro-5,5-dimethyl-1,2-oxaphosphol-2-ium, was postulated for the first time in 1978 [12].

We have recently reported on the generation, NMR characterization and reactions of oxaphospholium ions bearing phenyl or phenoxy substituents at the phosphorus atom of phosphorylallenes [13–16]. These cations were intermediates in Brønsted and Lewis acid-promoted intramolecular reactions of phosphorus-containing allenes with aromatic π-nucleophiles giving rise to various (bi)cyclic phosphorus-containing compounds [13–16].

It should be especially emphasized that intermolecular reactions of phosphorylallenes with arenes have not been yet achieved. In general, intermolecular hydroarylation of allenes has been developed for reactions catalyzed by complexes of various metals [17], such as Pd [18–20], Pt [21], Au [22–25], Ir [26], Rh [27–28], and Co [29]. However, only electron-rich allenes, bearing electron-donating substituents, take part in the metal-catalyzed reactions. There are just a few examples of Brønsted acid catalyzed intermolecular hydroarylations of allenes by electron-rich arenes, indoles [30] or phenols [31]. Other arenes (benzene and its substituted derivatives) have not been involved in these reactions. Concerning electron-deficient allenes, bearing electron-withdrawing groups, there is only one example of a trifluoroacetic acid-promoted hydroarylation with indoles [30]. To the best of our knowledge, up to the moment, there are no examples for an intermolecular hydroarylation of electron-deficient allenes by benzene derivatives under the action of strong Brønsted or Lewis acids.

The main goals of this work were to study transformations of various phosphorylallenes under electrophilic activation with Brønsted or Lewis (super)acids, including reactions with arenes as π-nucleophiles, and investigation of intermediate cationic species by means of NMR and DFT calculations.

**Figure 1:**
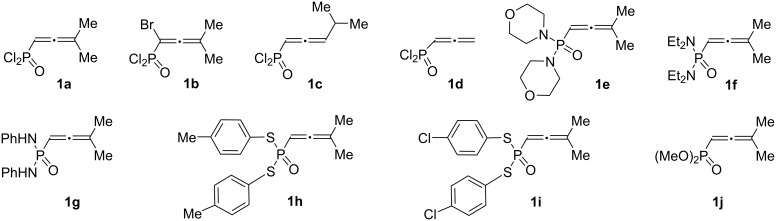
Allenes **1a–j** used in this study.

Allenes used in this study are presented in Figure 1. We explored allenes having different substituents at the phosphoryl group: chloro (**1a–d**), amino (**1e–g**), arylsulfanyl (**1h**,**i**), and methoxy (**1j**).

## Results and Discussion

### Reactions of allenes with Brønsted acids

Allenes **1a,b,e–j** upon dissolving in TfOH in an NMR tube at room temperature formed intensively colored solutions of the corresponding 1,2-oxaphospholium ions **A–H** (Table 1). These species are formed by protonation of the central carbon atom of the allene system that gives the corresponding allyl cations, which undergo cyclization onto the oxygen of the P=O group. These ions have similar NMR data: the signal of the new proton H4 is located in the range 6.30–8.07 ppm, the signal of vinyl carbon C4 at 166.8–171.9 ppm, and the signal of quaternary carbon C5 at 96.0–116.3 ppm. It is worth noting that 2,2-dichloro (**A**, **B**) and 2,2-diarylsulfanyl (**F**, **G**)-substituted cations exhibit down field shifted signals in the ^31^P NMR (δ 87.82–115.37 ppm) in comparison with 2,2-diamino (**C**, **D**, **E1**) and 2,2-dimethoxy (**H**)-substituted species (δ ^31^P 52.87–70.79 ppm). This reveals that, for amino and methoxy substituents, positive charge is delocalized onto these groups to a greater extent than in the case of chloro or arylsulfanyl ones.

**Table 1:** Selected ^1^H, ^13^C and ^31^P NMR data for cations **A–H** derived from the protonation of the corresponding allenes **1a**,**b**,**e–j** in TfOH at room temperature.

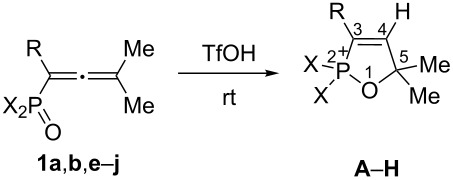										

Allene	Cation	R	X	^1^H NMR, δ, ppm (*J*, Hz)			^13^C NMR, δ, ppm (*J*, Hz)			^31^P NMR, δ, ppm
		
				H3	H4		C3	C4	C5	

**1a**	**A**	H	Cl	8.11 dd (68.3, 8.3)	7.00 dd (49.4, 8.3)		116.4 d (111.3)	169.9 d (14.3)	110.8 d (10.8)	97.04
**1b**	**B**	Br	Cl	–	8.07 dd (55.1)		104.9 d (137.0)	169.8 d (33.2)	113.6 d (5.4)	87.82
**1e**	**C**	H	O(CH_2_CH_2_)_2_N	7.89 dd (49.2, 8.3)	6.45 dd (36.9, 8.3)		109.3 d (131.9)	170.3 d (14.3)	98.9 d (10.0)	64.33
**1f**	**D**	H	Et_2_N	7.49 dd (49.3, 8.2)	6.56 dd (38.8, 8.2)		109.1 d (126.6)	170.0	102.0	70.79
**1g**	**E1**	H	PhNH	m (overlapping with other signals)	6.42 dd (37.1, 7.8)		111.6 d (137.5)	168.3 d (12.8)	96.0	52.87
**1h**	**F**	H	4-MeC_6_H_4_S	7.26 dd (54.0, 7.9)	6.33 dd (45.5, 7.9)		113.1 d (82.7)	167.1 d (10.0)	116.3 d (7.6)	115.37
**1i**	**G***^a^*	H	4-ClC_6_H_4_S	7.41 dd (54.4, 7.9)	6.40 dd (45.9, 7.9)		111.1 d (81.0)	166.8 d (10.3)	101.8	114.56
**1j**	**H**	H	MeO	7.93 dd (54.6, 8.5)	6.30 dd (35.8, 8.4)		107.3 d (159.4)	171.9 d (14.4)	97.8 d (13.2)	57.82

^a^Content of cation **G** in reaction solution was ≈50% based on ^31^P NMR data.

**Scheme 1:**
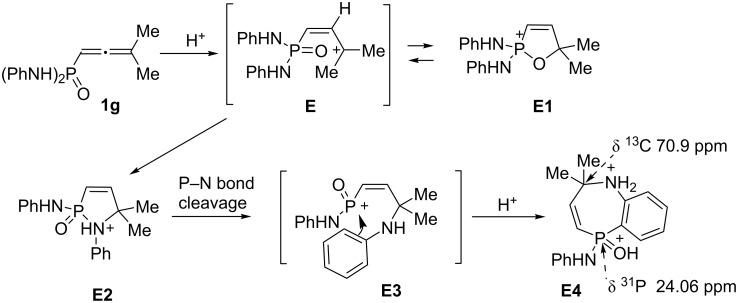
Transformations of allene **1g** in TfOH leading to the formation of cations **E1**, **E2** and **E4** including selected spectral data for cation **E4**.

**Figure 2:**
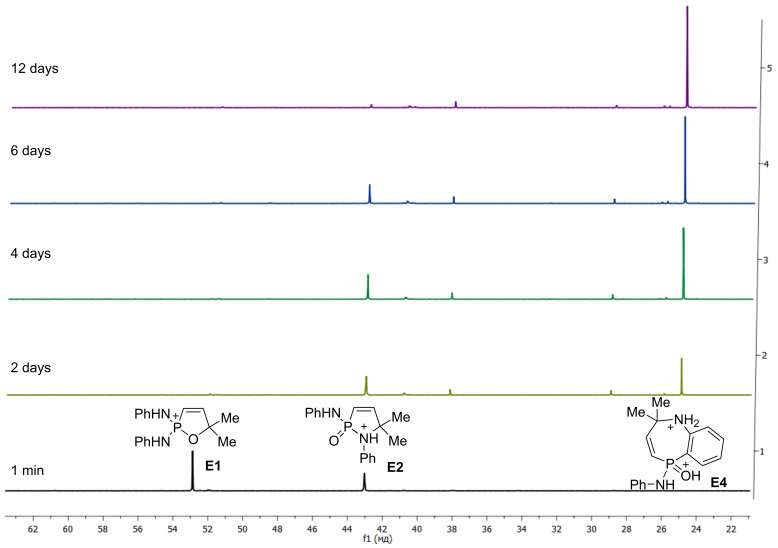
^31^P NMR monitoring of the progress of transformation of **E1** into **E2** and **E4** in TfOH at room temperature.

Cations **A–D**, and **F–H** are stable in TfOH at room temperature for a long time, they are not transformed into other species under the superacidic conditions. Unlike the others, allene **1g** undergoes consequent transformations in TfOH at room temperature (see Scheme 1 and Figure 2). First, when dissolved in the acid, allene **1g** forms oxaphospholium ion **E1** (Table 1) through an intermediate formation of allyl cation **E** (Scheme 1). Ion **E1** is transformed very fast into another species; after one minute new signals appear in the NMR spectra (see ^31^P NMR monitoring of this process in Figure 2), and after 12 hours it is completely converted to this new cation. It is most likely that this species is 1,2-azaphosphol-2-ium ion **E2**, which is formed through allyl cation **E**.

However, cation **E2**, in turn, is further transformed into one more species during several days. The set of spectral data (see below) for this final species indicates that, most likely it should be seven-membered heterocyclic cation **E4**, which is formed through the P–N bond cleavage in **E2** and formation of intermediate cation **E3** (Scheme 1).

In the ^31^P NMR spectra, the signal of **E4** is the most up field shifted (δ 24 ppm, see Figure 2 and Scheme 1) in comparison with signals of the species **E1** (δ 53 ppm) and **E2** (δ 43 ppm). This difference may reveal that phosphorus in cation **E4** is bound to a carbon atom, rather than to a heteroatom O or N, like in **E1** and **E2**. Structurally close six-membered ring cations, having the C–P bond, resonate at 30.5–31.9 in ^31^P NMR [16], that is close to the spectrum for species **E4**.

Apart from that, in ^13^C NMR spectra, the signals of quaternary carbon bearing two methyl groups in **E2** and **E4** are very close (δ 70.3–70.9 ppm, see Scheme 1). Contrary to that, the signal of this carbon for **E2** is very much down field shifted (δ 96.1 ppm). This indicates that in species **E2** and **E4** this carbon is connected to a protonated amino group, and in **E1** it is bound to oxygen. The same range of absorbance around 100 ppm for this carbon was observed previously for other oxaphospholium ions [14,16].

**Scheme 2:**
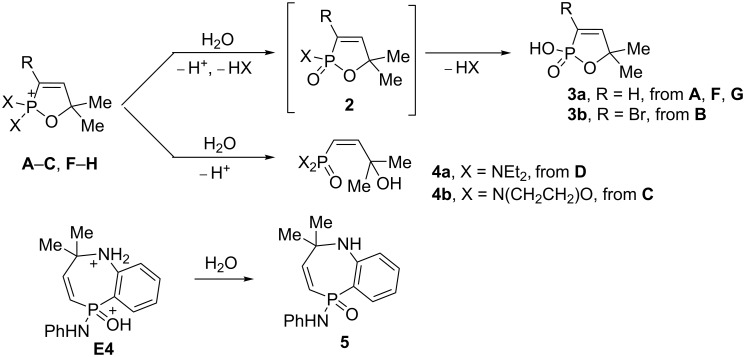
Results of the hydrolysis of cations **A**–**H**.

Then, we carried out hydrolysis of cations **A**–**H** (Scheme 2). Results of hydrolysis strongly depend on the substituent X on the phosphorus atom. Ions containing a labile P–X bond (X = Cl, O, S), namely **A**, **B**, and **F**–**H**, gave unstable adducts **2** (registered by GC–MS), which are further transformed into acids **3**. The structure of compound **3a** was confirmed by X-ray analysis (see Supporting Information File 1). On the other hand, hydrolysis of cations **C**,**D**, bearing a stable P–N bond, resulted in the formation of allyl alcohols **4**. Aqueous work-up of a superacidic solution of cation **E4** led to azaphosphepine-5-oxide **5**. This substance is insoluble in organic solvents, however, we were able to measure its ^1^H NMR spectrum in D_2_O at elevated temperature (80 °C, see Supporting Information File 1).

**Scheme 3:**
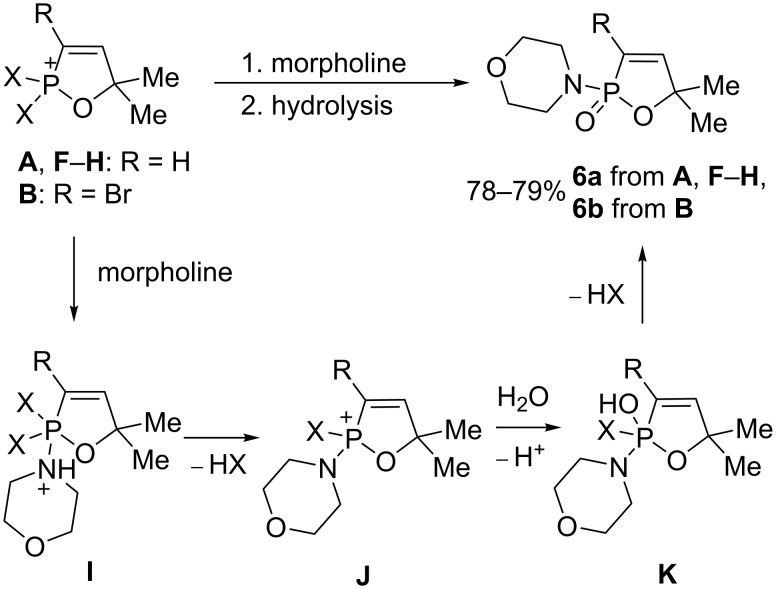
Preparation of amides **6a**,**b** from cations **A**, **B**, and **F**–**H**.

Taking into account the stability of the P–N bond against hydrolysis, we conducted reactions of the cations **A**, **B**, and **F**–**H** with morpholine (Scheme 3). Amides **6a,b** were isolated as products of these reactions in excellent yields. The plausible reaction mechanism includes at the first stage nucleophilic attack of morpholine onto the phosphorus cationic center that gives cation **I**, which is transformed into species **J**. Hydrolysis of the latter leads to cation **K** and then finally to amides **6a**,**b**.

**Scheme 4:**
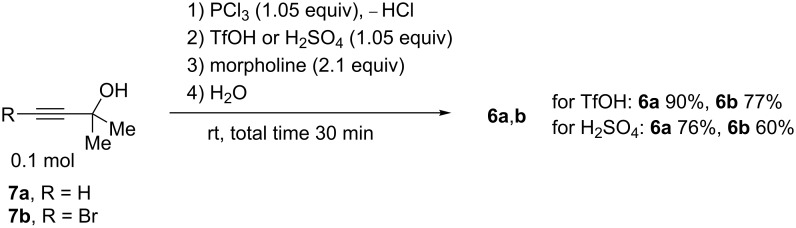
Large-scale one-pot solvent-free synthesis of amides **6a**,**b** from the corresponding propargylic alcohols.

We carried out a large-scale one-pot solvent-free synthesis of amides **6a**,**b** starting from propargyl alcohols **7a**,**b** at room temperature (Scheme 4). At the first step, alcohols **7a**,**b** in the reaction with PCl_3_ were transformed into the corresponding allenes **1a**,**h**. Then, the addition of Brønsted acid (TfOH or H_2_SO_4_) gave cations **A** and **B**, respectively. The interaction of these species with morpholine followed by hydrolysis furnished the target amides **6a**,**b** in total yields of 60–90% (see procedures in Supporting Information File 1).

It should be noted that allene **1d** bearing no alkyl groups and monoalkylated allene **1c** formed complex mixtures of oligomeric products under the action of various Brønsted acids (H_2_SO_4_, FSO_3_H, TfOH). In this case, the intermediate oxaphospholium ions are unstable and undergo consequent transformations. Apart from that, attempts to quench cations **A–H** with external aromatic π-nucleophiles failed. No products of intermolecular electrophilic aromatic substitution were obtained.

### Reactions of allenes with Lewis acid AlCl_3_

Then, we checked reactions of allenes **1a–j** with and without benzene under the action of the strong Lewis acid AlCl_3_, using benzene or dichloromethane as a solvent, followed by hydrolysis of the reaction mixtures. Allenes **1c**–**j** gave complex mixtures of oligomeric products under these conditions. However, allenes **1a**,**b** afforded the desired product of hydroarylation with benzene (vide infra).

AlCl_3_-promoted reactions of allene **1a** were studied under various conditions (Table 2). This compound in reaction with AlCl_3_ without benzene afforded a mixture of allyl alcohol *Z***-9** and diene *E-***10a** after aqueous work-up (Table 2, entries 1 and 2). The amount of 2.1 equivalents of AlCl_3_ is sufficient for activation of this transformation (compared to the amount of AlCl_3_ in entries 1 and 2, Table 2). On the other hand, 1 equivalent of AlCl_3_ is not enough to activate allene **1a**; thus, under these conditions, only acid **2** was obtained as a product of the hydrolysis of starting compound **1a** (Table 2, entry 3). Methanolysis of the reaction mixture gave diene *E*-**10b** (Table 2, entry 7). The reaction of allene **1a** with benzene resulted in the formation of alkene *Z*-**11a**, as a product of intermolecular hydroarylation of the carbon–carbon double bond (Table 2, entries 4–6). This reaction required 2.1 equivalents of AlCl_3_, 1.05 equivalents of benzene and five minutes at room temperature (Table 2, entry 4). It is worth noting, that the use of other Lewis acids, NiCl_2_, EuCl_3_, FeCl_3_, CuOTf, AgNO_3_, did not activate allene **1a**; in these reactions only the product of the hydrolysis **8** was finally isolated.

**Table 2:** AlCl_3_-promoted reactions of allene **1a** at room temperature with/without benzene at various conditions.

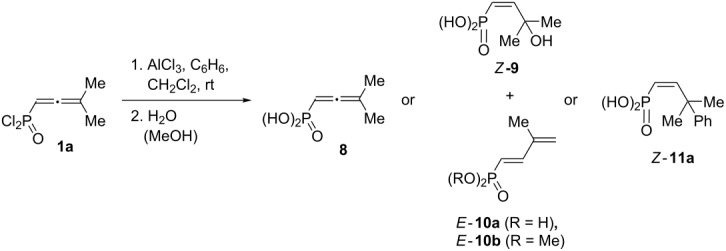						

Entry	Equiv of AlCl_3_	Equiv of benzene	Time, min	Yield of **8**, %	Yield of **9** + **10a**,**b**, %	Yield of **11a**, %

1	5	no benzene	15	–	33 (**9**) + 32 (**10a**)	–
2	2.1	no benzene	15	–	35 (**9**) + 36 (**10a**)	–
3	1	1.05	15	98	–	–
4	2.1	1.05	5	–	–	82
5	2.1	1.05	15	–	–	78
6	2.1	1.05	60	–	–	81
7^a^	2.1	no benzene	15	–	45 (**10b**)	–

^a^Reaction mixture was quenched with methanol.

The configuration of the carbon–carbon double bond in compounds *Z***-9**, *E***-10b** and *Z***-11a** was determined on the basis of the observed values of the spin–spin interaction constants for vinyl protons (13–14 Hz for *cis*-isomers and 17–18 Hz for *trans*-isomers), and using H,H-NOESY correlations for *Z-***11a** (see Supporting Information File 1).

Having these conditions for hydroarylation of allene **1a** in hand (Table 2, entry 4), we conducted reactions with the series of arenes (Table 3). An excess of methanol was used for quenching of reaction mixtures instead of water. This treatment produced dimethoxyphosphoryl groups [(MeO)_2_P=O] in the reaction products, rather than the acidic group [(HO)_2_P=O] in compounds **8–11a** (Table 2). The presence of the (MeO)_2_P=O group in the structures of reaction products makes them more soluble in organic solvents and easy to isolate in preparative reactions.

**Table 3:** AlCl_3_-promoted reactions of allene **1a** with arenes leading to alkenes **11** and indanes **12** at room temperature for 5 min.

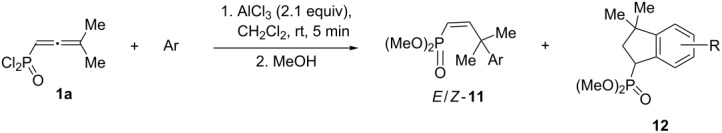		

Entry	Starting arene, ArH	Reaction products **11** and **12**, yield, %

1	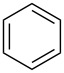	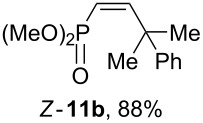
2	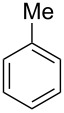	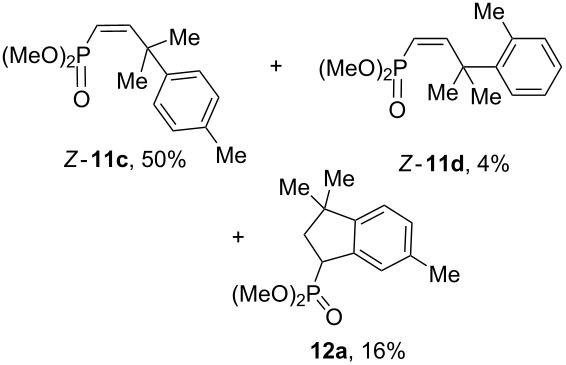
3	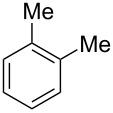	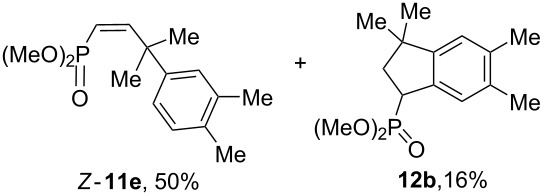
4	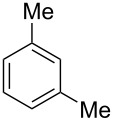	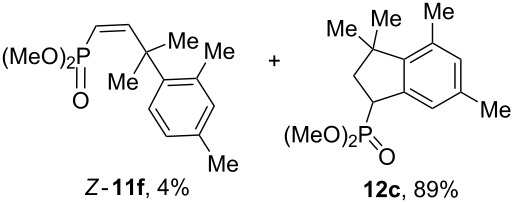
5	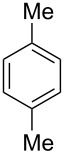	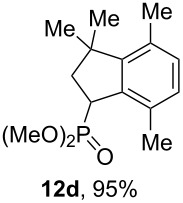
6**^a^**	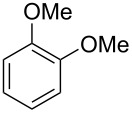	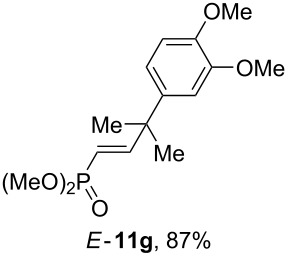
7	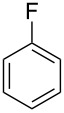	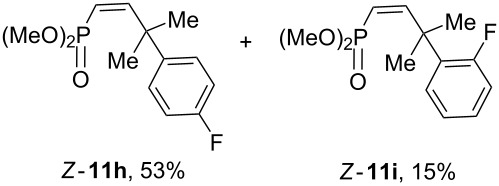
8	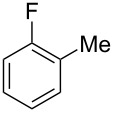	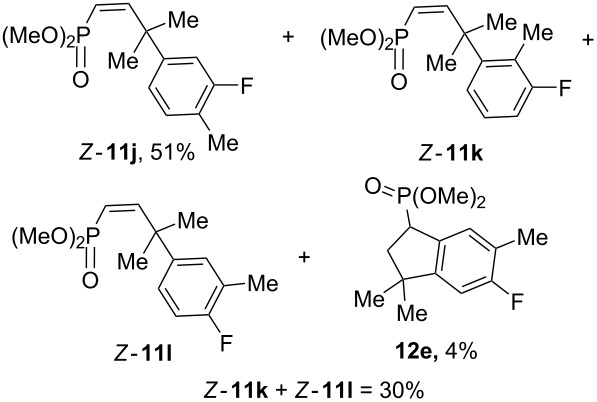
9	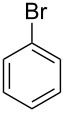	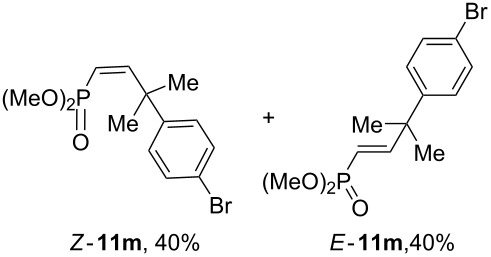

^a^Reaction was run with 3.1 equiv of AlCl_3._

Depending on the structure of the starting arene, allene **1a** gave two kinds of reaction products, *E*-/*Z*-alkenes **11** and/or indanes **12** (Table 3). Thus, in reactions with benzene, only *cis*-alkene *Z*-**11b** was obtained in 88% yield (Table 3, entry 1). The sole formation of alkenes *E*-**11g**, *Z*-**11h** and *Z*-**11i**, and *E*/*Z*-**11l** was also observed in reactions with 1,2-dimethoxybenzene (veratrole) (Table 3, entry 6), fluorobenzene (Table 3, entry 7) and bromobenzene (Table 3, entry 9), respectively. On the other hand, reactions with methylbenzenes (toluene, *o*- and *m*-xylenes, *o-*fluorotoluene) led to mixtures of alkenes **5** and indanes **6** (Table 3, entries 2–4, and 8). However, *p*-xylene gave the only reaction product, indane **6d**, in nearly quantitative yield of 95% (Table 3, entry 5).

It should be emphasized that compounds **11** and **12** were obtained as inseparable mixtures after TLC separation due to their close chromatographic retention parameters. However, *E*- and *Z*-isomers of alkenes **11** can be separated by preparative thin-layer chromatography, for instance, compounds *E*-**11m** and *Z*-**11m** (Table 3, entry 9 and Supporting Information File 1).

The *E*/*Z*-stereochemistry of compounds **11** was determined on the basis of the values of spin–spin interaction constants of vinyl protons, which were 13–14 Hz for *Z-*isomers and 17–18 Hz for *E*-isomers (see Supporting Information File 1).

Reactions of allene **1a** with strongly donating arenes, 1,3,5-trimethylbenzene (mesitylene), 1,2,4-trimethylbenzene (pseudocumene), phenol, thiophenol, 1,3-dimethoxybenzene, 1,4-dimethoxybenzene, and other arenes, such as 1,2-dichlorobenzene, 1,4-dibromobenzene, gave rise to complex mixtures of oligomeric compounds.

**Scheme 5:**
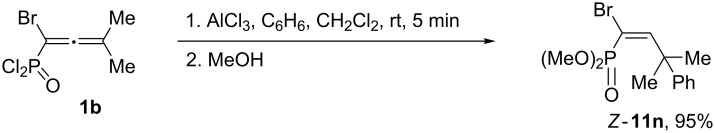
AlCl_3_-promoted hydroarylation of allene **1b** by benzene leading to alkene *Z***-11n**.

In the same reaction with benzene, allene **1b** afforded alkene *Z*-**11n** in high yield (Scheme 5).

**Scheme 6:**
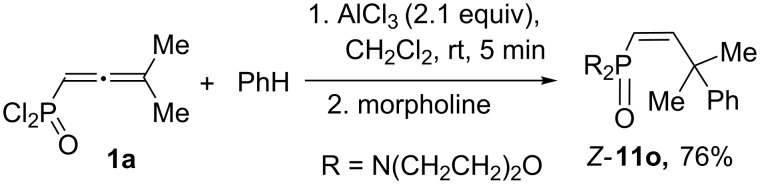
Reaction of allene **1a** with benzene under the action of AlCl_3_ followed by quenching of the reaction mixture with morpholine leading to amide *Z*-**11o**.

The use of morpholine for quenching of the superacidic reaction mixture gave amide *Z*-**11o** in the reaction of **1a** with benzene (Scheme 6).

**Scheme 7:**
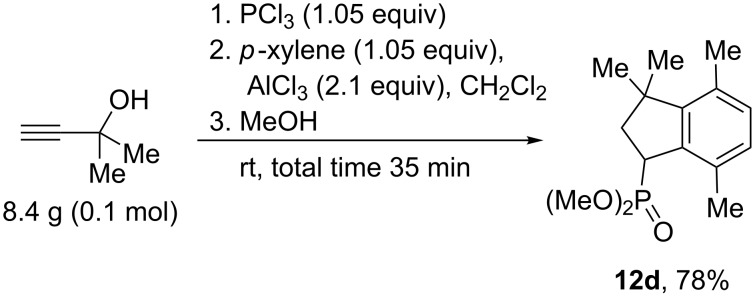
Multigram-scale one-pot synthesis of indane **12d** from 2-methylbut-3-yn-2-ol.

We also conducted a large-scale one-pot synthesis of indane **12d** starting from 2-methylbut-3-yn-2-ol (Scheme 7, see procedure in Supporting Information File 1). The first stage of this procedure gave allene **1a**, which was dissolved in CH_2_Cl_2_ and subjected to reaction with *p*-xylene under the action of AlCl_3_. Finally, methanolysis of the reaction mixture resulted in the formation of indane **12d** in a total yield of 78%.

**Figure 3:**
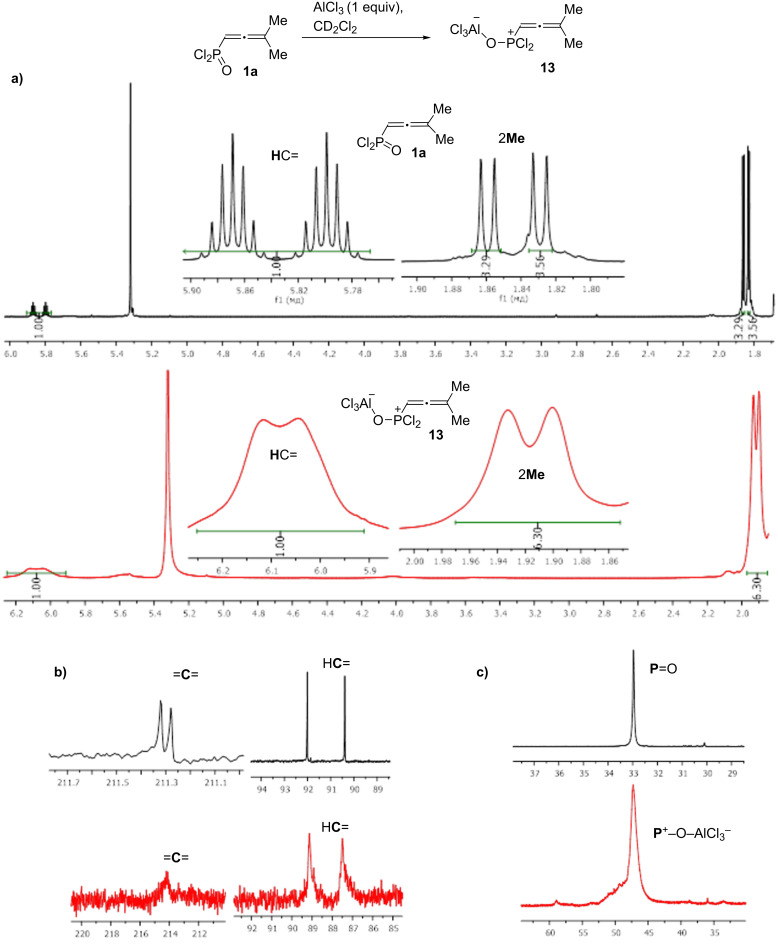
NMR spectra of starting allene **1a** (black) and its complex with 1 equivalent of AlCl_3_ **13** (red) in CD_2_Cl_2_ at room temperature: (a) ^1^H NMR, (b) ^13^C NMR (selected signals, doublets due to ^13^C–^31^P spin–spin interaction), (c) ^31^P NMR.

To elucidate the reaction mechanism additional experiments were conducted. First of all, alkenes **11** were subjected to the action of five-fold excess of AlCl_3_ at room temperature or elevated temperature. However, no formation of indanes **12** was detected. Then we carried out an NMR study to catch the reaction intermediates. Upon mixing of allene **1a** with 1 equivalent of AlCl_3_ in CD_2_Cl_2_ in an NMR tube at room temperature, a yellow solution was formed, which was most likely a complex of **1a** with AlCl_3_, which is coordinated onto oxygen of the P=O group. The comparison of ^1^H, ^13^C, and ^31^P NMR spectra of starting **1a** and its complex with AlCl_3_ **13** is presented in Figure 3 (see full spectral data in Supporting Information File 1). It is clear that the complex formation led to significant broadening of NMR spectral lines and, mainly, a downfield shift of the corresponding signals, due to large positive charge on the phosphorus atom. This solution was stable for a long time (several days) and complex **13** was not converted into other compounds. It must be reminded here, that allene **1a** did not react with benzene under the action of 1 equivalent of AlCl_3_ (see Table 2, entry 3).

**Scheme 8:**
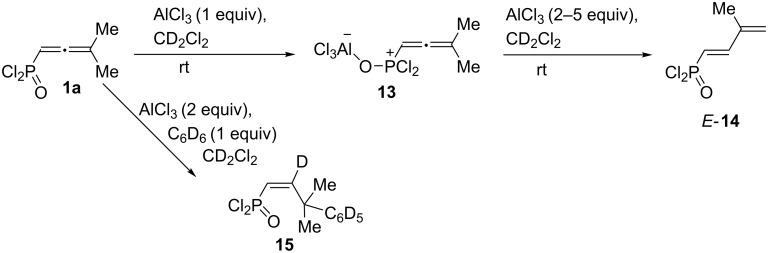
^1^H, ^13^C, and ^31^P NMR monitoring of AlCl_3_-promoted reactions of allene **1a** leading to compounds *E*-**14** or **15** at room temperature.

Addition of more than 1 equivalent of AlCl_3_ (2–5 equivalents) to a solution of **1a** in CD_2_Cl_2_ in an NMR tube resulted in an immediate formation of diene *E*-**14** as a part of a complex mixture (Scheme 8, see Supporting Information File 1 for NMR). Compare with the same transformations of **1a** followed by hydrolysis of the reaction mixture affording a mixture of alcohol *Z*-**9** and diene *E*-**10a** (Table 2, entries 1 and 2). The formation of compound **14** in an NMR monitoring experiment may also indicate that alcohol *Z*-**9** is formed upon hydrolysis of allene **8** (Table 2, entries 1 and 2). Reaction of allene **1a** with deuterobenzene C_6_D_6_ (1 equivalent) under the action of AlCl_3_ (2 equivalents) in CD_2_Cl_2_ in an NMR tube gave alkene **15** (Scheme 8) analogously to the formation of alkenes **11** (Table 3).

Thus, the different reactivity of these particular dichlorophosphorylallenes under the action of Brønsted or Lewis acids can be explained by involvement of the P=O group in intra- or intermolecular interactions at the formation of cationic intermediates. Strong coordination of Lewis acid AlCl_3_ with the P=O group completely deactivates it for further intramolecular reactions (Figure 3, Table 2). Despite solvation in the Brønsted superacid TfOH, the P=O group takes part in intramolecular cyclization into oxaphospholium ions (Table 1). These two different types of reaction intermediates, generated from such allenes in Brønsted and Lewis acids, lead to various reaction products.

**Scheme 9:**
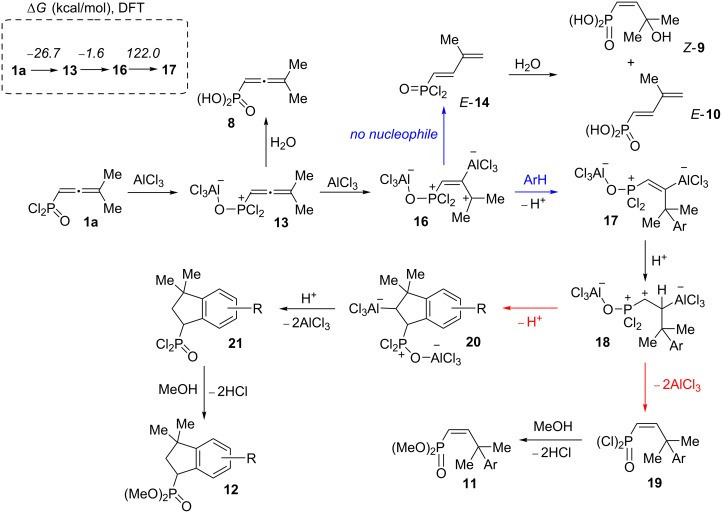
Plausible reaction mechanism A for the formation of compounds **9**, **10**, **11**, **12** from aillene **1a** involving AlCl_3_ attack on the central atom of allenic complex **13**.

**Scheme 10:**
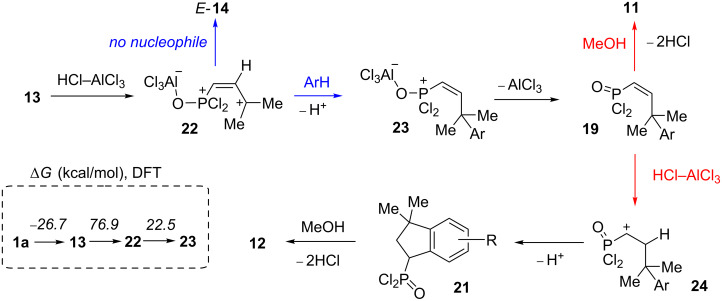
Plausible reaction mechanism B of formation of compounds **11**, **12** from allene **1a** involving HCl–AlCl_3_ attack on the central atom of allenic complex **13**.

Based on the data obtained, one may propose a plausible mechanism A for the transformation of allene **1a** in the presence of AlCl_3_ (Scheme 9). When the first equivalent of AlCl_3_ is added to allene **1a**, adduct **13** is formed as a result of electrophilic attack of AlCl_3_ on the oxygen atom. The second equivalent of AlCl_3_ is coordinated to the central atom of the allene system of the complex **13** and gives intermediate **16**. The latter, in the absence of nucleophiles (arene molecules), undergoes deprotonation from the methyl group affording butadiene **14**. Hydrolysis of the latter resulted in compounds *Z*-**9** and *E-***10a**. Whereas, in the presence of an arene, cation **16** reacts with it leading to species **17**. The latter can be protonated with the formation of cation **18**. This species may react in two different ways. The first option is it could lead to alkene **19** and finally to compounds **11** upon methanolysis of the reaction mixture. An alternative pathway for species **18** is cyclization into indane structure **20**, which is further transformed into **21** and **12**. At the same time, an alternative mechanism B, involving the formation of the protic superacid HCl–AlCl_3_ and its participation in the observed reaction should be considered (Scheme 10). The required catalytic amount of such superacid may be formed due to the presence of traces of HCl (byproduct in acetylene–allene rearrangement step) in the reaction mixture. Next, the protonaton of complex **13** occurs, leading to allylic cation **22**. As analogue of cation **16** (Scheme 9), the latter can interact with arenes giving hydroarylated complex **23**. Consequently, it eliminates AlCl_3_ and is transformed into P(O)Cl_2_ alkene **19**. The latter can further undergo a protonaton–cyclization sequence (alkene **19**→cation **24**→P(O)Cl_2_ indane **21**). Target P(O)OMe_2_ alkenes **11** and indanes **12** are formed during methanolysis of **19** and **21** consequently.

In accordance with both mechanisms A and B, yields of indanes **12** should be increased for substrates having electron-donating groups, Ar. Indeed, the highest yields of indanes **12** were achieved for the reactions of allene **1a** with the electron-rich arenes toluene and xylenes (Table 3, entries 2–5).

We carried out a DFT study [at the B3LYP/6-311+G(2d,2p) level of theory] for the observed AlCl_3_-involved reactions (Scheme 9, Scheme 10, Table 4 and Supporting Information File 1 for details of DFT calculations). First, the thermochemistry (Δ*G* of reaction) for selected transformations (**1a**→**13**→**16**→**17** for mechanism A, **1a**→**13**→**22**→**23** for mechanism B) was explored. Formation of complex **13** from allene **1a** and AlCl_3_ is exergonic (−26.7 kcal/mol) and thermodynamically favorable. The arylation stage for mechanism A (**16**→**17**) is significantly less endergonic (22.6 kcal/mol) than that in mechanism **B** (**22**→**23**, 122.0 kcal/mol, Scheme 9). At the same time, formation of allylic cation **22** (mechanism B) is accompanied by positive changes in Δ*G* (76.9 kcal/mol), whereas its analogue species **16** formed with slightly negative ΔG (−1.6 kcal/mol, Scheme 10).

**Table 4:** Comparison of selected electronic characteristics of species **16** (mechanism A) and species **22** (mechanism B) derived from allene **1a**.

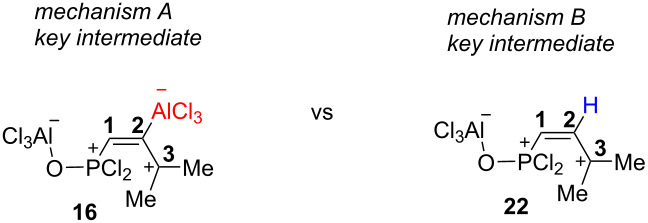							

Species	ω,^a^ eV	q (P),^b^ e	q (C^1^),^b^ e	q(C^3^),^b^ e	k_LUMO_(P),^c^ %	k_LUMO_(C^1^),^c^ %	k_LUMO_(C^3^),^c^ %

**16**	3.42	1.78	-0.64	**0.11**	16.6	3.2	**32.1**
**22**	17.1	1.73	-0.52	**0.44**	0.73	7.4	**56.6**

^a^Global electrophilicity index ω = (*E*_HOMO_ + *E*_LUMO_)^2^/8 (*E*_LUMO_ − *E*_HOMO_); ^b^natural charges; ^c^contribution of atomic orbital into the molecular orbital.

**Figure 4:**
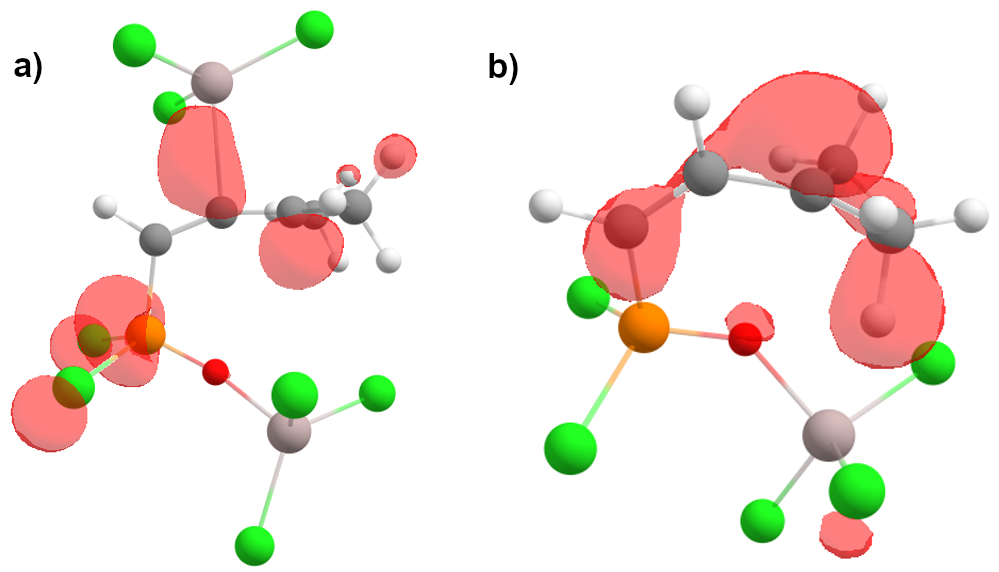
Visualization of LUMO, only positive values are shown, isosurface value 0.043: (a) species **16**, (b) species **22**.

Next, we compared electronic characteristics (global electrophilicity indexes ω, natural charges (NBO) and atomic orbital contributions into LUMO) of species **16** and **22** as key intermediates from mechanisms A and B. The calculations reveal that both charge and orbital factors coincide in electrophilic reactivity of carbon C3 in species **16**, **22** (Scheme 9 and Scheme 10). At the same time, the carbon C3 in C-protonated intermediate **22** bears a more positive charge (0.44 e) and gives a rather big contribution into LUMO (56.6%) compared to that of **16** (0.11 e, 32.1%). Also, species **22** is five times more electrophilic than **16** according to values of ω. Visualizations of the LUMO for **16** and **22** are shown on Figure 4.

## Conclusion

Transformations of various phosphorylallenes under the action of strong Brønsted or Lewis acids were studied. These allenes showed different reactivity depending on the type of the acid. In the Brønsted superacid TfOH, the allenes were transformed into oxophospholium cations. Hydrolysis (or morpholinolysis) of these species afforded a series of phosphorous-containing compounds, cyclic phosphoric acids and their derivatives, and other substances. Contrarily, reactions of dichlorophosphorylallenes with the Lewis acid AlCl_3_ proceeded through the formation of non-cyclic intermediates. Hydrolysis of the latter afforded phosphorylallyl alcohols and butadienes. For the first time, the intermolecular hydroarylation of the allene system of dichlorophosphorylallenes by arenes under the action of AlCl_3_ was achieved. This reaction gave rise to phosphoryl-substituted alkenes and indanes. The intermediates of these reactions were investigated by means of NMR and DFT calculations, that shed light on the reaction mechanisms.

## Supporting Information

File 1 Experimental part.
