# Supplementary material for: Different reactivity of phosphorylallenes under the action of Brønsted or Lewis acids: a crucial role of involvement of the P=O group in intra- or intermolecular interactions at the formation of cationic intermediates
Source: Beilstein J Org Chem. 2019 Jul 8;15:1491–504. doi: 10.3762/bjoc.15.151 (PMC6633813; doi:10.3762/bjoc.15.151)
Supplement: File 1 — Experimental part. [file Beilstein_J_Org_Chem-15-1491-s001.pdf]

BEILSTEIN JOURNAL OF ORGANIC CHEMISTRY

Supporting Information

for

Different reactivity of phosphorylallenes under the action of Brønsted or Lewis acids: a crucial role of involvement of the P=O group in intra- or intermolecular interactions at the formation of cationic intermediates

Stanislav V. Lozovskiy, Alexander Yu. Ivanov and Aleksander V. Vasilyev

Beilstein J. Org. Chem. **2019,** *15,* 1491–1504. doi:10.3762/bjoc.15.151

Experimental part

License and Terms: This is a supporting information file under the terms of the Creative Commons Attribution License (http://creativecommons.org/licenses/by/4.0). Please note that the reuse, redistribution and reproduction in particular requires that the authors and source are credited.
The license is subject to the *Beilstein Journal of Organic Chemistry* terms and conditions: (https://www.beilstein-journals.org/bjoc)

Table of contents

I. Experimental section..S2

II. Copies of NMR spectra S12

III. References S138

IV. DFT-calculations..S139

V.X-Ray data..S145

Experimental section

The NMR spectra of solutions of compounds in $CDCl_3$ were recorded at 400, 162 and 100 MHz for ^1H, ^{31}P and ^{13}C NMR spectra, respectively, at 25 °C with a Bruker-400 spectrometer. The solvent residual signal $CDCl_3$ (δ 7.26 ppm) for ^1H NMR spectra and the carbon signal of $CDCl_3$ (δ 77.0 ppm) for ^{13}C NMR spectra were used as references. H_3PO_4 (85%) was used as an external standard in ^{31}P NMR measurements. NMR spectra in TfOH were referenced to the signal of CH_2Cl_2 added as internal standard: δ 5.32 for ^1H NMR spectra and δ 54.0 ppm for ^{13}C NMR spectra, respectively. HRMS was carried out using a Bruker MicroTOF (ESI) instrument. The preparative reactions were monitored by thin layer chromatography carried out on silica gel plates using UV light for detection. Preparative TLC was performed on silica gel 5–40 μm.

DFT calculations. All computations were carried out at the DFT level of theory using functional B3LYP by using GAUSSIAN 2009 program packages [1]. The geometries optimization was performed using the B3LYP basis set (standard 6-311 basis set added with polarization (d, p) and diffuse functions). Inclusion of dispersion was added by using Grimme's D3BJ dispersion correction. Optimizations were performed on all degrees of freedom and gas-phase-optimized structures were verified as true minima with no imaginary frequencies. The Hessian matrix was calculated analytically for the optimized structures in order to prove the location of correct minima and to estimate the thermodynamic parameters. Atomic charges and contributions in LUMO for species **16**, **22** were obtained using NBO analysis.

X-ray diffraction study. A suitable crystal was selected and studied on a Bruker diffractometer for X-ray analysis. The crystal was kept at 100(2) K during data collection. Using Olex2 [2], the structure was solved with the ShelXS [3] structure solution program using Direct Methods and refined with the ShelXL refinement package using Least Squares minimization. CCDC 1870758-**3a**, contains the supplementary crystallographic data, which can be obtained free of charge at www.ccdc.cam.ac.uk/conts/retrieving.htmL or from the Cambridge Crystallographic Data Centre, 12 Union Road, Cambridge CB2 1EZ, UK; Fax: (internat.) + 44-1223-336-033; E-mail: deposit@ccdc.cam.ac.uk.

Initial allenes were obtained according to the literature procedure [4].

The following compounds has spectral data, in accordance with the literature: (3-methylbuta-1,2-dien-1-yl)phosphonic dichloride (**1a**) [5], dimethyl (3-methylbuta-1,2-dien-1-yl)phosphonate (**1g**) [5], (2-propa-1,2-dien-1-yl)phosphonic dichloride (**1k**) [6], (2-buta-1,2-dien-1-yl)phosphonic dichloride (**1l**) [6]**,** 3-bromo-2-hydroxy-5,5-dimethyl-5*H*-1,2-oxaphosphole 2-oxide (**3b**) [7].

P-(3-methyl-2λ^5-buta-1,2-dien-1-yl)dimorpholinophosphine oxide (**1e**). Yellow oil, yield of 99%; **^{1}H NMR** (400 MHz, $CDCl_3$) δ 5.01 – 4.93 (m, 1H), 3.42 (t, *J* = 4.5 Hz, 8H), 2.93 – 2.84 (m, 8H), 1.54 (dd, *J* = 6.4, 3.3 Hz, 1H). **^{13}C NMR** (100 MHz, $CDCl_3$) δ 209.6, 95.6 (d, *J* = 14.8 Hz),

78.7 (d, J = 161.4 Hz), 66.9 (d, J = 5.9 Hz), 44.2, 19.14 (d, J = 6.2 Hz). **^{31}P NMR** (162 MHz, $CDCl_3$) δ 21.71. **HRMS** (ESI): m/z calcd for $C_{13}H_{23}N_2O_3PNa$ $[M+Na]^+$ 309.1338, found 309.1340.

P-(3-Methyl-2λ^5-buta-1,2-dien-1-yl)-*N*,*N*,*N'*,*N`*-tetraethylphosphonic diamide (**1f**)**.** Yellow oil, yield of 45%; **^{1}H NMR** (400 MHz, $CDCl_3$) δ 5.13 – 4.99 (m, 1H), 2.96 – 2.76 (m, 8H), 1.55 (dd, J = 6.2, 3.3 Hz, 6H), 0.92 (t, J = 7.1 Hz, 12H). **^{13}C NMR** (101 MHz, $CDCl_3$) δ 208.3, 94.9 (d, J = 14.7 Hz), 81.6 (d, J = 160.0 Hz), 38.5 (d, J = 4.3 Hz), 19.1 (d, J = 6.2 Hz), 14.0 (d, J = 2.6 Hz). **^{31}P NMR** (162 MHz, $CDCl_3$) δ 25.12. **HRMS** (ESI): m/z calcd for $C_{13}H_{27}N_2OPNa$ $[M+Na]^+$ 281.1753, found 281.1753.

P-(3-Methyl-2λ^5-buta-1,2-dien-1-yl)-*N*,*N`*-diphenylphosphonic diamide (**1j**). White powder, 145°C, yield of 40%; **^{1}H NMR** (400 MHz, $CDCl_3$) δ 7.24 – 7.12 (m, 5H), 7.14 – 7.01 (m, 3H), 7.01 – 6.83 (m, 2H), 5.64 (1H), 5.48 – 5.35 (m, 1H), 1.56 (dd, J = 7.0, 3.1 Hz, 6H). **^{13}CNMR** (101 MHz, $CDCl_3$) δ 210.2, 139.8, 129.2, 122.0, 118.5 (d, J = 6.2 Hz),98.3 (d, J = 12.6 Hz), 81.1 (d, J = 167.9 Hz), 18.8 (d, J = 6.6 Hz). **^{31}P NMR** (162 MHz, $CDCl_3$) δ 7.18. **HRMS** (ESI): m/z calcd for $C_{17}H_{19}N_2OPN$ a$[M+Na]^+$ 321.1127, found 321.1133.

S,S-Di-4-methylphenyl(3-methyl-2λ^5-buta-1,2-dien-1-yl)phosphonodithioate **1h.** Yellow oil, yield of 98%; **^{1}H NMR** (400 MHz, $CDCl_3$) δ 7.56 (dd, J = 8.0, 1.6 Hz, 4H), 7.23 (d, J = 8.0 Hz, 4H), 5.51 (dhept, J = 19.4, 3.1 Hz, 1H), 2.41 (6H), 1.69 (dd, J = 8.8, 3.1 Hz, 6H). **^{13}C NMR** (101 MHz, $CDCl_3$) δ 209.4 (d, J = 2.8 Hz), 139.4 (d, J = 3.0 Hz), 135.5 (d, J = 4.2 Hz), 130.0 (d, J = 2.1 Hz), 123.2 (d, J = 5.8 Hz), 100.4 (d, J = 17.8 Hz), 86.0 (d, J = 112.2 Hz), 21.2, 19.0 (d, J = 7.3 Hz). **^{31}P NMR** (162 MHz, $CDCl_3$) δ 48.65. **HRMS** (ESI): m/z calcd for $C_{19}H_{21}OPS_2Na$ $[M+Na]^+$ 383.0663, found 383.0664.

S,*S*-bis(4-chlorophenyl) (3-methyl-2λ^5-buta-1,2-dien-1-yl)phosphonodithioate (**1i**)**.** Colorless oil, yield of 95%; **^{1}H NMR** (400 MHz, $CDCl_3$) δδ 7.49 (dd, J = 8.5, 1.8 Hz, 4H), 7.32 (d, J = 8.5 Hz, 4H), 5.46 (dhept, J = 20.9, 3.1 Hz, 1H), 1.66 (dd, J = 9.1, 3.1 Hz, 6H).**^{13}C NMR** (101 MHz, $CDCl_3$) δ 209.9 (d, J = 2.8 Hz), 136.7 (d, J = 4.2 Hz), 136.0 (d, J = 3.2 Hz), 129.4 (dd, J = 22.3, 2.1 Hz), 125.0 (d, J = 5.9 Hz), 101.30 (d, J = 18.2 Hz), 85.8 (d, J = 113.4 Hz), 19.1 (d, J = 7.4 Hz). **^{31}P NMR** (162 MHz, $CDCl_3$) δ 47.60. **HRMS** (ESI): m/z calcd for $C_{17}H_{15}Cl_2OPS_2Na$ $[M+Na]^+$ 422.9571, found 422.9573.

(1-Bromo-3-methyl-2-buta-1,2-dien-1-yl)phosphonic dichloride (**1b**)**.** Colorless oil, yield of 95%; **^{1}H NMR** (400 MHz, $CDCl_3$) δ 1.98 (3H), 1.96 (3H). **^{13}C NMR** (101 MHz, $CDCl_3$) δ 205.4 (d, J = 19.3 Hz), 113.8 (d, J = 16.0 Hz), 79.9 (d, J = 204.1 Hz), 19.7 (d, J = 6.6 Hz). **^{31}P NMR** (162 MHz, $CDCl_3$) δ 24.05. **HRMS** (ESI): m/z calcd for $C_{17}H_{15}Cl_2OPS_2Na$ $[M+Na]^+$ 284.8614, found 284.8614.

Procedure for the synthesis of compounds 3, 4, 5 and 6. Allene **1** (0.144 mmol) was added to a solution of 21.6 mg (0.144 mmol) TfOH in CH_2Cl_2 (1 mL) with vigorous stirring. After 3 min the mixture was quenched with excess of water (morpholine was used instead of water for the

preparation of amides **6a**,**b**). The mixture was diluted with CH_2Cl_2 (25 mL). The organic phase was washed with water, a saturated aqueous solution of $NaHCO_3$, water, and dried with Na_2SO_4. The solvent was distilled off under reduced pressure to give pure reaction products.

2-Hydroxy-5,5-dimethyl-5*H*-1,2-oxaphosphole 2-oxide (**3a**)**.** Dark green crystals, yield of 99%. See SI for X-Ray data. **^{1}H NMR** (400 MHz, $CDCl_3$) δ 10.17 (1H), 6.93 (dd, *J* = 47.8, 8.3 Hz, 1H), 6.07 (dd, *J* = 31.8, 8.3 Hz, 1H), 1.48 (6H). **^{13}C NMR** (101 MHz, $CDCl_3$) δ 156.9 (d, *J* = 16.1 Hz), 119.2 (d, *J* = 141.8 Hz), 89.9 (d, *J* = 10.3 Hz), 27.9 (d, *J* = 3.3 Hz). **^{31}P NMR** (162 MHz, $CDCl_3$) δ 44.51. **HRMS** (ESI): m/z calcd for $C_5H_8O_3P$ $[M-H]^-$ 147.0211, found 147.0221.

3-Bromo-2-hydroxy-5,5-dimethyl-5*H*-1,2-oxaphosphole 2-oxide (**3b**). Dark crystals, yield of 98% [8].

(*Z*)-(3-Hydroxy-3-methylbut-1-en-1-yl)dimorpholinophosphine oxide (**4a**)**.** Yellow oil, yield of 90%; **^{1}H NMR** (400 MHz, $CDCl_3$) δ 7.57 (dd, *J* = 49.3, 8.2 Hz, 1H), 7.00 (dd, *J* = 33.8, 8.2 Hz, 1H), 3.70 (t, *J* = 4.3 Hz, 8H), 3.33 – 3.22 (m, 8H), 2.46 (OH), 1.60 (6H). **^{13}C NMR** (101 MHz, $CDCl_3$) δ 163.9, 112.3 (d, *J* = 129.6 Hz), 92.8 (d, *J* = 10.9 Hz), 66.5 (d, *J* = 4.2 Hz), 45.2 (d, *J* = 1.6 Hz), 27.3 (d, *J* = 1.3 Hz). **^{31}P NMR** (162 MHz, $CDCl_3$) δ 63.26. **HRMS** (ESI): m/z calcd for $C_{13}H_{25}N_2O_4PNa$ $[M+Na]^+$ 327.1444, found 327.1448.

(*Z*)-(3-Hydroxy-3-methylbut-1-en-1-yl-*N*,*N*,*N*`,*N*`-tetraethylphosphonic diamide (**4b**)**.** Colorless oil, yield of 85%; **^{1}H NMR** (400 MHz, $CDCl_3$) δ 7.63 (dd, *J* = 47.2, 8.4 Hz, 1H), 6.64 (dd, *J* = 35.7, 8.4 Hz, 1H), 3.21 – 3.08 (m, 8H), 2.29 (s, 1H), 1.59 (6H), 1.19 (t, *J* = 7.1 Hz, 12H). **^{13}C NMR** (101 MHz, $CDCl_3$) δ 163.2 (d, *J* = 11.8 Hz), 112.1 (d, *J* = 129.2 Hz), 92.9 (d, *J* = 9.8 Hz), 40.4 (d, *J* = 5.4 Hz), 26.9 (d, *J* = 1.5 Hz), 13.8 (d, *J* = 2.1 Hz). **^{31}P NMR** (162 MHz, $CDCl_3$) δ 66.96. **HRMS** (ESI): m/z calcd for $C_{13}H_{29}N_2O_2P$ $[M+Na]^+$ 299.1858, found 299.1854.

5,5-Dimethyl-1-phenyl-2-(phenylamino)-1,5-dihydro-1,2-azaphosphole 2-oxide (**5**)**.** Gray oil, yield of 55%; **^{1}H NMR** (400 MHz, D_2O) δ7.98 – 7.91 (m, 1H), 7.85 – 7.77 (m, 1H), 6.74 (d, *J* = 14.7 Hz, 1H),6.64 (dd, *J* = 81.5, 14.7 Hz, 1H), 6.57 (dd, *J* = 45.7, 14.5 Hz, 1H),1.90 (1H). **^{31}P NMR** (162 MHz, $CDCl_3$) δ 9.38. **HRMS** (ESI): m/z calcd for $C_{17}H_{19}N_2NaOP$ $[M+Na]^+$ 321.1133, found 321.1135.

Cations A–**H** were generated through the protonation of allenes **1a**–**h** using TfOH directly in NMR tubes.

2,2-Dichloro-5,5-dimethyl-2,5-dihydro-1,2-oxaphosphol-2-ium (**A**)**.** **^{1}H NMR** (400 MHz, TfOH) δ 8.11 (dd, *J* = 68.3, 8.3 Hz, 1H), 7.00 (dd, *J* = 49.4, 8.3 Hz, 1H), 1.90 (6H). **^{13}C NMR** (101 MHz, TfOH) δ 169.9 (d, *J* = 14.3 Hz), 116.4 (d, *J* = 111.3 Hz), 110.8 (d, *J* = 10.8 Hz), 27.0. **^{31}P NMR** (162 MHz, TfOH) δ 97.04.

5,5-Dimethyl-2,2-dimorpholino-2,5-dihydro-1,2-oxaphosphol-2-ium (**C**)**.** **^{1}H NMR** (400 MHz, TfOH) δ 7.89 (dd, *J* = 49.2, 8.3 Hz, 1H), 6.45 (dd, *J* = 36.9, 8.3 Hz, 1H), 4.51 – 4.39 (m, 8H), 3.72 (m, 8H), 1.79 (6H). **^{13}C NMR** (101 MHz, TfOH) δ 170.3 (d, *J* = 12.3 Hz), 109.3 (d, *J* =

131.9 Hz), 98.9 (d, J = 10.0 Hz), 72.2, 45.15, 27.49.^{31}**P NMR** (162 MHz, TfOH) δ 64.33. ^{15}**N NMR** (from N-H HMBC) (400 MHz, TfOH) δ 41.53.

2,2-Bis(diethylamino)-5,5-dimethyl-2,5-dihydro-1,2-oxaphosphol-2-ium (**D**). ^{1}**H NMR** (400 MHz, TfOH) δ 7.94 (dd, J = 49.3, 8.2 Hz, 1H), 6.56 (dd, J = 38.8, 8.3 Hz, 1H), 3.64 – 3.32 (m, 8H), 1.81 (6H), 1.57 – 1.20 (m, 12H). ^{13}**C NMR** (101 MHz, TfOH) δ 170.0, 109.1 (d, J = 125.6 Hz), 102.0, 44.4, 27.0, 13.2. ^{31}**P NMR** (162 MHz, TfOH) δ 70.79.

5,5-Dimethyl-2,2-bis(phenylamino)-2,5-dihydro-1,2-oxaphosphol-2-ium (**E1**). ^{1}**H NMR** (400 MHz, TfOH) δ 7.97 – 6.96 (m, 11H), 6.42 (dd, J = 37.1, 7.8 Hz, 1H), 1.42 (6H). ^{13}**C NMR** Selected signals (101 MHz, TfOH) δ 168.35 (d, J = 12.8 Hz), 134.51, 131.22, 127.19 (d, J = 3.6 Hz), 111.65 (d, J = 137.5 Hz), 96.12 (d, J = 9.2 Hz), 26.80. ^{31}**P NMR** (162 MHz, TfOH) δ 52.87.

5,5-Dimethyl-1-phenyl-2-(phenylamino)-2,5-dihydro-1*H*-1,2-azaphosphol-1-ium (**E2**). ^{1}**H NMR** (400 MHz, TfOH) δ 7.87 (dd, J = 50.2, 8.9 Hz, 1H), 7.75 – 7.40 (m, 10H), 6.68 (dd, J = 34.2, 8.9 Hz, 1H), 1.44 (3H), 1.30 (3H). ^{13}**C NMR** Selected signals (101 MHz, TfOH) δ 169.33 (d, J = 14.8 Hz), 132.38, 132.03, 111.94 (d, J = 146.5 Hz), 70.36 (d, J = 7.5 Hz), 26.07, 24.02. ^{31}**P NMR** (162 MHz, TfOH) δ 43.02.

Cation **E4.** ^{1}**H NMR** (400 MHz, TfOH) δ8.08-8.04(m, 1H), 7.80 – 7.7 (m, 3H), 7.65 – 7.60 (m, 2H), 7.55 – 7.50 (m, 4H),7.26 (dd, J = 56.0, 12.0Hz, 1H),7.26 (dd, J = 12.0, 12.0Hz, 1H), 1.97 (6H). ^{13}**C NMR** (101 MHz, TfOH) δ157.1, 133.9, 132.5, 132.5, 132.2, 130.5, 129.2, 125.8, 124.1, 116.2 (d, J = 195.4 Hz), 70.5 (d, J = 7.9 Hz), 25.20. ^{31}**P NMR** (162 MHz, TfOH) δ 24.06.

5,5-Dimethyl-2,2-bis(4-methylphenylthio)-2,5-dihydro-1,2-oxaphosphol-2-ium **F.** ^{1}**H NMR** (400 MHz, TfOH) δ 7.53 – 7.50 (m, 4H), 7.48 – 7.35 (m, 4H), 7.26 (dd, J = 54.0, 7.9 Hz, 1H), 6.33 (dd, J = 45.5, 7.9 Hz, 1H), 2.44 (d, J = 2.7 Hz, 6H), 0.96 (6H). ^{13}**C NMR** (101 MHz, TfOH) δ 167.1 (d, J = 10.0 Hz), 145.9 (d, J = 3.8 Hz), 138.2 (d, J = 3.5 Hz), 133.0, 116.3 (d, J = 7.6 Hz), 113.1 (d, J = 82.7 Hz), 102.6, 26.4, 21.3. ^{31}**P NMR** (162 MHz, TfOH) δ 115.37.

2,2-Bis((4-chlorophenyl)thio)-5,5-dimethyl-2,5-dihydro-1,2-oxaphosphol-2-ium (**G**). ^{1}**H NMR** (400 MHz, TfOH) δ 7.54 – 7.48 (m, 5H), 7.44 – 7.38 (m, 5H), 7.26 (dd, J = 54.0, 7.9 Hz, 1H), 6.33 (dd, J = 45.5, 7.9 Hz, 1H), 2.44 (d, J = 2.7 Hz, 6H), 0.96 (6H). ^{13}**C NMR** (101 MHz, TfOH) δ 167.1 (d, J = 10.0 Hz), 145.9, 138.2 (d, J = 3.5 Hz), 133.0, 116.3 (d, J = 7.6 Hz), 113.1 (d, J = 82.7 Hz), 102.6, 26.4, 21.3. ^{31}**P NMR** (162 MHz, TfOH) δ 115.37.

2,2-Dimethoxy-5,5-dimethyl-2,5-dihydro-1,2-oxaphosphol-2-ium **H.** ^{1}**H NMR** (400 MHz, $CDCl_3$) δ 8.05 (dd, J = 54.6, 8.5 Hz, 1H), 6.41 (dd, J = 35.8, 8.5 Hz, 1H), 4.27 (d, J = 12.4 Hz, 6H), 1.89 (6H). ^{13}**C NMR** (101 MHz, $CDCl_3$) δ 171.8 (d, J = 14.4 Hz), 107.3 (d, J = 159.4 Hz), 97.7 (d, J = 13.2 Hz), 59.5 (d, J = 6.6 Hz), 26.8. ^{31}**P NMR** (162 MHz, $CDCl_3$) δ 57.82.

3-Bromo-2,2-dichloro-5,5-dimethyl-2,5-dihydro-1,2-oxaphosphol-2-ium (**B**). ^{1}**H NMR** (400 MHz, TfOH) δ 8.13 (d, J = 55.1 Hz, 1H), 2.00 (6H). ^{13}**C NMR** (101 MHz, TfOH) δ 165.8 (d, J =

33.2 Hz), 113.6 (d, J = 5.4 Hz), 104.9 (d, J= 137.0 Hz), 27.3 (d, J = 1.4 Hz).^{31}P NMR (162 MHz, TfOH) δ 87.82.

Protocol for the one-pot preparation of amides 6a,b

PCl_3 (0.105 mol) was added dropwise to 0.1 mol of the corresponding propargylic alcohol placed in owen-dried one-necked flask with vigorous stirring at room temperature. After 2 min, the evolving gas was driven off by a water-jet pump. Then, acid (0.105 mol of TfOH or H_2SO_4) was added dropwise during 2 min. After 5 min of stirring, the flask was placed in cooling bath (−5 to 0 °C) and 0.21 mol of morpholine was carefully added, avoiding heating the reaction mixture. After 3 min the mixture was quenched with excess of water. The mixture was diluted with CH_2Cl_2 (250 ml). The organic phase was washed with water, a saturated aqueous solution of $NaHCO_3$, water, and dried with Na_2SO_4. The solvent was distilled off under reduced pressure to give pure reaction products. Yields: with TfOH: **6a** - 90%, **6b** - 77%; with H_2SO_4: **6a** - 76%, **6b** - 60%.

5,5-Dimethyl-2-morpholino-5*H*-1,2-oxaphosphole 2-oxide (**6a**). Yellow oil; **^{1}H NMR** (400 MHz, $CDCl_3$) δ 6.91 (dd, J = 45.3, 8.1 Hz, 1H), 5.90 (dd, J = 31.3, 8.1 Hz, 1H), 3.72 – 3.49 (m, 4H), 3.08 (dd, J = 7.9, 4.8 Hz, 2H), 1.47 (3H), 1.38 (3H). **^{13}C NMR** (101 MHz, $CDCl_3$) δ 156.3 (d, J = 13.8 Hz), 116.9 (d, J = 147.9 Hz), 84.8 (d, J = 9.0 Hz), 81.6, 67.3 (d, J = 4.7 Hz), 43.8 (d, J = 2.0 Hz), 26.4 (d, J = 4.0 Hz). ^{31}P NMR (162 MHz, TfOH) δ 40.66. **HRMS** (ESI): m/z calcd for $C_9H_{16}NNaO_3P$ $[M+Na]^+$ 240.0765, found 240.0768.

3-Bromo-5,5-dimethyl-2-morpholino-5*H*-1,2-oxaphosphole 2-oxide (**6b**). Yellow oil; **^{1}H NMR** (400 MHz, $CDCl_3$) δ 6.95 (d, J = 34.3 Hz, 1H), 3.66-3.60 (m, 4H), 3.21 – 3.00 (m, 4H), 1.54 (3H), 1.46 (3H). **^{13}C NMR** (101 MHz, $CDCl_3$) δ 152.5 (d, J = 27.1 Hz), 110.4 (d, J = 166.4 Hz), 85.2 (d, J = 4.2 Hz), 67.2 (d, J = 4.4 Hz), 43.2 (d, J = 2.2 Hz), 26.8 (d, J = 4.1 Hz).**^{31}P NMR** (162 MHz, $CDCl_3$) δ 30.48. **HRMS** (ESI): m/z calcd for $C_9H_{15}BrNNaO_3P$ $[M+Na]^+$ 317.9871, found 317.9871.

Procedure for the synthesis of compounds *Z*-9, *E*-10a,b, *Z*-11a.

$AlCl_3$ (2 equiv) was added to a solution of 0.144 mmol of allene (**1a** and 1.05 equiv of benzene for the synthesis of *Z*-**11a**) in CH_2Cl_2 (1 mL) with vigorous stirring. After 5 min the mixture was quenched with excess of water (methanol for *E*-**10b**) The mixture was diluted with EtOAc (10 mL). The organic phase was washed with water, a saturated aqueous solution of $NaHCO_3$, water, and dried with Na_2SO_4. The solvent was distilled off under reduced pressure and the residue was subjected to preparative TLC on silica gel using hexanes/ethyl acetate mixtures as eluent.

(*Z*)-(3-Methyl-3-phenylbut-1-en-1-yl)phosphonic acid (*Z*-**11a**). Colorless oil, yield of 90%; **^{1}H NMR** (400 MHz, $CDCl_3$) δ 7.46 – 7.33 (m, $4H_{arom}$+2OH), 7.32 – 7.25 (m, 1H), 6.84 (dd, J = 77.2, 13.9 Hz, 1H), 6.15 (dd, J = 34.3, 13.9 Hz, 1H), 1.75 (6H). **^{13}C NMR** (101 MHz, $CDCl_3$) δ 164.0, 146.9, 128.5, 127.5 (d, J = 165.4 Hz), 126.4, 123.5 (d, J = 143.2 Hz), 43.0 (d, J = 8.4 Hz),

29.4. **^{31}P NMR** (162 MHz, $CDCl_3$) δ 28.47. **HRMS**(ESI): m/z calcd for $C_{11}H_{16}O_3P$ $[M+H]^+$ 227.0837, found 227.0840.

Procedure for the reaction of allenes 1a,b with arenes ArH. Synthesis of compounds 11 and 12. $AlCl_3$ (2.1 equiv or 3.1 equiv in case of reaction with veratrol) was added to a solution of 0.144 mmol of allene **1a,b** and arene (1.05 equiv) in CH_2Cl_2 (1 mL) with vigorous stirring. After 5 min the mixture was quenched with excess of methanol (morpholine for synthesis of *Z*-**11o**). The mixture was diluted with CH_2Cl_2 (25 mL). The organic phase was washed with water, a saturated aqueous solution of $NaHCO_3$, water, and dried with Na_2SO_4. The solvent was distilled off under reduced pressure and the residue was subjected to preparative TLC on silica gel using hexanes/ethyl acetate mixtures as eluent. Yields of compounds **5** and **6** are given in Table 2, and Schemes 1 and 2.

Large scale procedure for the synthesis of indane 12e.

PCl_3 (9.2ml, 0.105 mol) was added dropwise to 8.4 g (0.1 mol) of 2-methylbut-3-yn-2-ol placed in owen-dried one-necked flask with vigorous stirring at room temperature. After 2 min, the evolving gas was driven off by a water-jet pump. Then, 100 mL of CH_2Cl_2, 11.15 g (0.105 mol) of *p*-xylene and 28 g (0.21 mol, by parts) of $AlCl_3$ were consequently added. After 5 min of stirring, methanol (8 g, 0.25 mol) was added very carefully. After 1 min, the organic phase was washed with water, a saturated aqueous solution of $NaHCO_3$, water, and dried with Na_2SO_4. The solvent was distilled off under reduced pressure to give pure indane **6e**. Yield of 78%.

Dimethyl (*Z*)-(3-methyl-3-phenylbut-1-en-1-yl)phosphonate (*Z*-**11b).** Colorless oil, yield of 88%; **^{1}H NMR** (400 MHz, $CDCl_3$) δ 7.40 – 7.35 (m, 2H), 7.34 – 7.28 (m, 2H), 7.24 – 7.10 (m, 1H), 6.66 (dd, *J* = 54.7, 14.7 Hz, 1H), 5.58 (not resolved dd, *J* = 14.7, 14.4 Hz, 1H), 3.59 (d, *J* = 11.2 Hz, 6H), 1.63 (6H). **^{13}C NMR** (101 MHz, $CDCl_3$) δ 162.3 (d, *J* = 3.7 Hz), 148.2, 128.2, 126.2, 126.2 (d, *J* = 13.6 Hz), 113.6 (d, *J* = 188.4 Hz), 52.0 (d, *J* = 6.2 Hz), 42.7 (d, *J* = 6.9 Hz), 28.9. **^{31}P NMR** (162 MHz, $CDCl_3$) δ 18.52. **HRMS**(ESI): m/z calcd for $C_{13}H_{19}O_3PNa$ $[M+Na]^+$ 277.0964, found 277.0964.

Mixture of dimethyl (*Z*)-(3-methyl-3-(4-methylphenyl)but-1-en-1-yl)phosphonate (*Z*-**11c**), dimethyl (*Z*)-(3-methyl-3-(2-methylphenyl)but-1-en-1-yl)phosphonate (*Z*-**11d**), dimethyl (3,3,6-trimethyl-2,3-dihydro-1*H*-inden-1-yl)phosphonate (**12a**).

Z-**11c**, selected signals: **^{1}H NMR** (400 MHz, $CDCl_3$) δ 7.29 (d, *J* = 7.9 Hz, 2H), 7.15 (d, *J* = 7.9 Hz, 2H), 6.65 (dd, *J* = 54.8, 14.7 Hz, 1H), 5.58 (dd, *J* = 14.7, *J* = 14.7 Hz, 1H), 3.64 (d, *J* = 11.2 Hz, 6H), 2.34 (3H), 1.64 (6H). **^{13}C NMR** (101 MHz, $CDCl_3$) δ 162.4 (d, *J* = 3.8 Hz), 145.2 (d, *J* = 1.5 Hz), 135.6, 128.9, 126.1, 113.2 (d, *J* = 188.3 Hz), 52.0 (d, *J* = 6.1 Hz), 42.5 (d, *J* = 6.8 Hz), 28.8, 20.9. **^{31}P NMR** (162 MHz, $CDCl_3$) δ 18.72.

Z-**11d**, selected signals: **^{1}H NMR** (400 MHz, $CDCl_3$) δ 6.67 (dd, *J* = 54.8, 14.7 Hz, 3H), 5.60 (dd, *J* = 14.7 Hz, 14.7 Hz, 1H), 2.37 (s, 3H). **^{13}C NMR** (101 MHz, $CDCl_3$) δ 162.4 (d, *J* = 3.8 Hz),

113.4 (d, *J* = 188.3 Hz), 52.0 (d, *J* = 6.7 Hz), 42.8 (d, *J* = 4.6 Hz), 29.6, 21.6. **^{31}P NMR** (162 MHz, $CDCl_3$) δ 18.67.

12a, selected signals: **^{1}H NMR** (400 MHz, $CDCl_3$) δ 3.79 (d, *J* = 10.7 Hz, 3H), 3.73 (d, *J* = 10.4 Hz, 3H), 2.55 – 2.40 (m, 1H), 2.37 (s, 3H), 2.32 – 2.17 (m, 2H), 1.41 (3H), 1.21 (3H). **^{13}C NMR** (101 MHz, $CDCl_3$) δ 148.3 (d, *J* = 1.4 Hz), 137.5 (d, *J* = 2.3 Hz), 133.6 (d, *J* = 4.9 Hz), 53.2 (d, *J* = 6.7 Hz), 52.6 (d, *J* = 7.0 Hz), 42.3 (d, *J* = 4.6 Hz), 39.8, 28.7, 21.4. ^{31}P NMR (162 MHz, $CDCl_3$) δ 31.93. **HRMS**(ESI) (for mixture of isomers): m/z calcd for $C_{14}H_{21}NaO_3P$ $[M+Na]^+$ 291.1126, found 291.1126.

Mixture of dimethyl (*Z*)-(3-(3,4-dimethylphenyl)-3-methylbut-1-en-1-yl)phosphonate (***Z*-11e**), dimethyl (3,3,5,6-tetramethyl-2,3-dihydro-1*H*-inden-1-yl)phosphonate (**12b**).

***Z*-11e**: **^{1}H NMR** (400 MHz, $CDCl_3$) δ 7.20 – 7.06 (m, 3H), 6.65 (dd, *J* = 54.8, 14.7 Hz, 1H), 5.59 (dd, *J* = 14.4, 14.4 Hz, 1H), 3.67 (d, *J* = 11.2 Hz, 6H), 2.30 (3H), 2.26 (3H), 1.65 (6H). **^{13}C NMR** (101 MHz, $CDCl_3$) δ 162.6 (d, *J* = 3.6 Hz), 145.7, 136.2, 134.3, 129.5, 127.5, 123.4, 112.9 (d, *J* = 188.5 Hz), 52.0 (d, *J* = 6.1 Hz), 42.5 (d, *J* = 6.9 Hz), 28.7, 19.9, 19.2. **^{31}P NMR** (162 MHz, $CDCl_3$) δ 18.91.

12b: **^{1}H NMR** selected signals (400 MHz, $CDCl_3$) δ 7.31 (d, *J* = 17.4 Hz, 2H), 7.07 – 7.00 (m, 1H), 3.81 (dd, *J* = 10.4, 3.8 Hz, 3H), 3.75 (d, *J* = 10.4 Hz, 3H), 2.28 (3H), 2.19 (3H). **^{13}C NMR** selected signals (101 MHz, $CDCl_3$) δ 145.8, 135.0, 129.0, 126.8, 126.1, 122.5, 42.35 (d, *J* = 3.9 Hz), 29.6, 28.8, 20.3, 19.8. **^{31}P NMR** (162 MHz, $CDCl_3$) δ 32.09. **HRMS** (ESI) (for mixture of isomers): m/z calcd for $C_{15}H_{23}NaO_3P$ $[M+Na]^+$ 305.1283, found 305.1285.

Mixture of dimethyl (*Z*)-(3-(2,4-dimethylphenyl)-3-methylbut-1-en-1-yl)phosphonate (***Z*-5f),** dimethyl (3,3,4,6-tetramethyl-2,3-dihydro-1*H*-inden-1-yl)phosphonate (**6c**).

***Z*-11f**: **^{1}H NMR** selected signals (400 MHz, $CDCl_3$) δ 6.65 (dd, *J* = 55.0, 14.6 Hz, 1H), 5.58 (dd, *J* = 14.6, 14.6 Hz, 1H), 3.66 (d, *J* = 11.3 Hz, 6H), 1.63 (6H).^{31}P NMR (162 MHz, $CDCl_3$) δ 18.86.

12c: **^{1}H NMR** (400 MHz, $CDCl_3$) δ 6.85 (1H), 6.81 (1H), 3.70 (d, *J* = 10.6 Hz, OMe), 3.62 (d, *J* = 10.5 Hz, OMe), 2.42 (ArMe), 2.51 – 2.16 (m, CH_2+CH), 2.32 (d, *J* = 2.0 Hz, ArMe), 1.40 (Me), 1.28 (Me). **^{13}C NMR** (101 MHz, $CDCl_3$) δ 153.4 (d, *J* = 7.4 Hz), 137.7 (d, *J* = 3.7 Hz), 135.0 (d, *J* = 4.1 Hz), 132.4 (d, *J* = 7.3 Hz), 129.5 (d, *J* = 3.5 Hz), 120.9 (d, *J* = 2.8 Hz), 52.8 (d, *J* = 6.9 Hz), 52.3 (d, *J* = 7.2 Hz), 43.4 (d, *J* = 0.6 Hz), 42.4 (d, *J* = 3.7 Hz), 40.1 (d, *J* = 141.1 Hz), 31.4 (d, *J* = 2.9 Hz), 29.7 (d, *J* = 1.6 Hz),, 21.2 (d, *J* = 1.0 Hz), 20.0 (d, *J* = 0.9 Hz). **^{31}P NMR** (162 MHz, $CDCl_3$) δ 32.35. **HRMS** (ESI) (for mixture of isomers): m/z calcd for $C_{15}H_{23}NaO_3P$ $[M+Na]^+$ 305.1283, found 305.1282.

Dimethyl (3,3,4,7-tetramethyl-2,3-dihydro-1*H*-inden-1-yl)phosphonate (**6d**). Orange oil, yield of 95%; **^{1}H NMR** (400 MHz, $CDCl_3$) δ 6.91 (2H), 3.65 (d, *J* = 10.6 Hz, OMe), 3.59 (d, *J* = 10.5 Hz, OMe), 2.51 – 2.11 (m, CH_2+CH), 2.39 (d, *J* = 1.4 Hz, ArMe), 2.36 (ArMe), 1.50 (Me), 1.38 (Me).

^{13}C NMR (101 MHz, $CDCl_3$) δ 149.4 (d, *J* = 7.0 Hz), 136.3 (d, *J* = 7.4 Hz), 133.0 (d, *J* = 4.4 Hz), 131.3 (d, *J* = 3.0 Hz), 131.1 (d, *J* = 3.7 Hz), 128.7 (d, *J* = 3.6 Hz), 52.9 (d, *J* = 7.0 Hz, OMe), 52.4 (d, *J* = 7.2 Hz, OMe), 45.0, 43.9 (d, *J* = 3.9 Hz, CH_2), 40.5 (d, *J* = 140.2 Hz, CH), 30.2 (d, *J* = 3.3 Hz), 27.0 (d, *J* = 2.0 Hz), 19.9 (d, *J* = 1.0 Hz), 18.84.**^{31}P NMR** (162 MHz, $CDCl_3$) δ 32.24. **HRMS**(ESI): m/z calcd for $C_{15}H_{23}NaO_3P$ $[M+Na]^+$ 305.1283, found 305.1287.

Dimethyl (*E*)-(3-(3,4-dimethoxyphenyl)-3-methylbut-1-en-1-yl)phosphonate (*E*-**11g**). Colorless oil, yield of 87%; **^{1}H NMR** (400 MHz, $CDCl_3$) δ 6.96 (dd, *J* = 22.6, 17.4 Hz, 1H), 6.84 (2H), 6.81 (1H), 5.60 (dd, *J* = 19.9, 17.4 Hz, 1H), 3.89 (6H), 3.74 (d, *J* = 11.0 Hz, 6H), 1.47 (6H). **^{13}C NMR** (101 MHz, $CDCl_3$) δ 162.5, 148.7, 147.7, 121.7, 121.7, 118.2, 110.7, 110.5 (d, *J* = 107.3 Hz), 55.9 (d, *J* = 2.9 Hz), 52.3 (d, *J* = 5.8 Hz), 27.7.**^{31}P NMR** (162 MHz, $CDCl_3$) δ 22.52. **HRMS**(ESI): m/z calcd for $C_{15}H_{23}NaO_5P$ $[M+Na]^+$ 337.1181, found 337.1189.

Mixture of dimethyl (*Z*)-(3-(4-fluorophenyl)-3-methylbut-1-en-1-yl)phosphonate (*Z*-**11h**), dimethyl (*Z*)-(3-(2-fluorophenyl)-3-methylbut-1-en-1-yl)phosphonate (*Z*-**11i**).

Z-**11h**: **^{1}H NMR** (400 MHz, $CDCl_3$) δ 7.39 – 7.31 (m, 2H), 7.05 – 6.94 (m, 2H), 6.64 (dd, *J* = 54.6, 14.6 Hz, 1H), 5.59 (dd, *J* = 14.3,14.3 Hz, 1H), 3.62 (d, *J* = 11.2 Hz, 6H), 1.62 (6H). **^{13}C NMR** (101 MHz, $CDCl_3$) δ 174.5, 162.1 (d, *J* = 3.6 Hz), 143.8, 127.9 (d, *J* = 7.9 Hz), 114.7 (d, *J* = 21.0 Hz), 113.8 (d, *J* = 188.7 Hz), 52.0 (d, *J* = 6.2 Hz), 42.2 (d, *J* = 7.0 Hz), 29.2. **^{31}P NMR** (162 MHz, $CDCl_3$) δ 18.26.

Z-**11i: ^{1}H NMR** (400 MHz, $CDCl_3$) δ 7.72 (m, 1H), 7.57 – 7.41 (m, 2H), 7.23 – 7.09 (m, 1H), 6.90 (ddd, *J* = 40.0, 14.5, 3.9 Hz, 1H), 5.57 – 5.47 (dd, *J* = 14.3, 14.3 Hz, 1H), 3.45 (d, *J* = 11.2 Hz, 6H), 1.65 (6H). **^{13}C NMR** (101 MHz, $CDCl_3$) δ 171.1, 162.5, 128.8, 128.1 (d, *J* = 3.6 Hz), 123.5 (d, *J* = 3.2 Hz), 115.6 (d, *J* = 22.9 Hz), 113.4 (dd, *J* = 187.7, 2.7 Hz), 66.1, 51.7 (d, *J* = 6.0 Hz), 39.95(d, *J* = 7.7 Hz), 28.6. **^{31}P NMR** (162 MHz, $CDCl_3$) δ 17.77 (d, *J* = 1.6 Hz). **HRMS** (ESI) (for mixture of isomers): m/z calcd for $C_{13}H_{18}FNaO_3P$ $[M+Na]^+$ 295.0875, found 295.0878.

Mixture of dimethyl (*Z*)-(3-(3-fluoro-4-methylphenyl)-3-methylbut-1-en-1-yl)phosphonate (*Z*-**11j**), dimethyl (*Z*)-(3-(4-fluoro-3-methylphenyl)-3-methylbut-1-en-1-yl)phosphonate (*Z*-**11k**), dimethyl (*Z*)-(3-(3-fluoro-2-methylphenyl)-3-methylbut-1-en-1-yl)phosphonate (*Z*-**11l**), dimethyl (5-fluoro-3,3,6-trimethyl-2,3-dihydro-1*H*-inden-1-yl)phosphonate (**12e**).

Z-**11j**: **^{1}H NMR** (400 MHz, $CDCl_3$) δ 7.21 – 7.09 (m, 2H), 6.98 – 6.86 (m, 1H), 6.61 (dd, *J* = 54.6, 14.6 Hz, 1H), 5.56 (t, *J* = 14.6, 14.6 Hz, 1H), 3.61 (d, *J* = 11.2 Hz, 6H), 2.26 (d, *J* = 1.6 Hz, 6H), 1.60 (6H). **^{13}C NMR** (101 MHz, $CDCl_3$) δ 162.19, 162.15, 143.60, 129.48, 129.43, 125.14, 125.07, 114.61, 112.73, 52.03, 51.96, 42.27, 42.20, 29.10, 14.69, 14.65.**^{31}P NMR** (162 MHz, $CDCl_3$) δ 18.35.

Minor isomers *Z*-**11k** and *Z*-**11l** cannot be distinguished.

First minor: ^{1}**H NMR** selected signals (400 MHz, $CDCl_3$) δ 6.60 (dd, *J* = 54.4, 14.4 Hz, 1H), 5.58 (dd, *J* = 14.4, 14.4 Hz, 1H), 3.44 (d, *J* = 11.2 Hz, 6H), 2.24-2.22 m (3H), 1.62 (3H). ^{31}**P NMR** (162 MHz, $CDCl_3$) δ 18.19.

Second minor: ^{1}**H NMR** selected signals (400 MHz, $CDCl_3$) δ 5.50 (dd, *J* = 14.1 Hz, 1H), 3.62 (d, *J* = 11.1 Hz, 6H), 2.24-2.22 m (3H), 1.43 (3H). ^{31}**P NMR** (162 MHz, $CDCl_3$) δ 17.86.

12e: ^{1}**H NMR** selected signals (400 MHz, $CDCl_3$) δ 3.71 (dd, *J* = 10.7, 3.8 Hz, 1H), 1.36 (3H), 1.16 (3H). ^{31}**P NMR** (162 MHz, $CDCl_3$) δ 31.47 (d, *J* = 2.4 Hz). **HRMS** (ESI) (for mixture of isomers): m/z calcd for $C_{14}H_{20}FNaO_3P$ $[M+Na]^+$ 309.1032, found 309.1036.

Dimethyl (*E*)-(3-(4-bromophenyl)-3-methylbut-1-en-1-yl)phosphonate (*E*-**11m**). Colorless oil, yield of 40%; ^{1}**H NMR** (400 MHz, $CDCl_3$) δ 7.44 (d, *J* = 8.6 Hz, 2H), 7.16 (d, *J* = 8.6 Hz, 2H), 6.93 (dd, *J* = 22.7, 17.4 Hz, 1H), 5.59 (dd, *J* = 19.6, 17.5 Hz, 1H), 3.73 (d, *J* = 11.1 Hz, 6H), 1.45 (6H). ^{13}**C NMR** (101 MHz, $CDCl_3$) δ 174.7, 161.7 (d, *J* = 4.6 Hz), 145.0, 131.5, 127.9, 120.4, 112.3 (d, *J* = 189.2 Hz), 52.4 (d, *J* = 5.8 Hz), 42.0 (d, *J* = 20.0 Hz), 27.6. ^{31}**P NMR** (162 MHz, $CDCl_3$) δ 22.18. **HRMS** (ESI): m/z calcd for $C_{13}H_{18}BrNaO_3P$ $[M+Na]^+$ 355.0075, found 355.0080.

Dimethyl (*Z*)-(3-(4-bromophenyl)-3-methylbut-1-en-1-yl)phosphonate (*Z*-**11m**). Colorless oil, yield of 40%; ^{1}**H NMR** (400 MHz, $CDCl_3$) δ 7.44 (d, *J* = 8.5 Hz, 2H), 7.27 (d, *J* = 8.5 Hz, 2H), 6.64 (dd, *J* = 54.4, 14.6 Hz, 1H), 5.60 (dd, *J* = 14.3, 14.3 Hz, 1H), 3.61 (d, *J* = 11.2 Hz, 6H), 1.62 (6H). ^{13}**C NMR** (101 MHz, $CDCl_3$) δ 161.6 (d, *J* = 3.6 Hz), 147.2, 131.1, 128.3, 120.0, 114.4 (d, *J* = 188.6 Hz), 52.0 (d, *J* = 6.2 Hz), 42.4 (d, *J* = 7.0 Hz), 29.1.^{31}**P NMR** (162 MHz, $CDCl_3$) δ 18.01. **HRMS** (ESI): m/z calcd for $C_{13}H_{18}BrNaO_3P$ $[M+Na]^+$ 355.0075, found 355.0082.

Dimethyl (*E*)-(1-bromo-3-methyl-3-phenylbut-1-en-1-yl)phosphonate (*Z*-**11n**). Yellow oil, yield of 95%; ^{1}**H NMR** (400 MHz, $CDCl_3$) δ 7.50 (d, *J* = 40.1 Hz, 1H), 7.40 – 7.12 (m, 5H), 3.54 (d, *J* = 11.4 Hz, 6H), 1.67 (6H). ^{13}**C NMR** (101 MHz, $CDCl_3$) δ 163.7 (d, *J* = 14.8 Hz), 149.0, 128.8 (d, *J* = 114.4 Hz), 128.0, 126.3, 126.2, 126.1 (d, *J* = 38.0 Hz), 125.9, 53.0 (d, *J* = 5.8 Hz), 43.9 (d, *J* = 3.4 Hz), 30.8. ^{31}**P NMR** (162 MHz, $CDCl_3$) δ 8.60. **HRMS** (ESI): m/z calcd for $C_{13}H_{18}BrNaO_3P$ $[M+Na]^+$ 355.0075, found 355.0071.

(*Z*)-(3-Methyl-3-phenylbut-1-en-1-yl)dimorpholinophosphine oxide (*Z*-**11o**). Yellow oil, yield of 76%; ^{1}**H NMR** (400 MHz, $CDCl_3$) δ 7.45 – 7.12 (m, 5H), 6.85 (dd, *J* = 46.9, 14.7 Hz, 1H), 5.41 (dd, *J* = 14.7, 14.7 Hz, 1H), 3.60 (t, *J* = 4.5 Hz, 8H), 3.03 – 2.99 (m, 8H), 1.72 (6H). ^{13}**C NMR** (101 MHz, $CDCl_3$) δ 163.2 (d, *J* = 2.0 Hz), 149.8 (d, *J* = 1.1 Hz), 128.9, 128.4, 128.0, 126.3, 125.7, 116.3 (d, *J* = 150.0 Hz), 67.4 (d, *J* = 5.8 Hz), 44.0, 29.9 (d, *J* = 0.8 Hz). ^{31}**P NMR** (162 MHz, $CDCl_3$) δ 15.87. **HRMS** (ESI): m/z calcd for $C_{19}H_{29}N_2NaO_3P$ $[M+Na]^+$ 387.1813, found 387.1818.

Trichloro((dichloro(3-methyl-2λ^5-buta-1,2-dien-1-yl)phosphonio)oxy)aluminate (**13**). ^{1}**H NMR** (400 MHz, CD_2Cl_2) δ 6.08 (broadened d, *J* = 28.2 Hz, 1H), 1.92 (br. d, *J* = 12.8 Hz, 6H). ^{13}**C NMR** (101 MHz, CD_2Cl_2) δ 212.7 (br. d, *J* = 8.7 Hz), 107.9 (br. d, *J* = 5.2 Hz), 86.9 (br. d, *J* = 164.6 Hz), 19.1 (br. d, *J* = 9.9 Hz). ^{31}**P NMR** (162 MHz, CD_2Cl_2) δ 47.34 (br. s).

(E)-3-Methylbuta-1,3-diene-1-phosphonyl dichloride (**14**). **^{1}H NMR** (400 MHz, CD_2Cl_2-$AlCl_3$) δ 7.44 (dd, J = 34.5, 17.0 Hz, 1H), 6.41 (dd, J = 44.5, 17.0 Hz, 1H), 4.42 (br. s, 1H), 4.32 (br. s, 1H), 2.46 (3H). **^{13}C NMR** (101 MHz, CD_2Cl_2-$AlCl_3$) δ 162.4 (d, J = 6.7 Hz), 137.0 (d, J = 30.5 Hz), 135.9, 118.2 (d, J = 150.4 Hz), 29.0. **^{31}P NMR** (162 MHz, CD_2Cl_2-$AlCl_3$) δ 67.80.

(Z)-3-Methyl-3-(phenyl-d_5)-2-d-butene-1-phosphonyl dichloride (**15**). **^{1}H NMR** (400 MHz, CD_2Cl_2-$AlCl_3$) δ 6.26 (d, J = 37.4 Hz, 1H), 1.69 (d, J = 4.0 Hz, 6H). **^{13}C NMR** (101 MHz, CD_2Cl_2-$AlCl_3$) δ 173.9 (dt, J = 24.4, 23.3 Hz), 144.4, 129.99 – 128.69 (m), 128.74 – 127.78 (m), 127.52 – 126.70 (m), 116.6 (d, J = 146.6 Hz), 46.96 – 43.28 (m), 29.81. **^{31}P NMR** (162 MHz, CD_2Cl_2-$AlCl_3$) δ 52.58 (t, J = 12.9 Hz).

I. Copies of NMR spectra

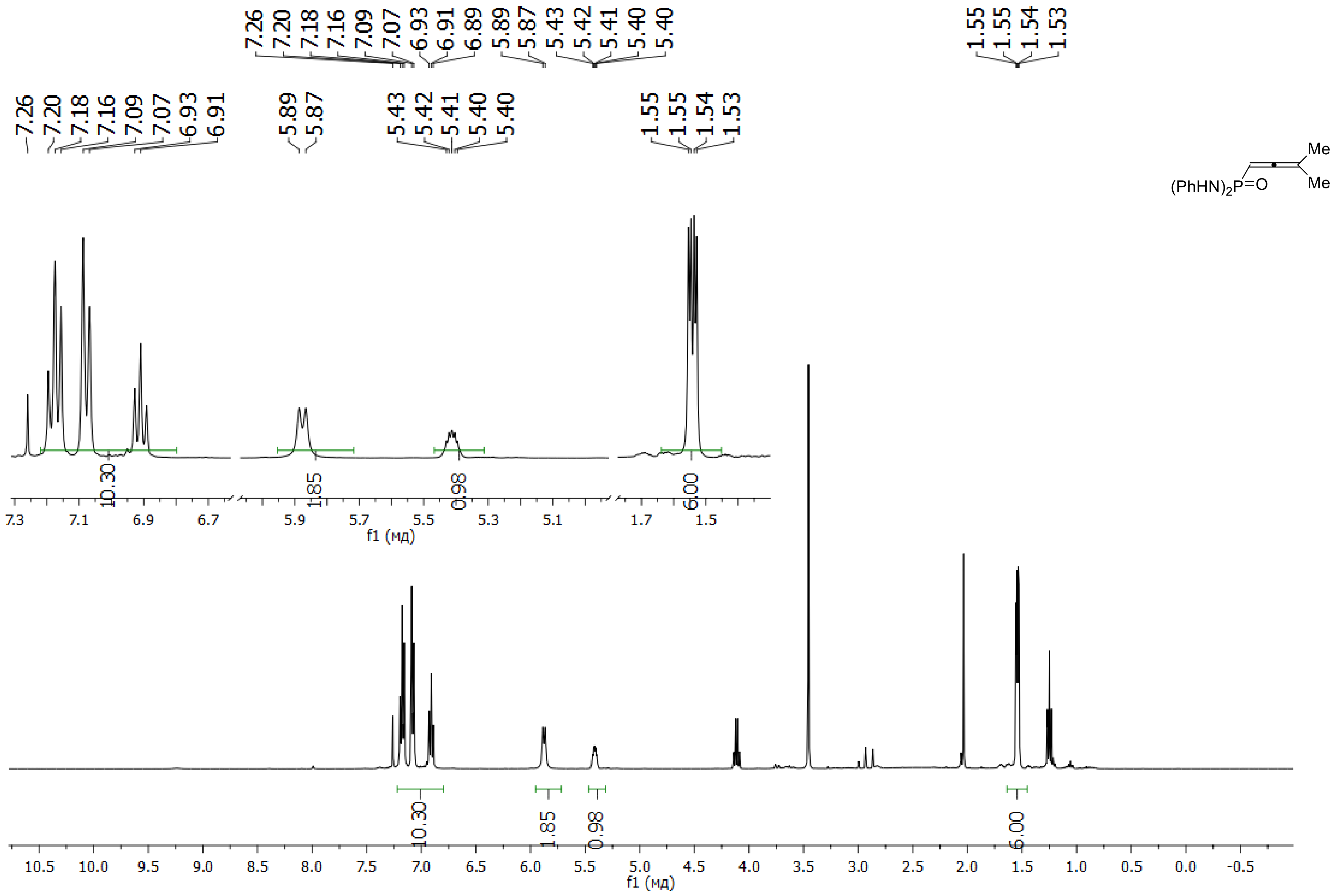

Figure S1. ^{1}H NMR spectrum of the compound **1g** (400 MHz, $CDCl_3$).

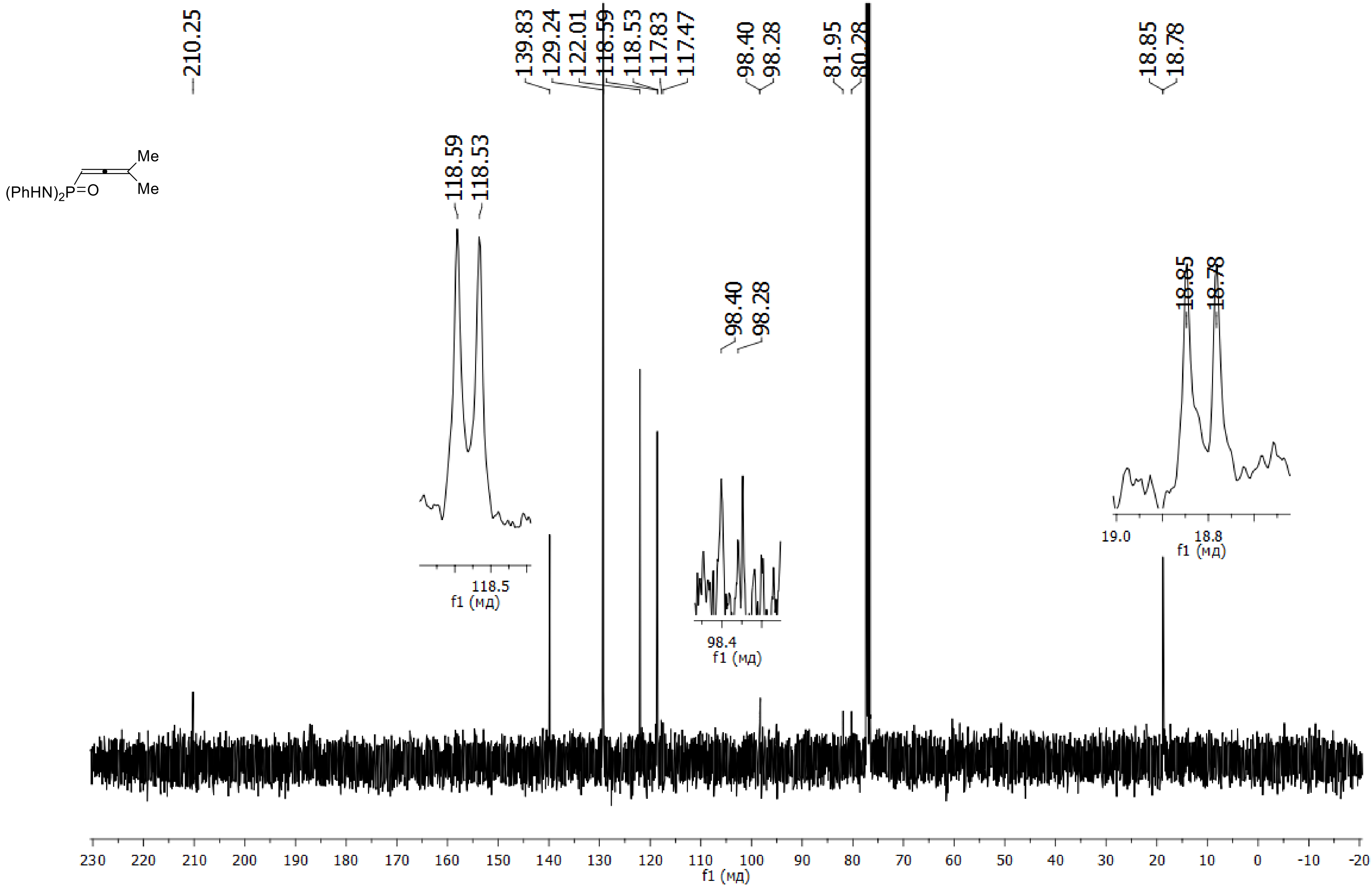

Figure S2. ^{13}C NMR spectrum of the compound **1g** (100 MHz, $CDCl_3$).

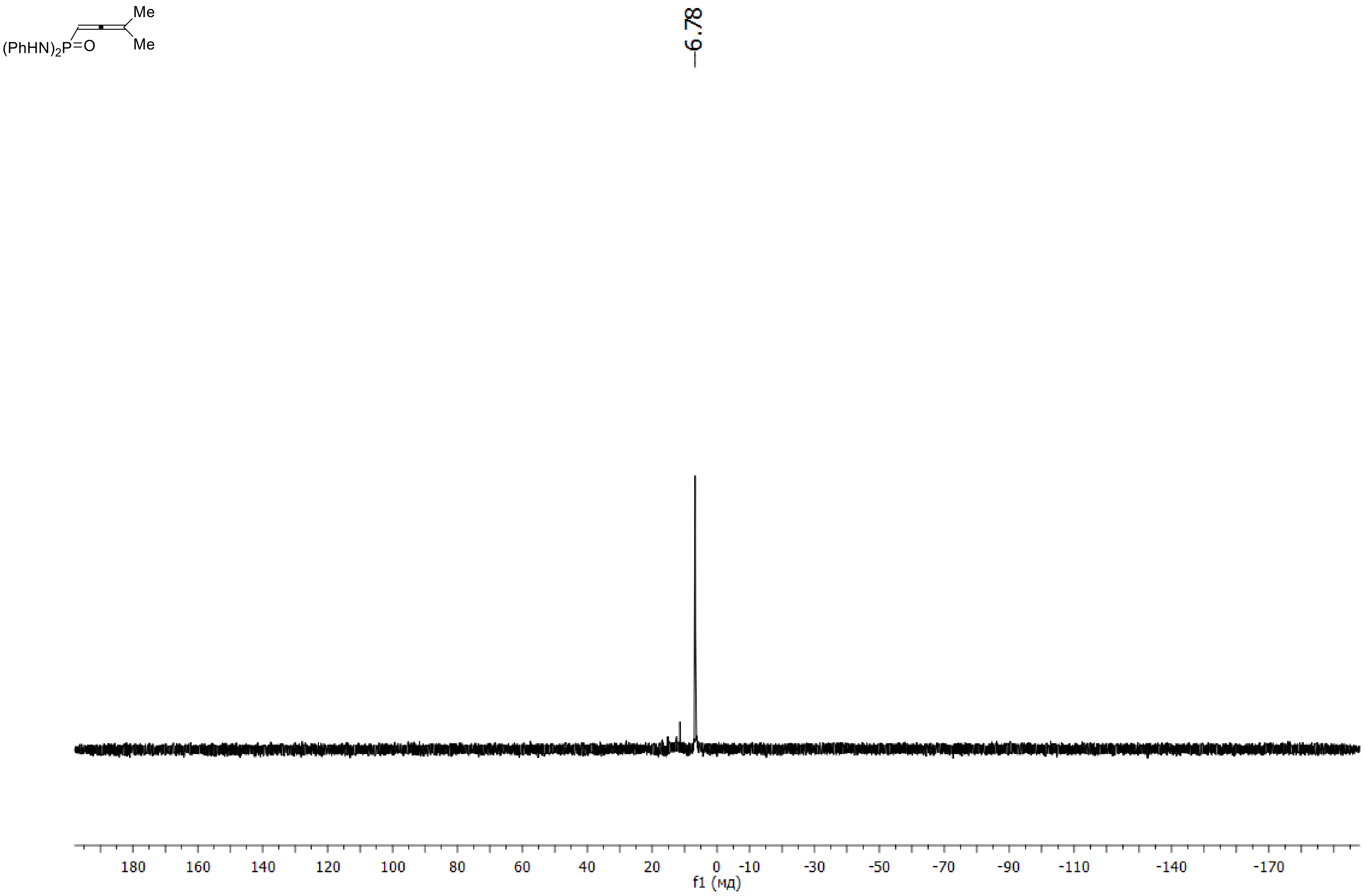

Figure S3. ^{31}P NMR spectrum of the compound **1g** (162 MHz, $CDCl_3$).

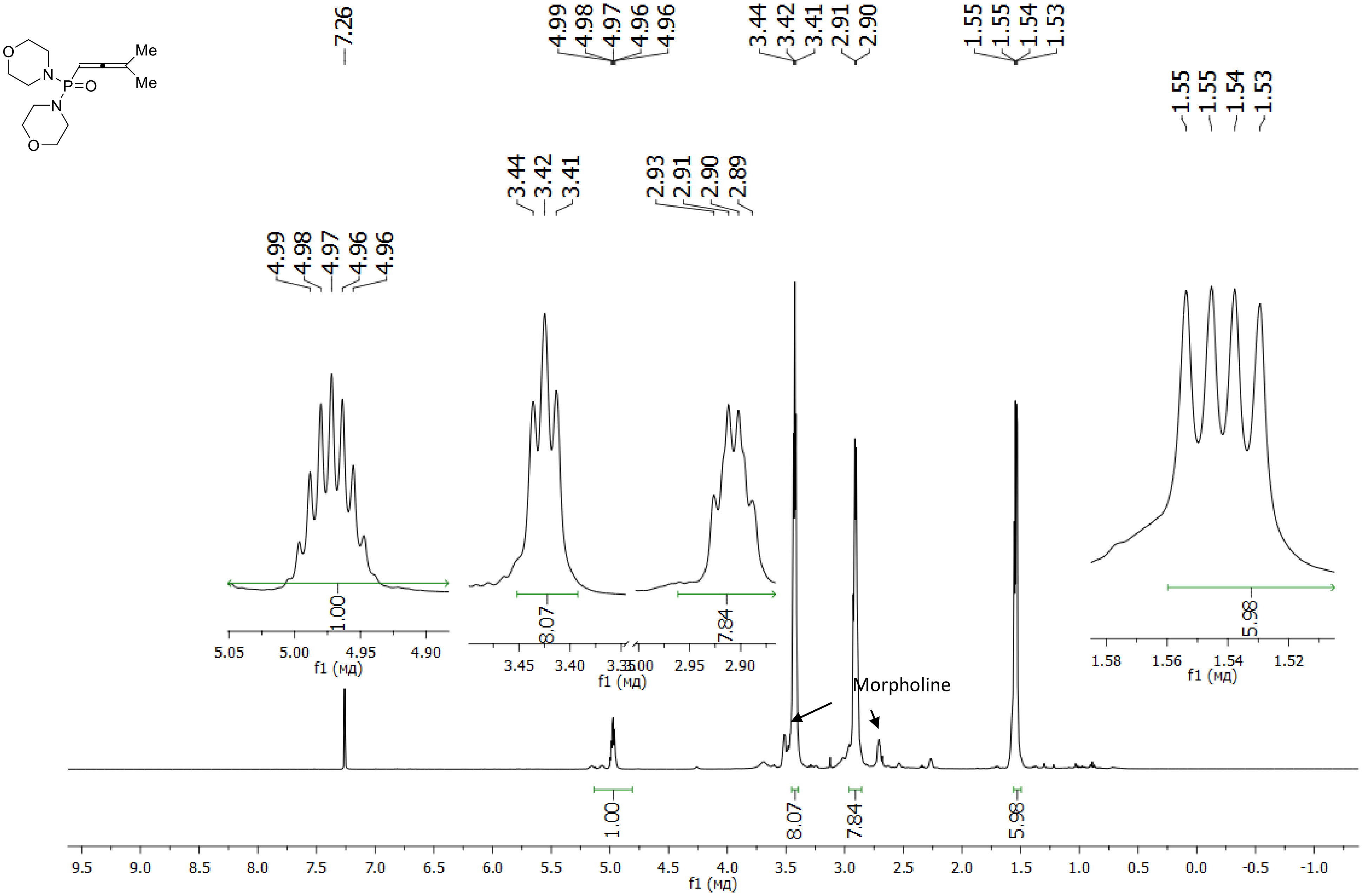

Figure S4. ^{1}H NMR spectrum of the compound **1e** (400 MHz, $CDCl_3$).

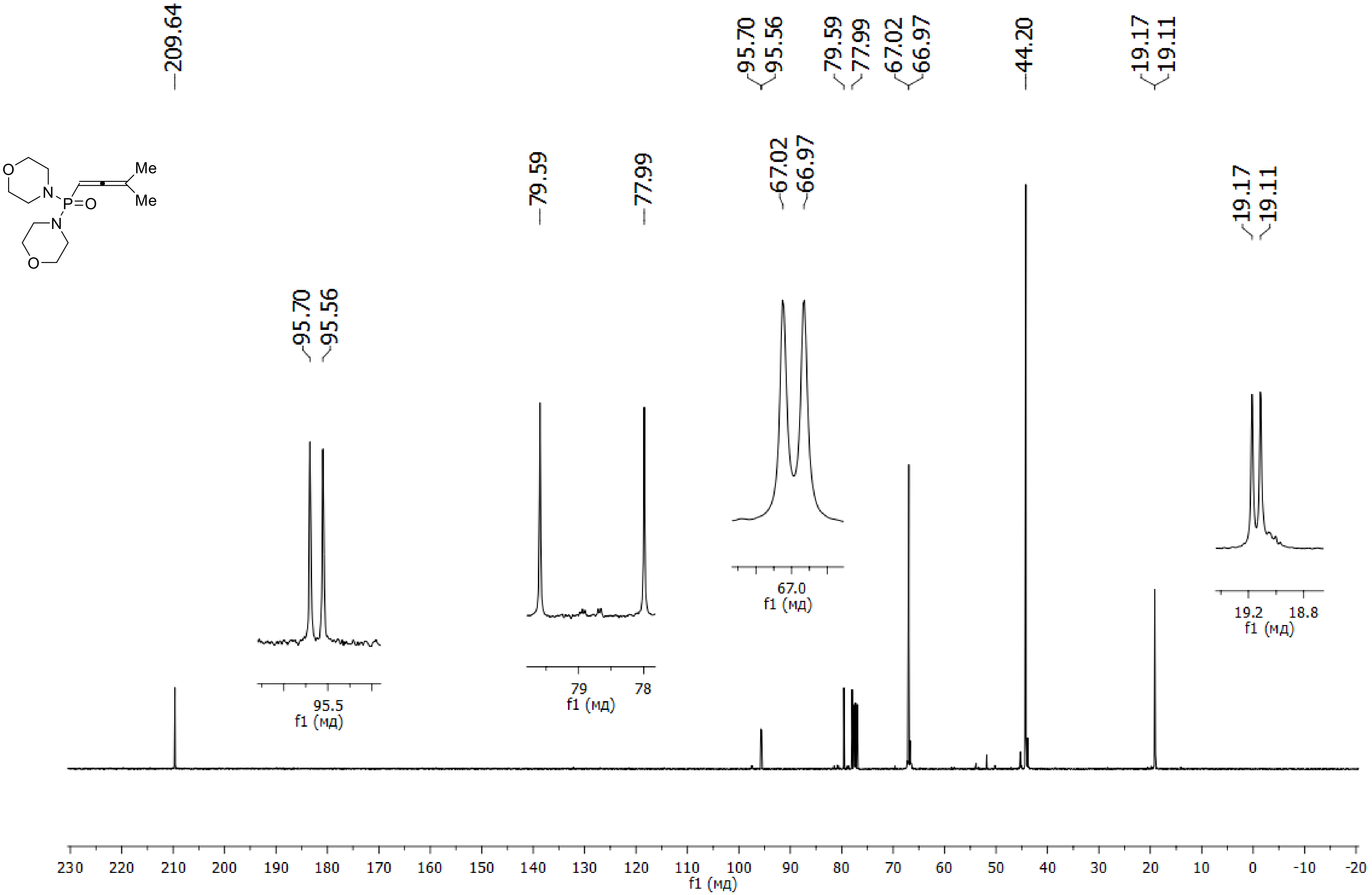

Figure S5. ^{13}C NMR spectrum of the compound **1e** (100 MHz, $CDCl_3$).

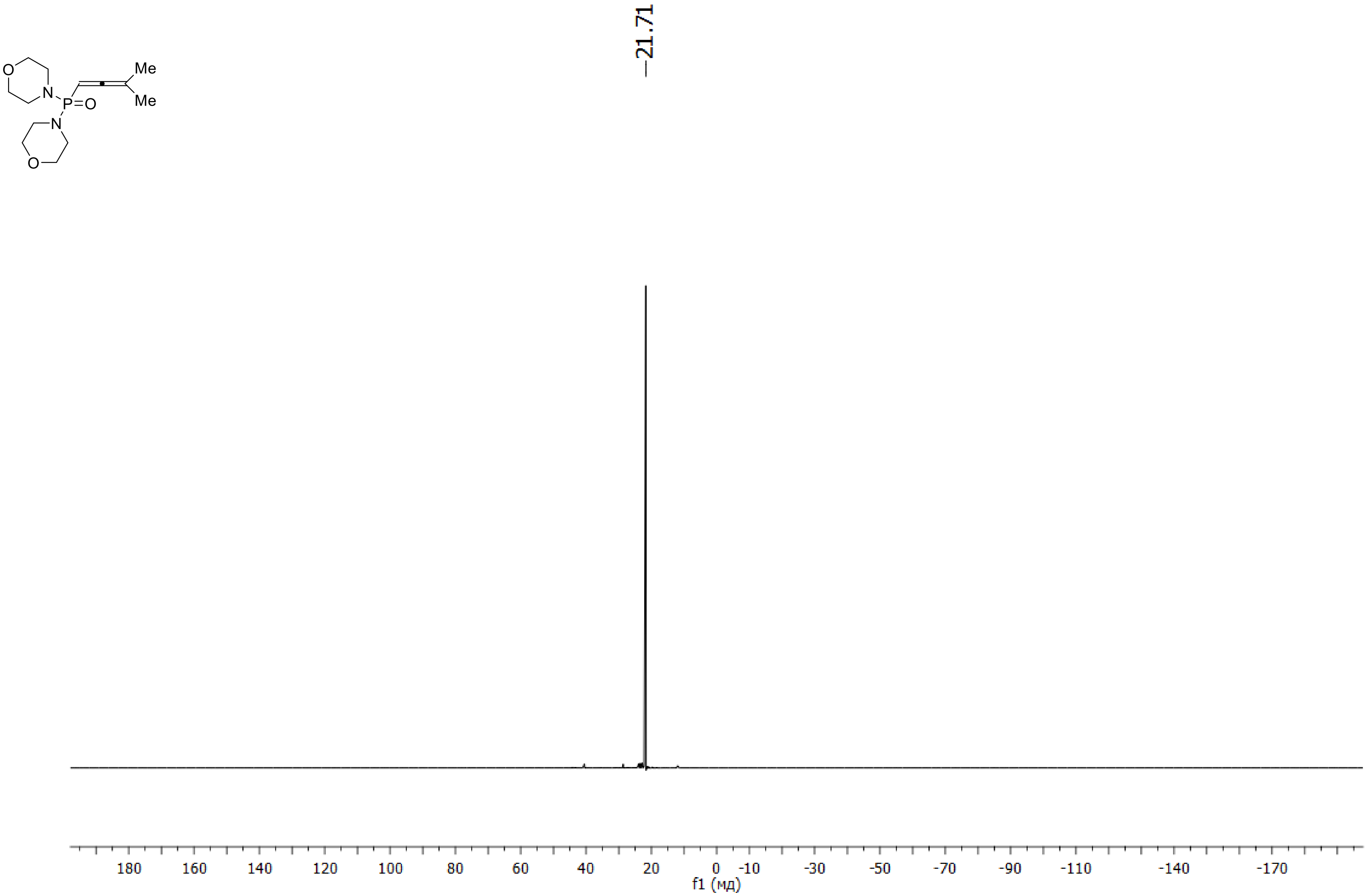

Figure S6. ^{31}P NMR spectrum of the compound **1e** (162 MHz, $CDCl_3$).

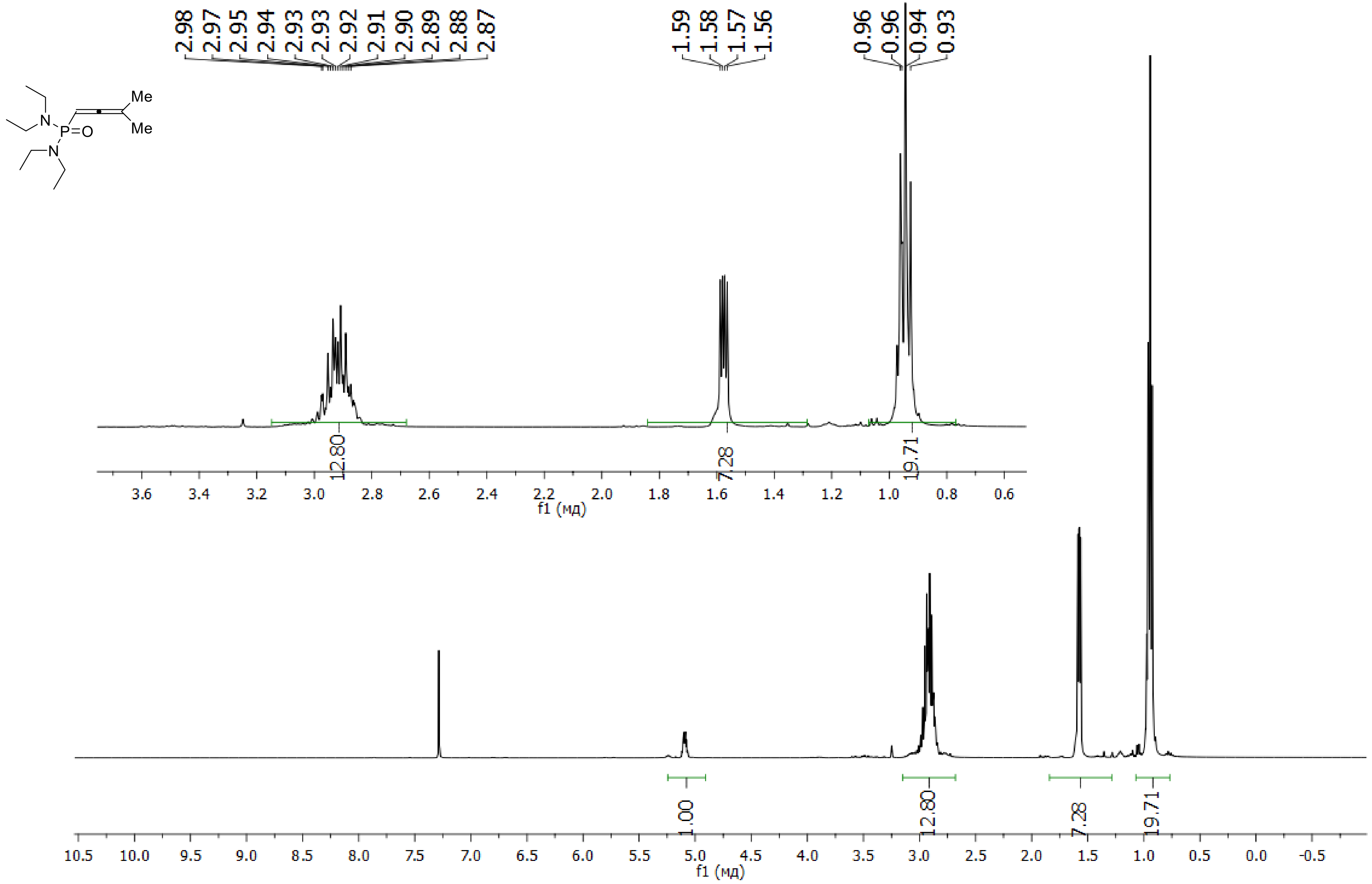

Figure S7. ^{1}H NMR spectrum of the compound **1f** (400 MHz, $CDCl_3$).

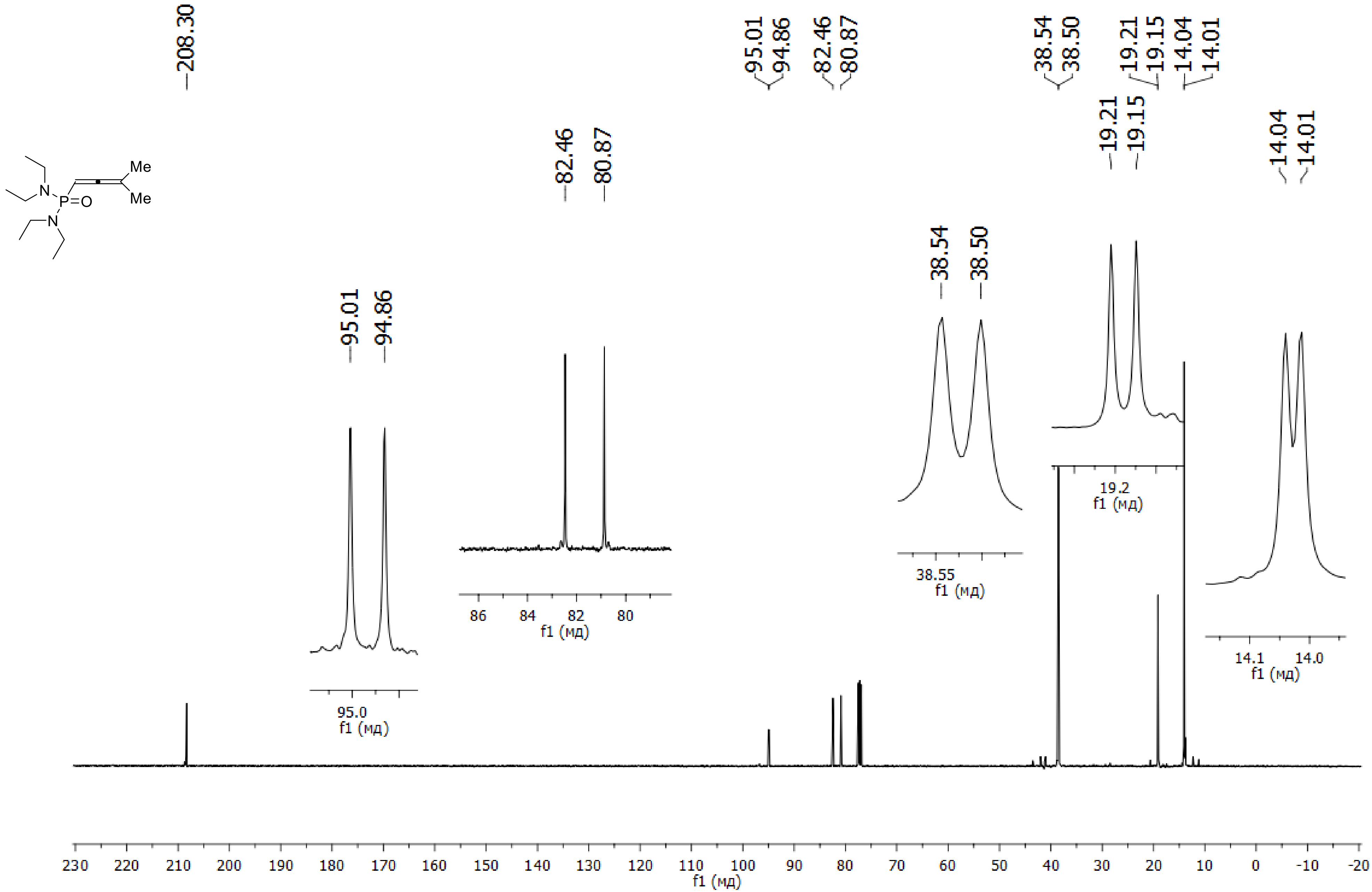

Figure S8. ^{13}C NMR spectrum of the compound **1f** (100 MHz, $CDCl_3$).

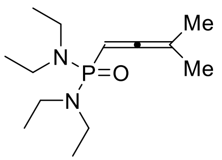

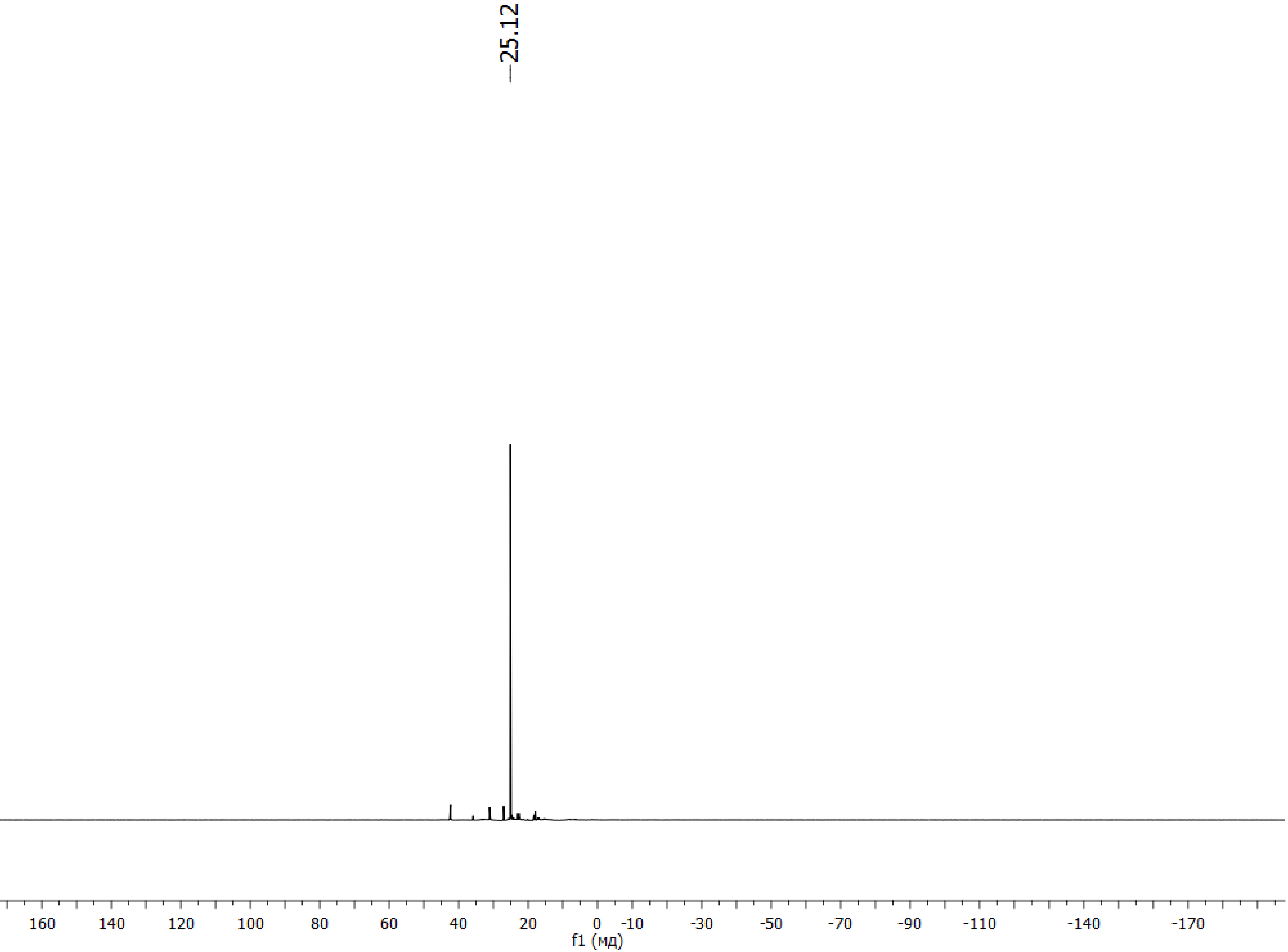

Figure S9. ^{31}P NMR spectrum of the compound **1f** (162 MHz, $CDCl_3$).

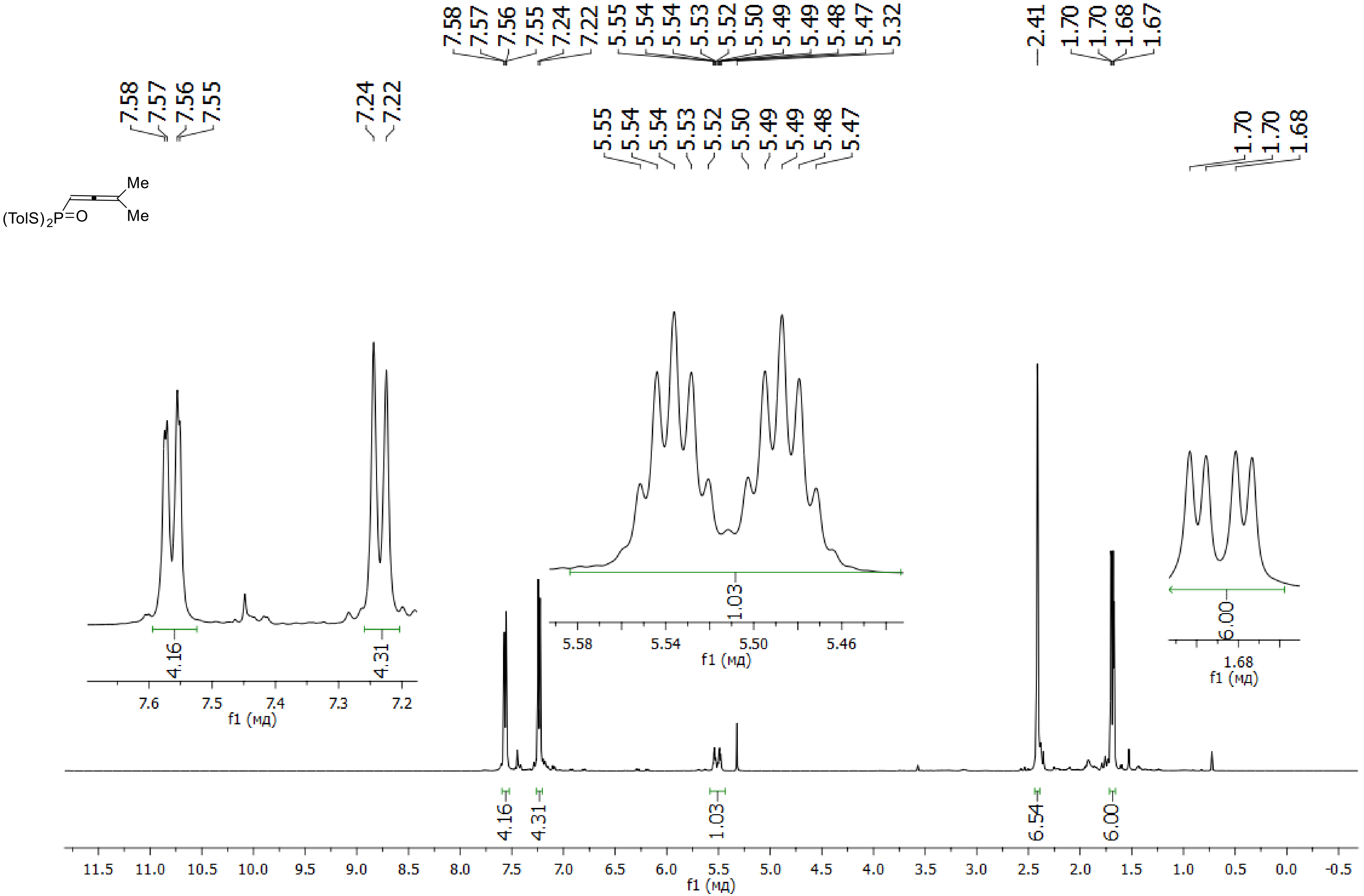

Figure S10. ^{1}H NMR spectrum of the compound **1h** (400 MHz, $CDCl_3$).

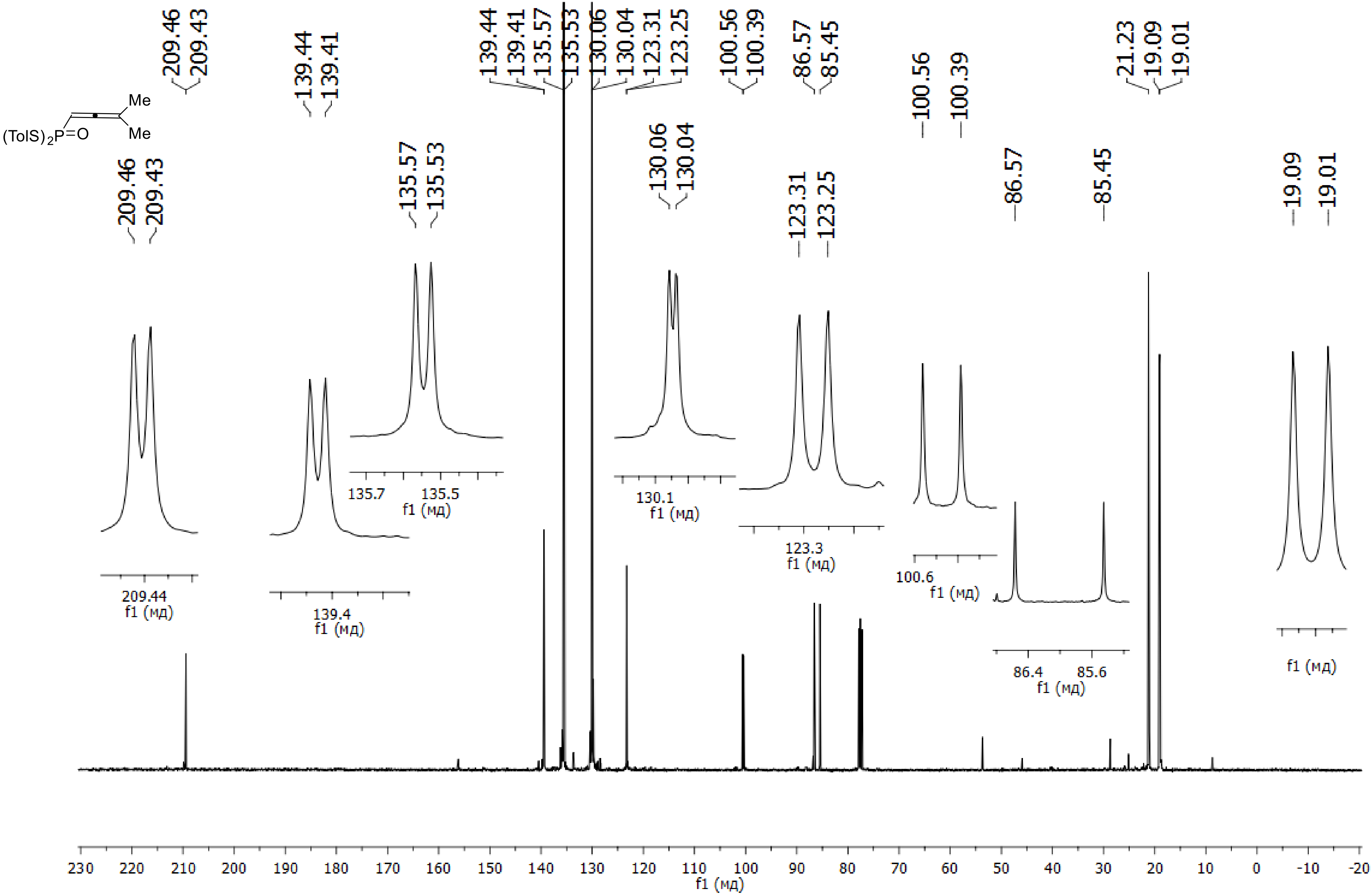

Figure S11. ^{13}C NMR spectrum of the compound **1h** (100 MHz, $CDCl_3$).

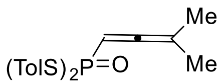

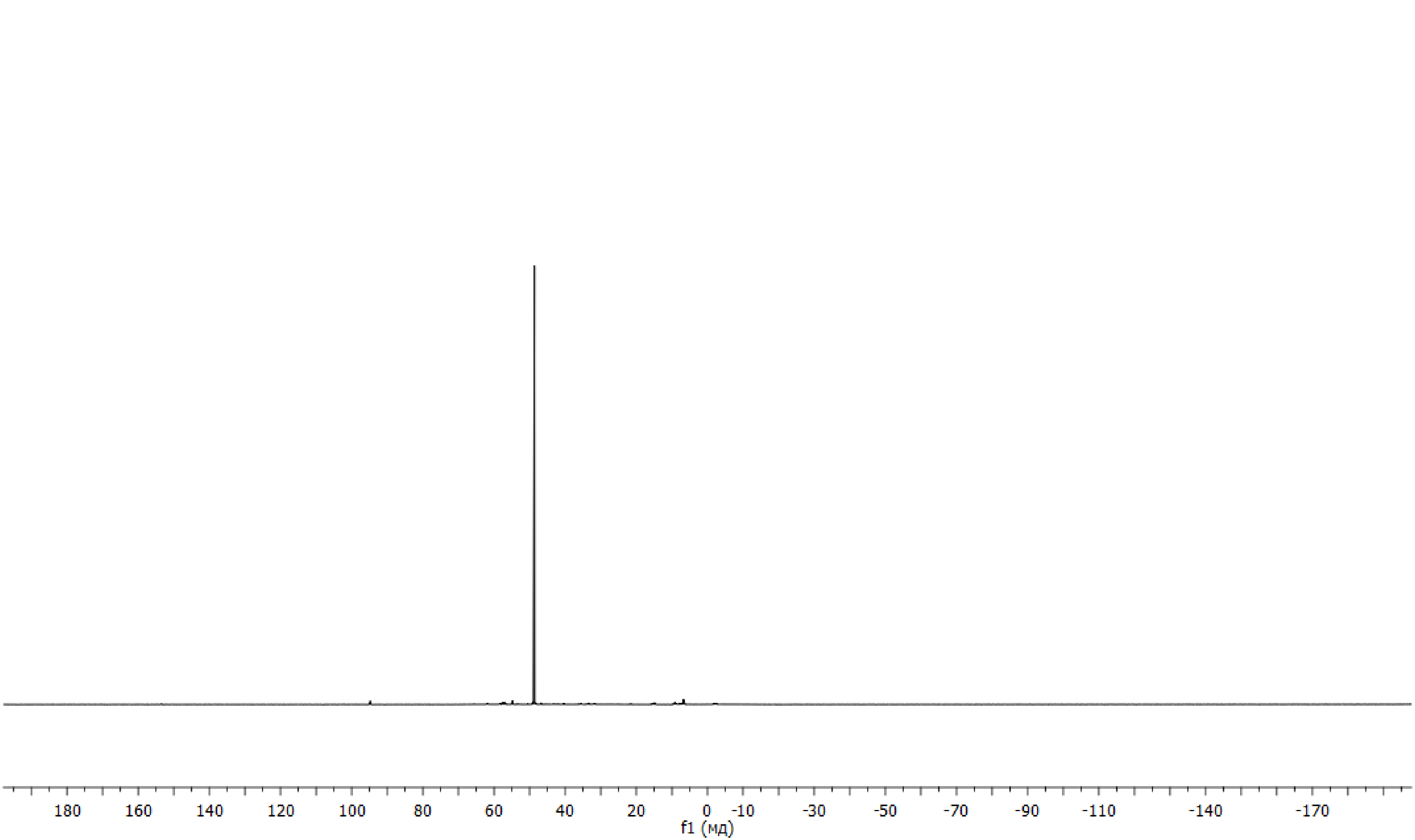

Figure S12. ^{31}P NMR spectrum of the compound **1h** (162 MHz, $CDCl_3$).

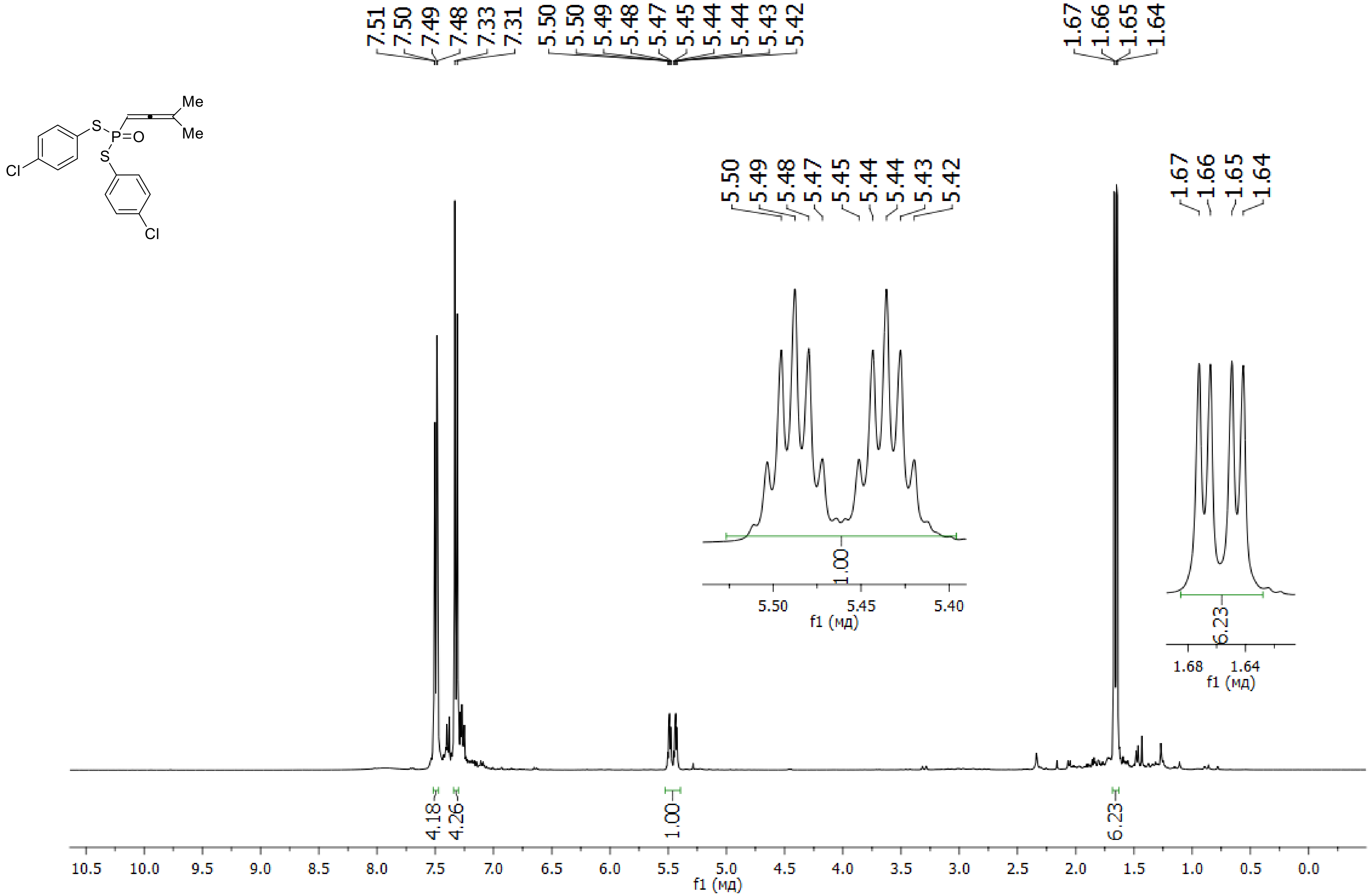

Figure S13. ^{1}H NMR spectrum of the compound **1i** (400 MHz, $CDCl_3$).

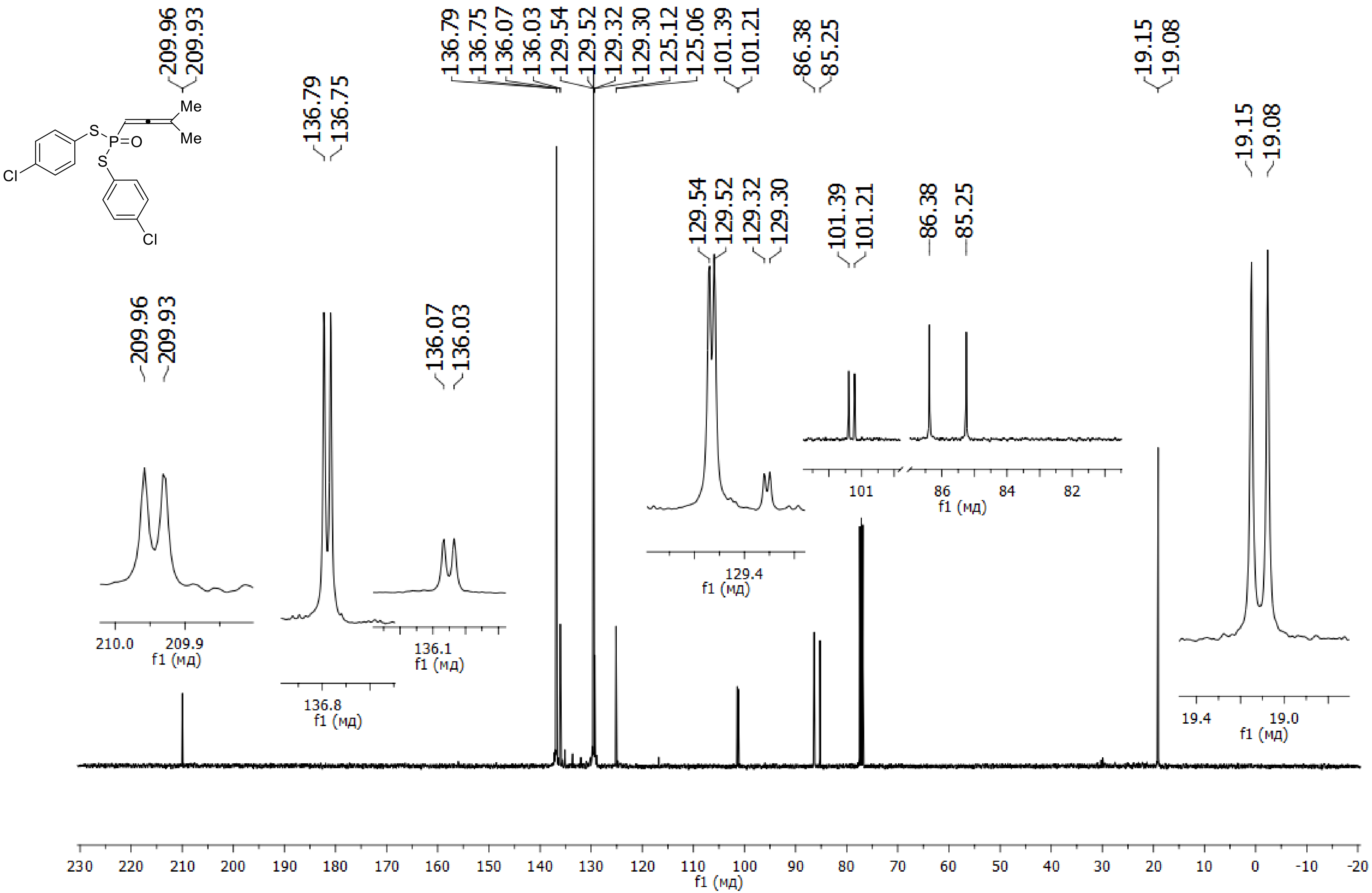

Figure S14. ^{13}C NMR spectrum of the compound **1i** (100 MHz, $CDCl_3$).

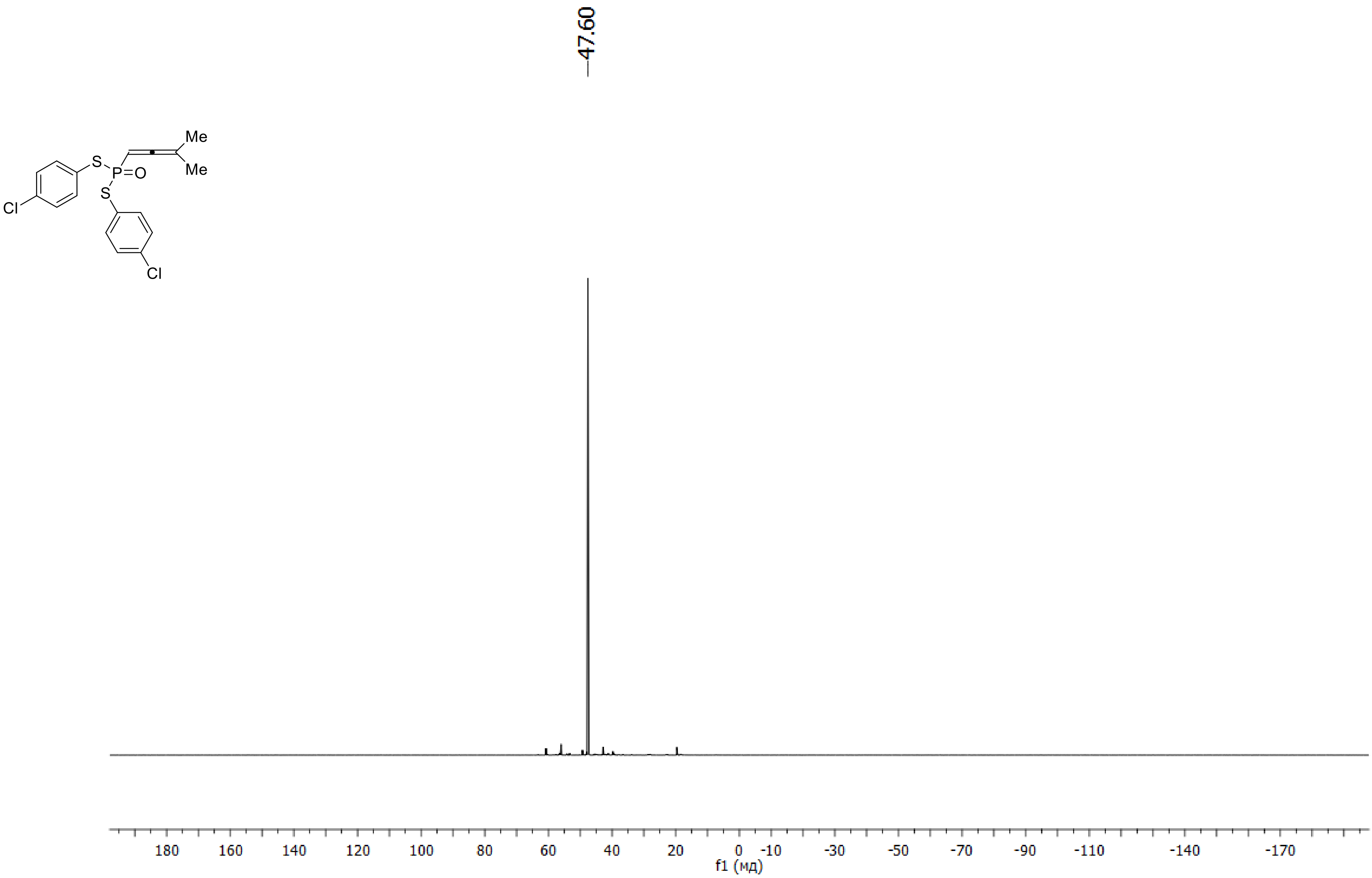

Figure S15. ^{31}P NMR spectrum of the compound **1i** (162 MHz, $CDCl_3$).

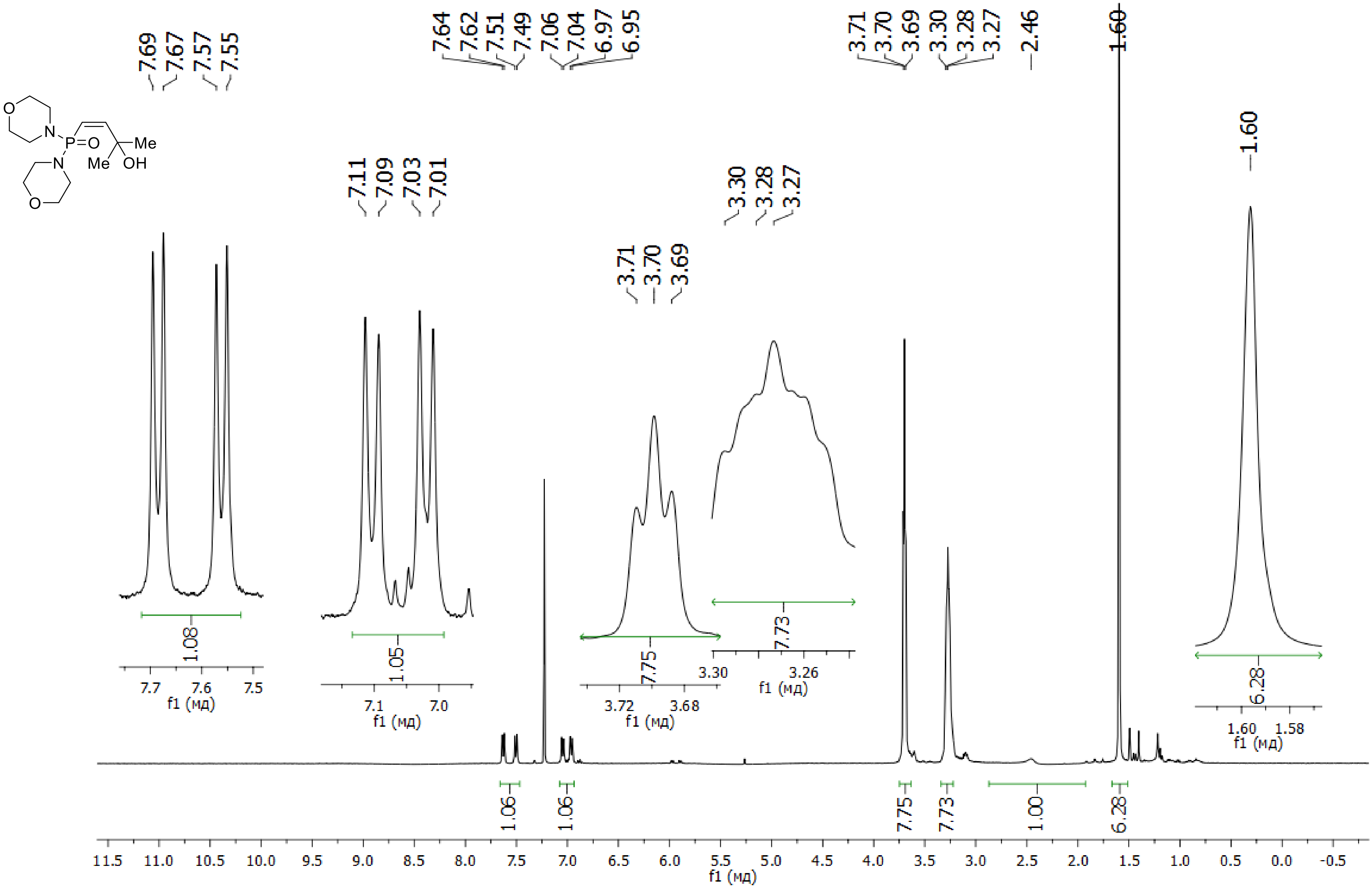

Figure S16. ^{1}H NMR spectrum of the compound **4a** (400 MHz, $CDCl_3$).

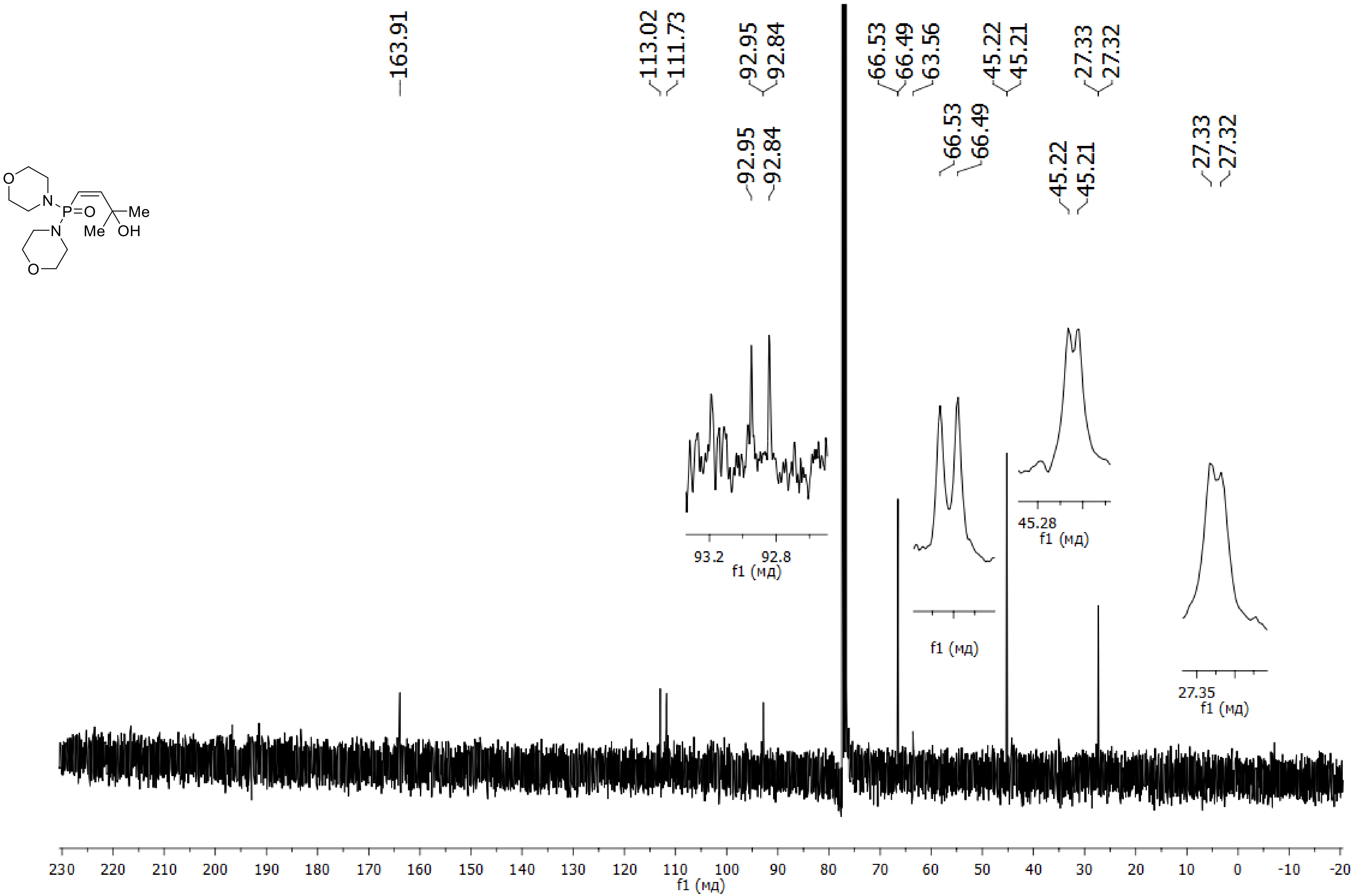

Figure S17. ^{13}C NMR spectrum of the compound **4a** (100 MHz, $CDCl_3$).

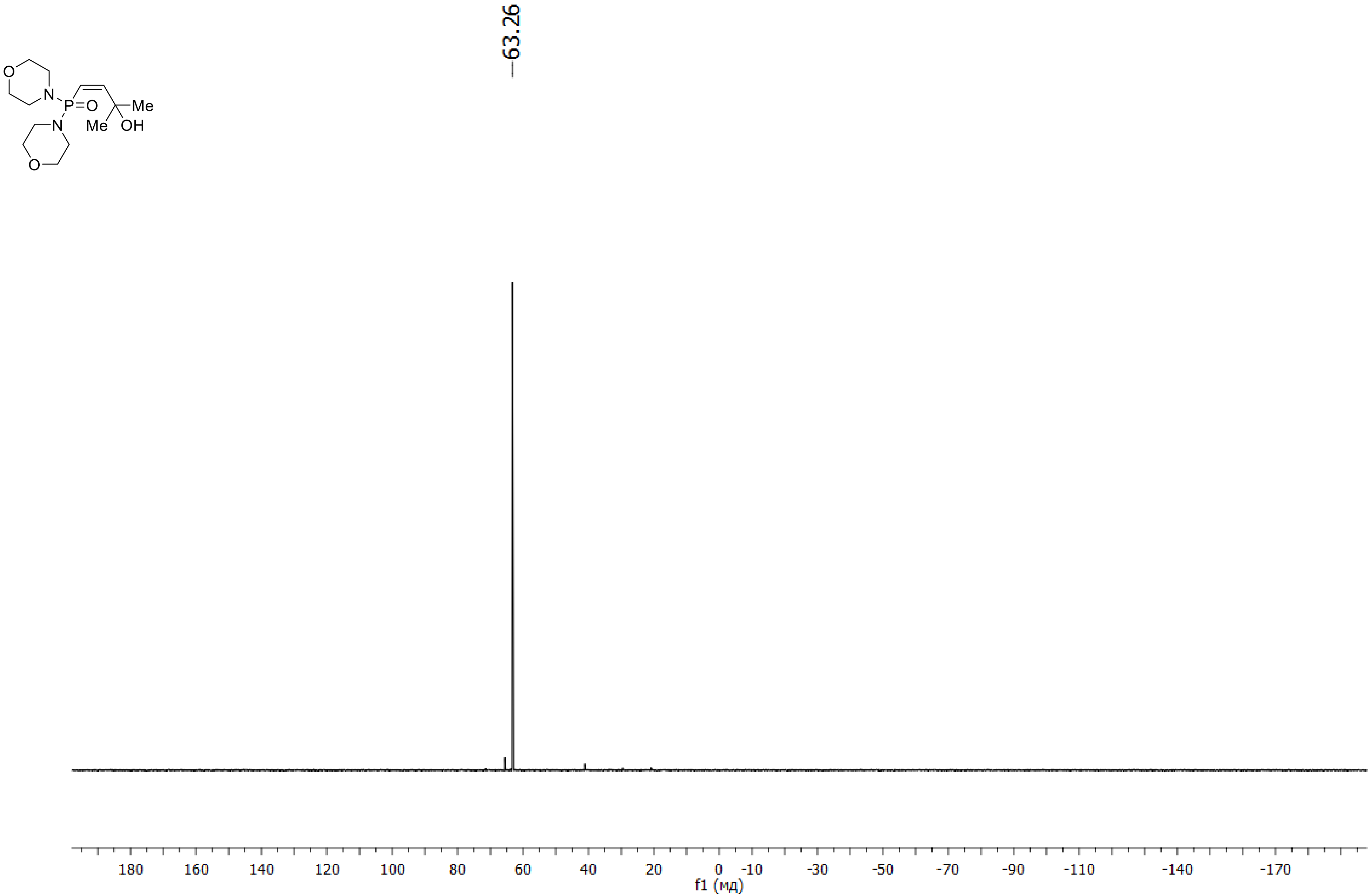

Figure S18. ^{31}P NMR spectrum of the compound **4a** (162 MHz, $CDCl_3$).

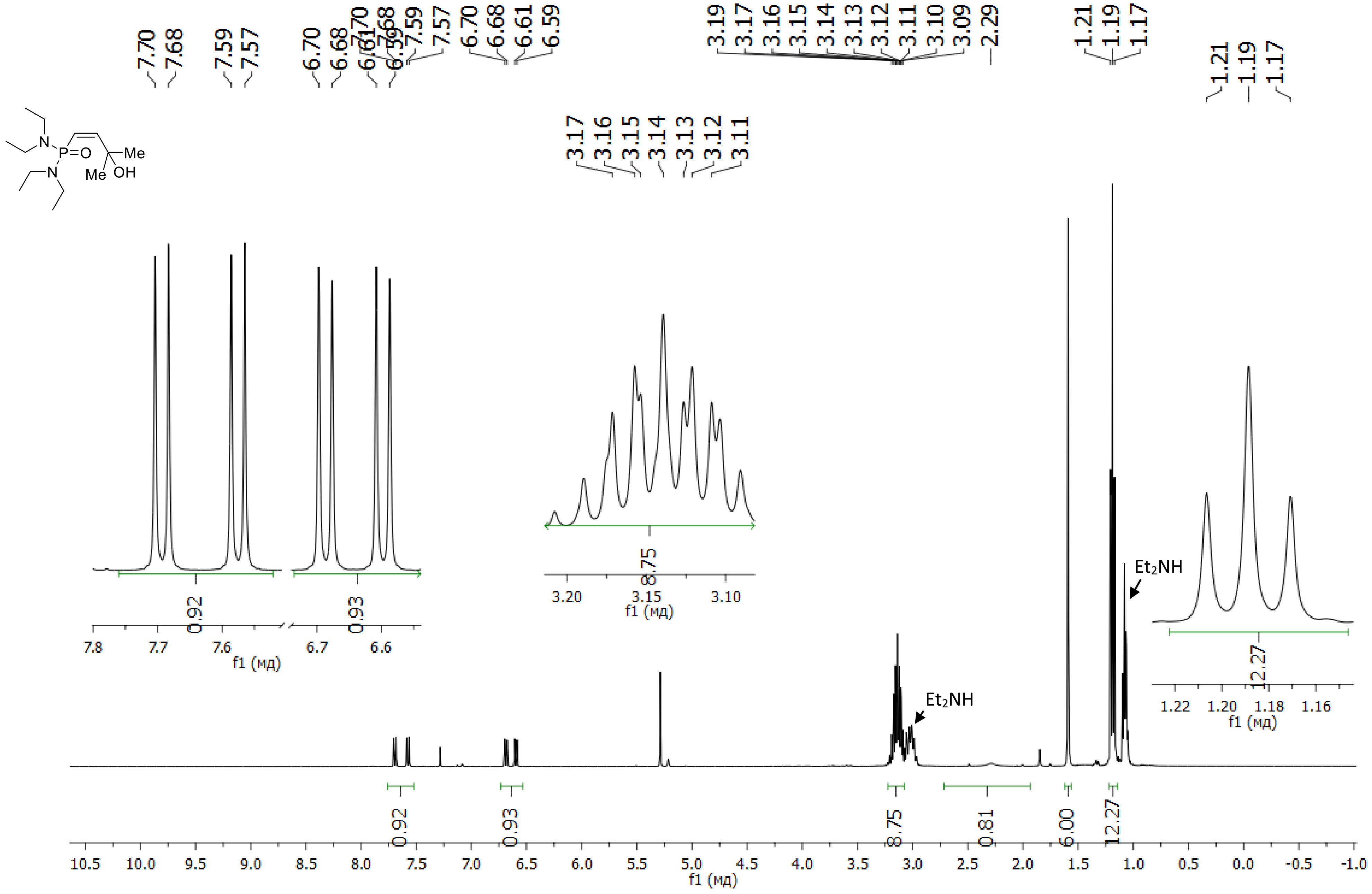

Figure S19. ^{1}H NMR spectrum of the compound **4b** (400 MHz, $CDCl_3$).

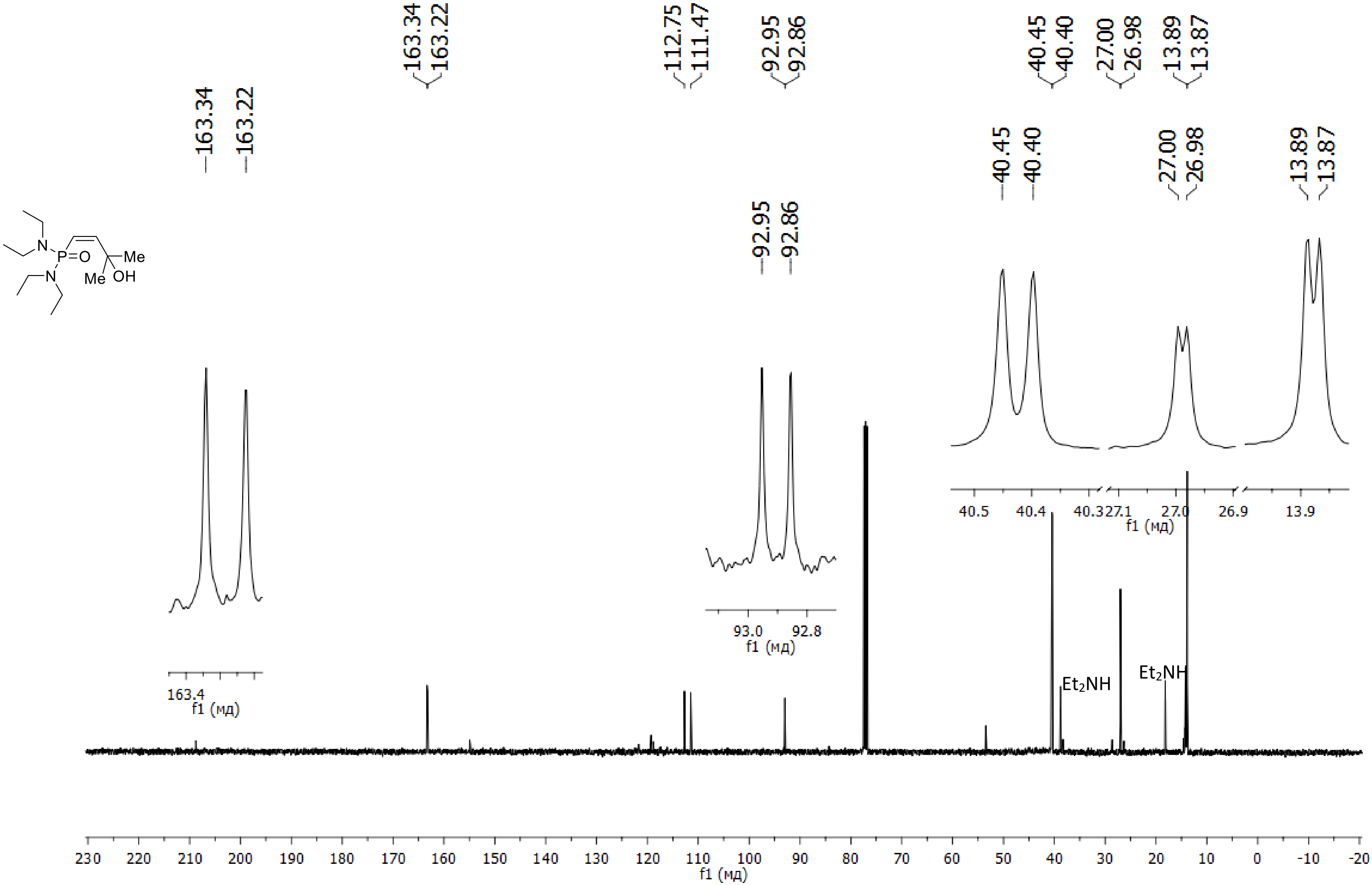

Figure S20. ^{13}C NMR spectrum of the compound **4b** (100 MHz, $CDCl_3$).

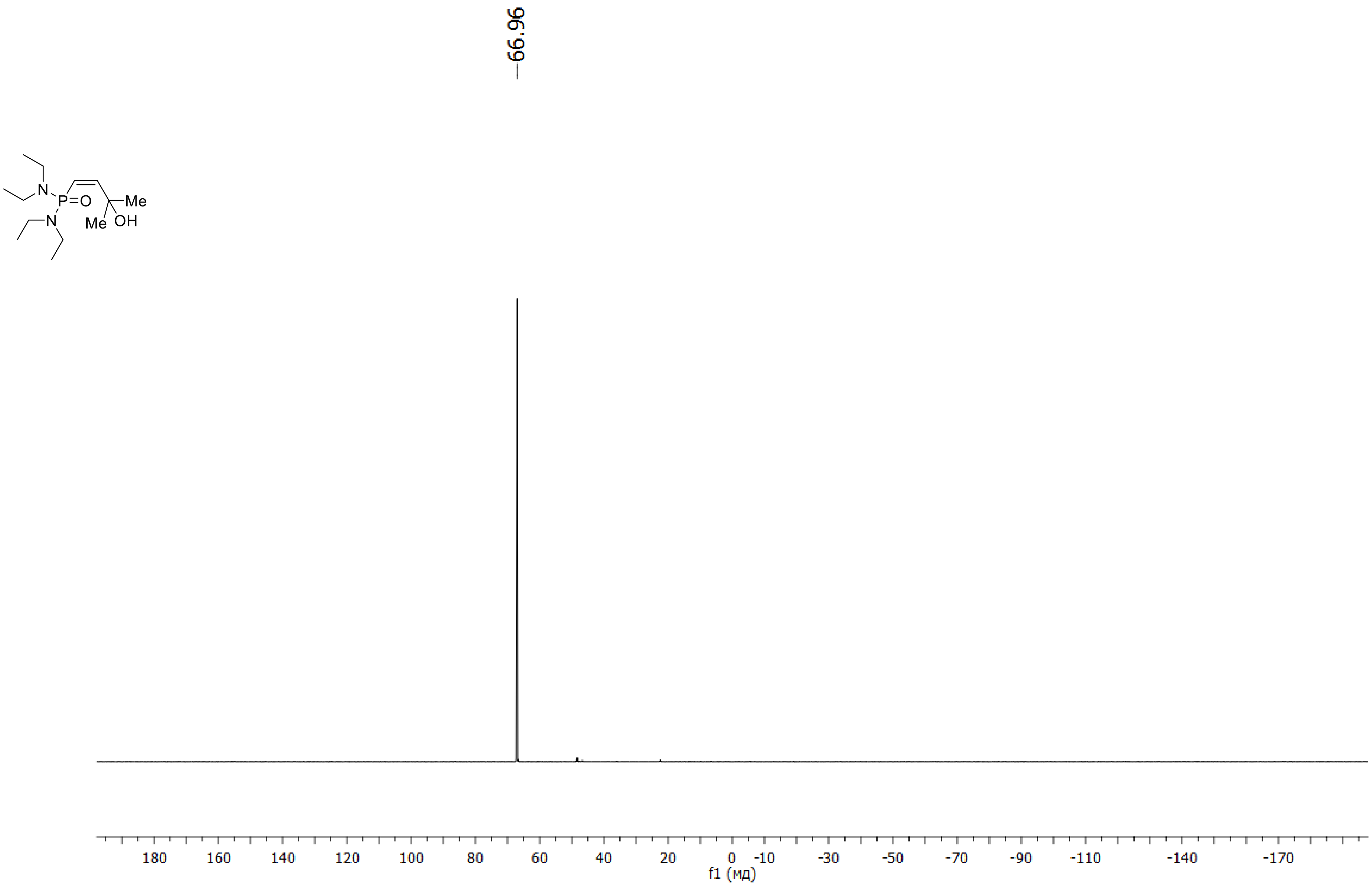

Figure S21. ^{31}P NMR spectrum of the compound **4b** (162 MHz, $CDCl_3$).

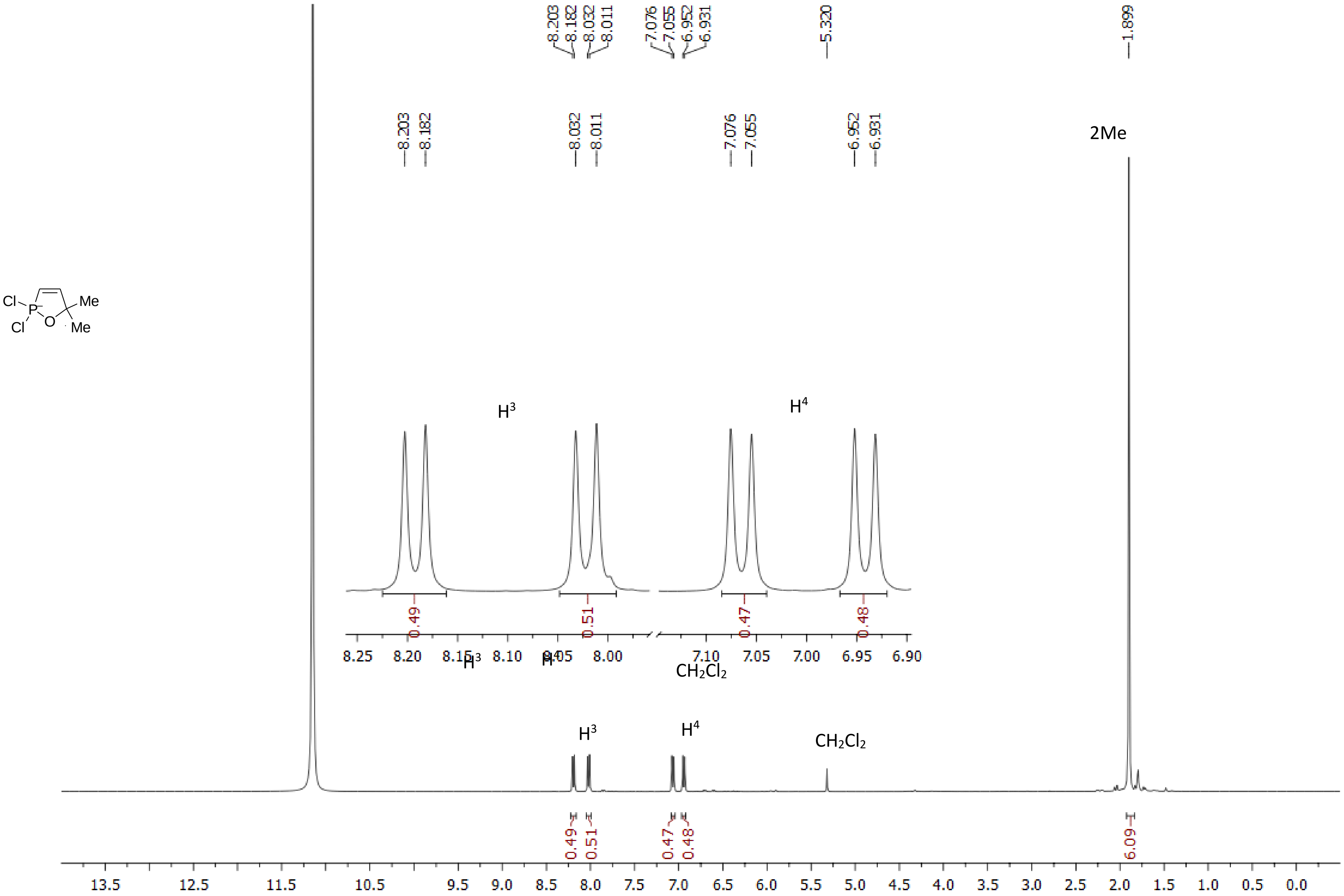

8.203
8.182
8.032
8.011
7.076
7.055
6.952
6.931
5.320
1.899
2Me
H3
H4
CH2Cl2
0.49
0.51
0.47
0.48
6.09
Cl
Cl
P
O
Me
Me
8.25 8.20 8.15 8.10 8.05 8.00
7.10 7.05 7.00 6.95 6.90
13.5 12.5 11.5 10.5 9.5 9.0 8.5 8.0 7.5 7.0 6.5 6.0 5.5 5.0 4.5 4.0 3.5 3.0 2.5 2.0 1.5 1.0 0.5 0.0

Figure S22. ^{1}H NMR spectrum of the compound **A** (400 MHz, TfOH).

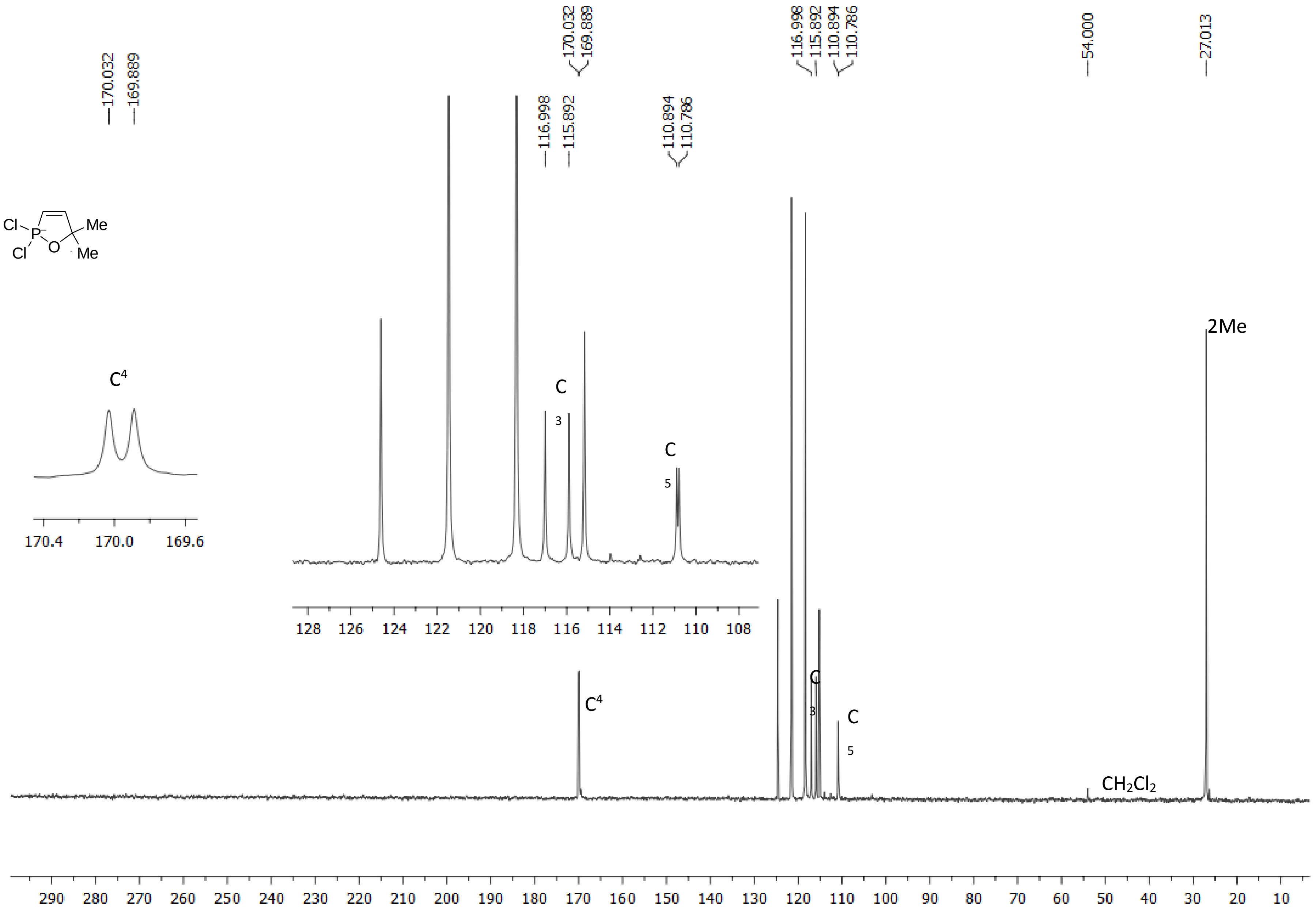

Figure S23 ^{13}C NMR spectrum of the compound **A** (101 MHz, TfOH).

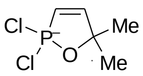

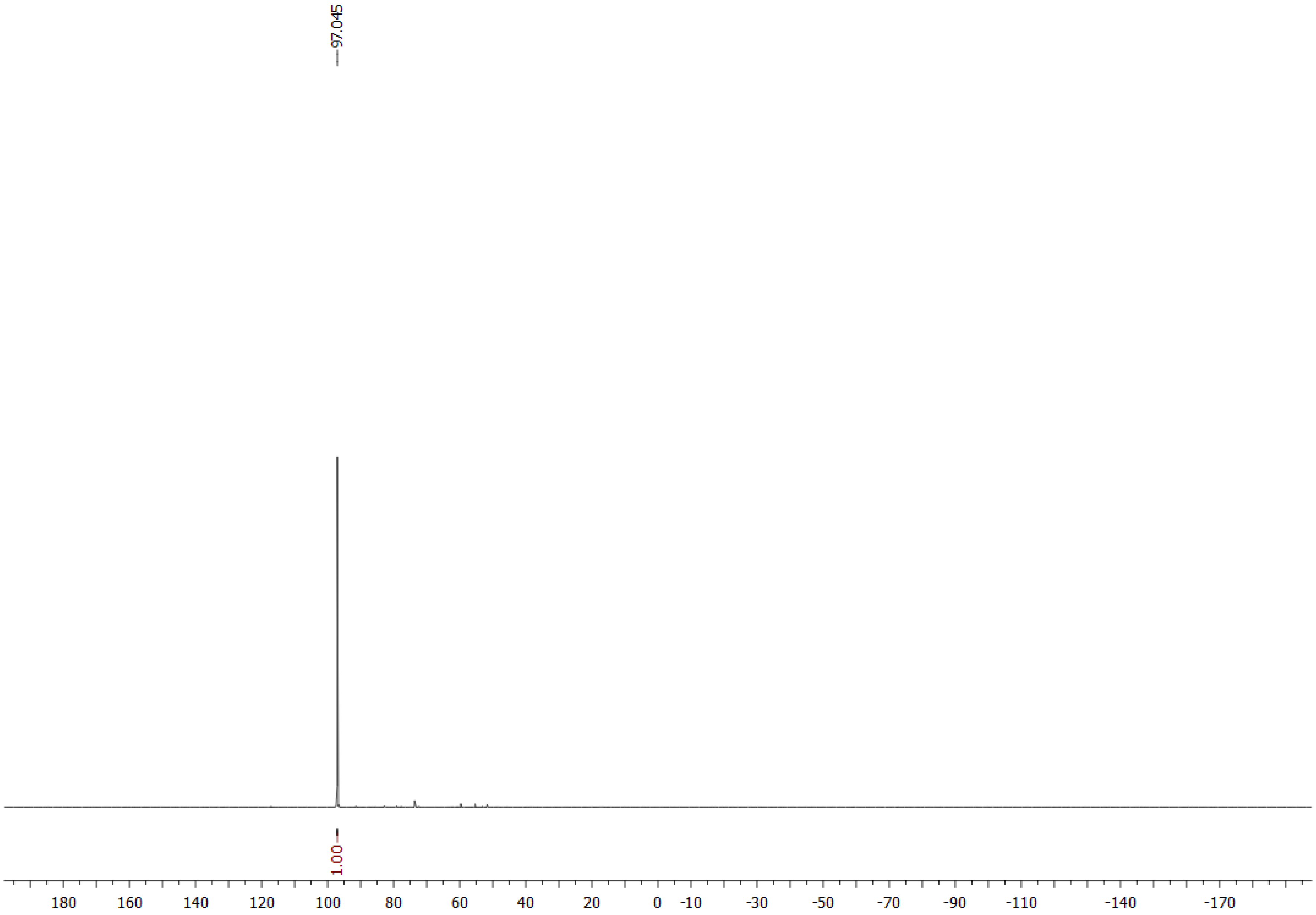

Figure S24 ^{31}P NMR spectrum of the compound **A** (162 MHz, TfOH).

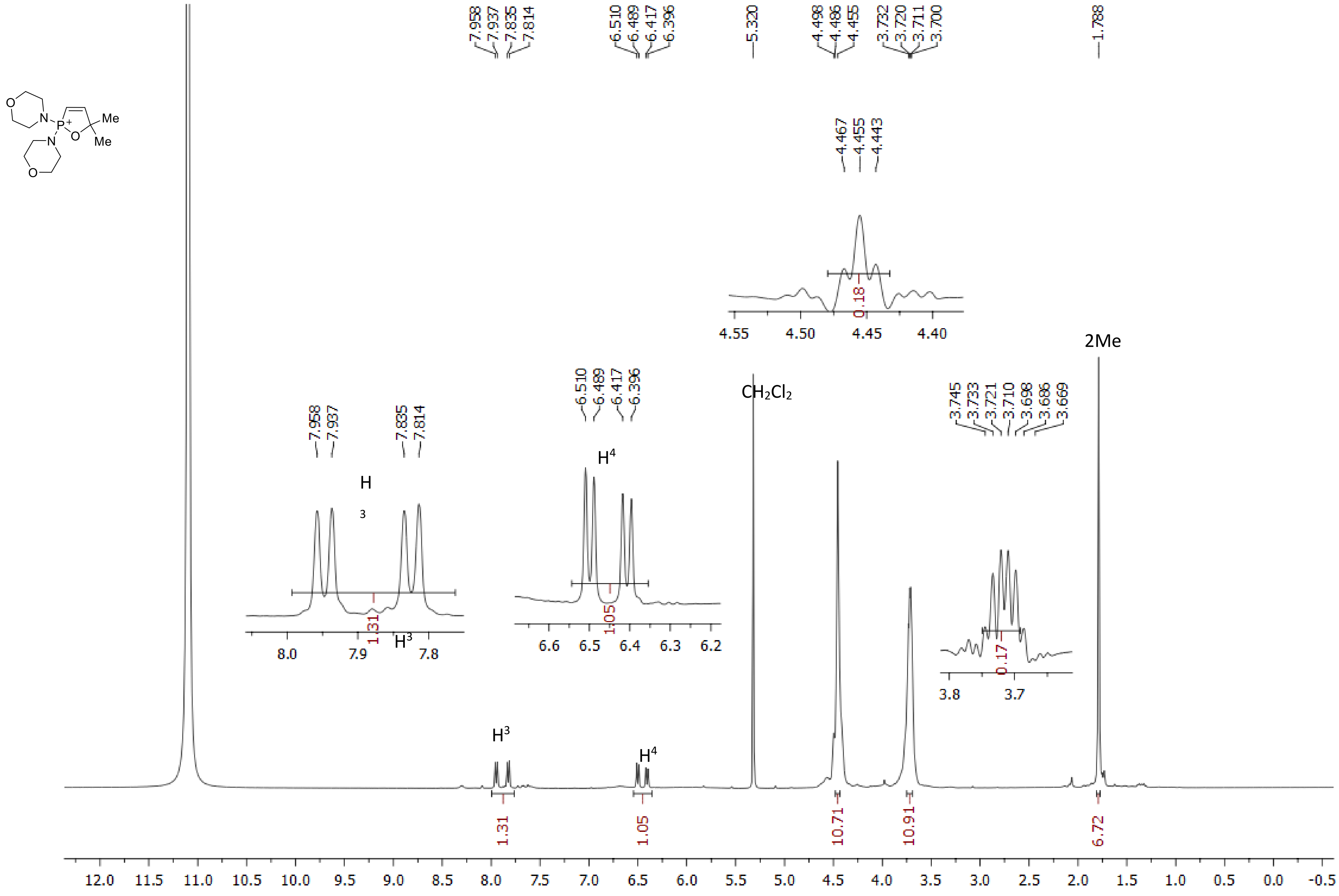
7.958
7.937
7.835
7.814
6.510
6.489
6.417
6.396
5.320
4.498
4.486
4.455
3.732
3.720
3.711
3.700
1.788
4.467
4.455
4.443
0.18
2Me
CH_2Cl_2
3.745
3.733
3.721
3.710
3.698
3.686
3.669
0.17
H^4
1.05
H
3
H^3
1.31
H^3
H^4
1.31
1.05
10.71
10.91
6.72
Me
Me
O
N
P+
O
N
O
12.0 11.5 11.0 10.5 10.0 9.5 9.0 8.5 8.0 7.5 7.0 6.5 6.0 5.5 5.0 4.5 4.0 3.5 3.0 2.5 2.0 1.5 1.0 0.5 0.0 -0.5

Figure S25. ^{1}H NMR spectrum of the compound **C** (400 MHz, TfOH).

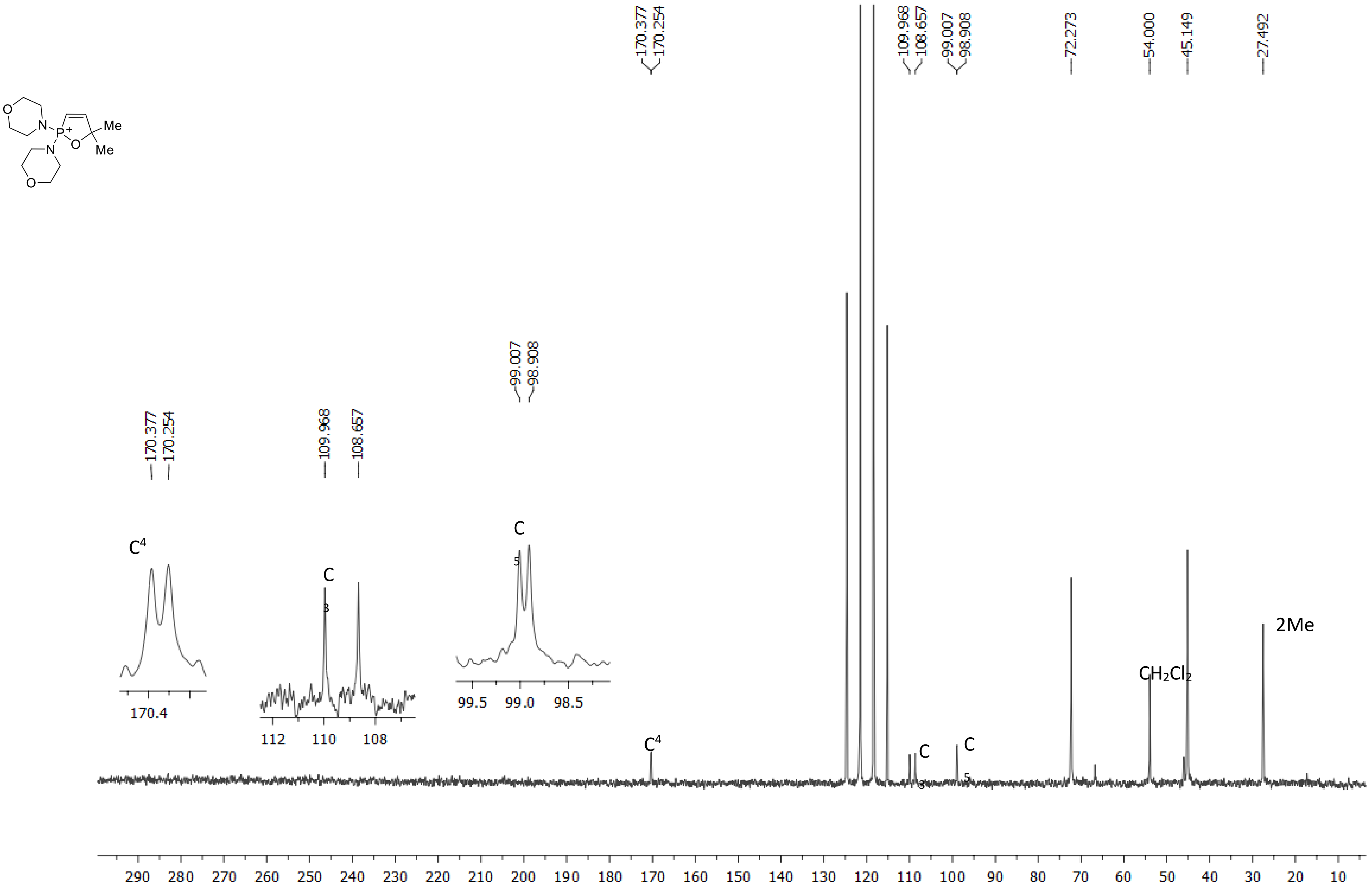

Figure S26. ^{13}C NMR spectrum of the compound **C** (101 MHz, TfOH).

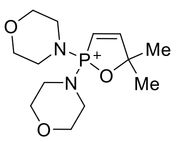

O
N
P
+
O
Me
Me
N
O

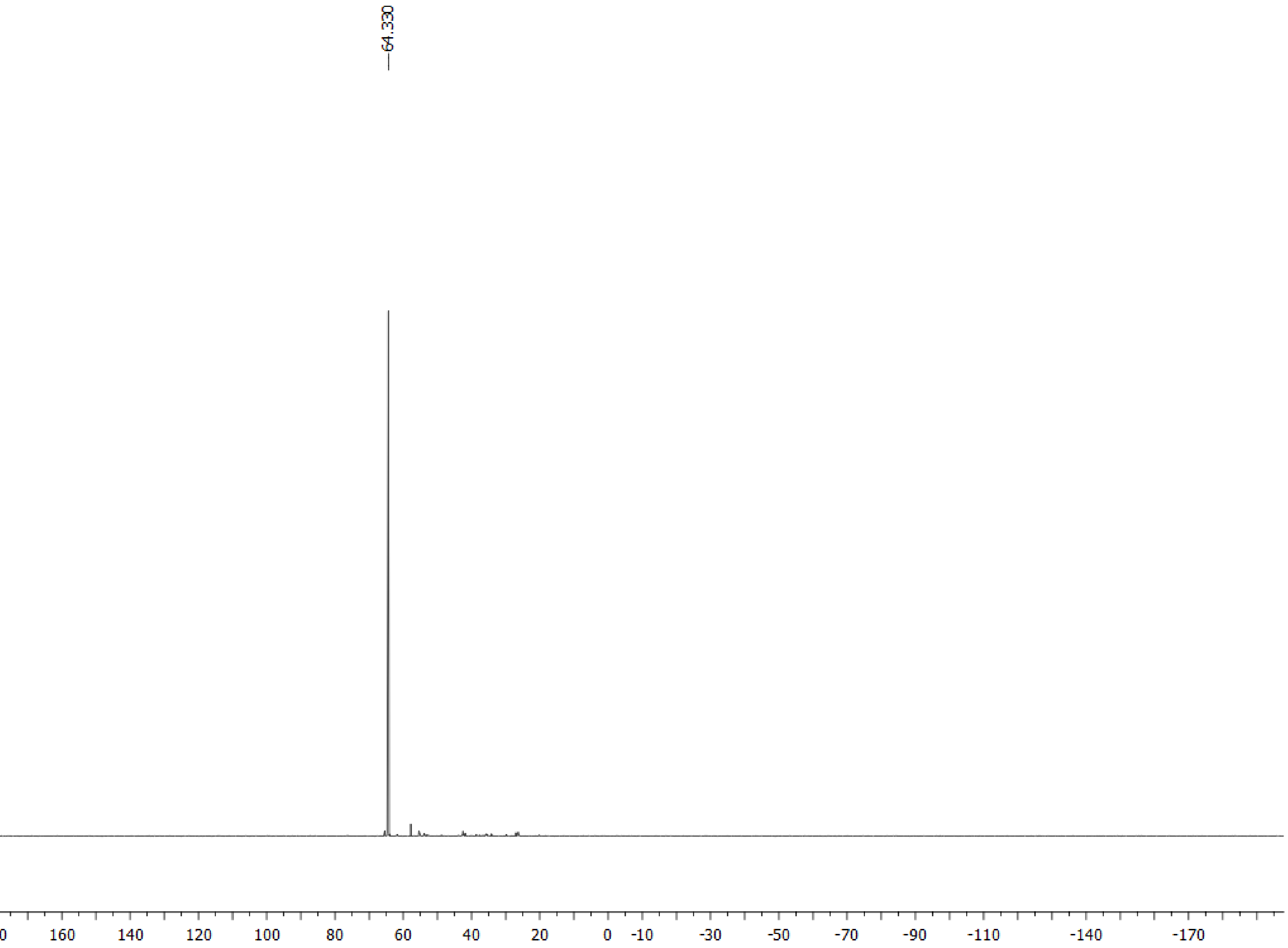

64.330
180
160
140
120
100
80
60
40
20
0
-10
-30
-50
-70
-90
-110
-140
-170

Figure S27 ^{31}P NMR spectrum of the compound **C** (162 MHz, TfOH).

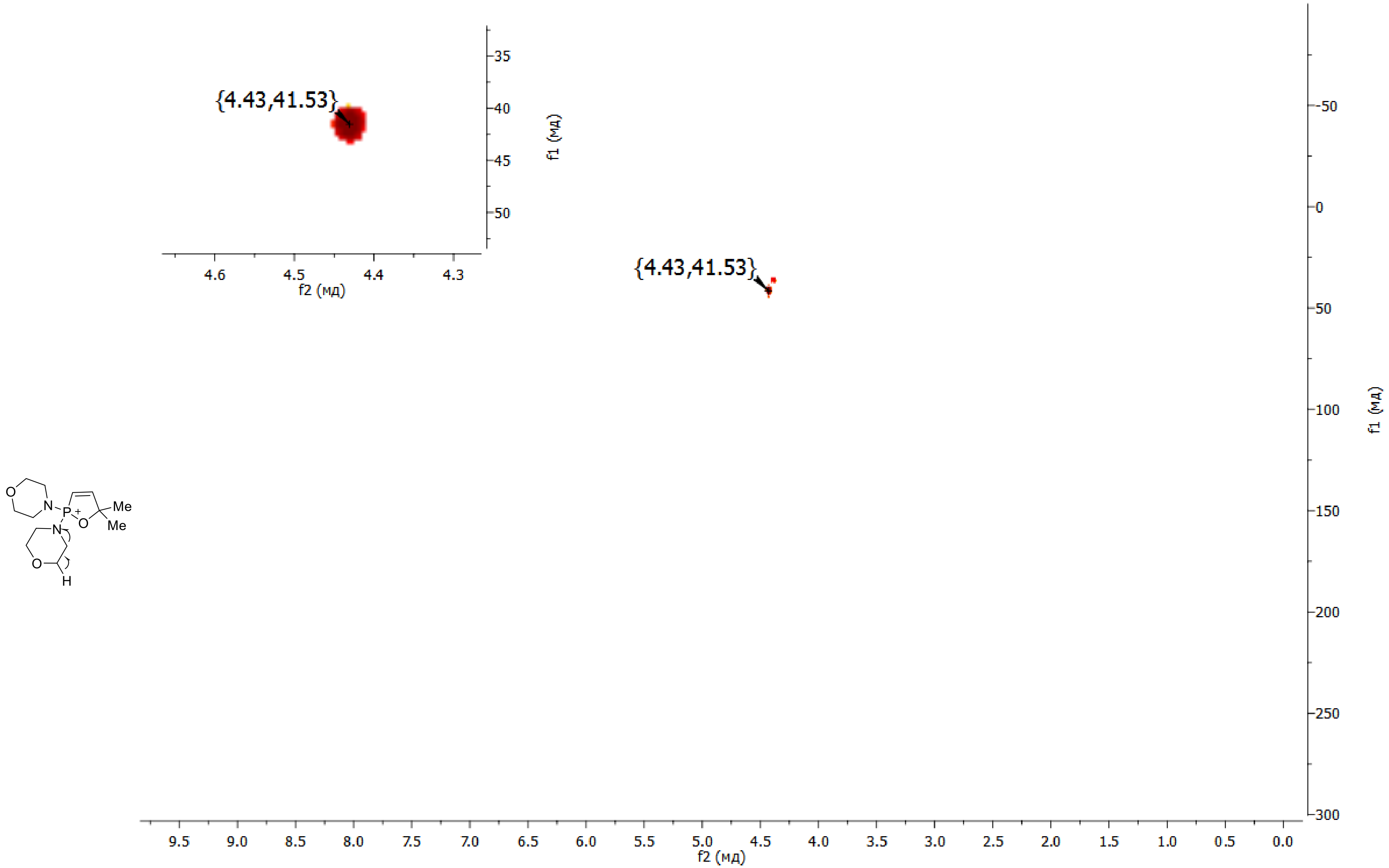

Figure S28 HMBC N-H NMR spectrum of the compound **C** (TfOH).

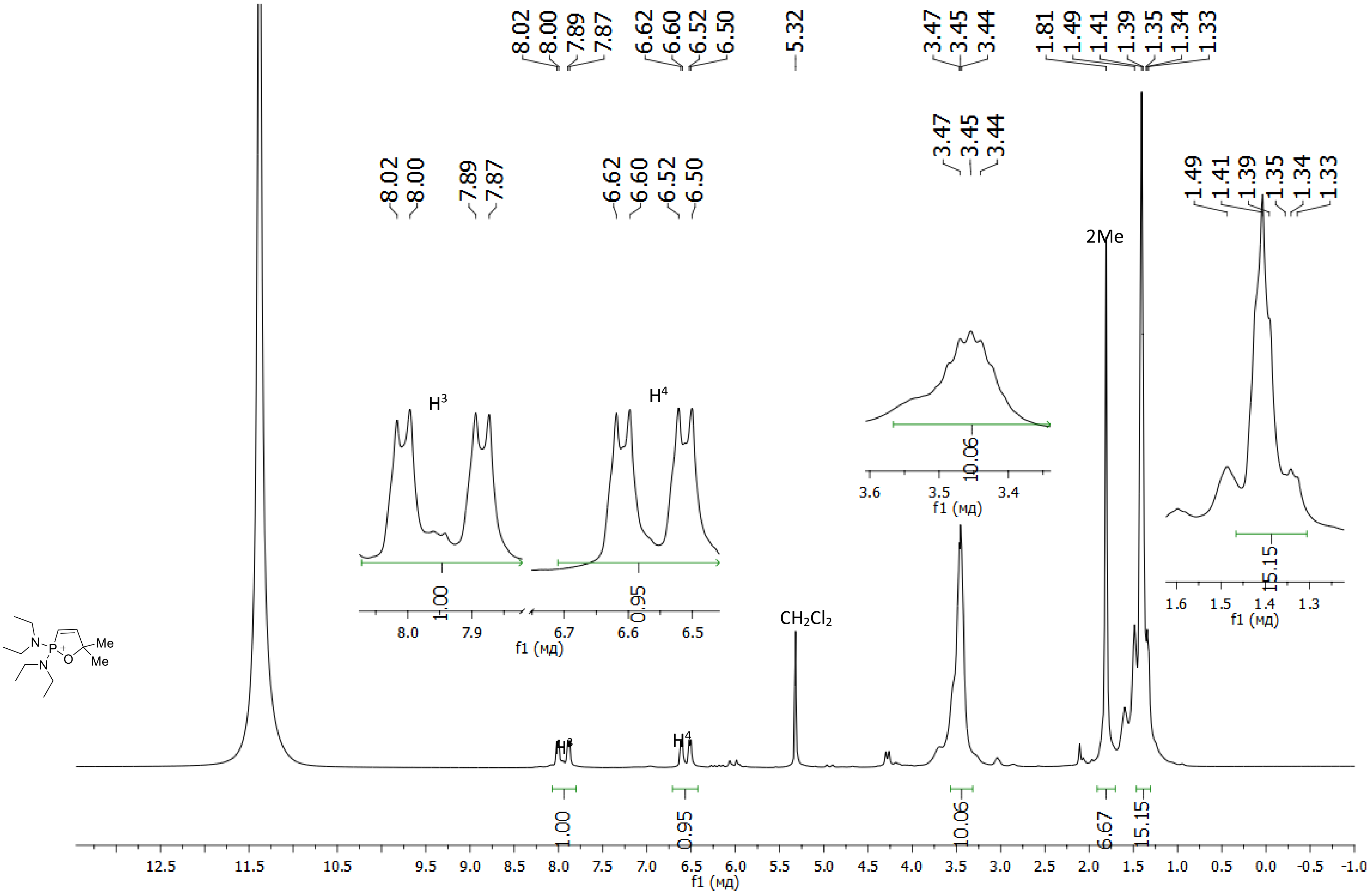

Figure S29. ^{1}H NMR spectrum of the compound **D** (400 MHz, TfOH).

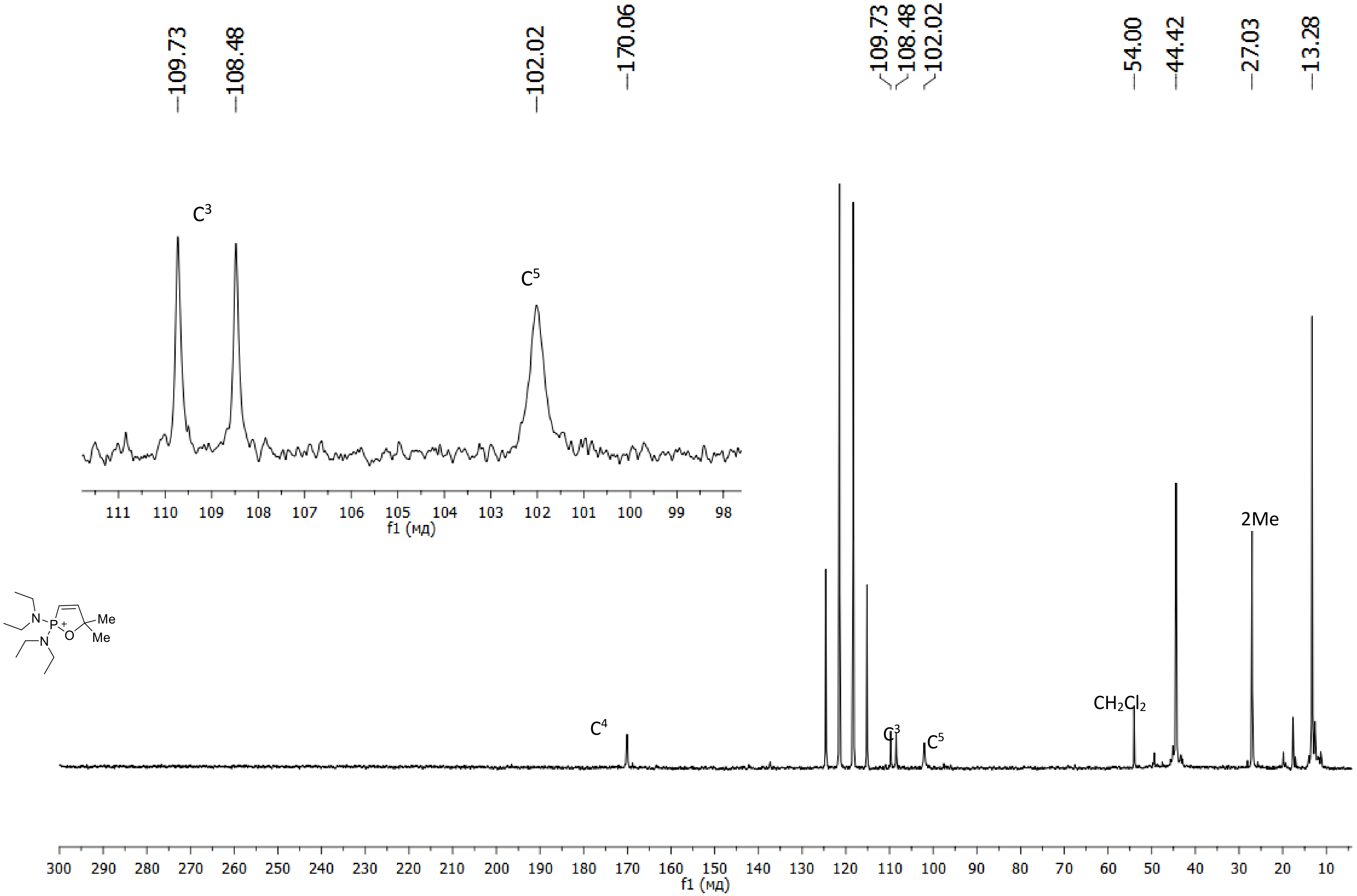

Figure S30. ^{13}C NMR spectrum of the compound **D** (101 MHz, TfOH).

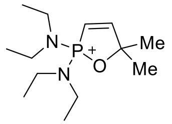

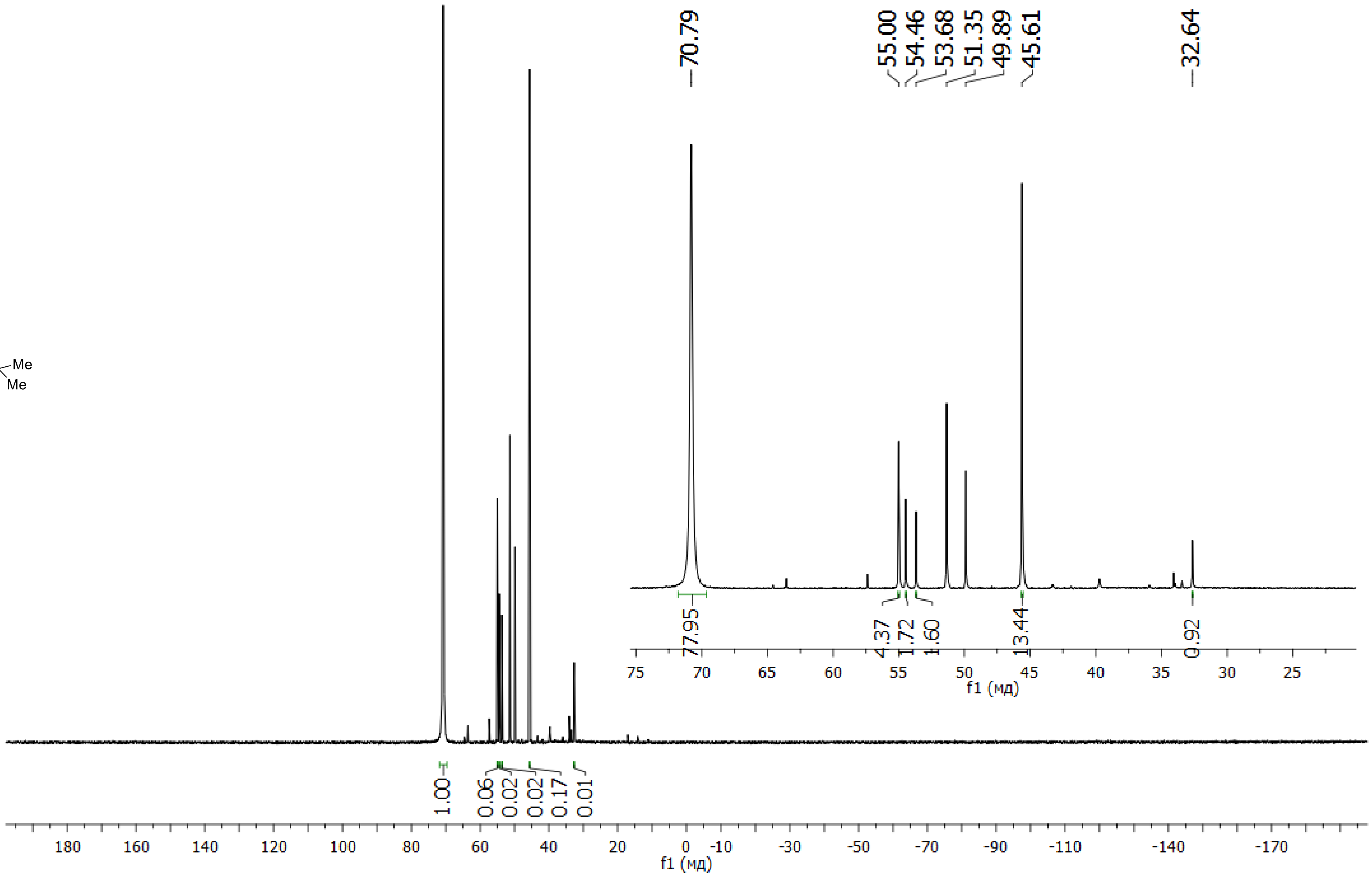

Figure S31. ^{31}P NMR spectrum of the compound **D** (162 MHz, TfOH).

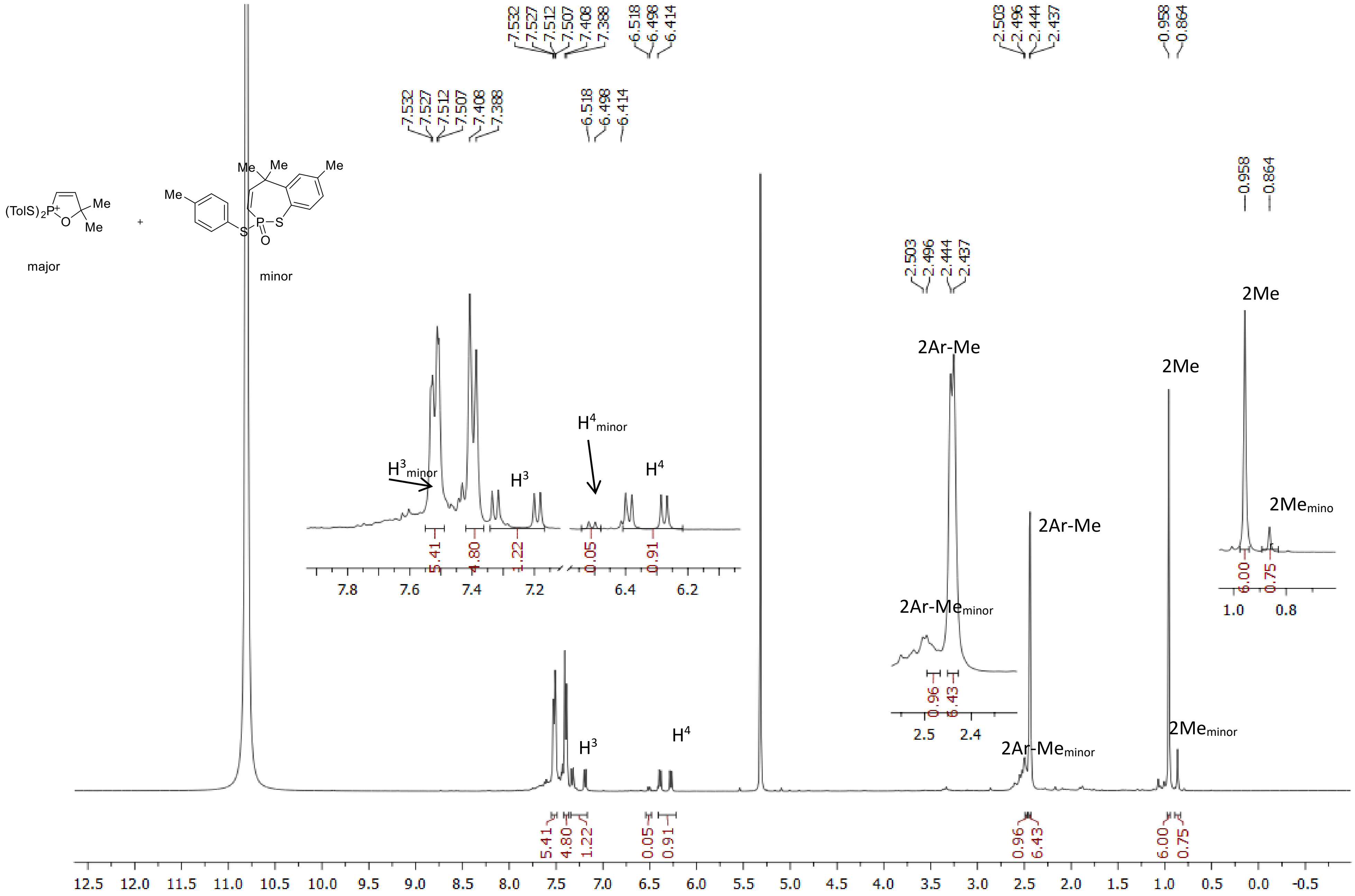

Figure S32. ^{1}H NMR spectrum of the compound **F** (400 MHz, TfOH).

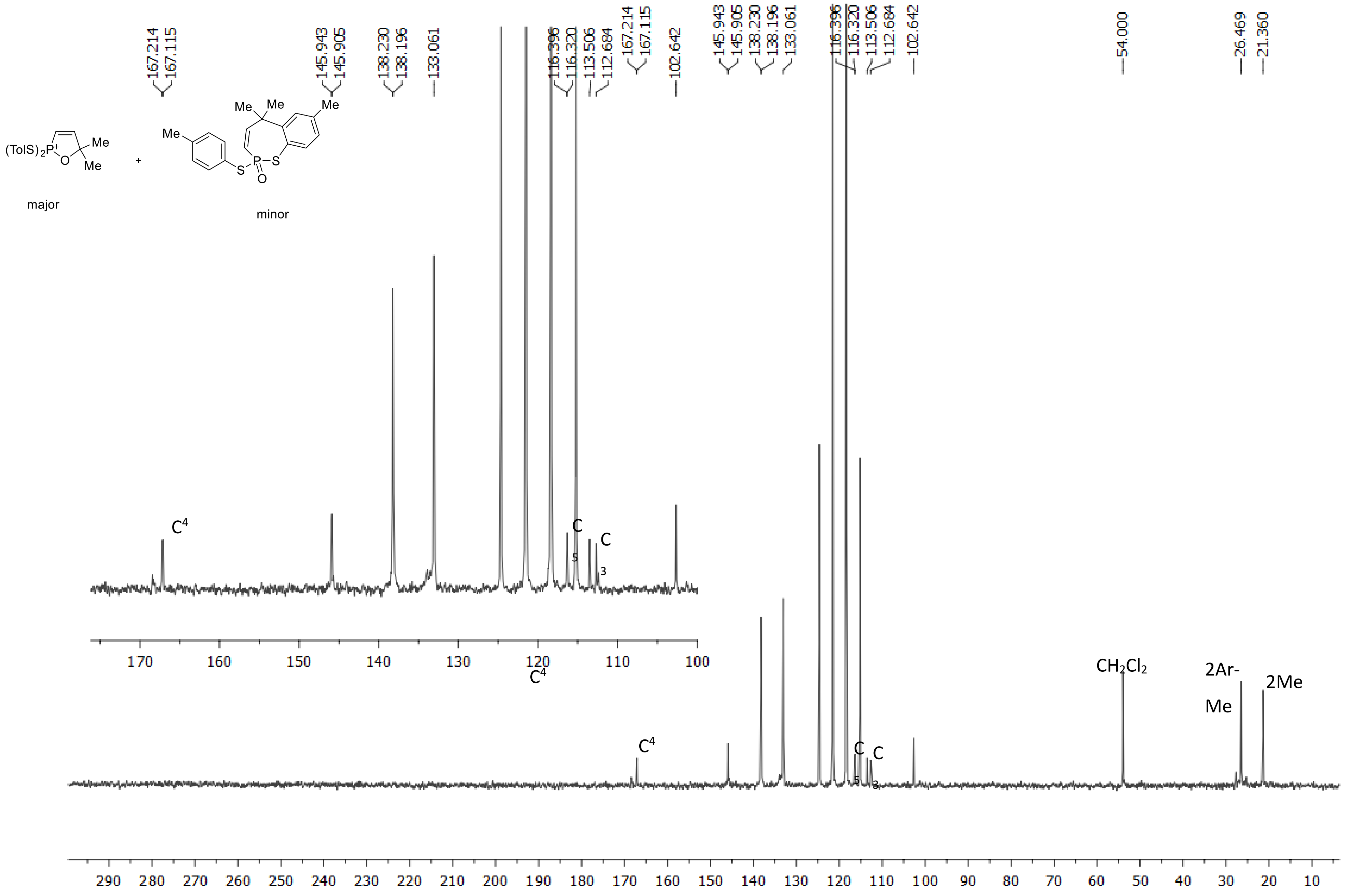

Figure S33. ^{13}C NMR spectrum of the compound **F** (101 MHz, TfOH).

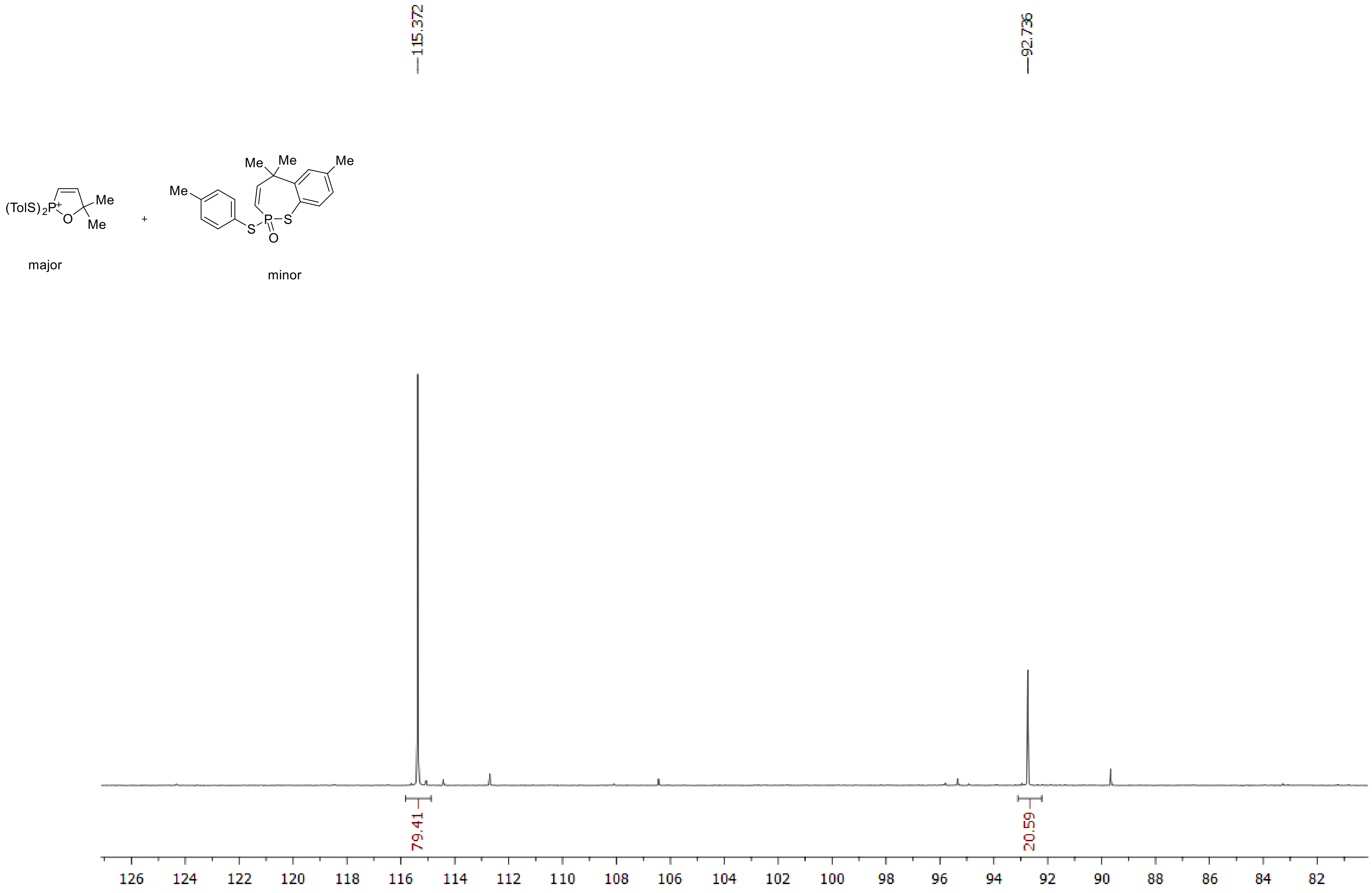

Figure S34. ^{31}P NMR spectrum of the compound **F** (162 MHz, TfOH).

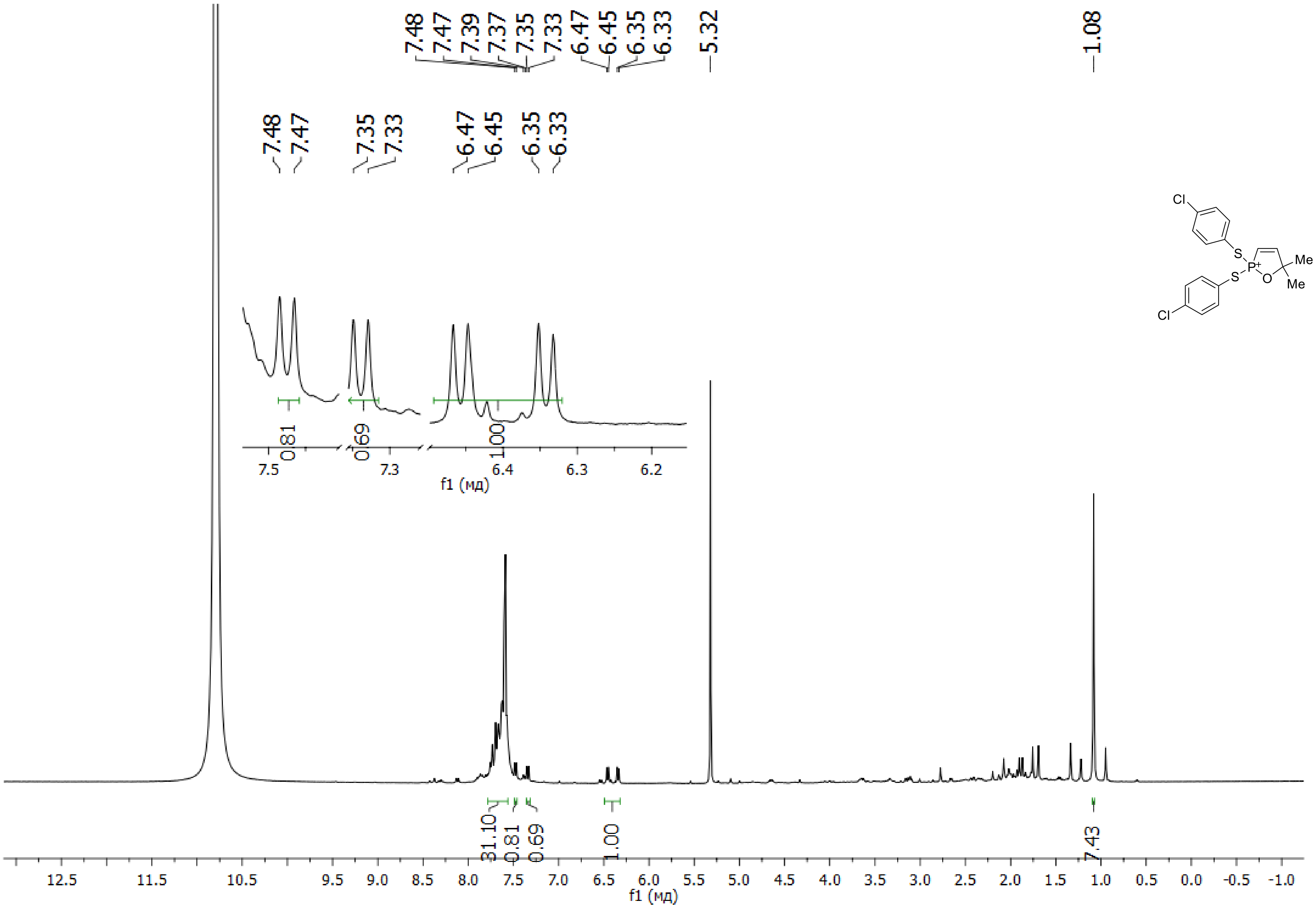

Figure S35. ^{1}H NMR spectrum of the compound **G** (400 MHz, TfOH).

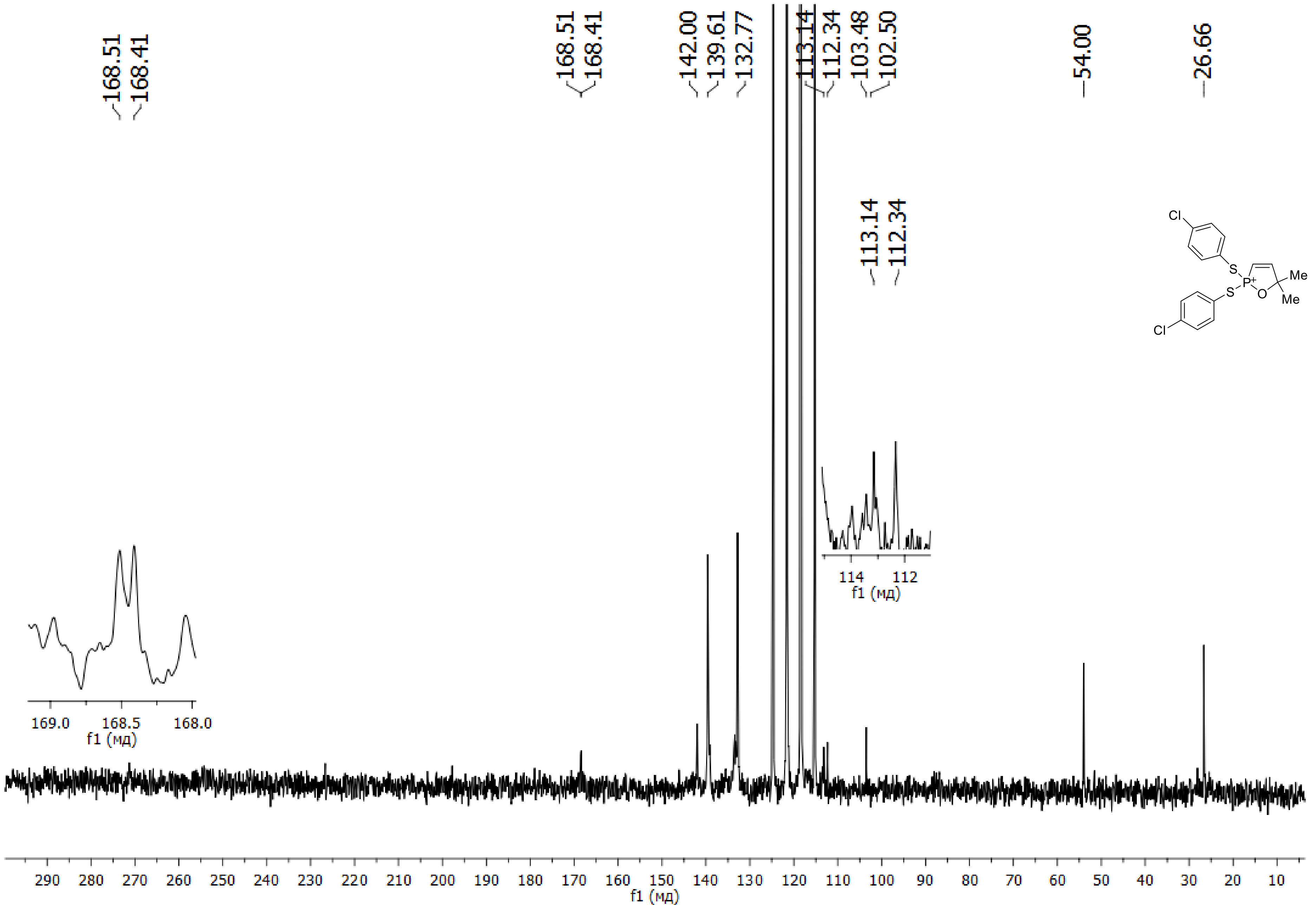

Figure S36. ^{13}C NMR spectrum of the compound **G** (101 MHz, TfOH).

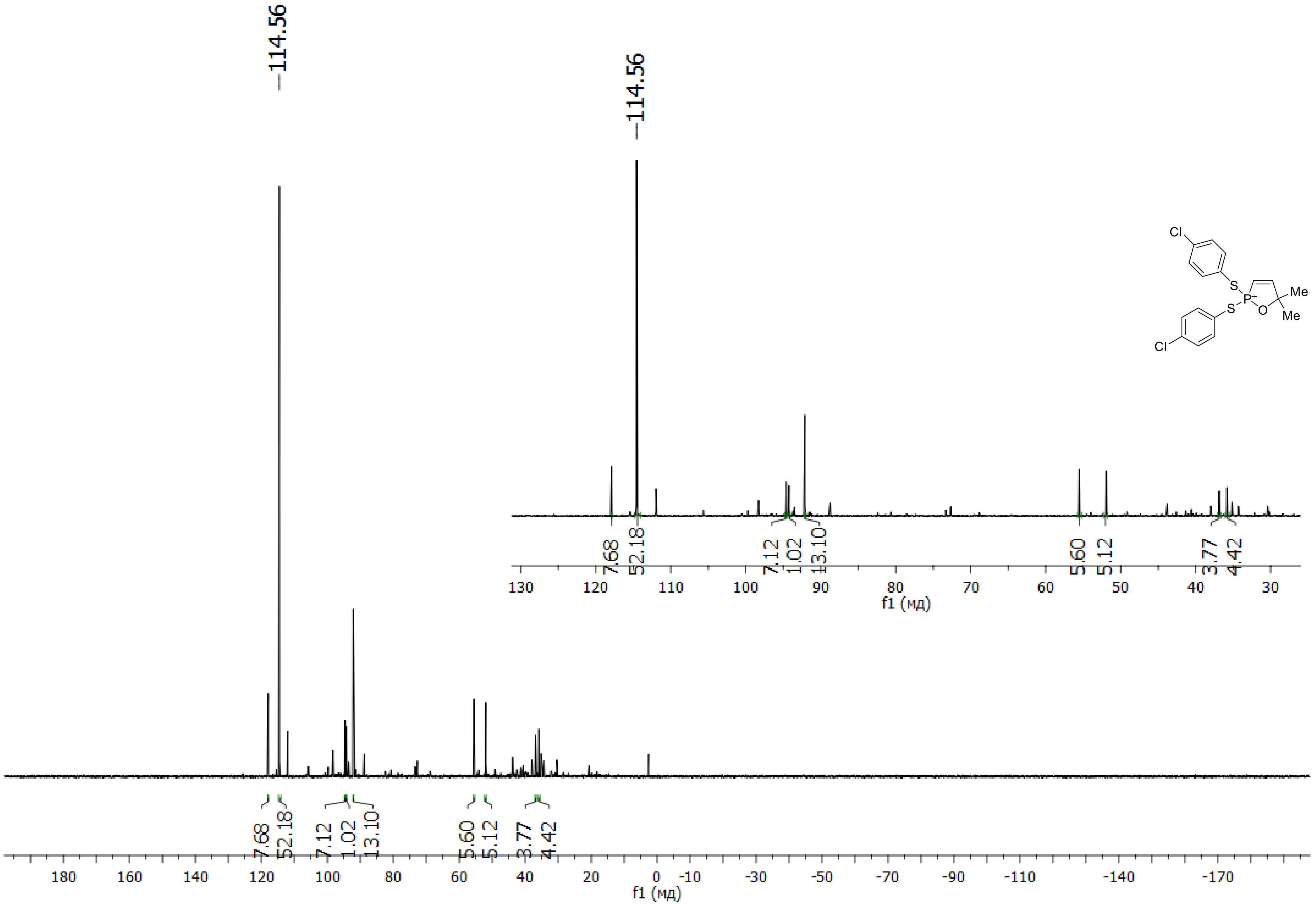

Figure S37. ^{31}P NMR spectrum of the compound **G** (162 MHz, TfOH).

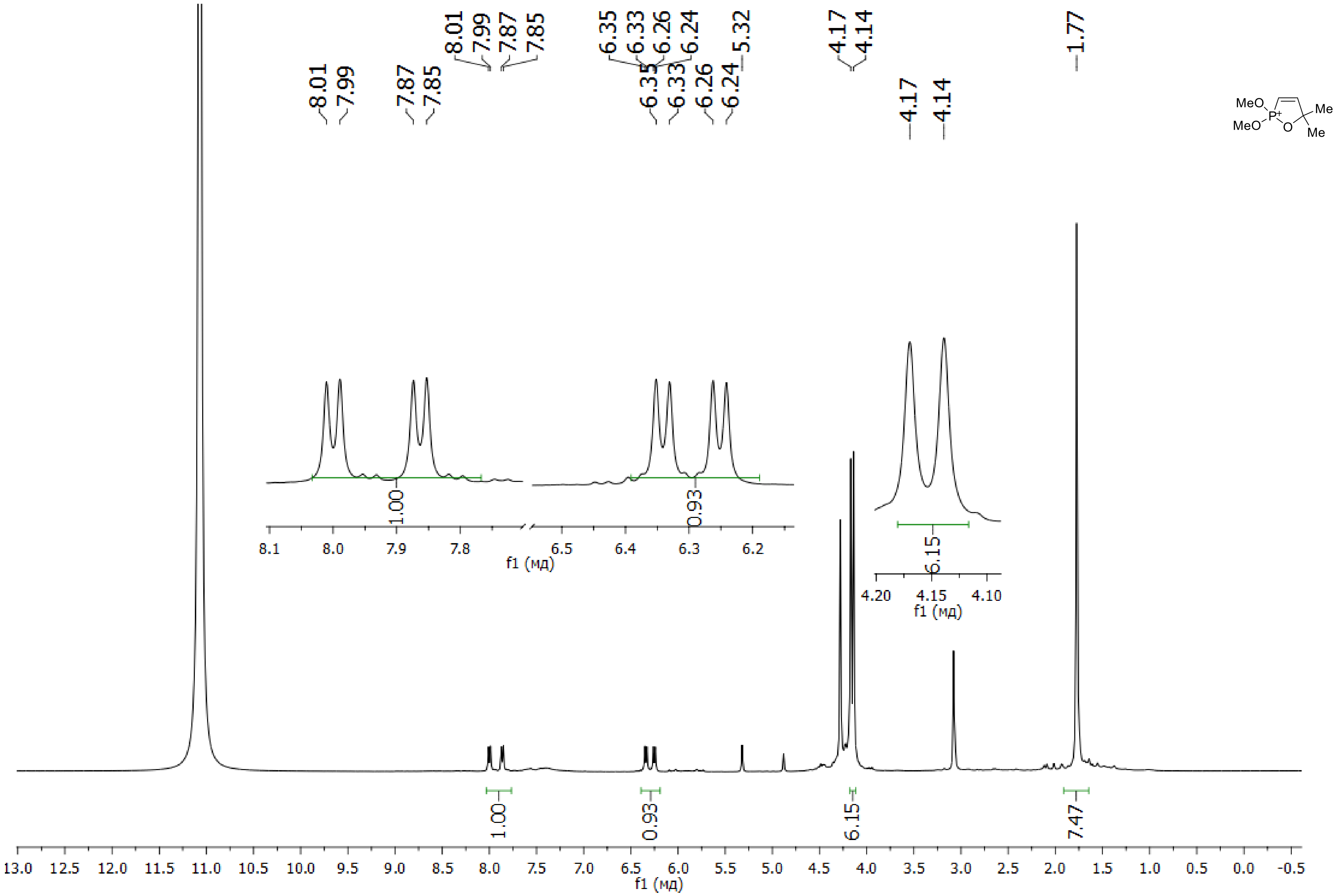

Figure S38. ^{1}H NMR spectrum of the compound **H** (400 MHz, TfOH).

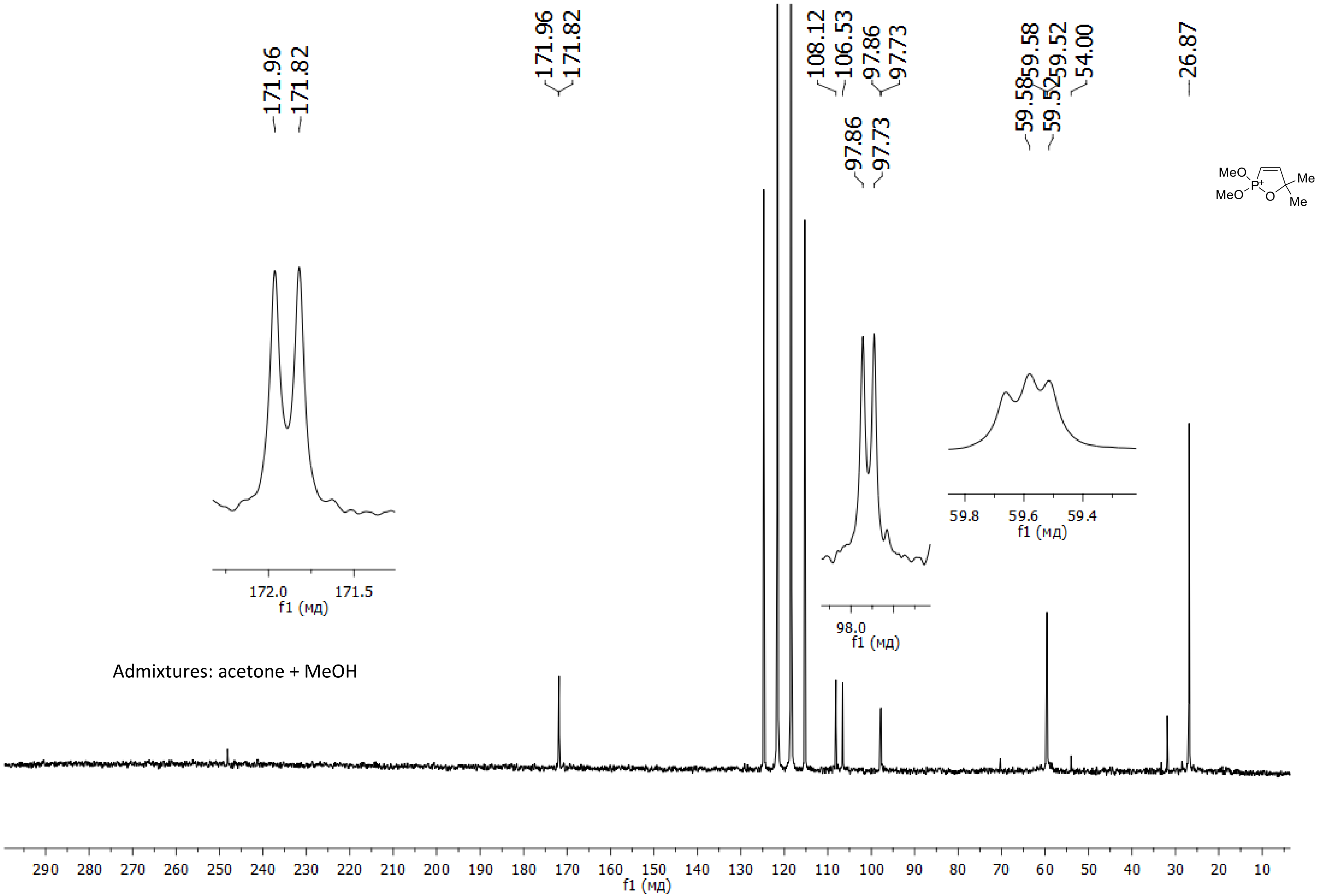

Figure S39. ^{13}C NMR spectrum of the compound **H** (101 MHz, TfOH).

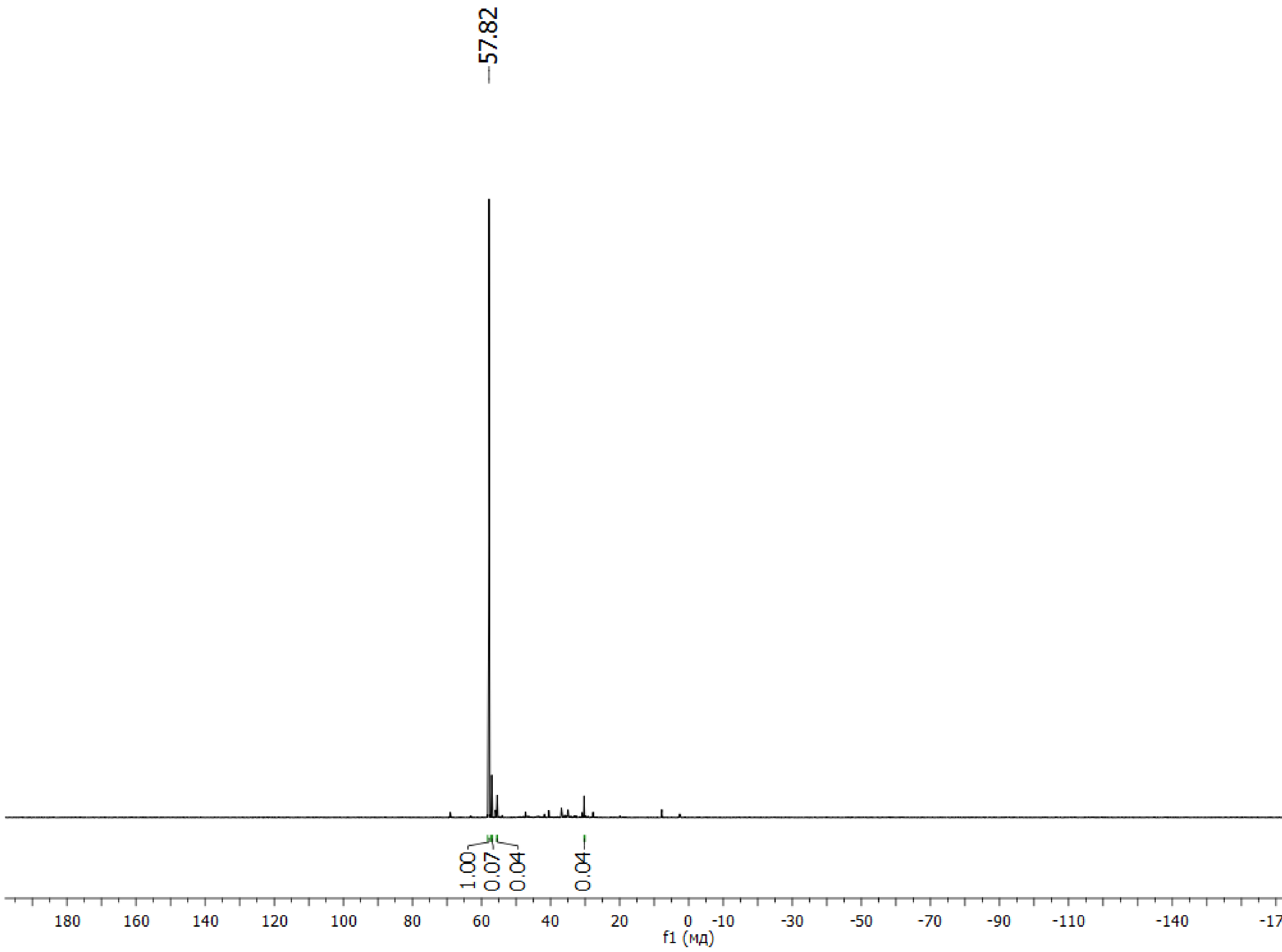

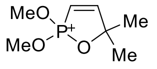

Figure S40. ^{31}P NMR spectrum of the compound **H** (162 MHz,TfOH).

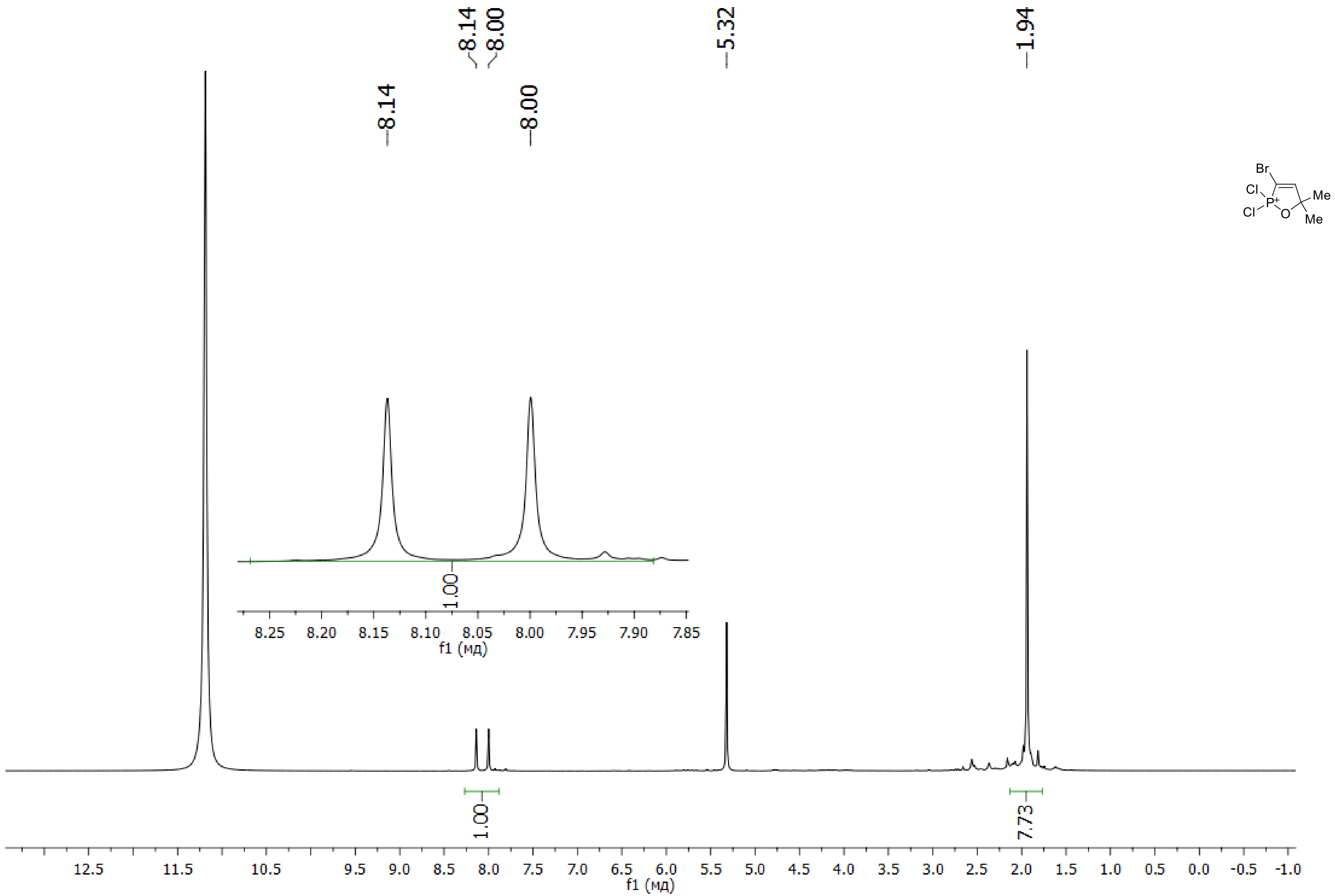

Figure S41. ^{1}H NMR spectrum of the compound **B** (400 MHz, TfOH).

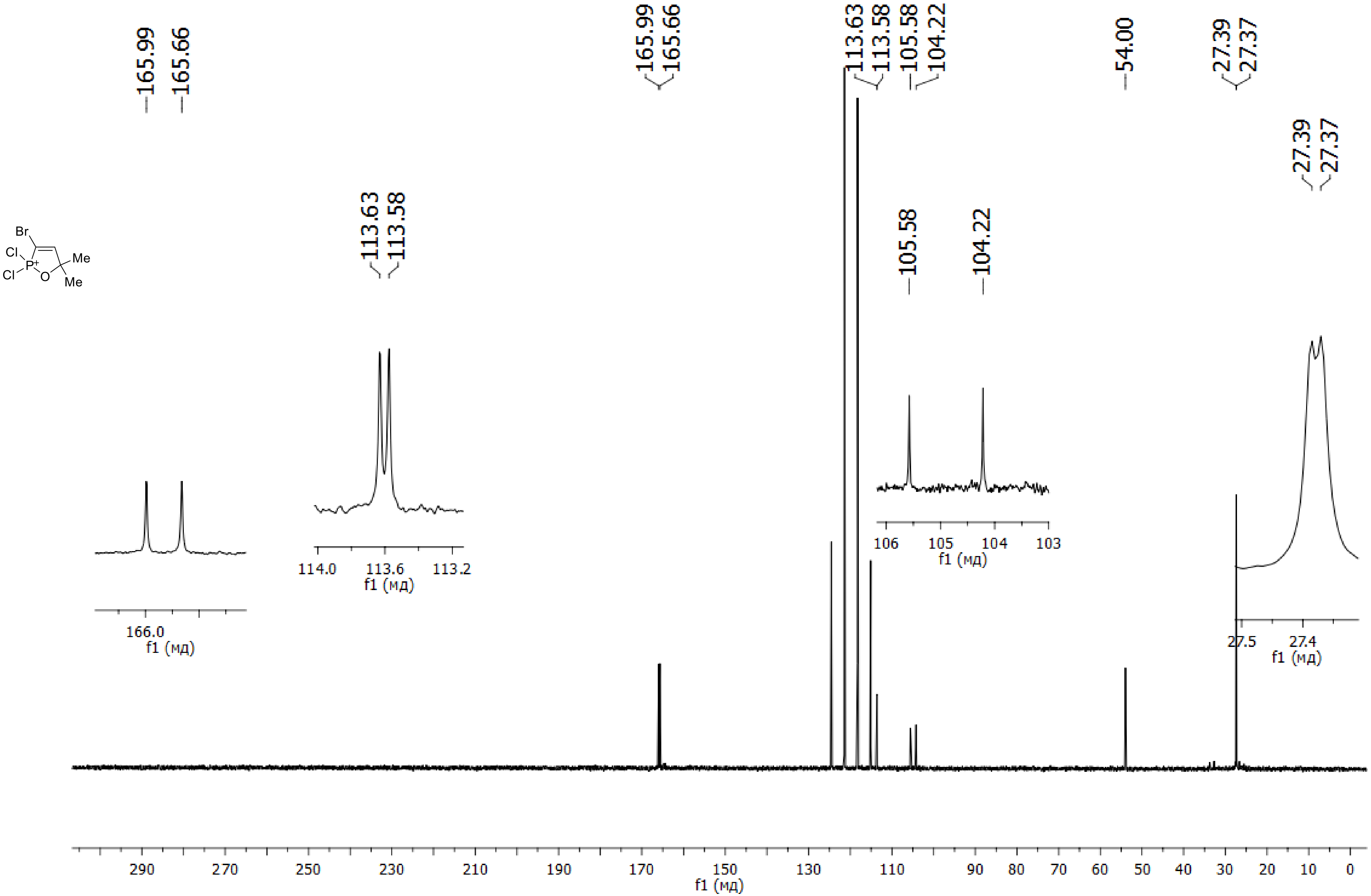

Figure S42. ^{13}C NMR spectrum of the compound **B** (101 MHz, TfOH).

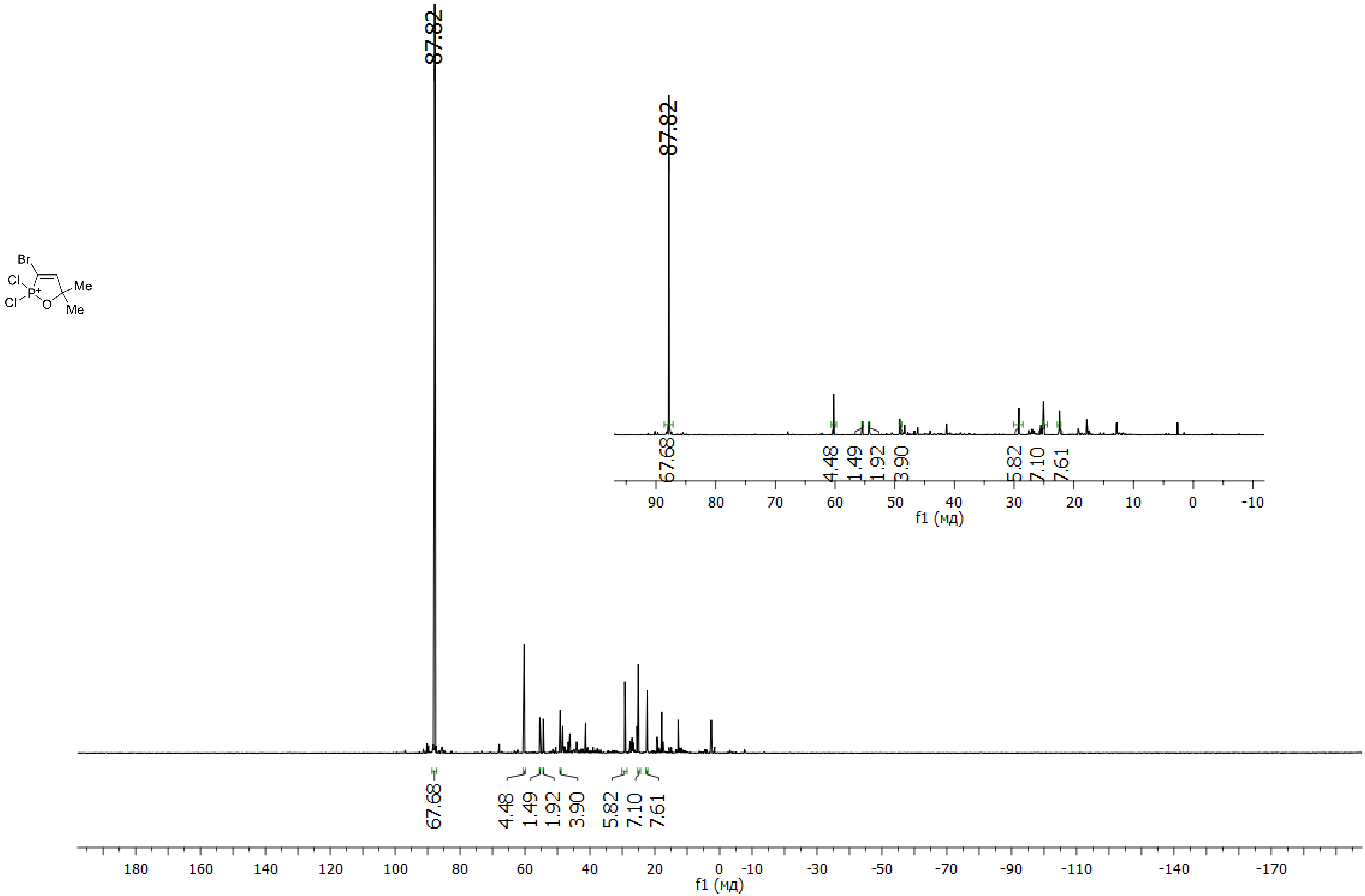

Figure S43. ^{31}P NMR spectrum of the compound **B** (162 MHz,TfOH).

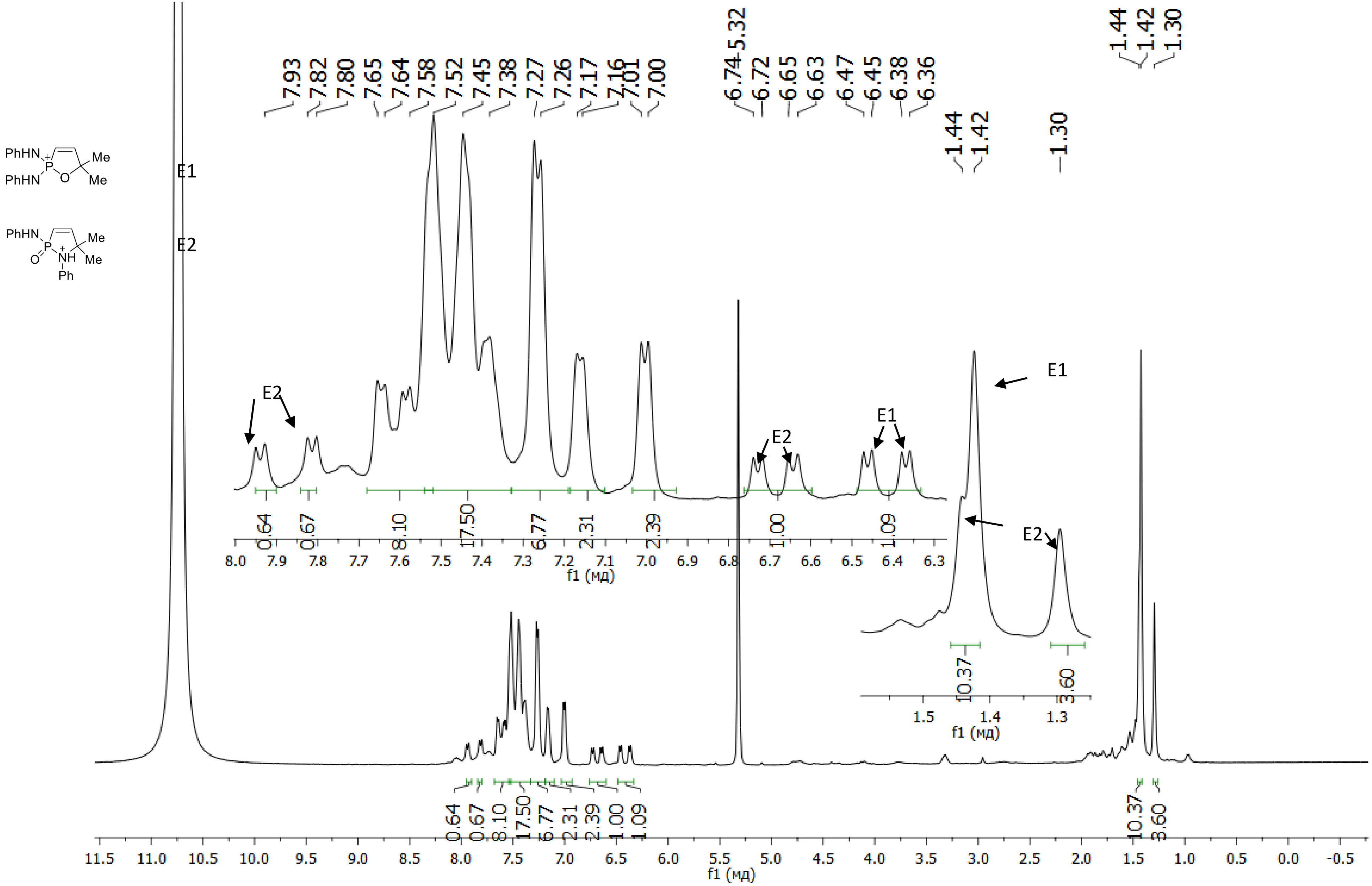
PhHN
PhHN
Me
Me
E1
E2
f1 (мд)

Figure S44. ^{1}H NMR spectrum of **1g** after **1 min** (400 MHz, TfOH).

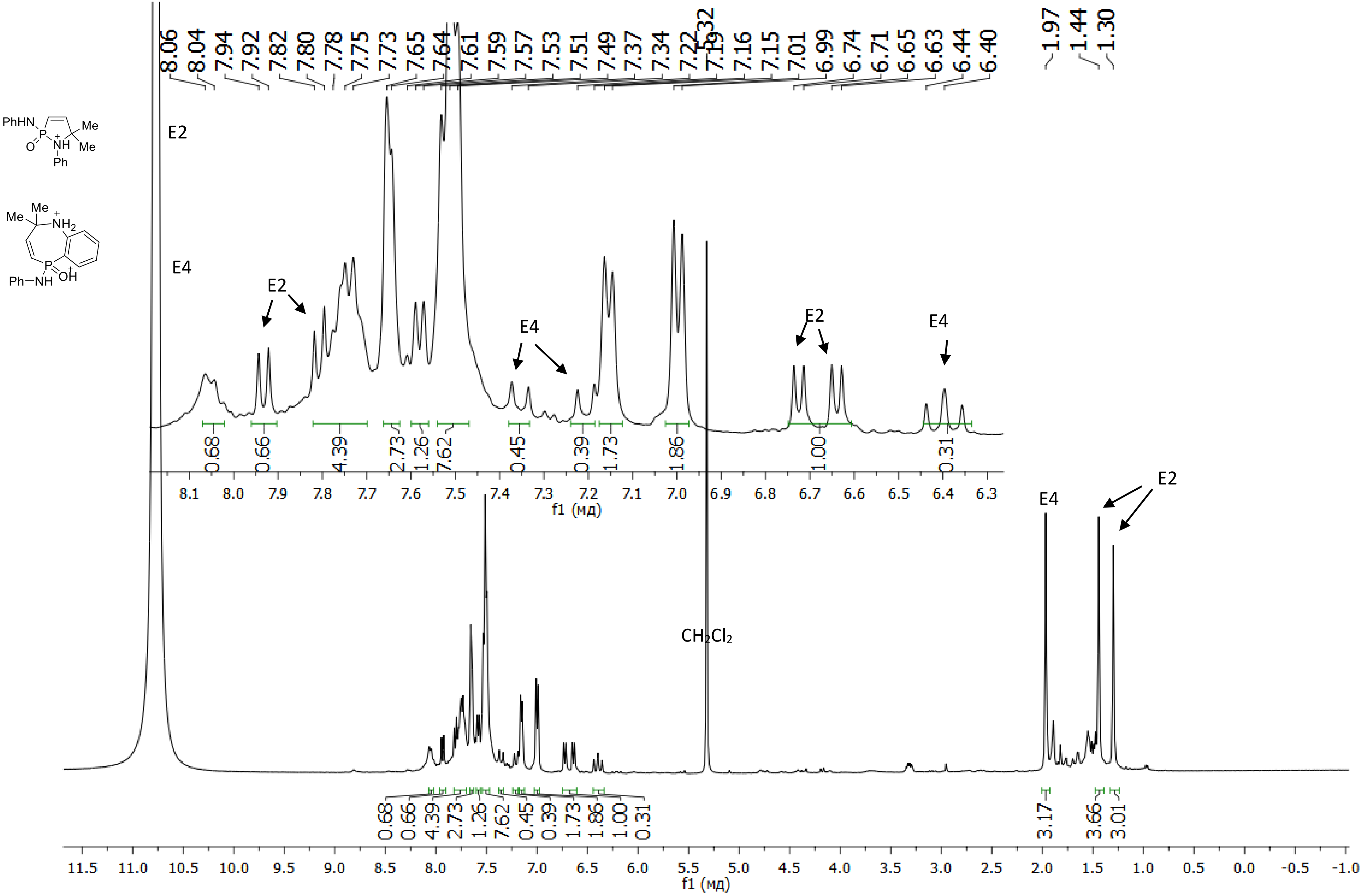

Figure S45. ^{1}H NMR spectrum of **1g** after **2 days** (400 MHz, TfOH).

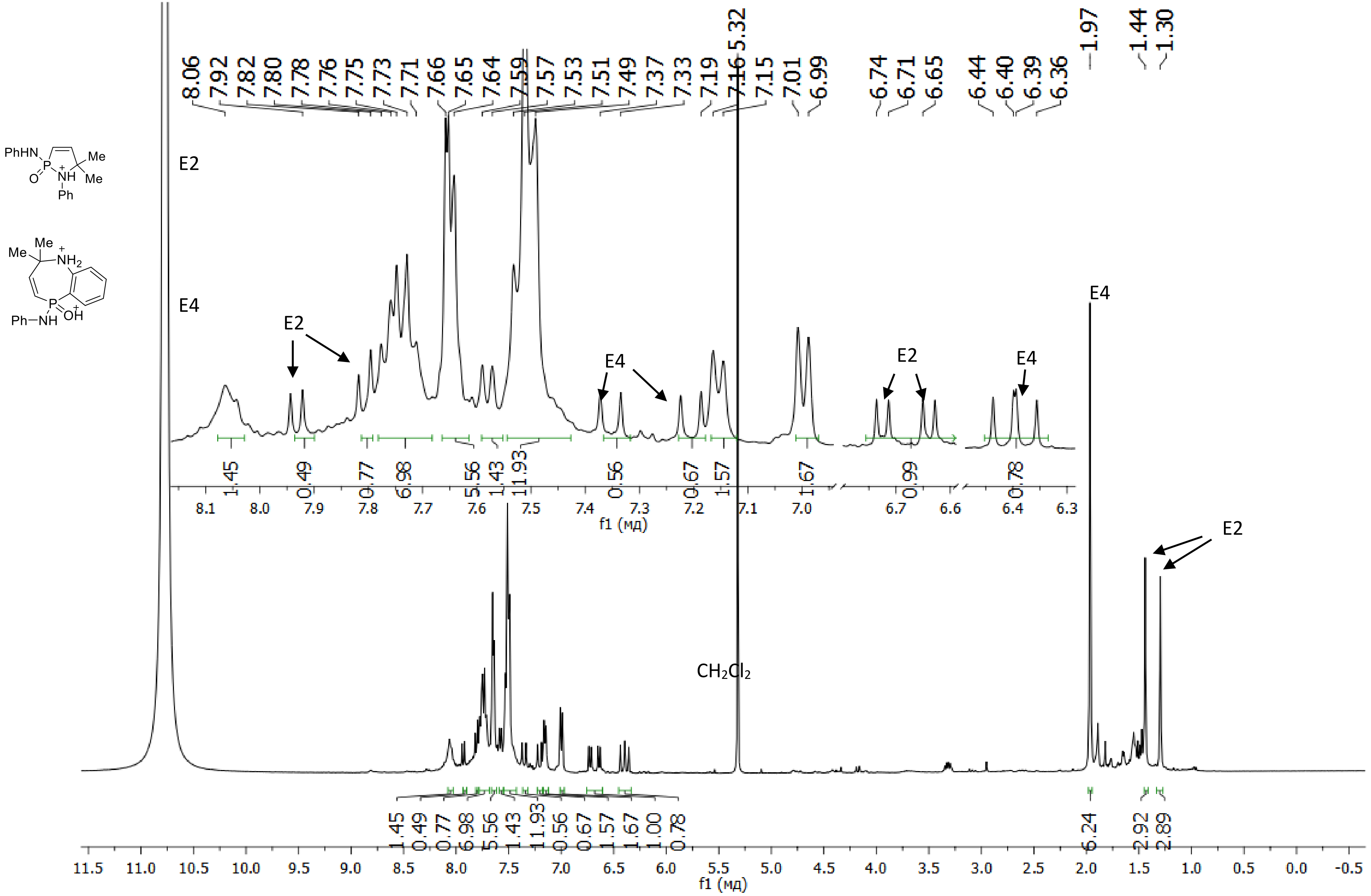

Figure S46. ^{1}H NMR spectrum of **1g** after **4 days** (400 MHz, TfOH).

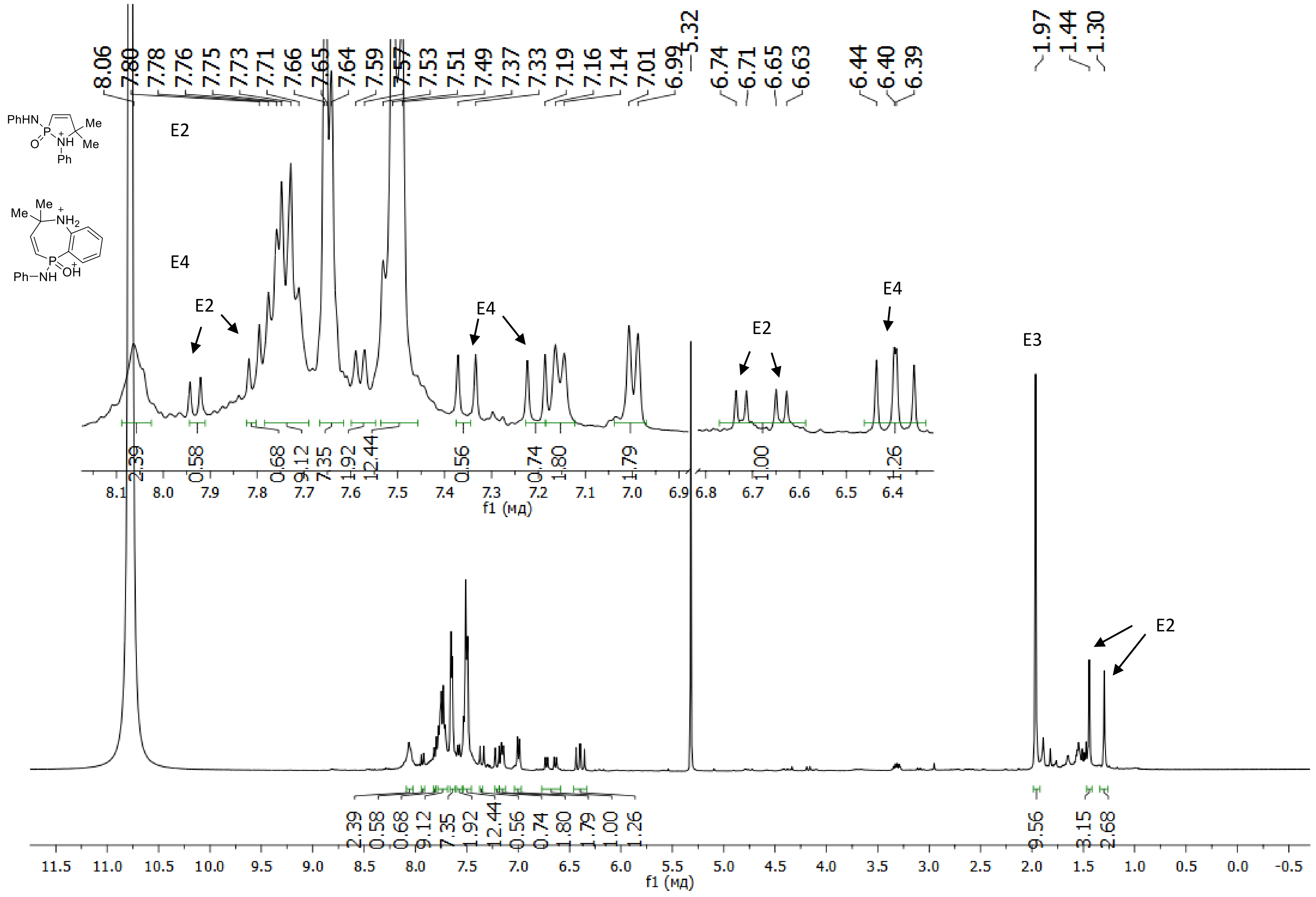
E2
E4
E3
f1 (мд)

Figure S47. ^{1}H NMR spectrum of **1g** after **6 days** (400 MHz, TfOH).

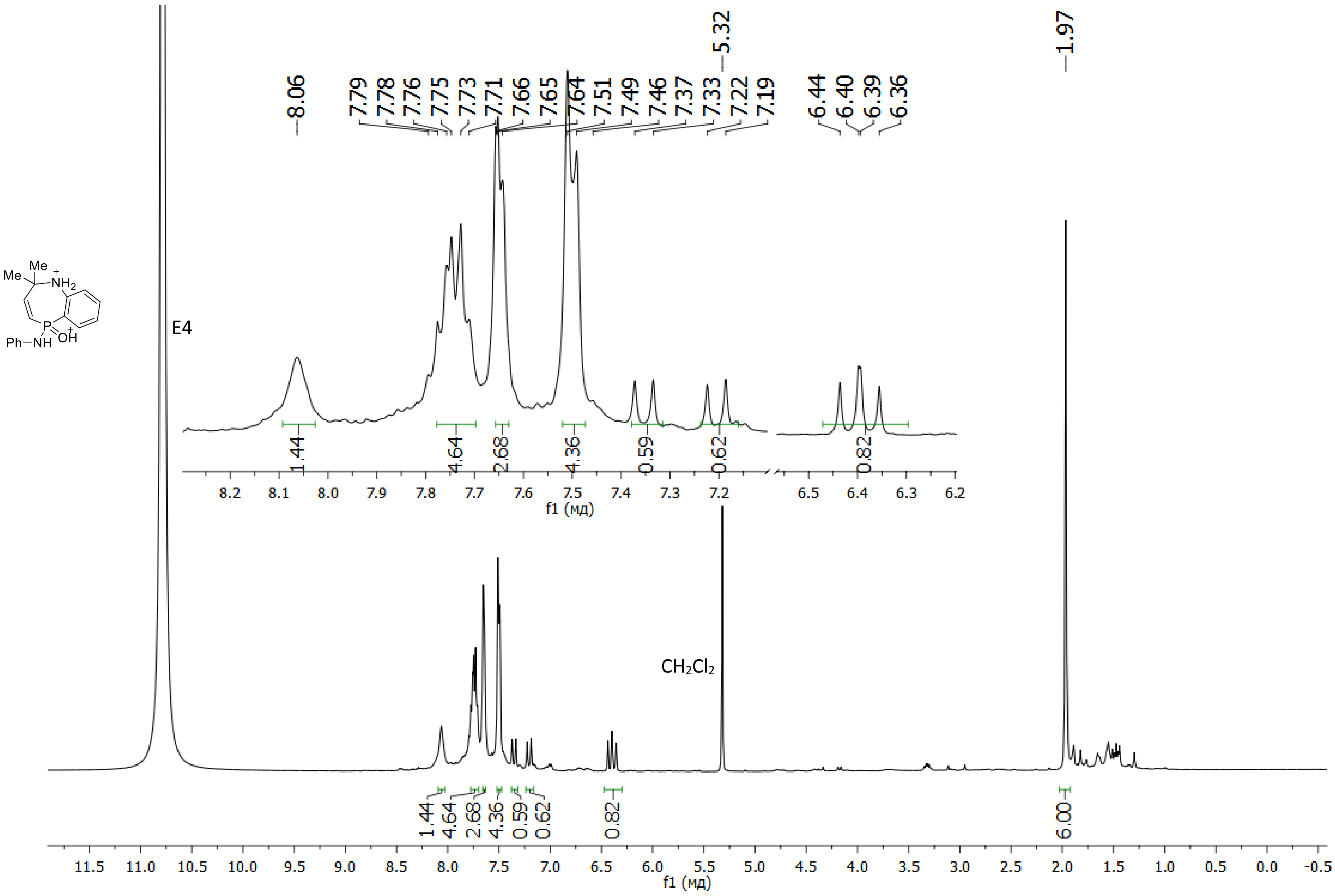

Figure S48. ^{1}H NMR spectrum of **1d** after **12 days** (400 MHz, TfOH).

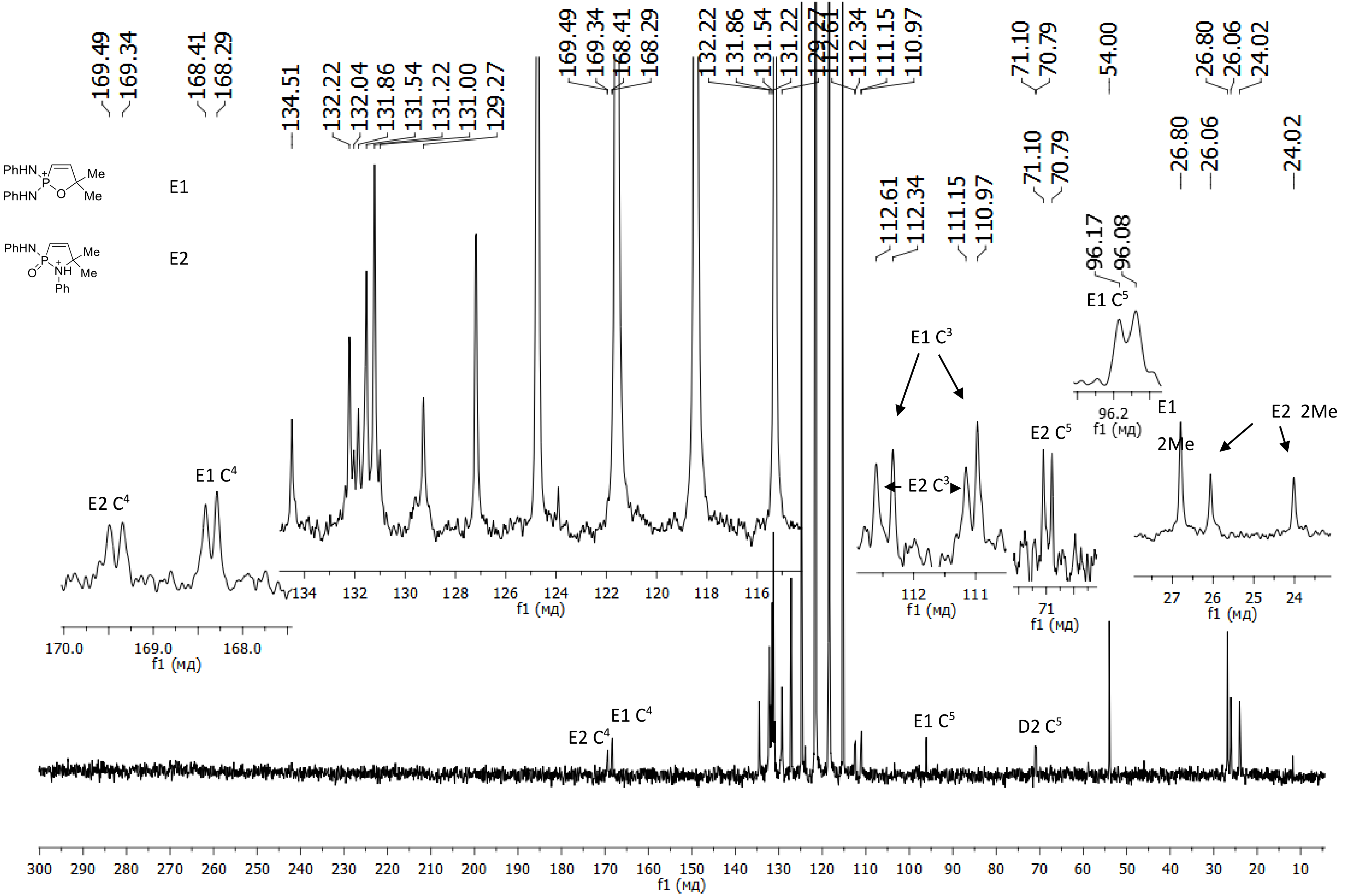

E1
E2
PhHN
Me
O
Ph
E2 C4
E1 C4
E1 C3
E2 C3
E1 C5
E2 C5
D2 C5
E1 2Me
E2 2Me
169.49
169.34
168.41
168.29
134.51
132.22
132.04
131.86
131.54
131.22
131.00
129.27
112.61
112.34
111.15
110.97
96.17
96.08
71.10
70.79
54.00
26.80
26.06
24.02
f1 (мд)

Figure S49. ^{13}C NMR spectrum of **1g** after **1 min** (101 MHz, TfOH).

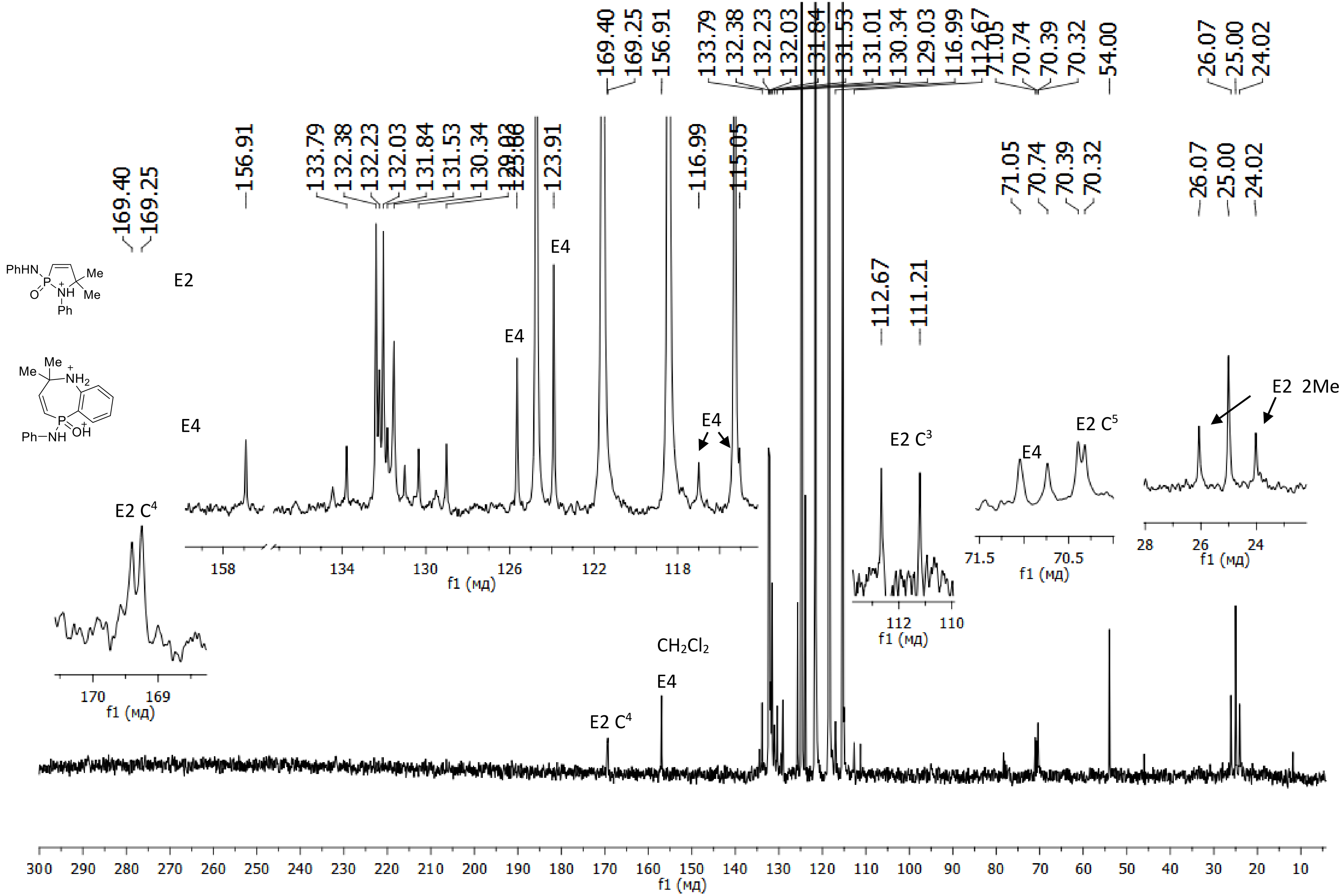

Figure S50. ^{13}C NMR spectrum of **1g** after **4 days** (101 MHz, TfOH).

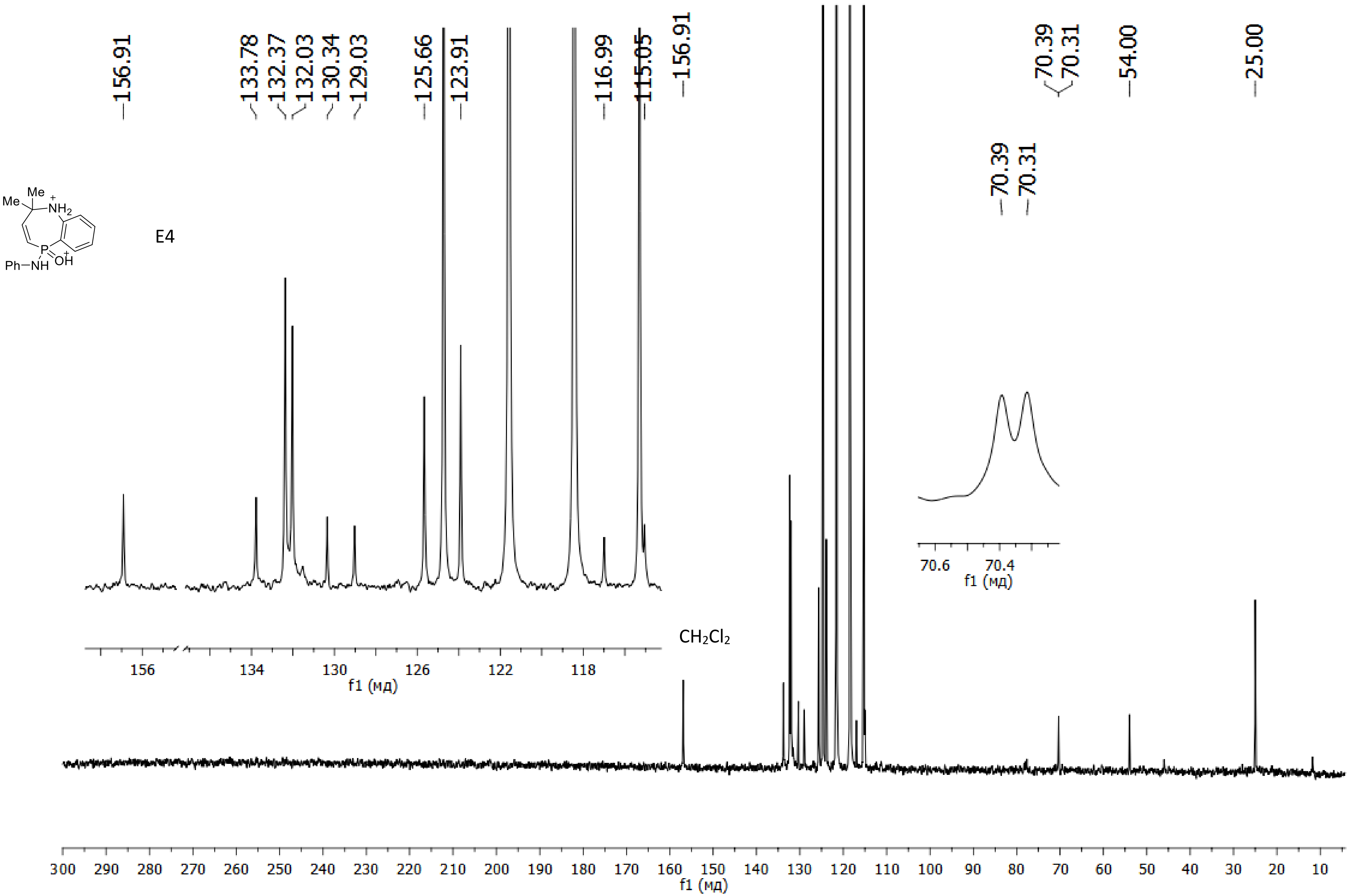

Figure S51. ^{13}C NMR spectrum of **1g** after **12 days** (101 MHz, TfOH).

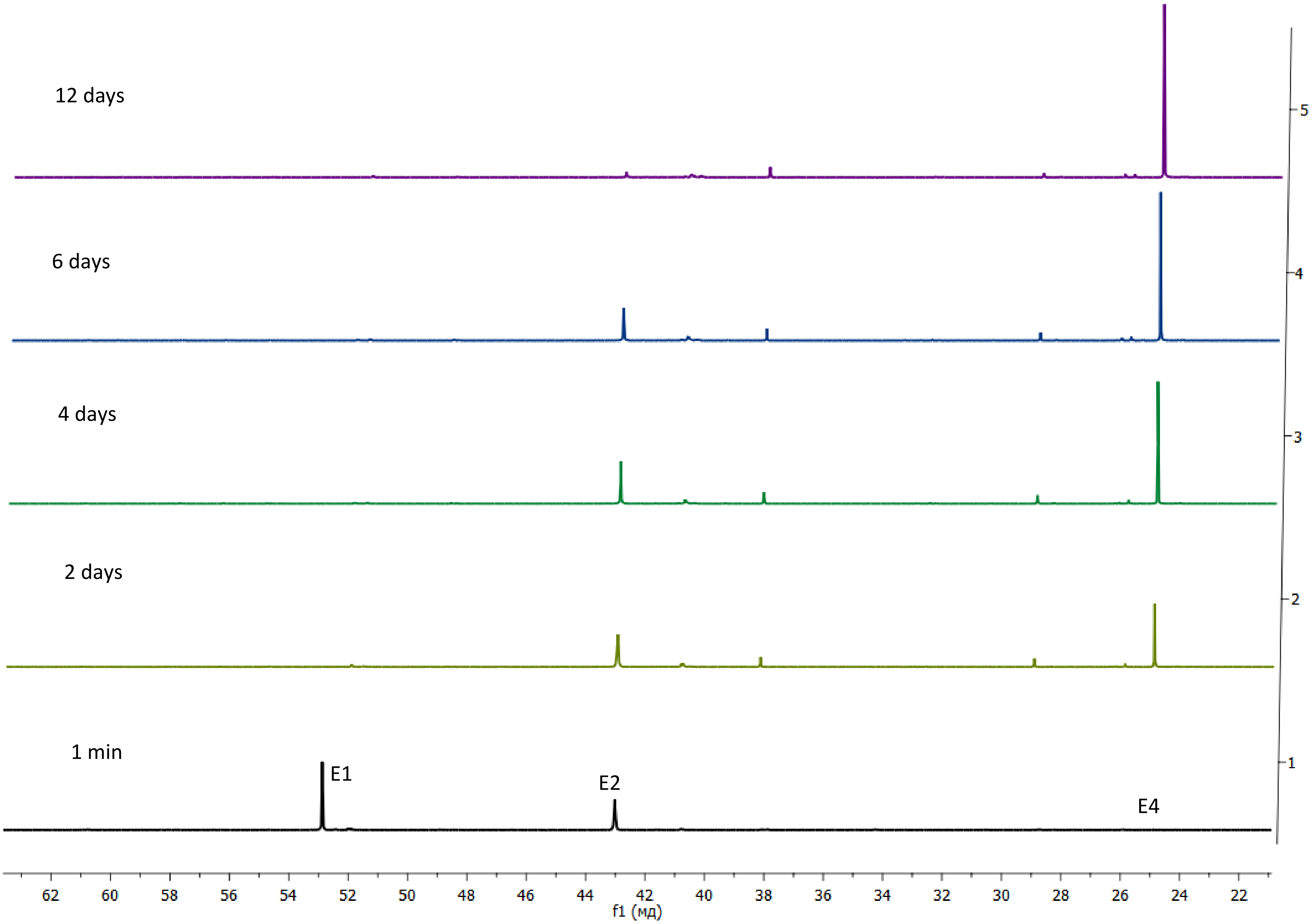

Figure S52. ^{31}P NMR spectrum of **1g** (162 MHz, TfOH).

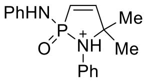

E2

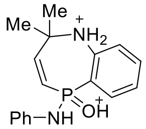

E4

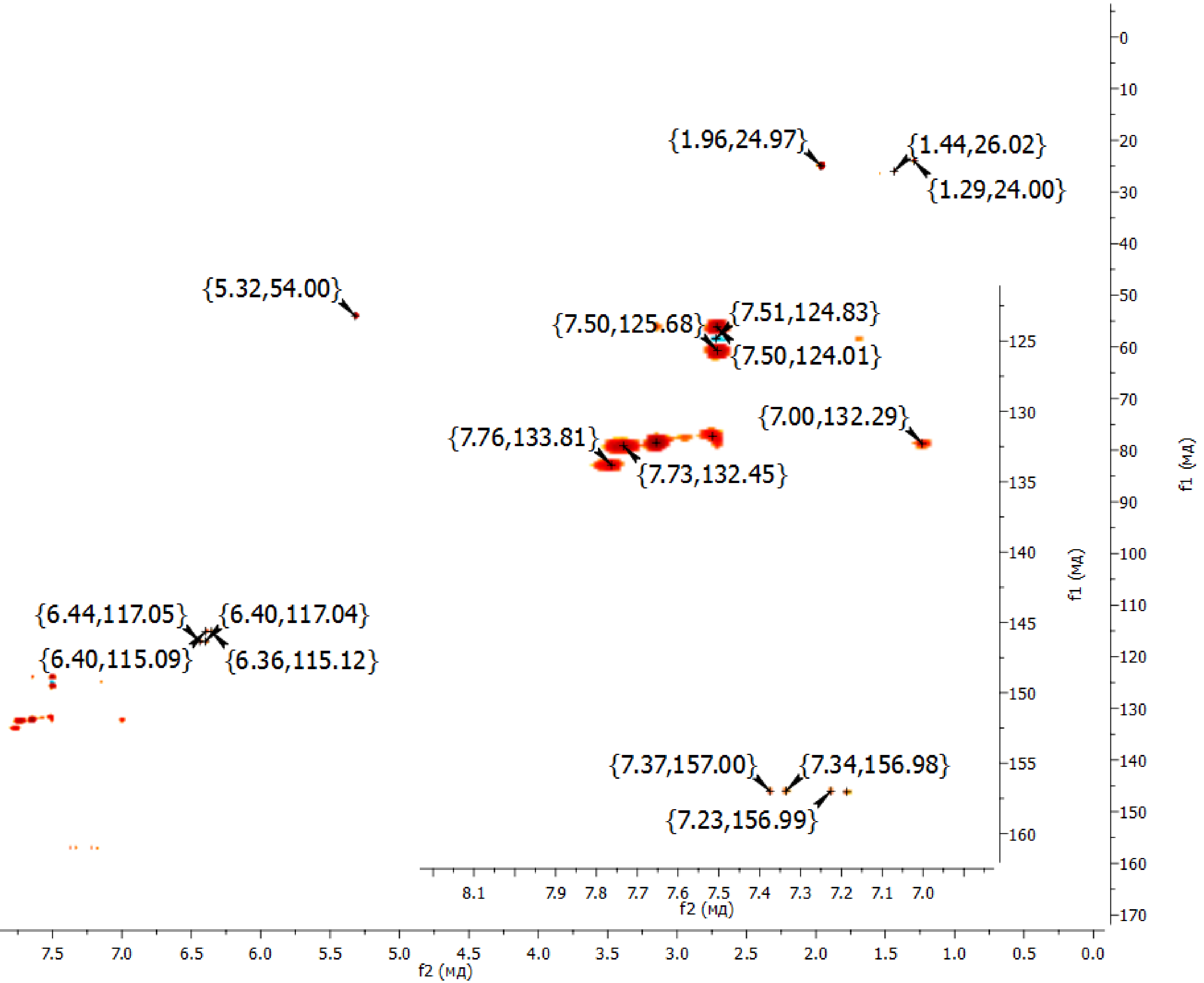

Figure S53. HSQC NMR spectrum of **1g** in TfOH after **6 days** (162 MHz, TfOH).

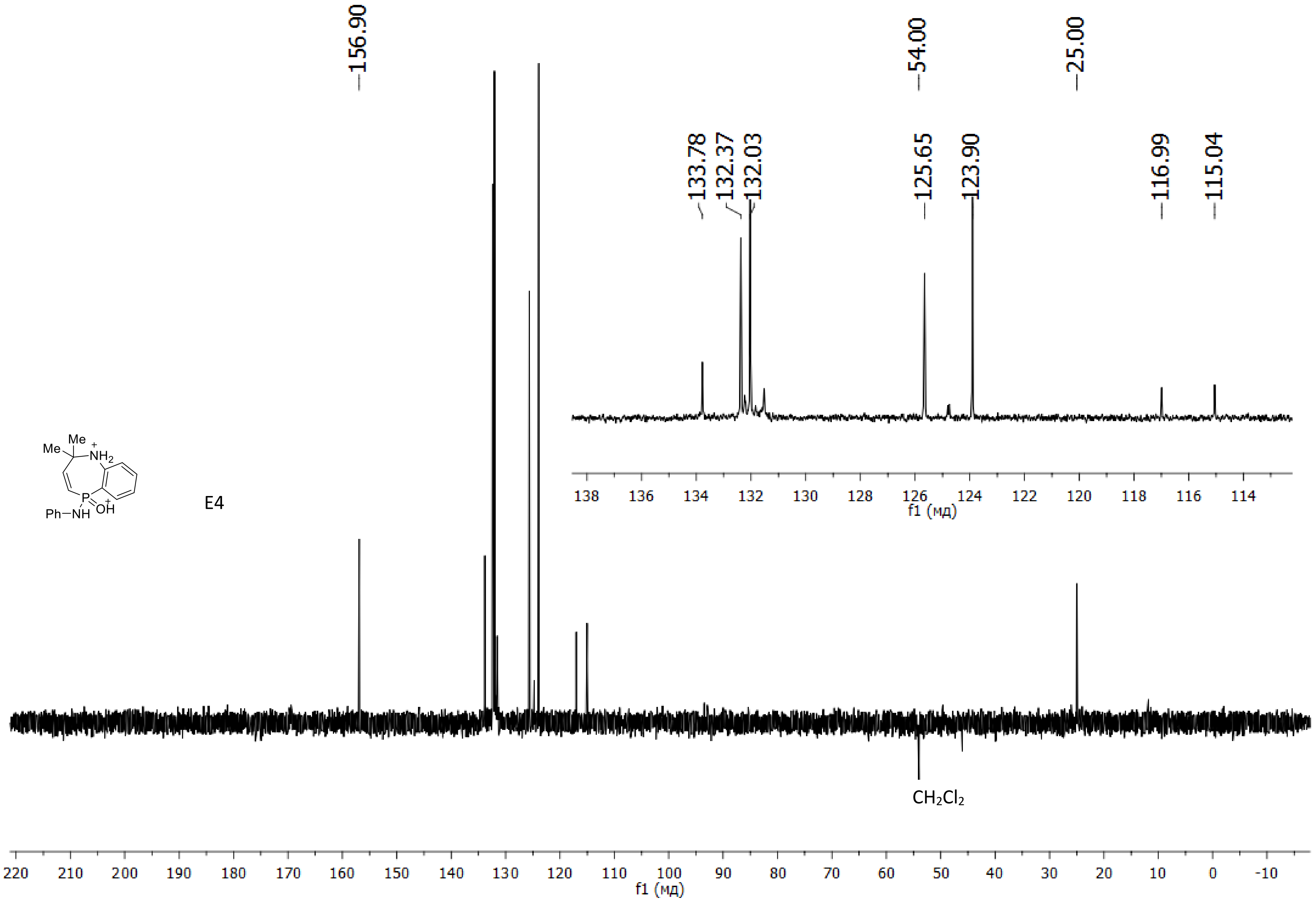

Figure S54. DEPT NMR spectrum of **1g** in TfOH after **6 days** (162 MHz, TFOH).

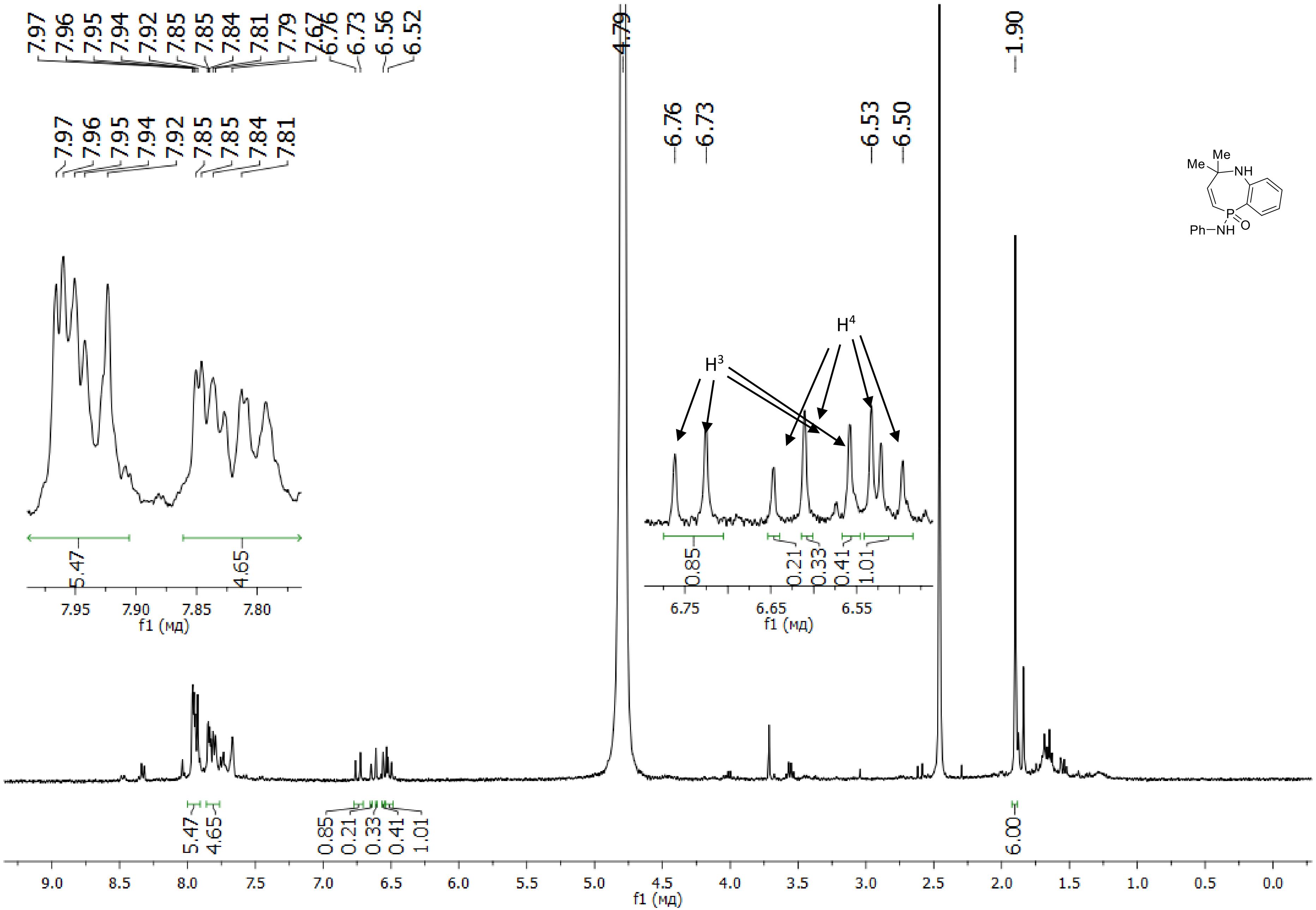

Figure S55. ^{1}H NMR spectrum of the compound **5** (400 MHz, D_2O).

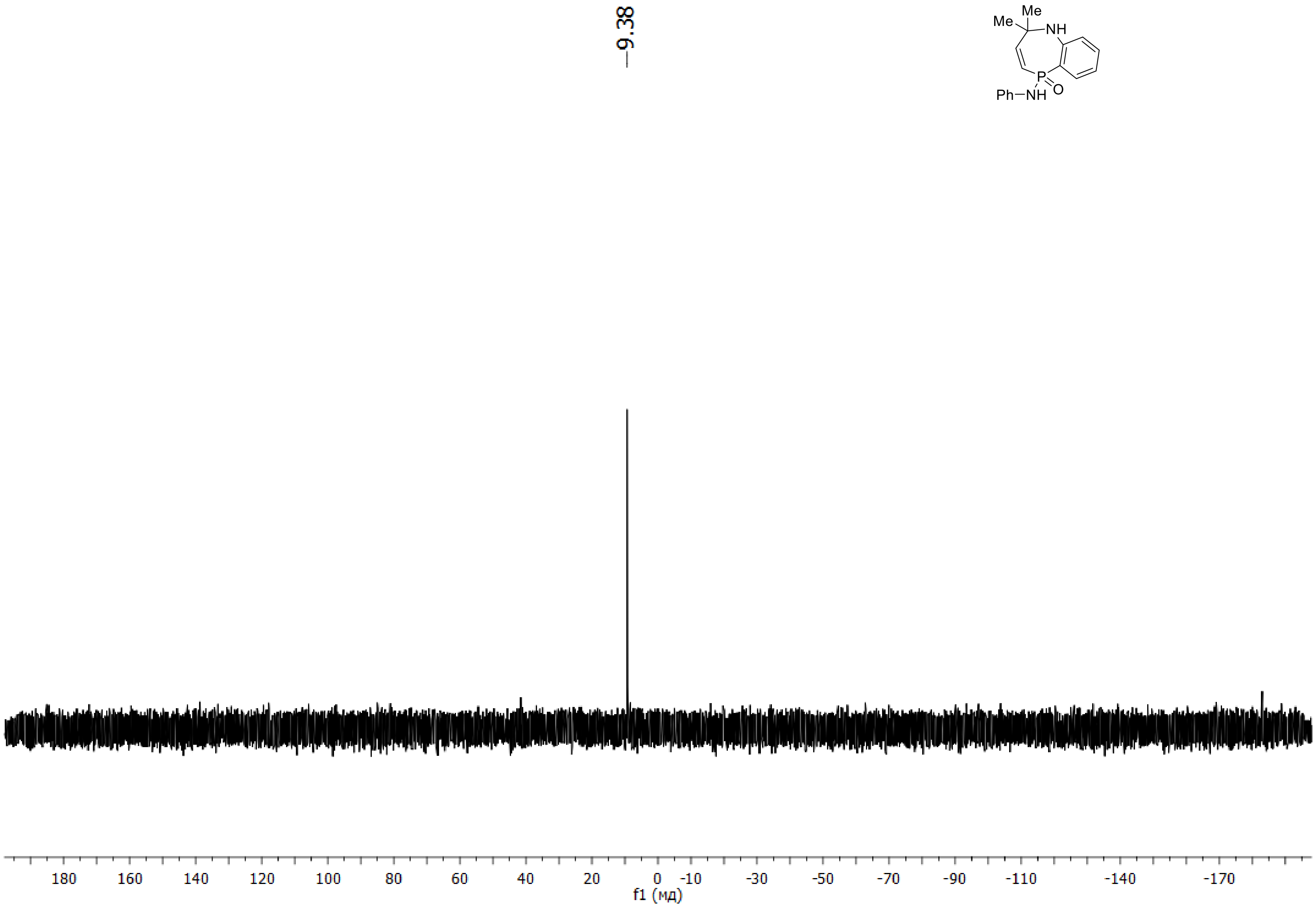

Figure S 56. ^{31}P NMR spectrum of the compound **5** (162 MHz, D_2O).

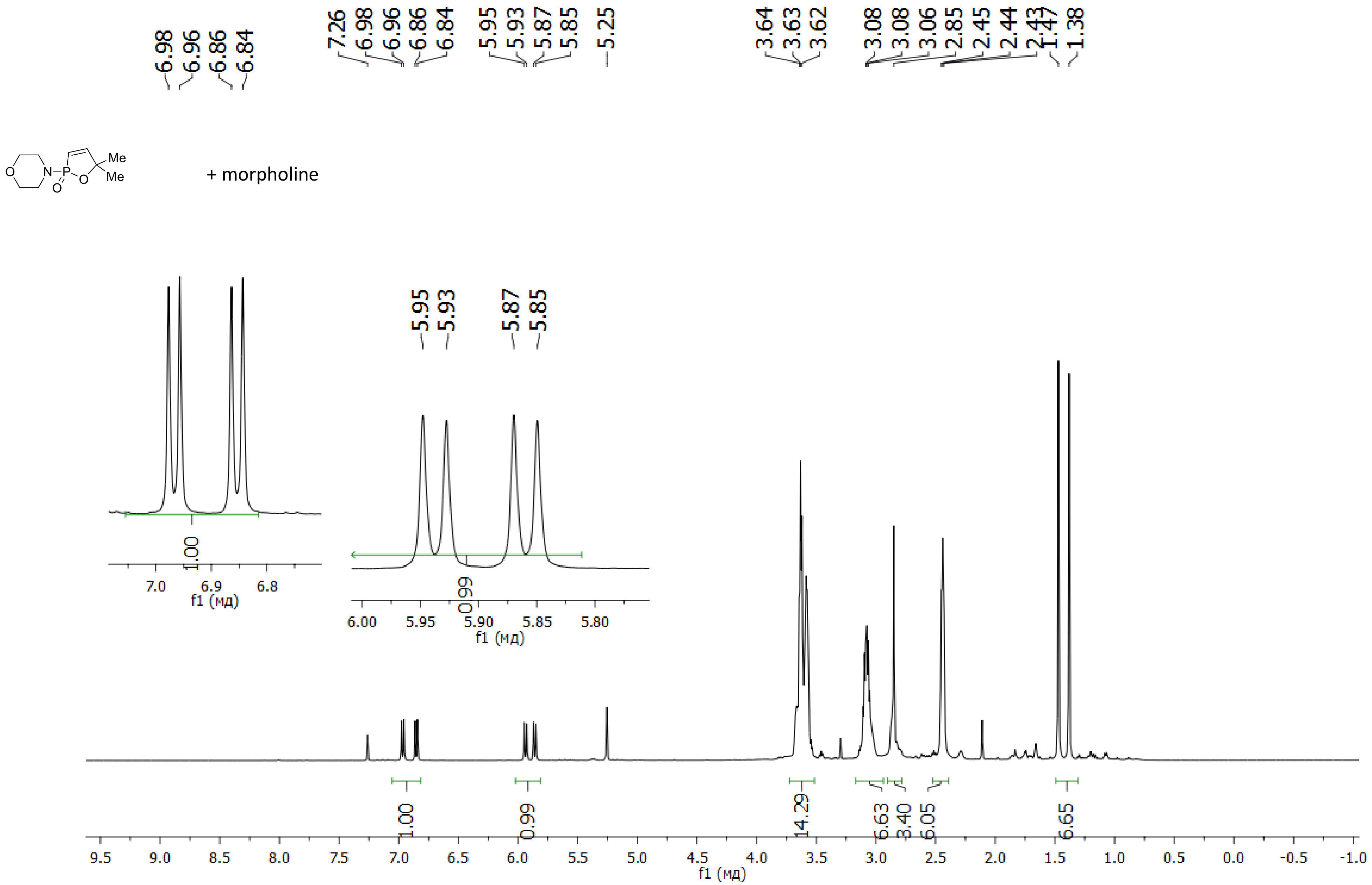

Figure S57. ^{1}H NMR spectrum of the compound **6a** (400 MHz, $CDCl_3$).

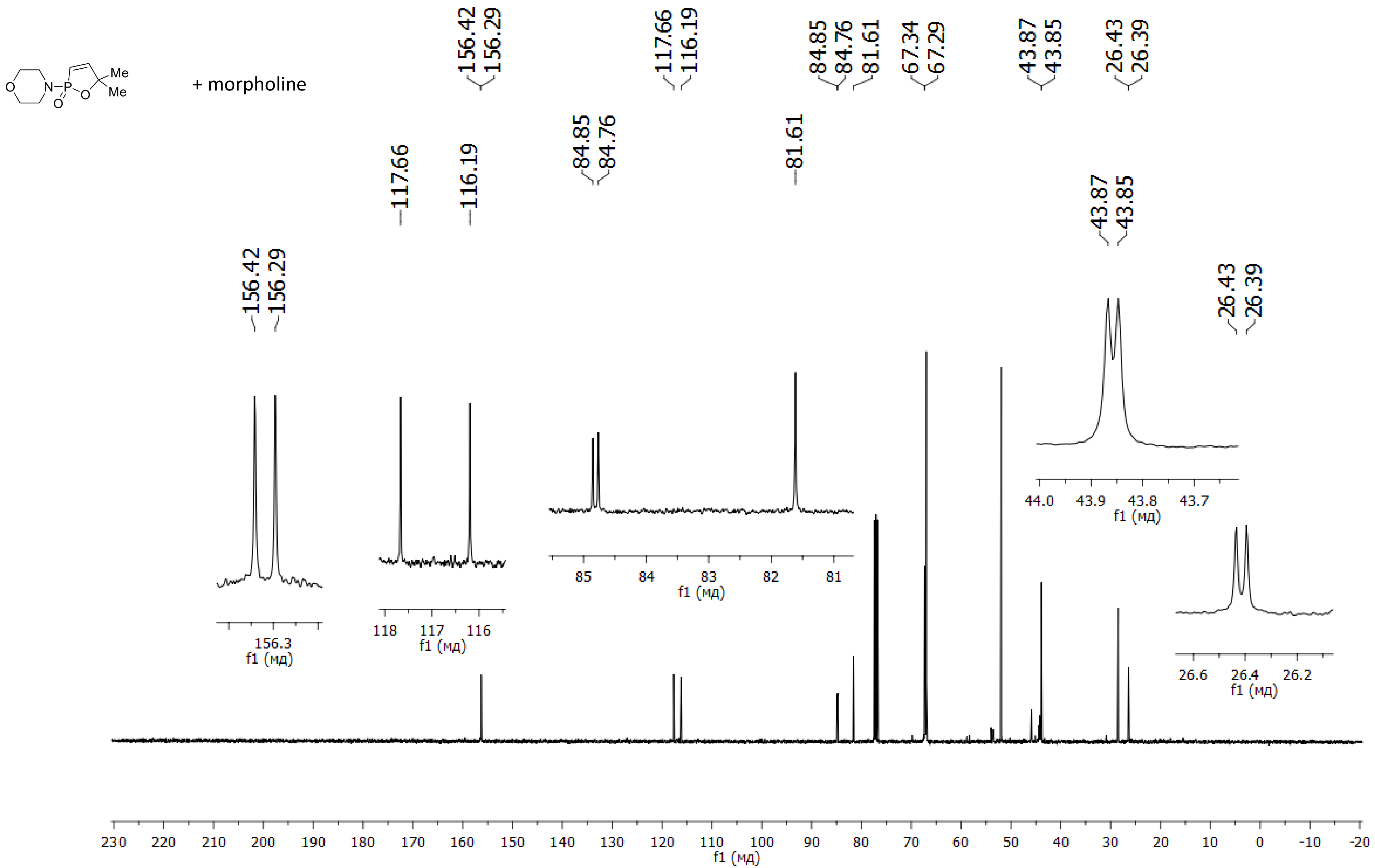

Figure S58. ^{13}C NMR spectrum of the compound **6a** (100 MHz, $CDCl_3$).

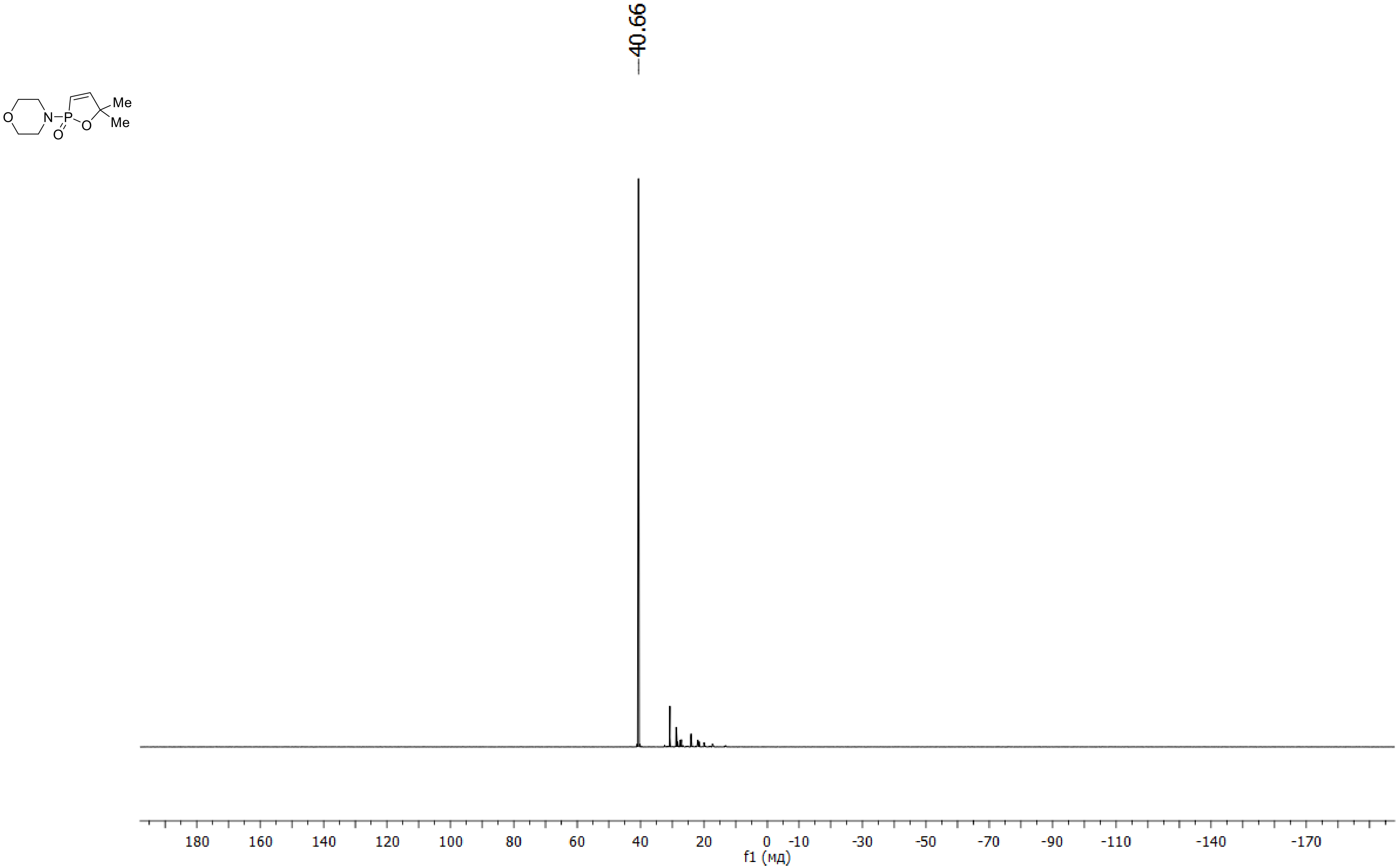

Figure S59. ^{31}P NMR spectrum of the compound **6a** (162 MHz, $CDCl_3$).

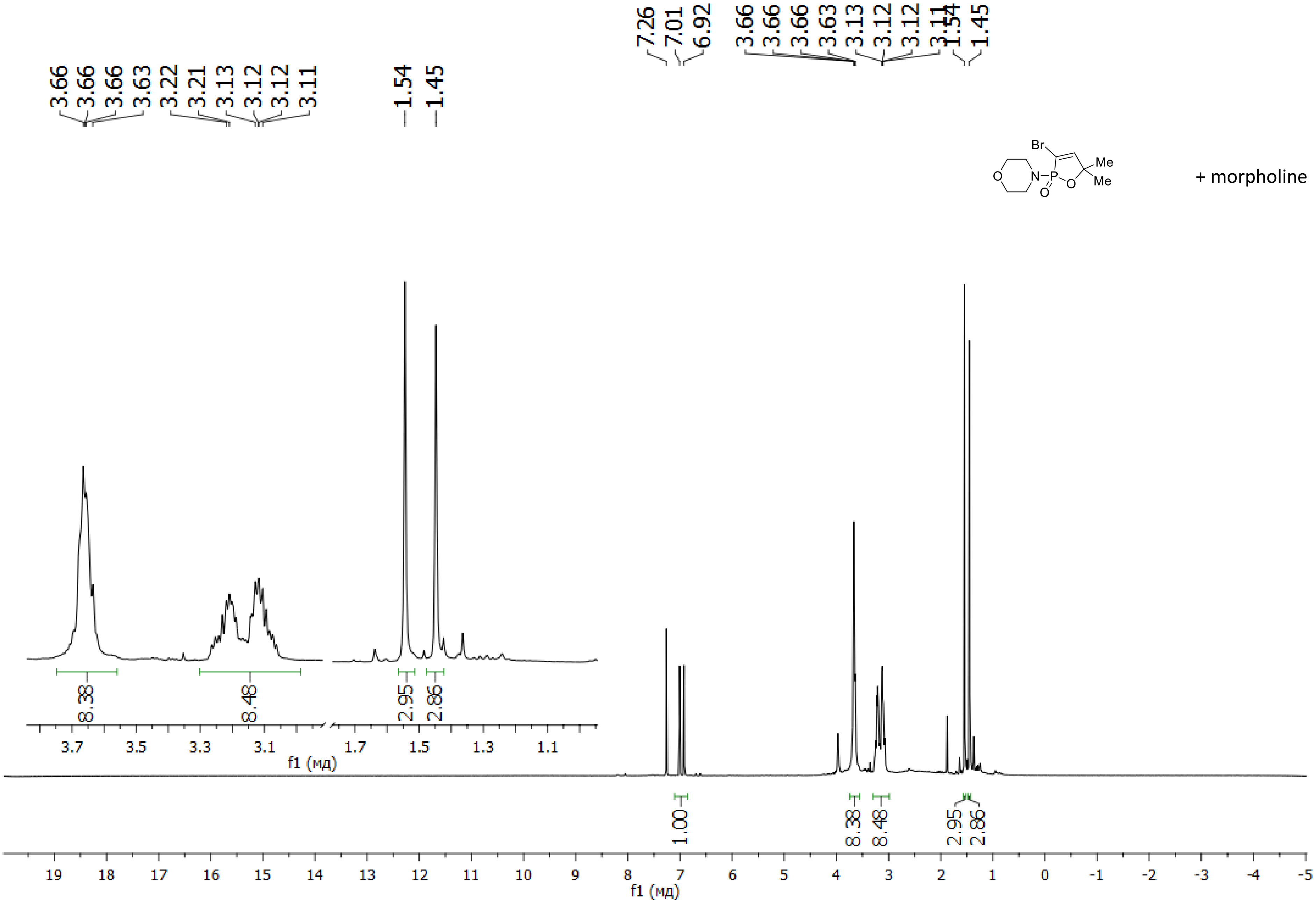

Figure S60. ^{1}H NMR spectrum of the compound **6b** (400 MHz, $CDCl_3$).

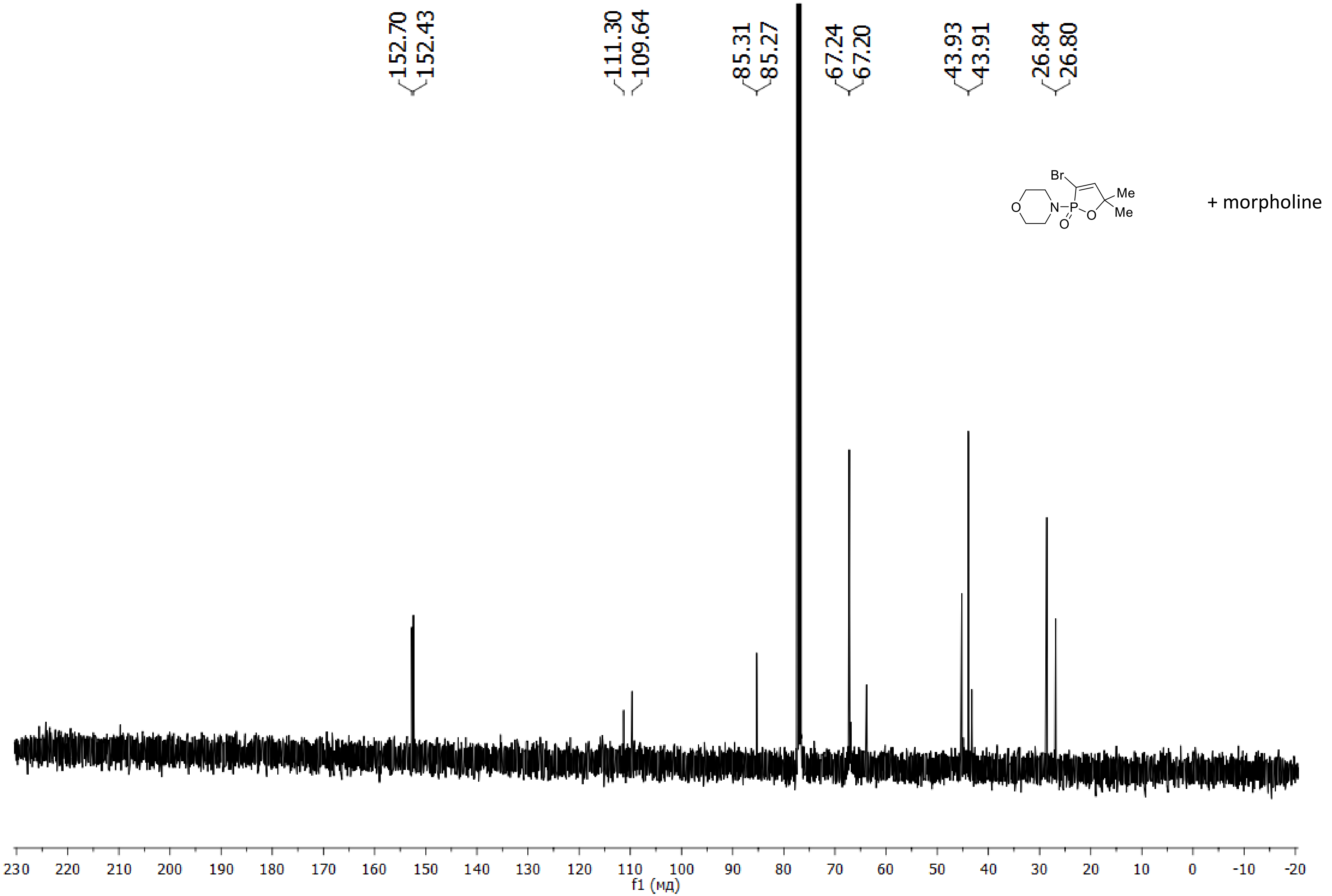

Figure S61. ^{13}C NMR spectrum of the compound **6b** (100 MHz, $CDCl_3$).

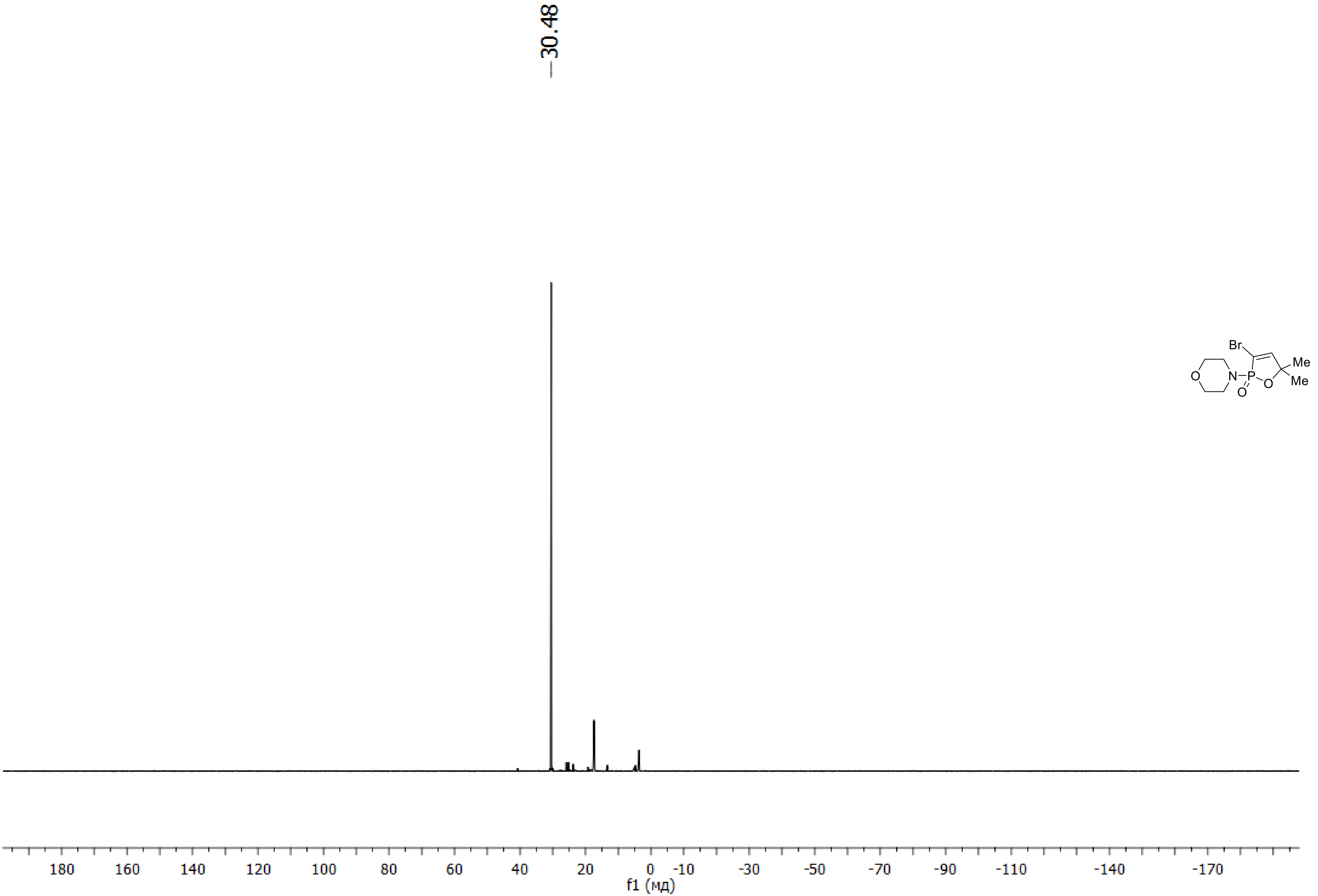

Figure S62. ^{31}P NMR spectrum of the compound **6b** (162 MHz, $CDCl_3$).

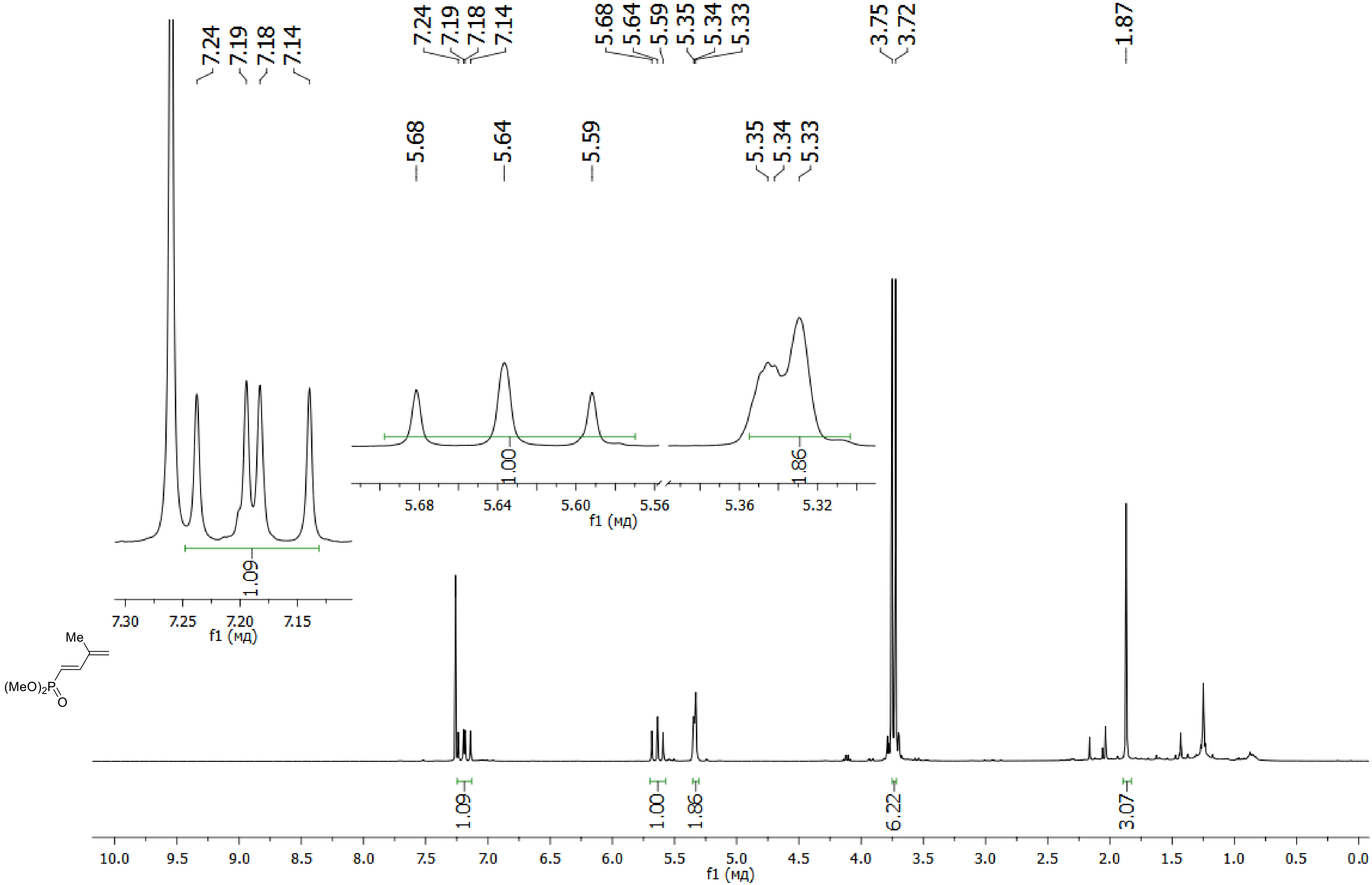

7.24
7.19
7.18
7.14
5.68
5.64
5.59
5.35
5.34
5.33
3.75
3.72
1.87
1.09
1.00
1.86
6.22
3.07
f1 (мд)
Me
(MeO)2P
O

Figure S63. ^{1}H NMR spectrum of the compound **10b** (400 MHz, $CDCl_3$).

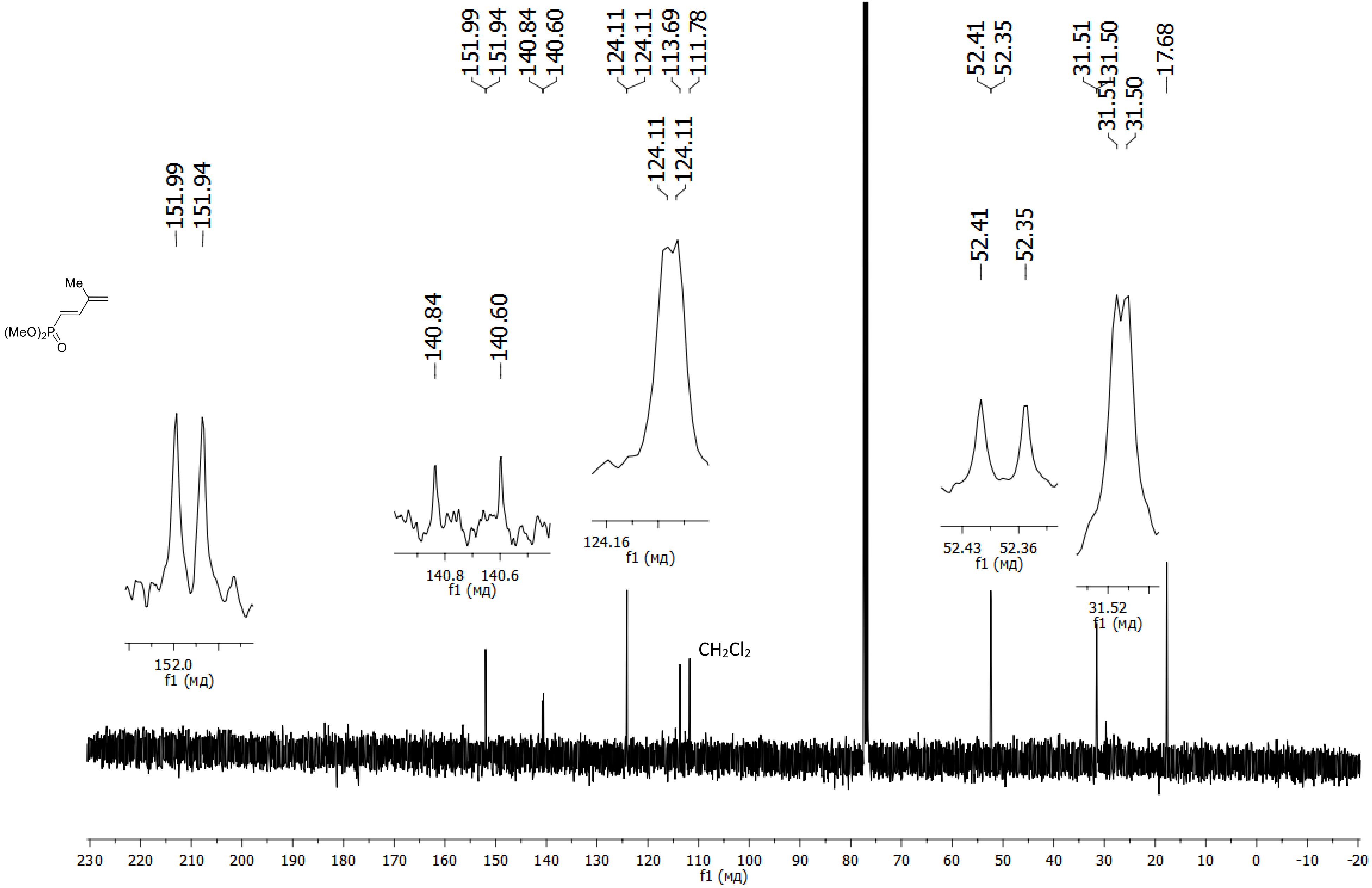

Figure S64. ^{13}C NMR spectrum of the compound **10b** (101 MHz, $CDCl_3$).

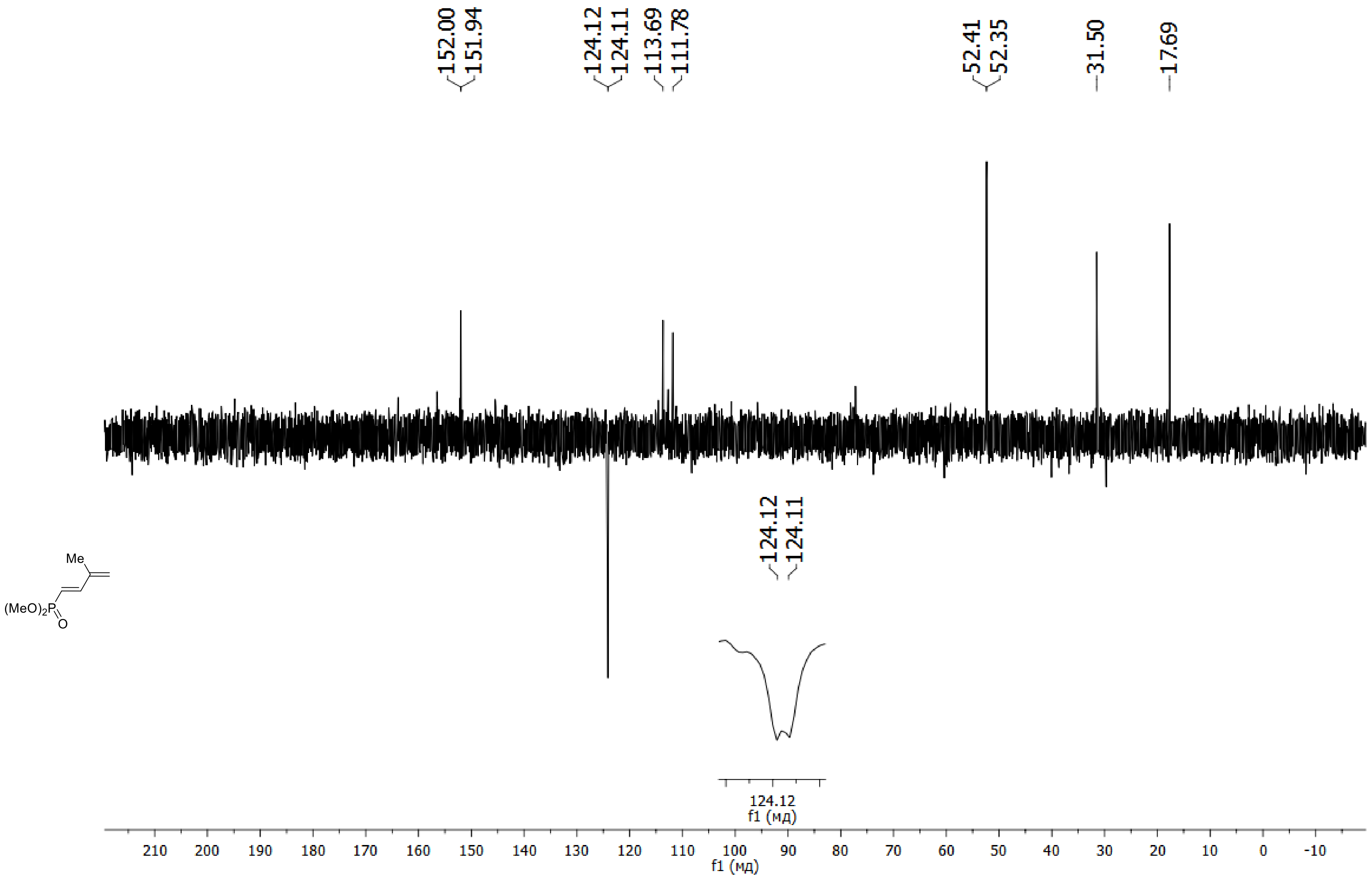

Figure S65. DEPT NMR spectrum of the compound **10b** (101 MHz, $CDCl_3$).

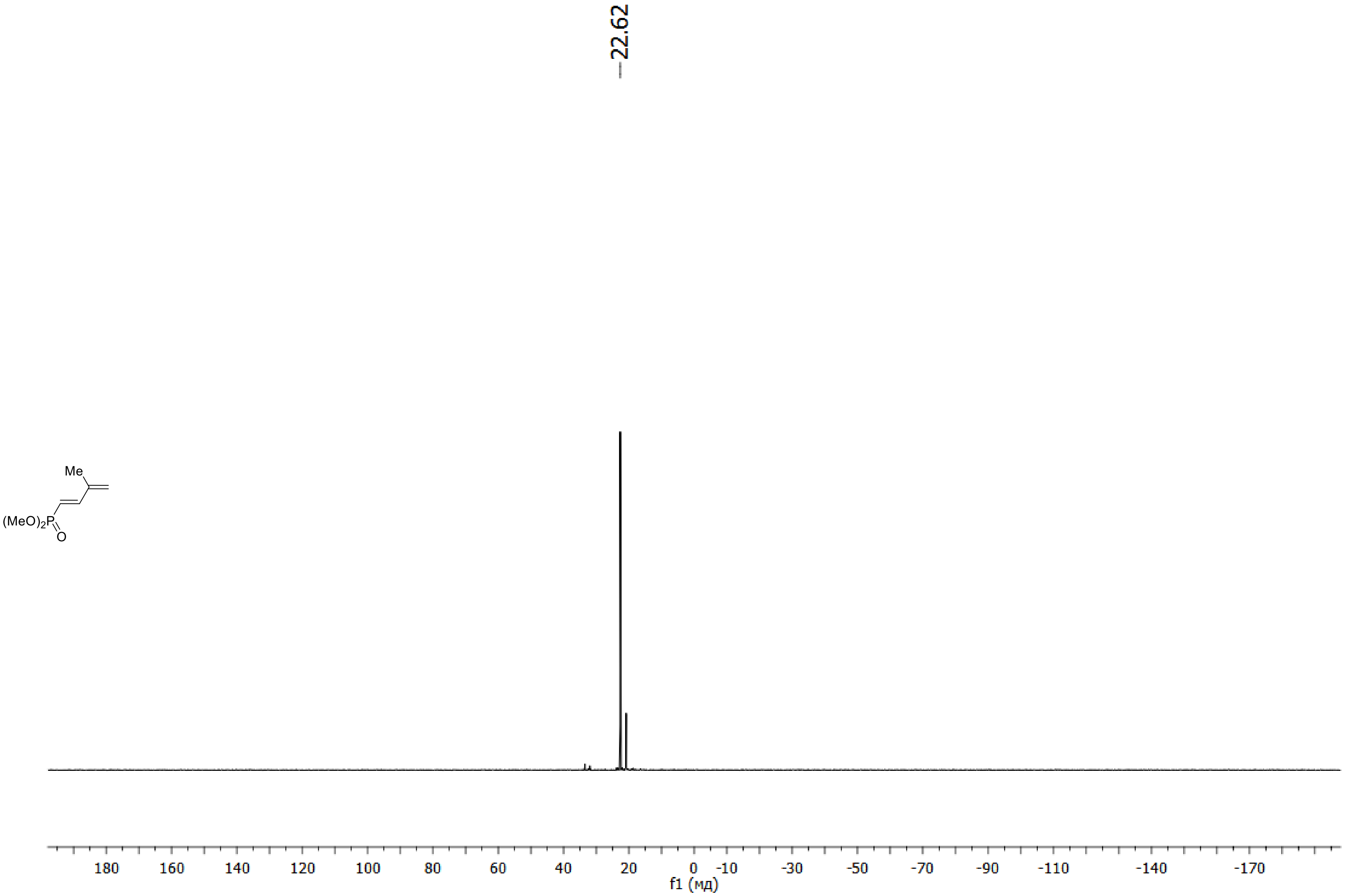

Figure S66. ^{31}P NMR spectrum of the compound **10b** (162 MHz, $CDCl_3$).

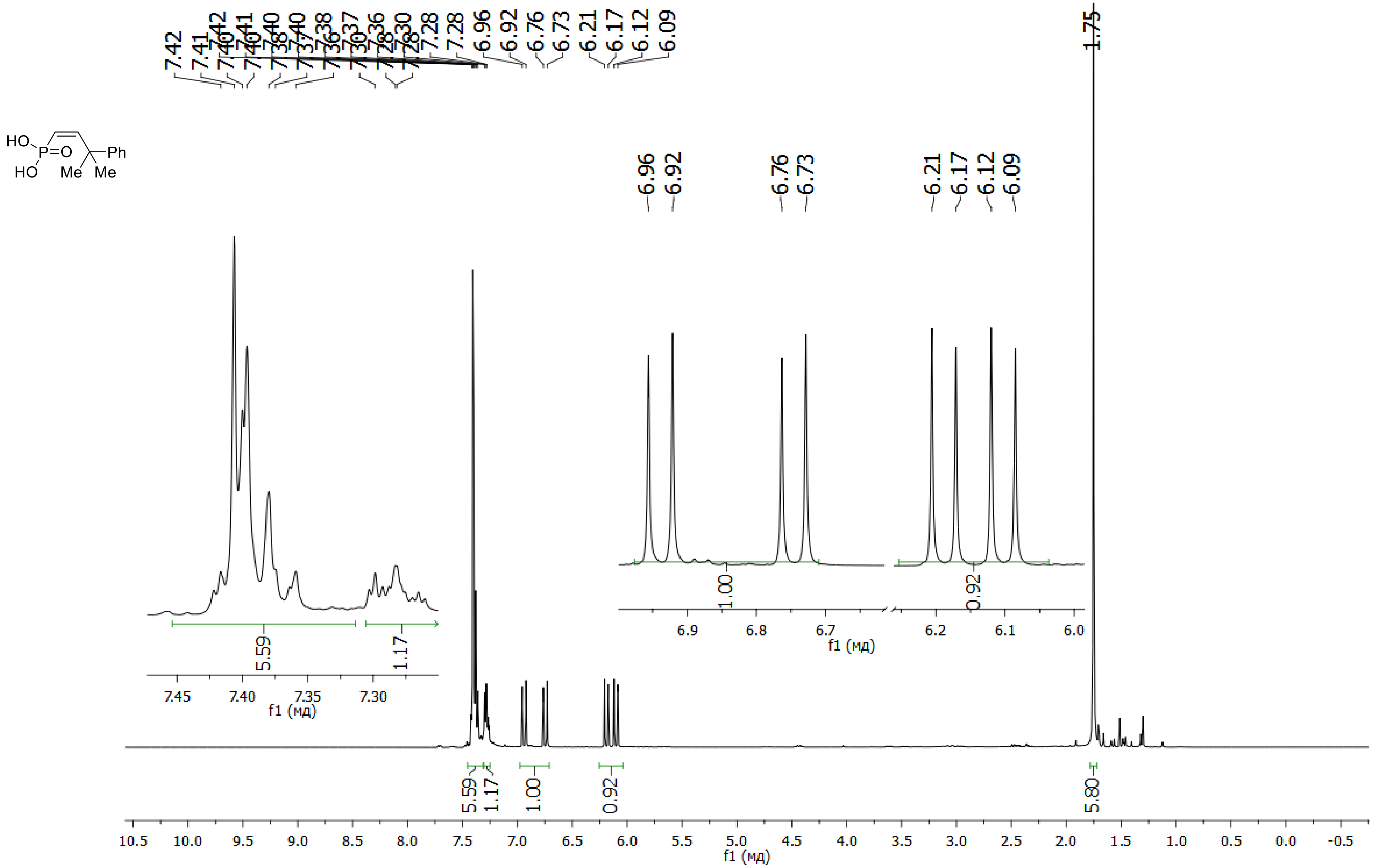

Figure S67. ^{1}H NMR spectrum of the compound **11a** (400 MHz, $CDCl_3$).

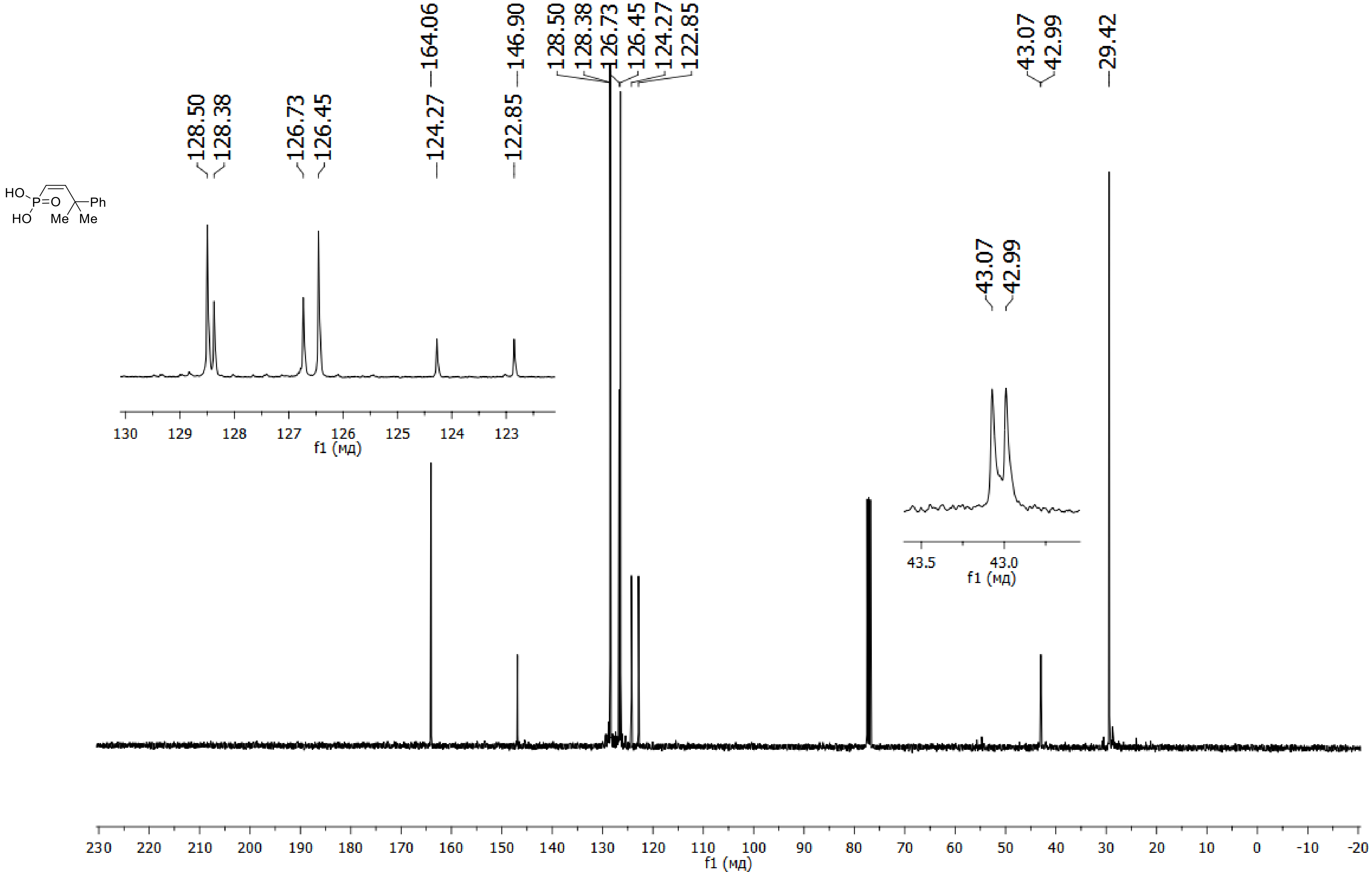

Figure S68. ^{13}C NMR spectrum of the compound **11a** (100 MHz, $CDCl_3$).

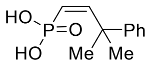

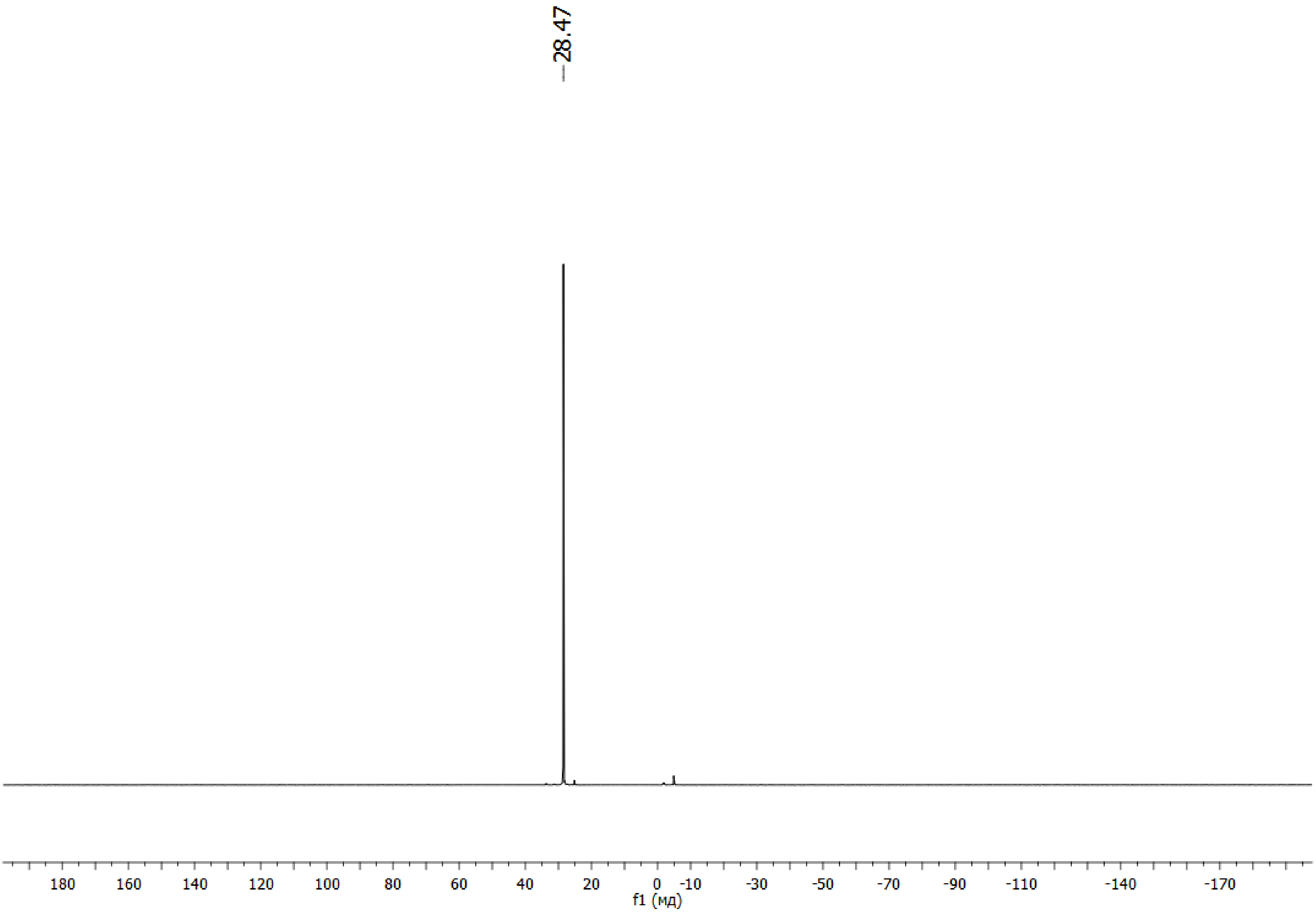

Figure S69. ^{31}P NMR spectrum of the compound **11a** (162 MHz, $CDCl_3$).

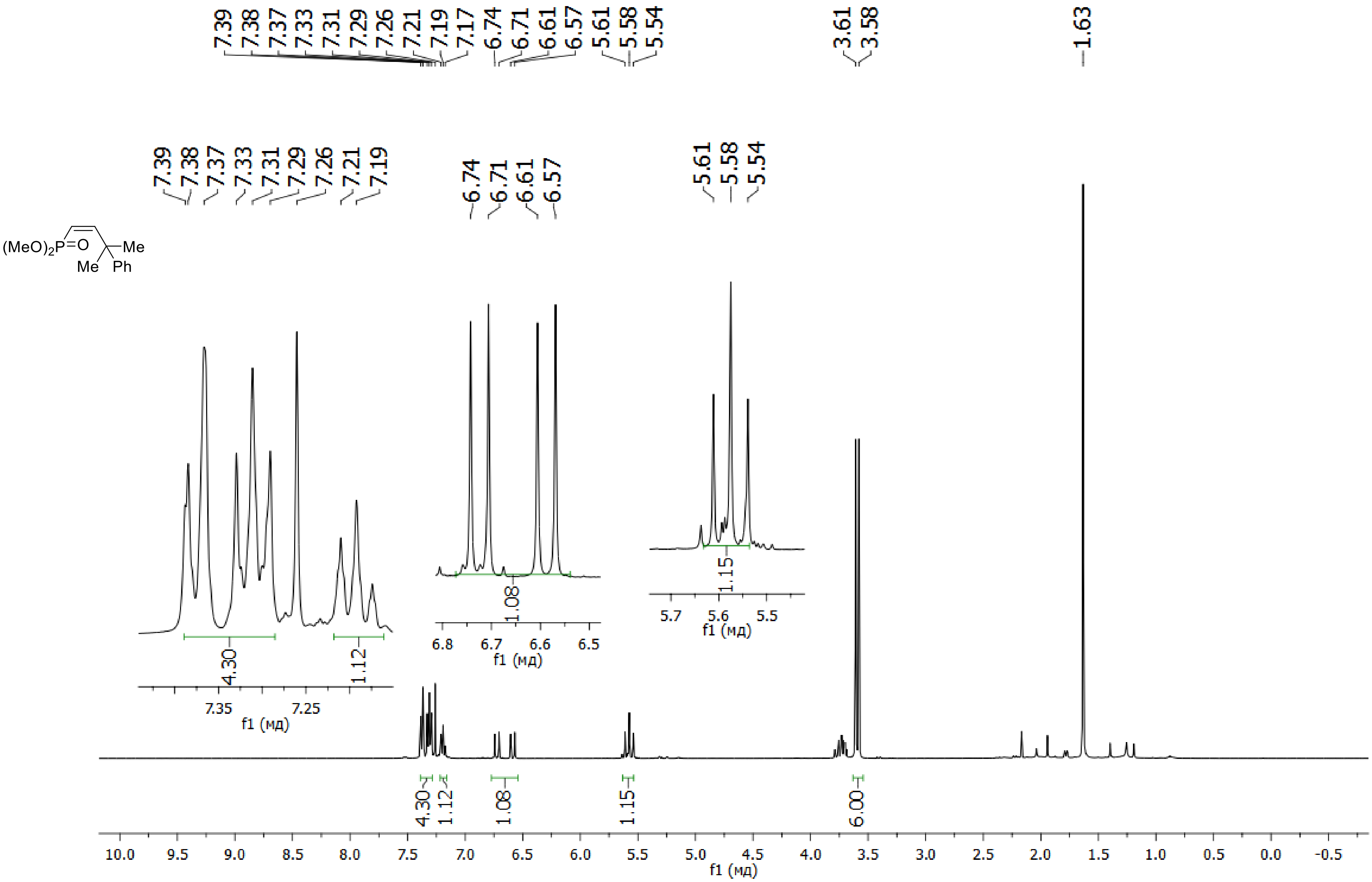

Figure S70. ^{1}H NMR spectrum of the compound **11b** (400 MHz, $CDCl_3$).

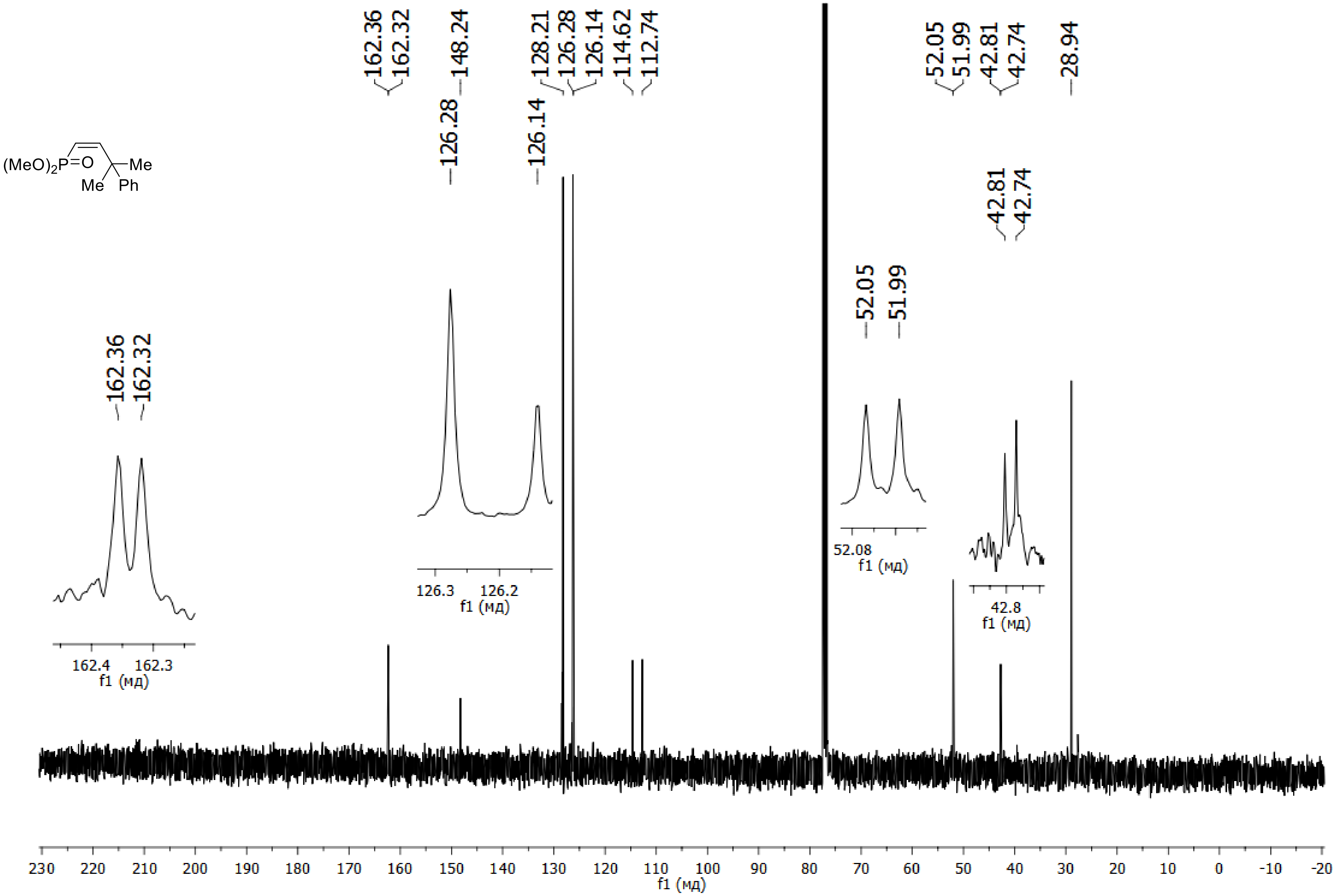

Figure S71. ^{13}C NMR spectrum of the compound **11b** (100 MHz, $CDCl_3$).

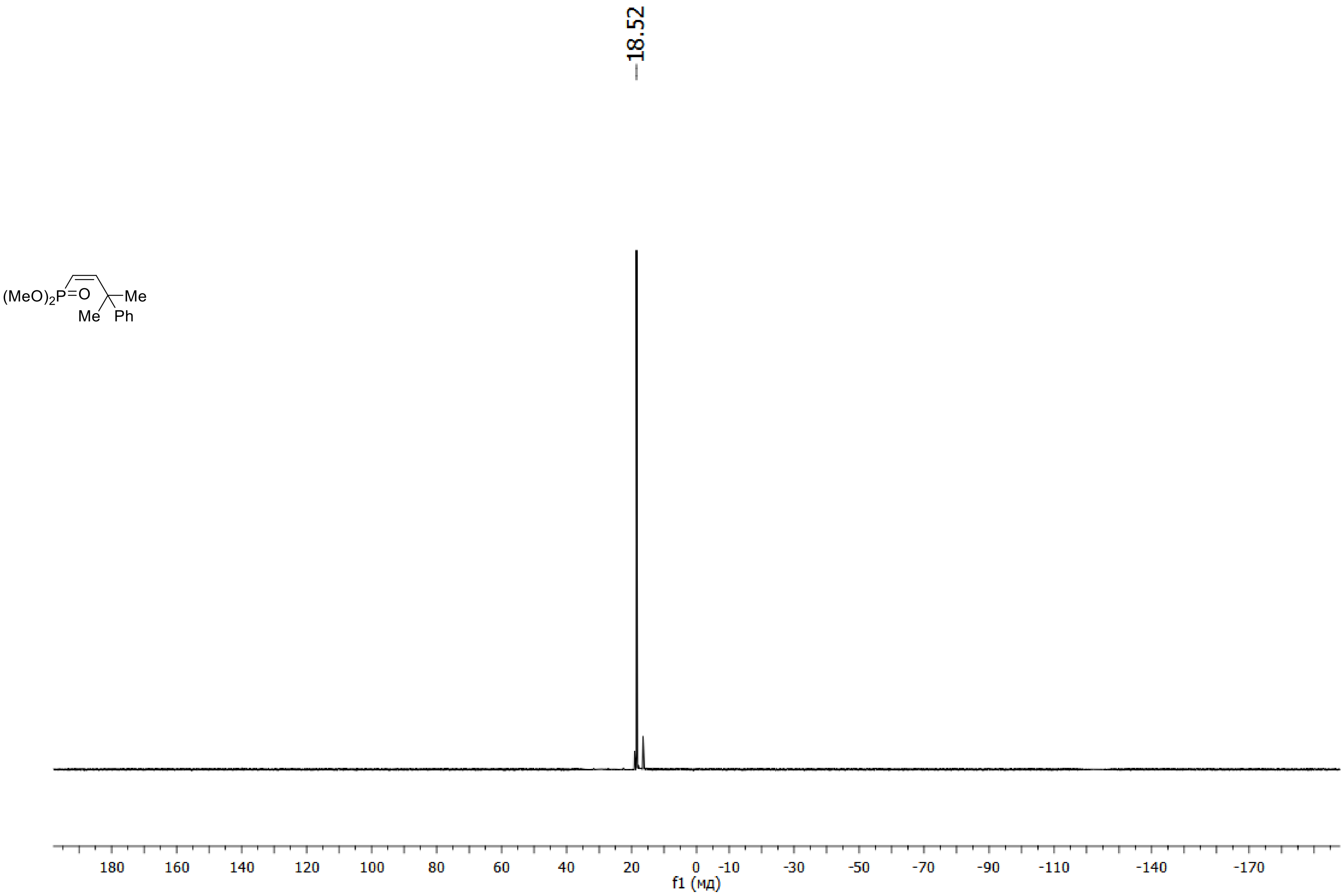

Figure S72. ^{31}P NMR spectrum of the compound **11b** (162 MHz, $CDCl_3$).

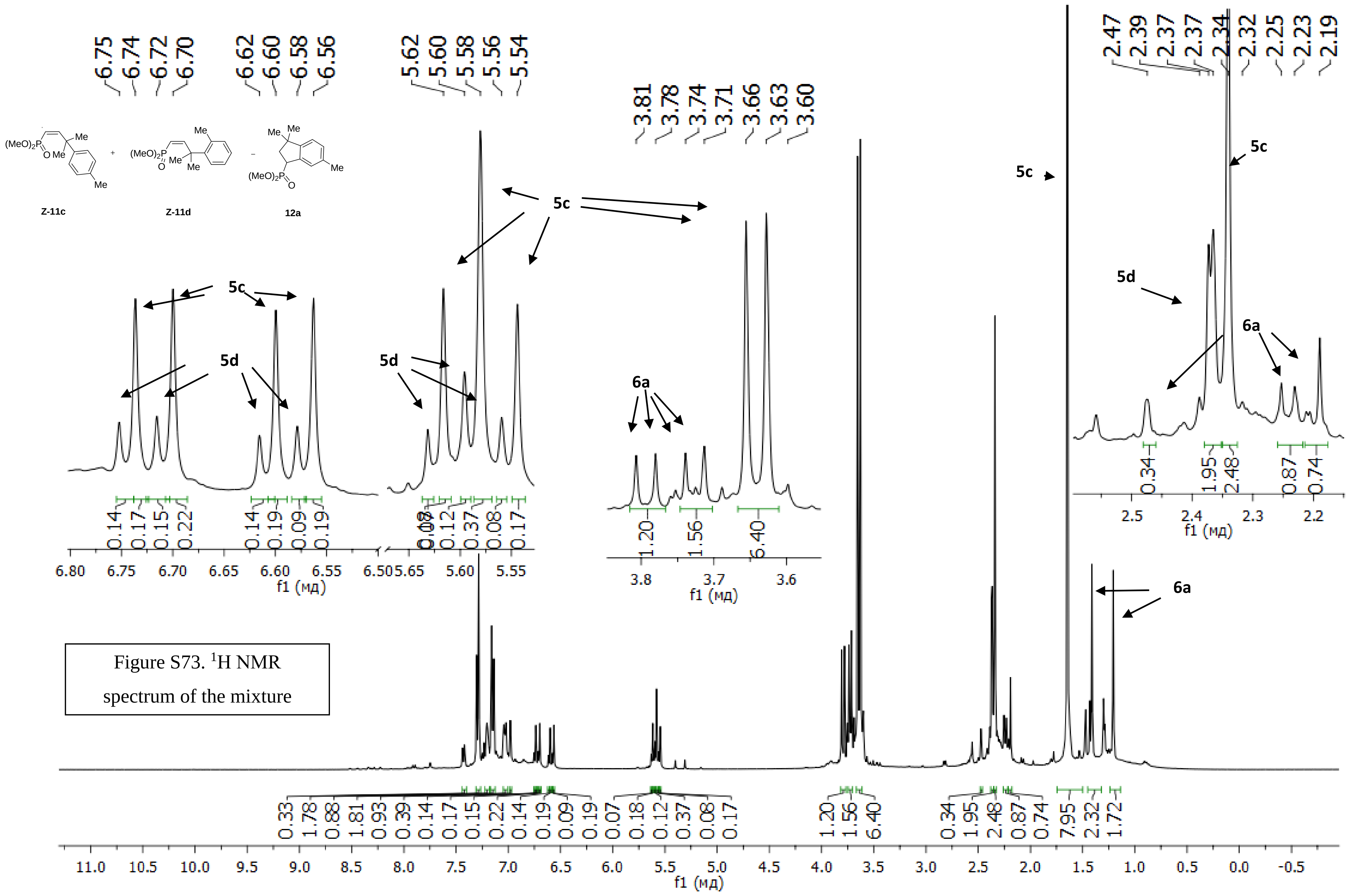

Figure S73. ^{1}H NMR spectrum of the mixture

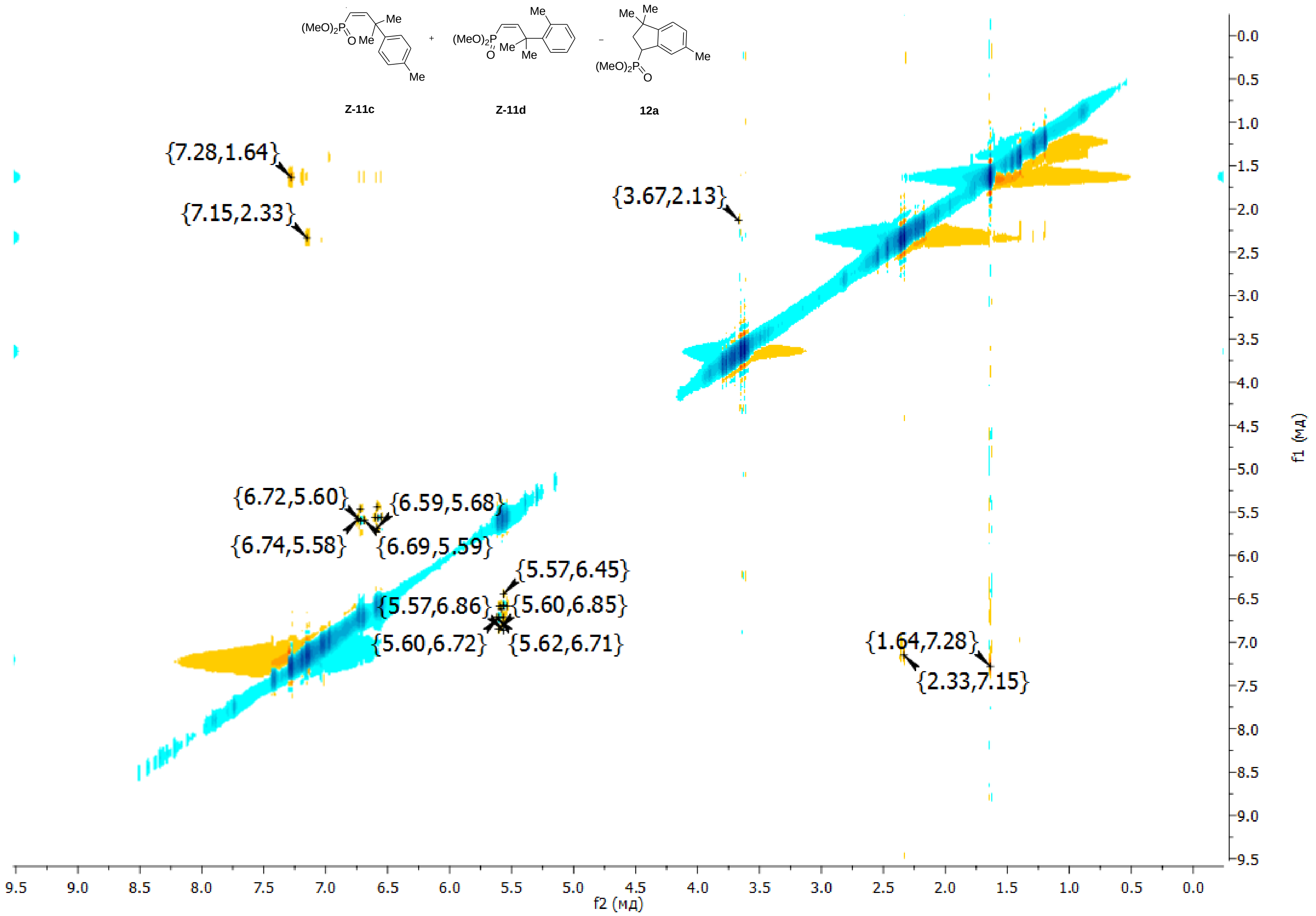

Figure S74. H-H COSY NMR spectrum of the mixture **11c**+**11d**+**12a** (400 MHz, $CDCl_3$).

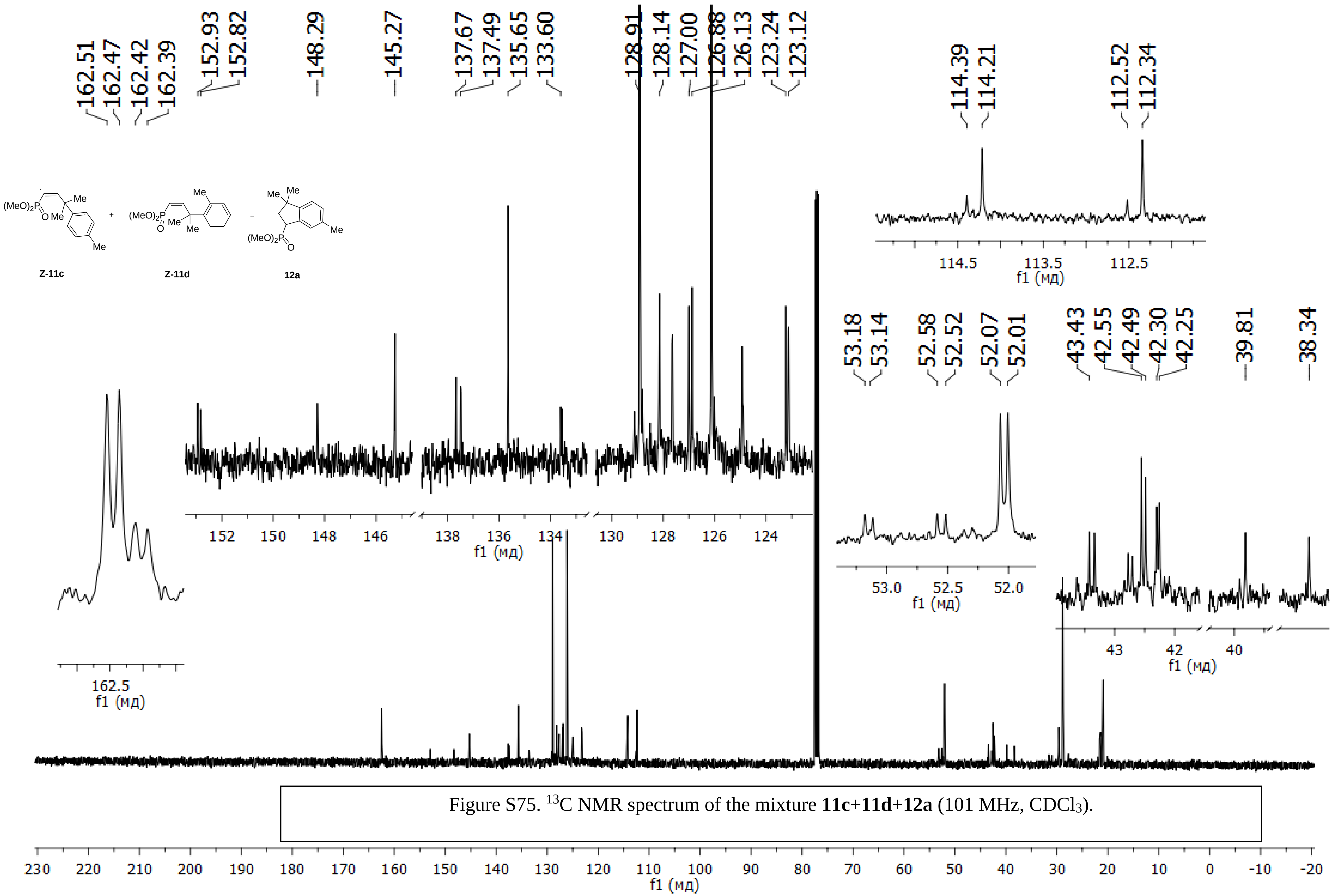

Figure S75. ^{13}C NMR spectrum of the mixture **11c**+**11d**+**12a** (101 MHz, $CDCl_3$).

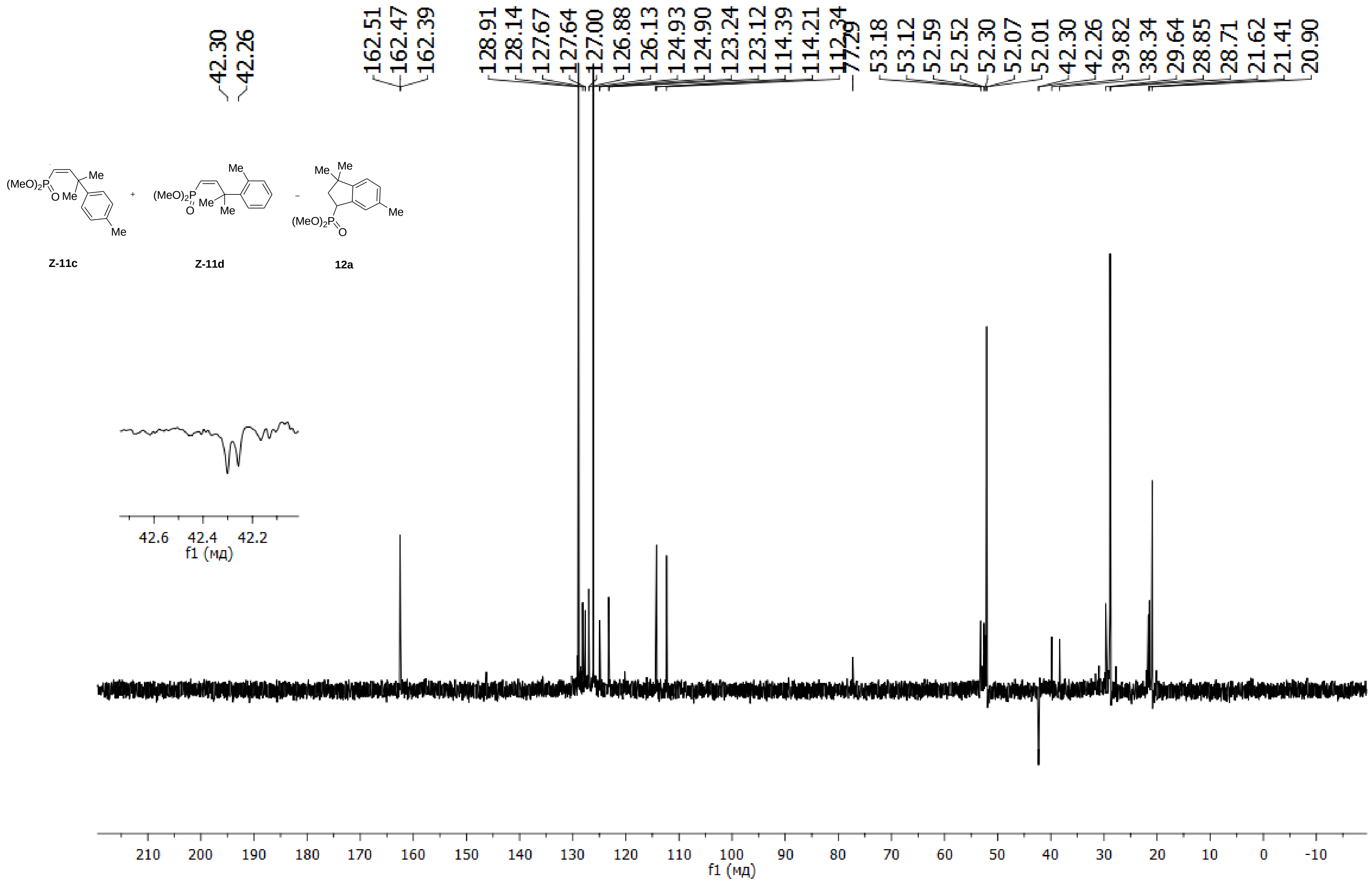

Figure S76. DEPT NMR spectrum of the mixture **11c**+**11d**+**12a** (101 MHz, $CDCl_3$).

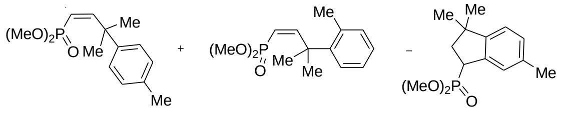

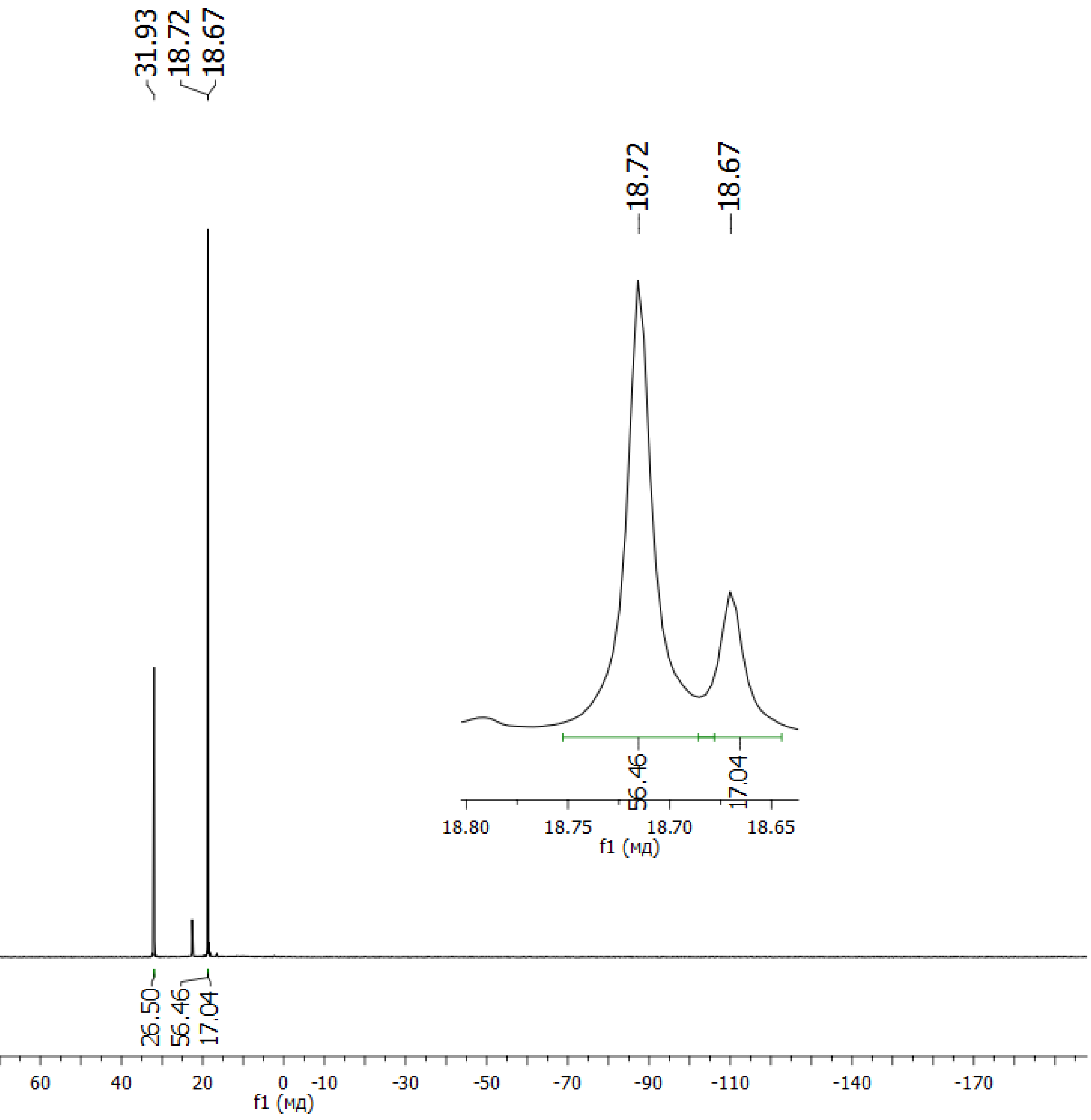

Figure S77. ^{31}P NMR spectrum of the mixture **11c**+**11d**+**12a** (162 MHz, $CDCl_3$).

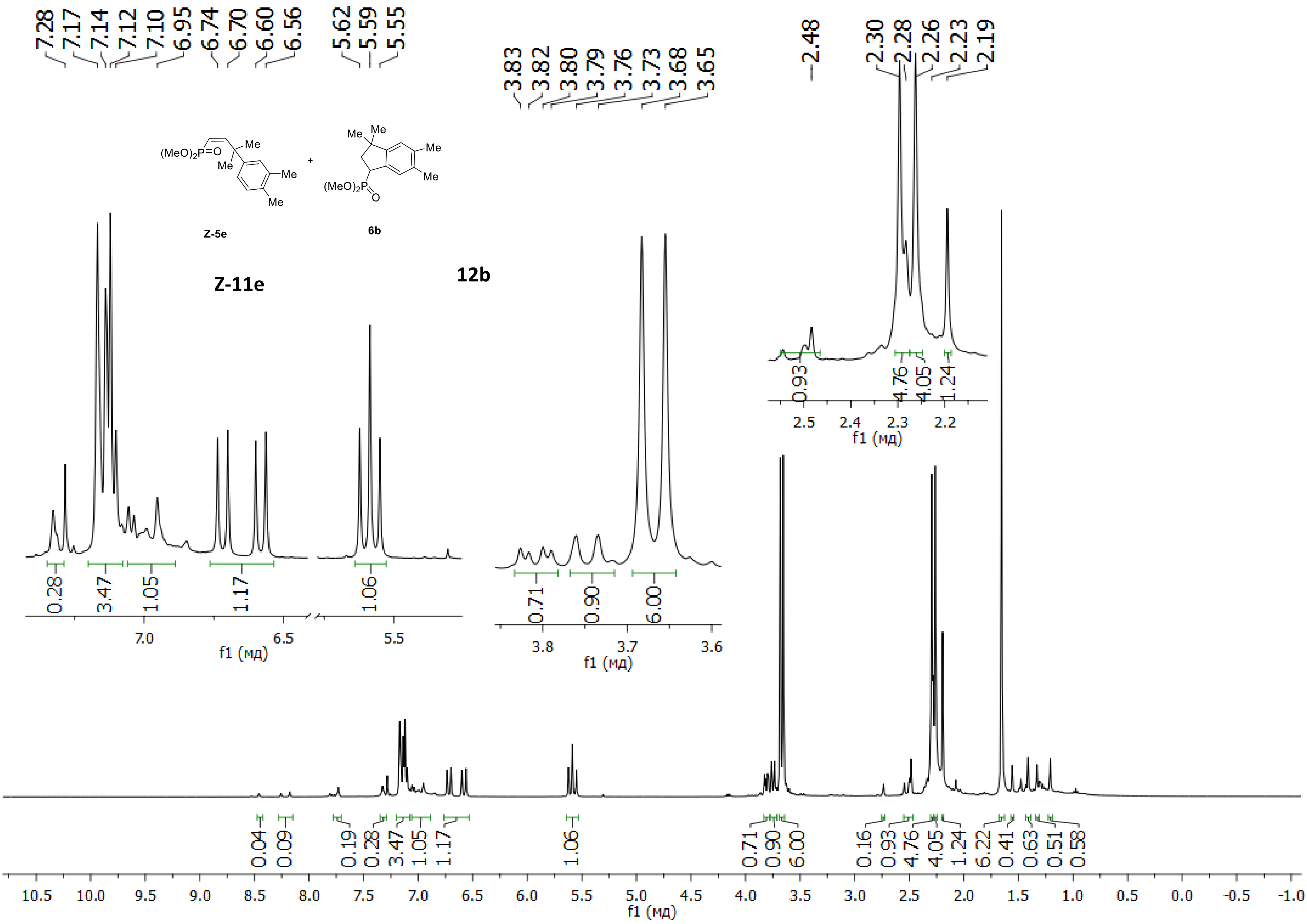

Figure S78. ^{1}H NMR spectrum of the mixture **11e**+**12b** (400 MHz, $CDCl_3$).

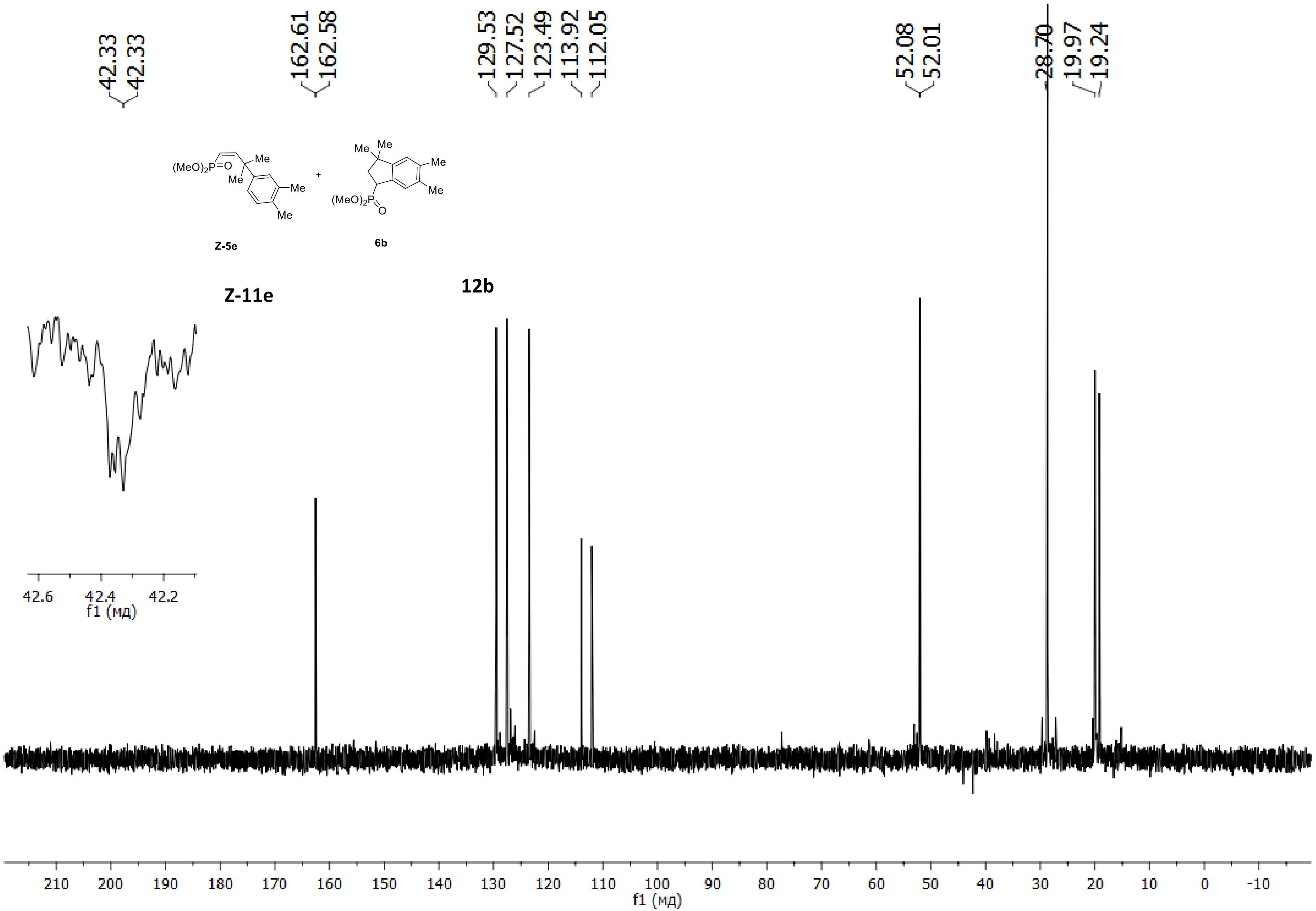

Figure S79. DEPT NMR spectrum of the mixture **11e**+**12b** (101 MHz, $CDCl_3$).

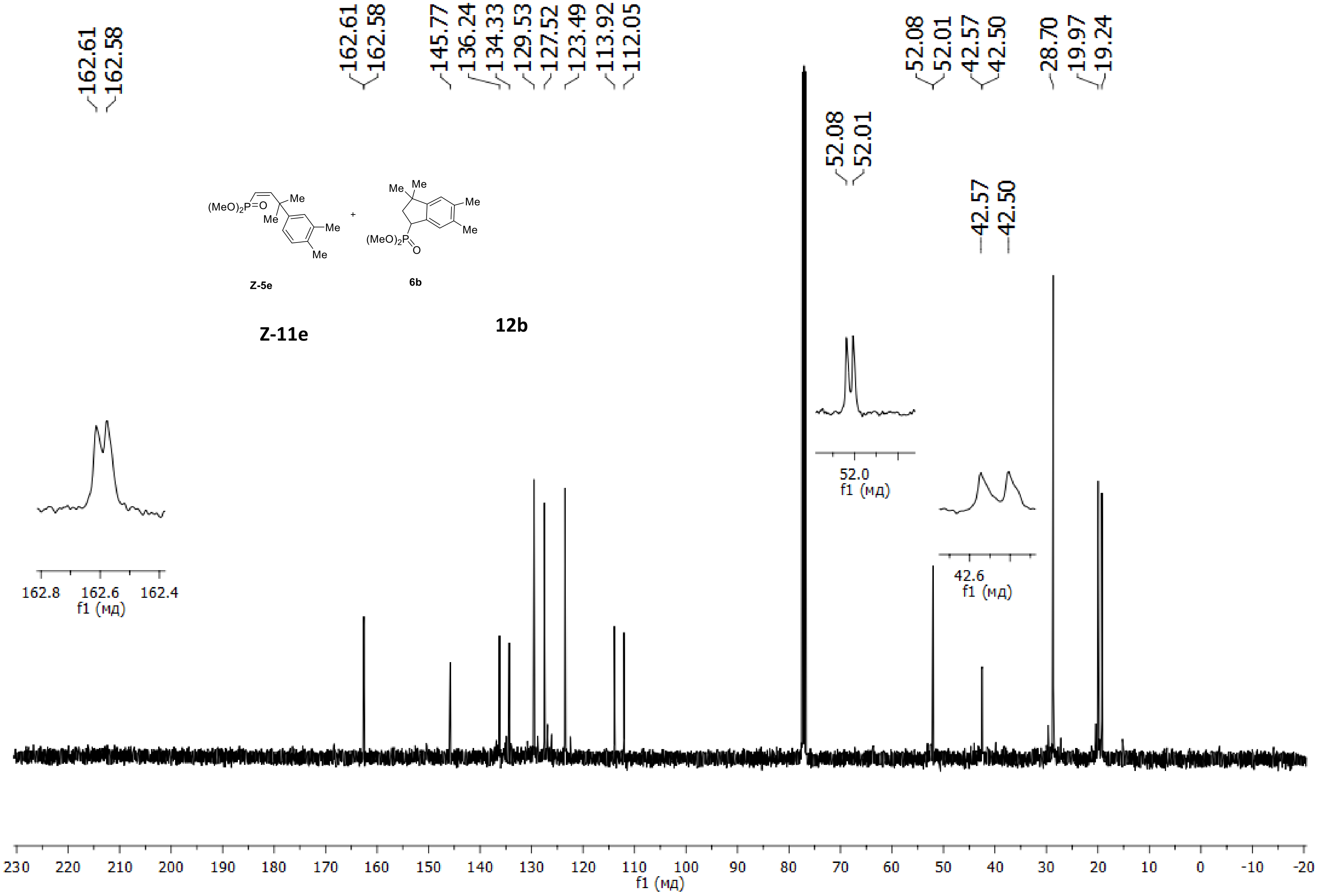

Figure S80. ^{13}C NMR spectrum of the mixture **11e**+**12b** (101 MHz, $CDCl_3$).

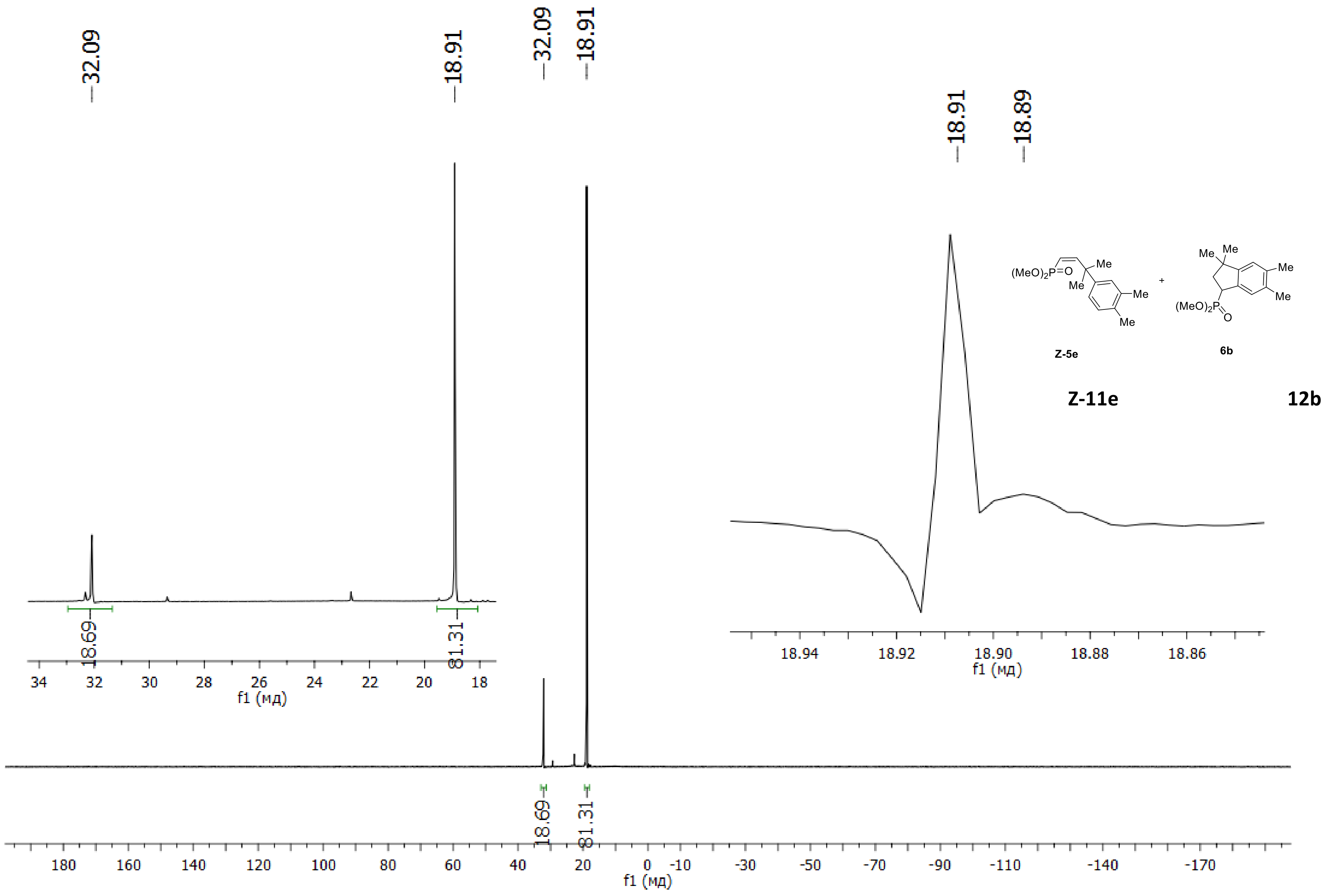

Figure S81. ^{31}P NMR spectrum of the mixture **11e**+**12b** (162 MHz, CDCl$_3$).

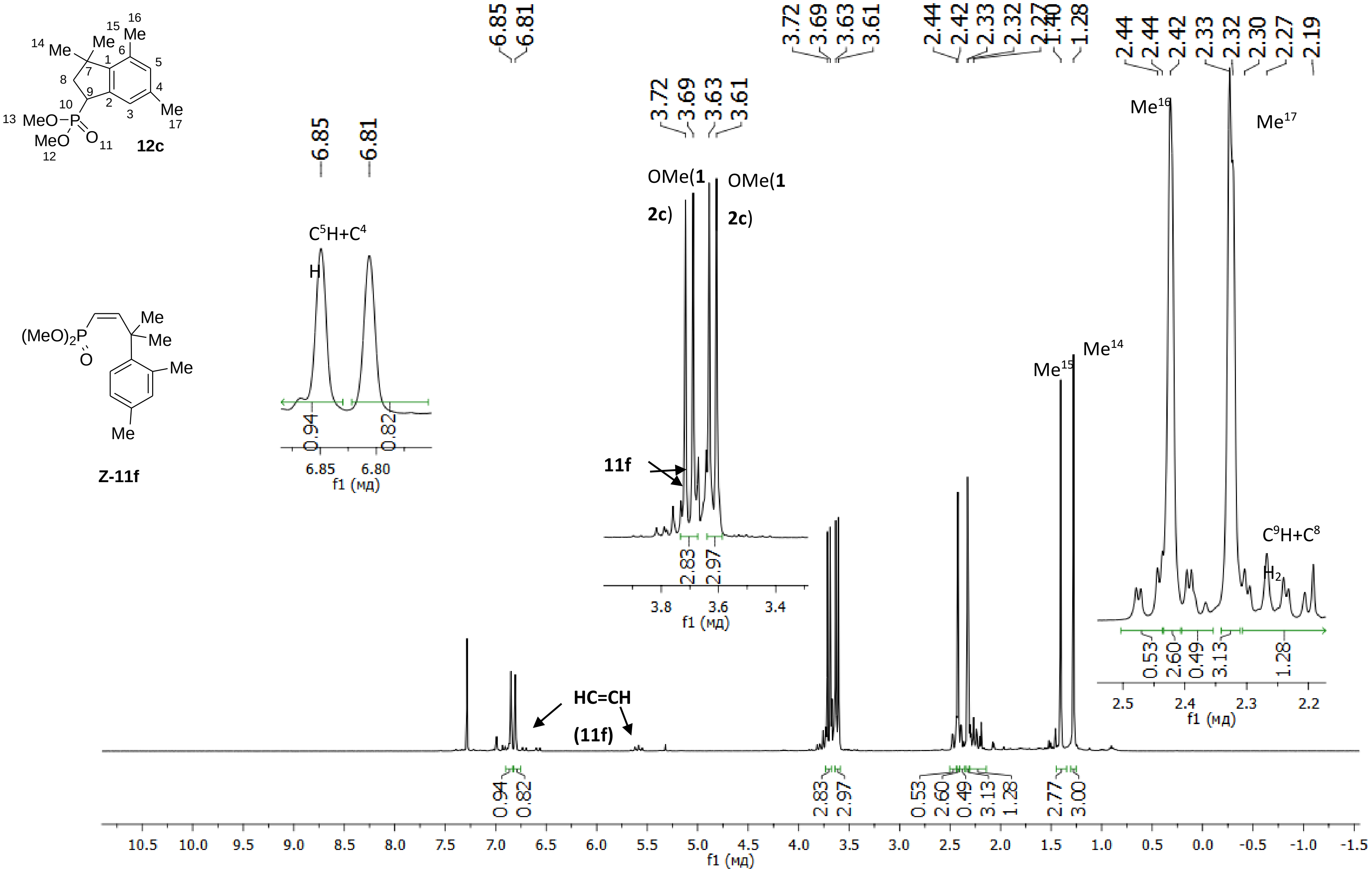

Figure S82. ^{1}H NMR spectrum of the mixture **12c**+**11f** (400 MHz, $CDCl_3$).

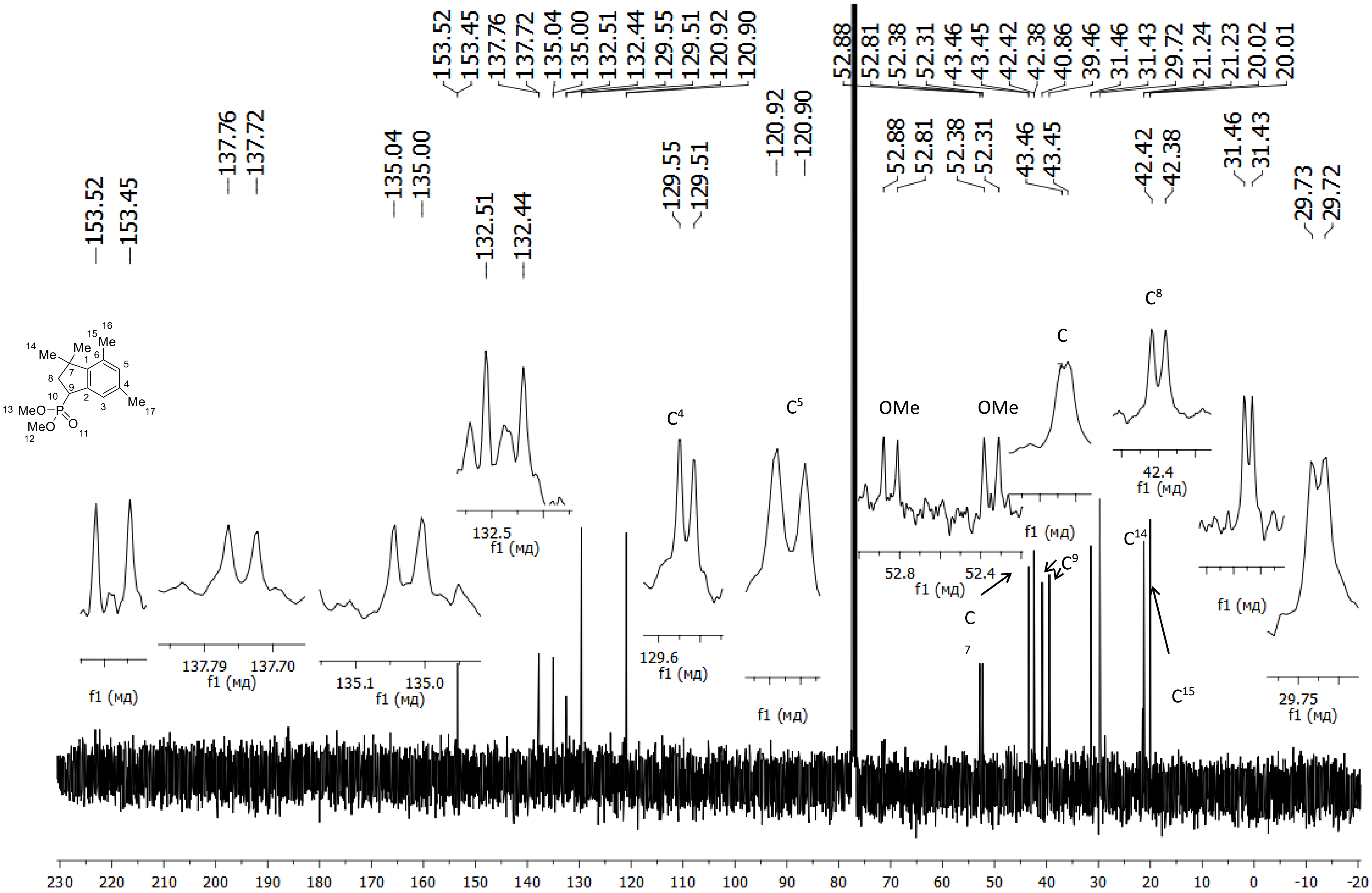

Figure S83. ^{13}C NMR spectrum of the mixture **12c** (101 MHz, $CDCl_3$).

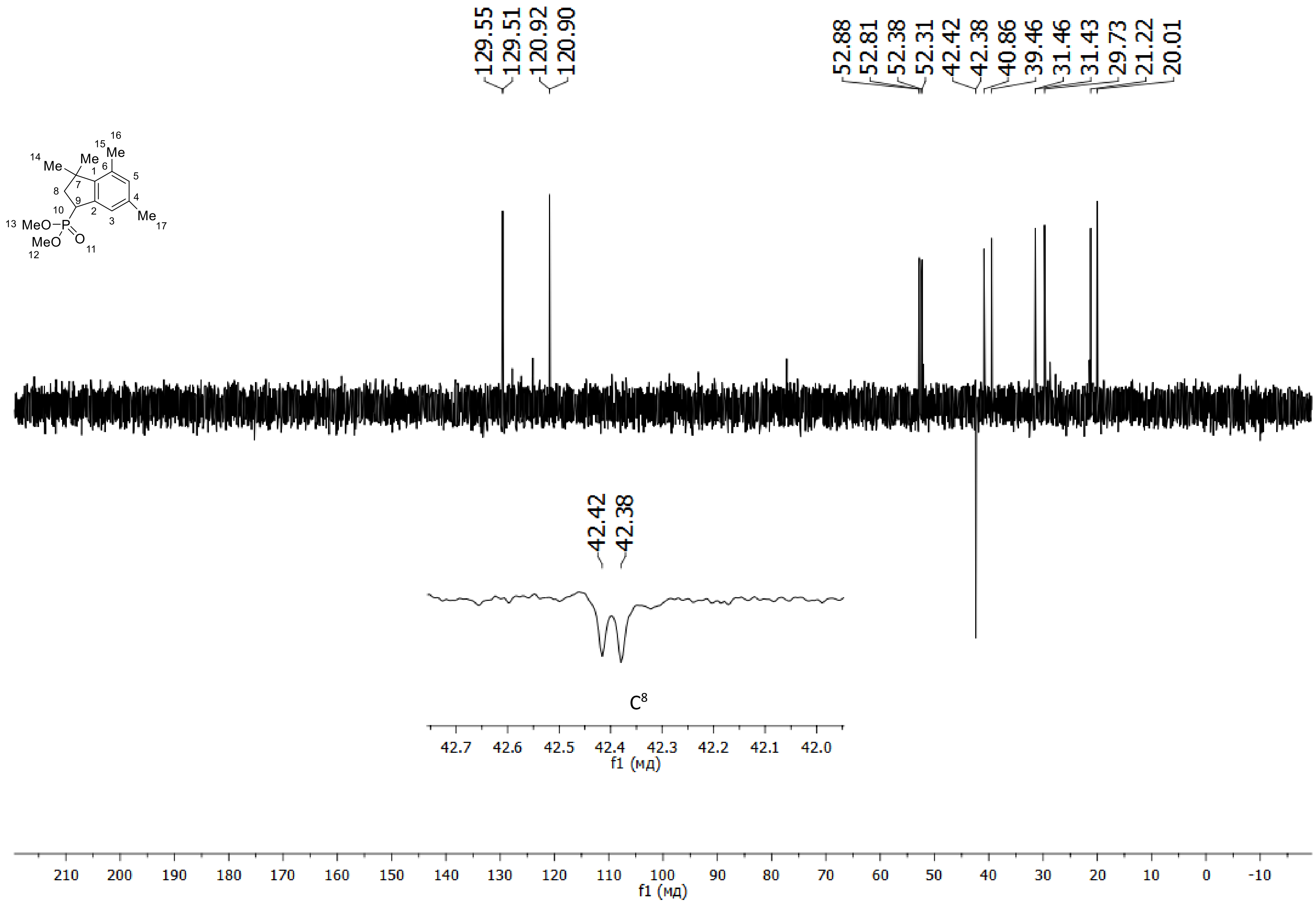

Figure S84. DEPT NMR spectrum of the compound **12c** (101 MHz, $CDCl_3$).

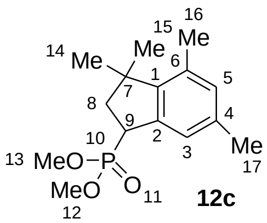

12c

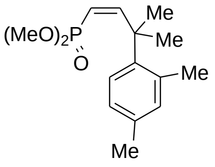

Z-11f

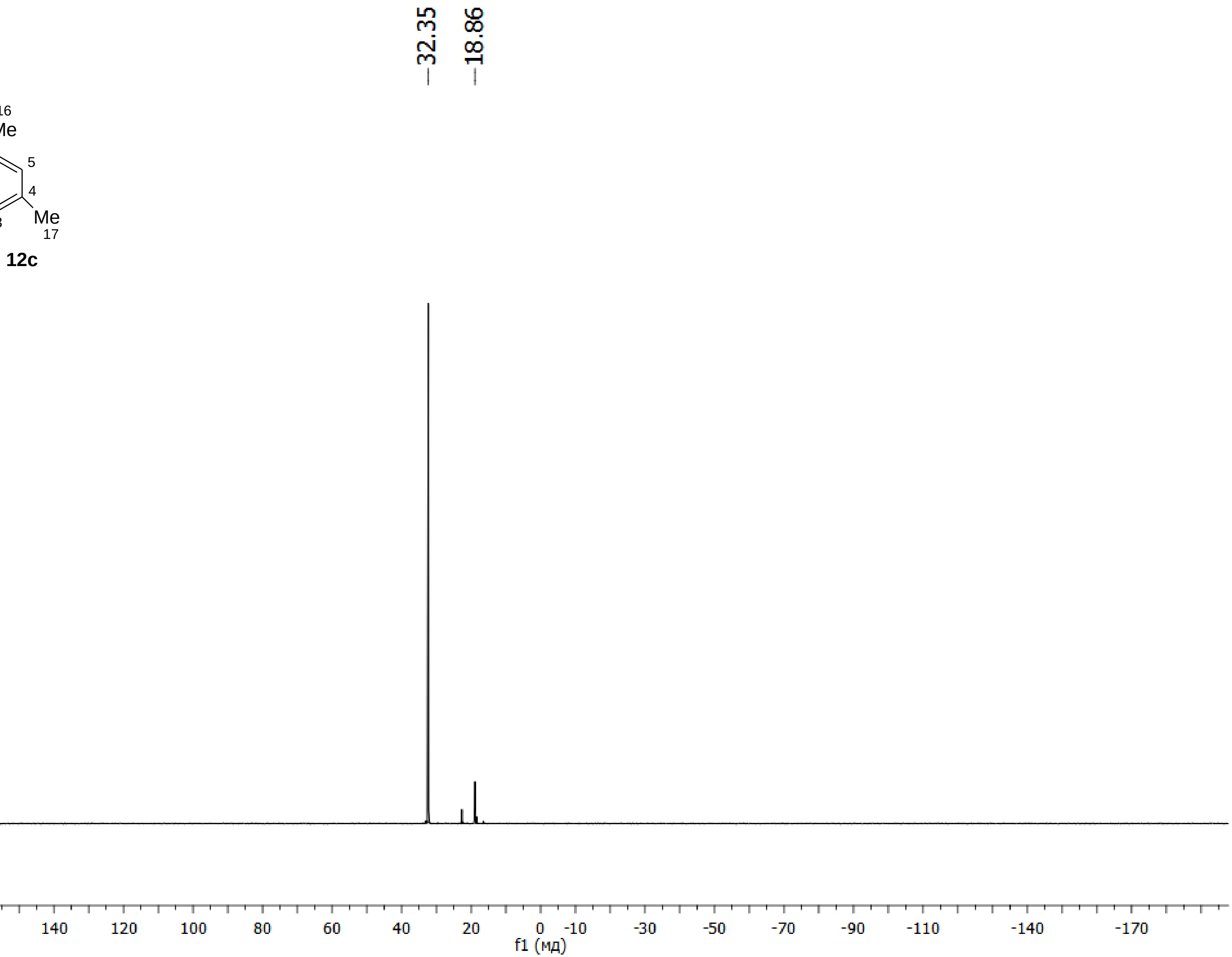

Figure S85. ^{31}P NMR spectrum of the mixture **12c+11f** (162 MHz, $CDCl_3$).

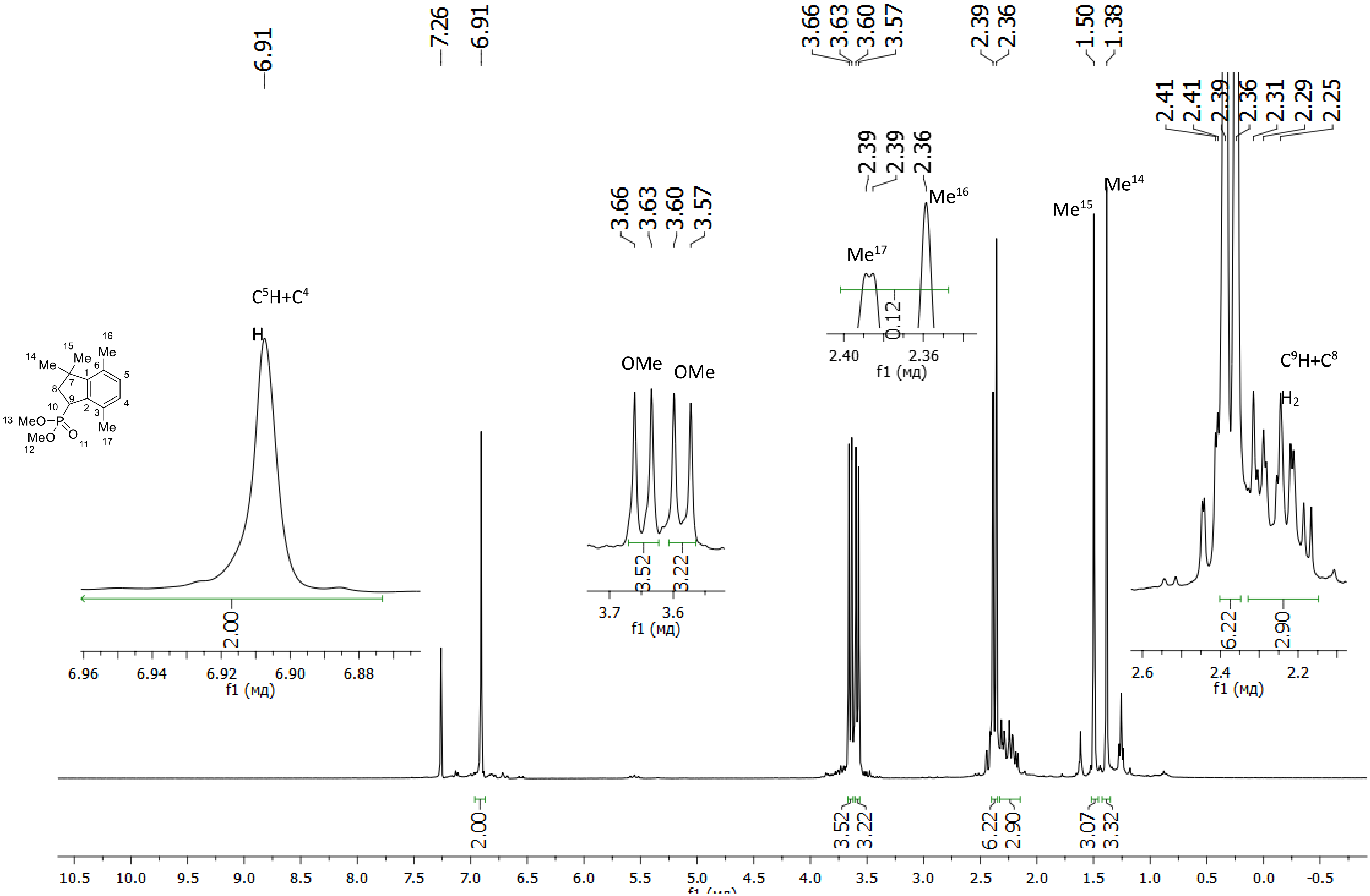

Figure S86. ^{1}H NMR spectrum of the compound **12d** (400 MHz, CDCl$_3$

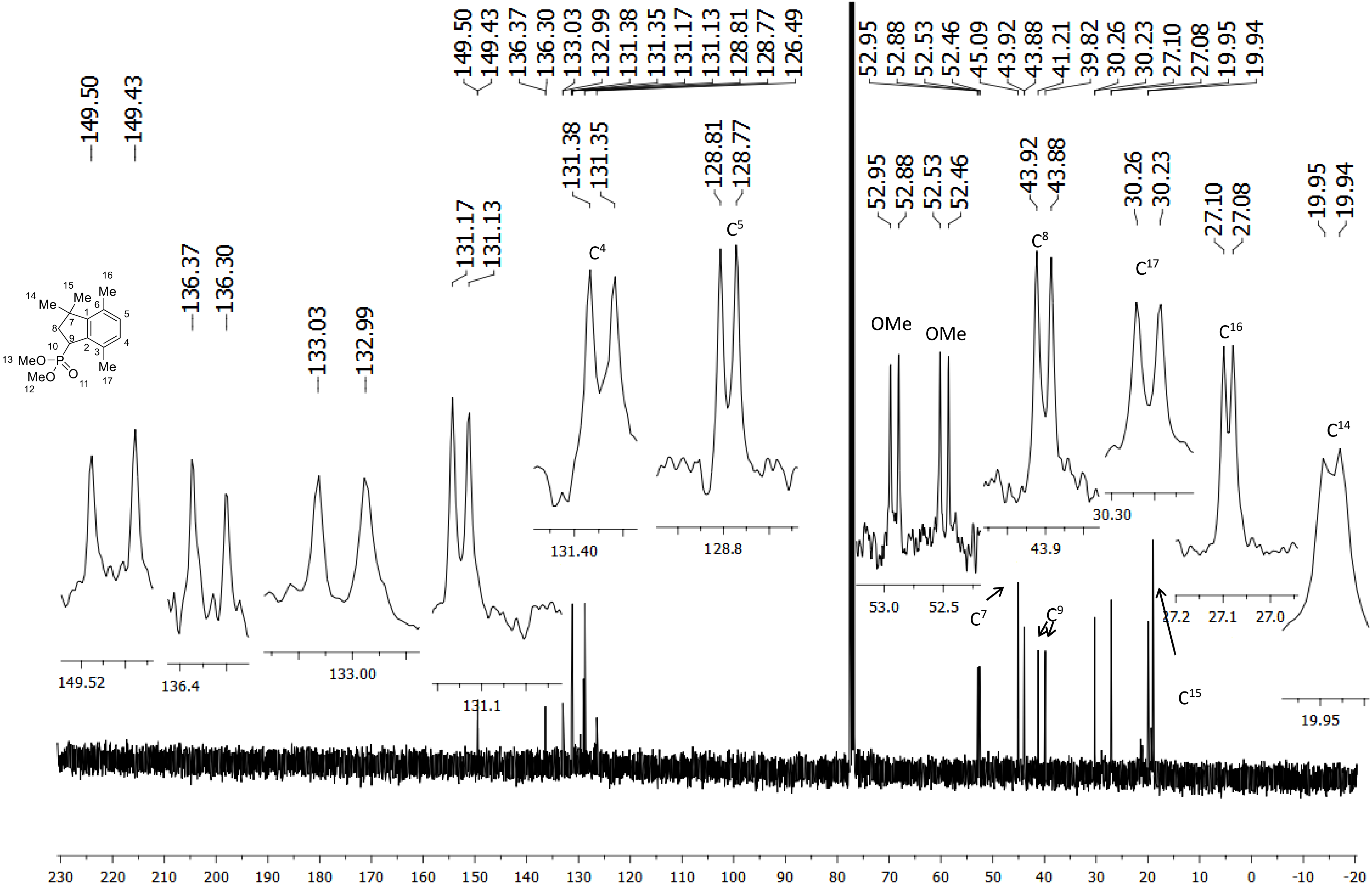

Figure S87. ^{13}C NMR spectrum of the compound **12d** (101 MHz, $CDCl_3$).

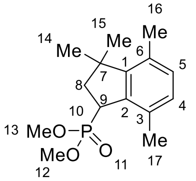

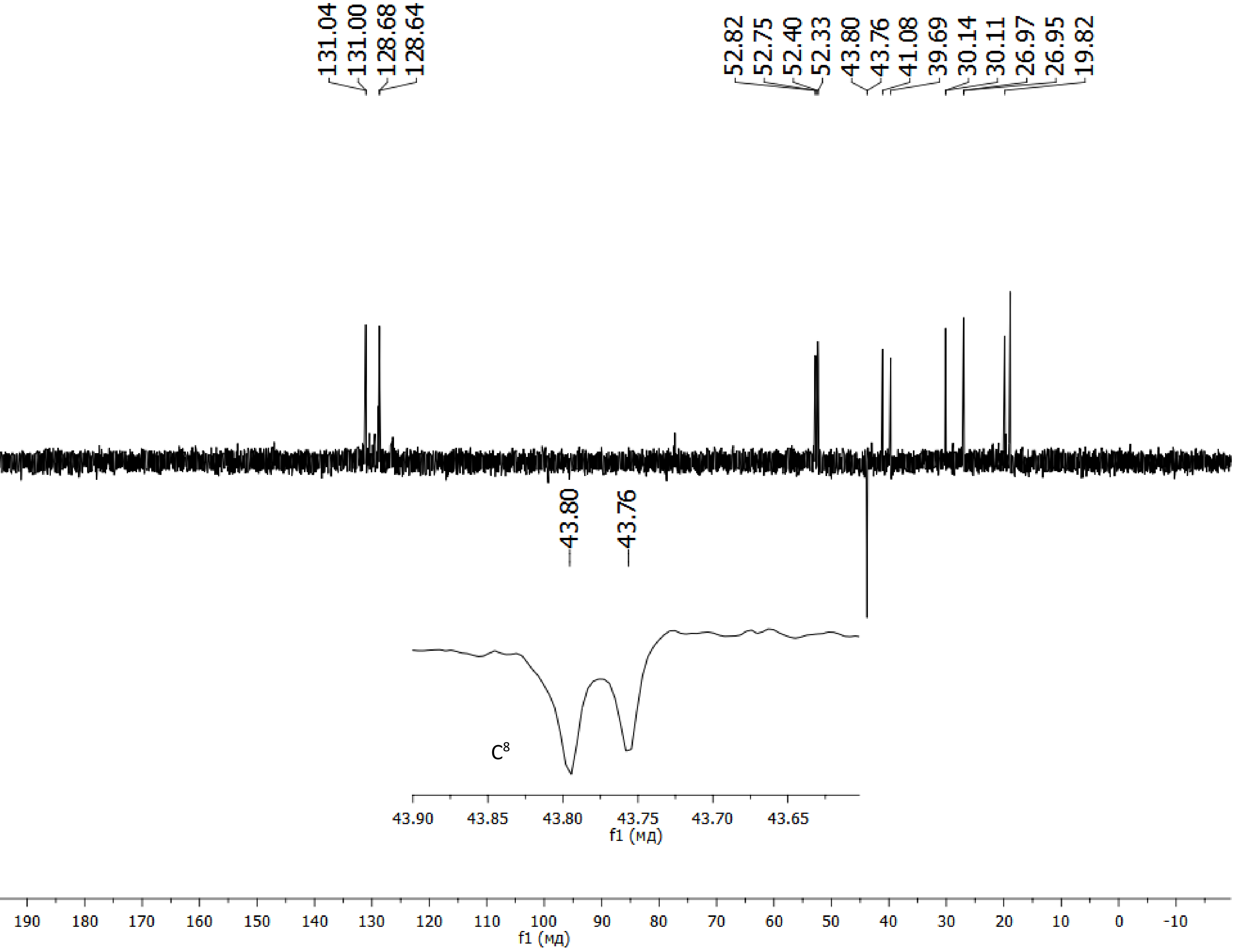

Figure S88. DEPT NMR spectrum of the compound **12d** (101 MHz, $CDCl_3$).

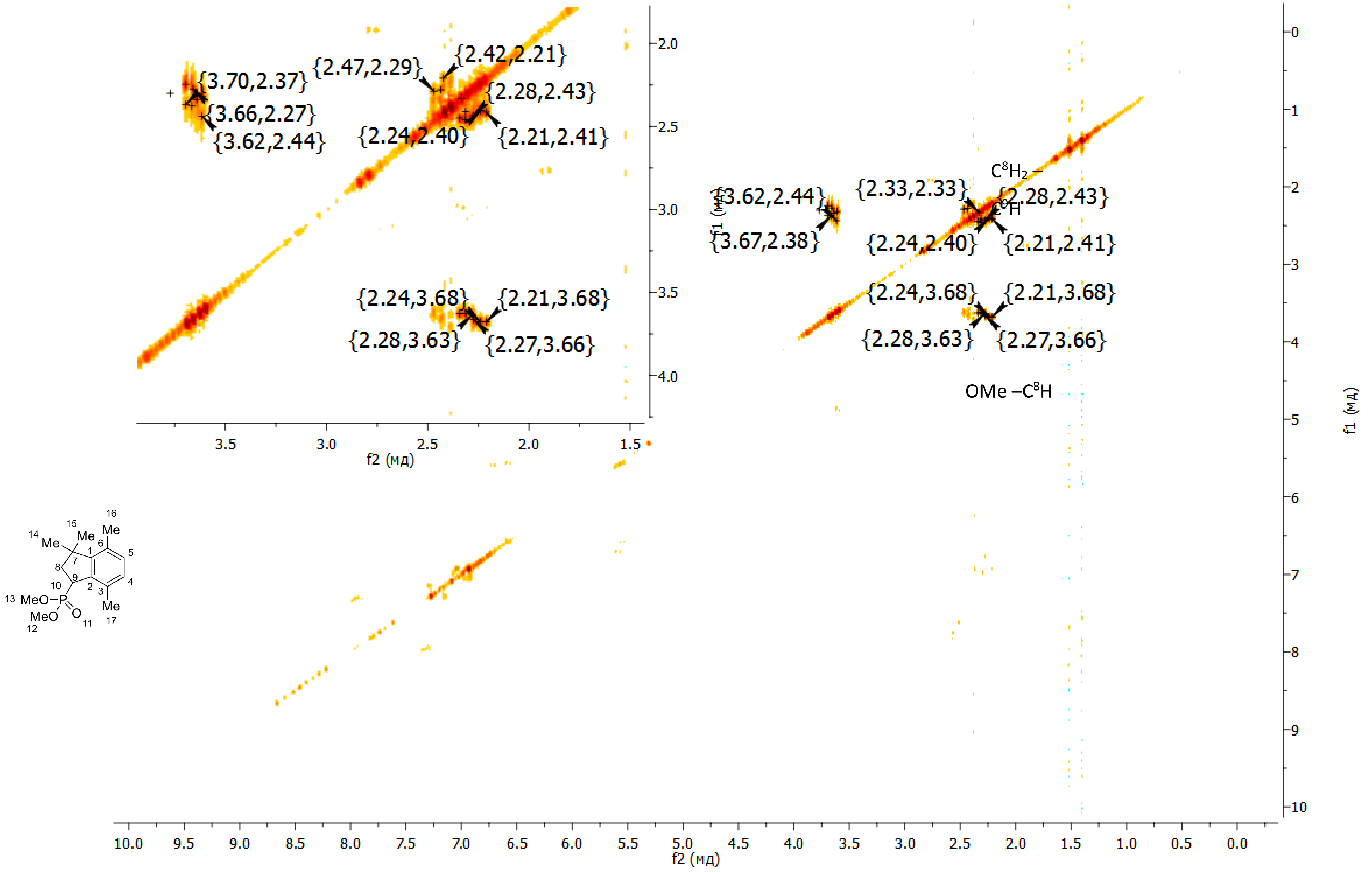

Figure S89. COSY NMR spectrum of the compound **12d** (400 MHz, $CDCl_3$).

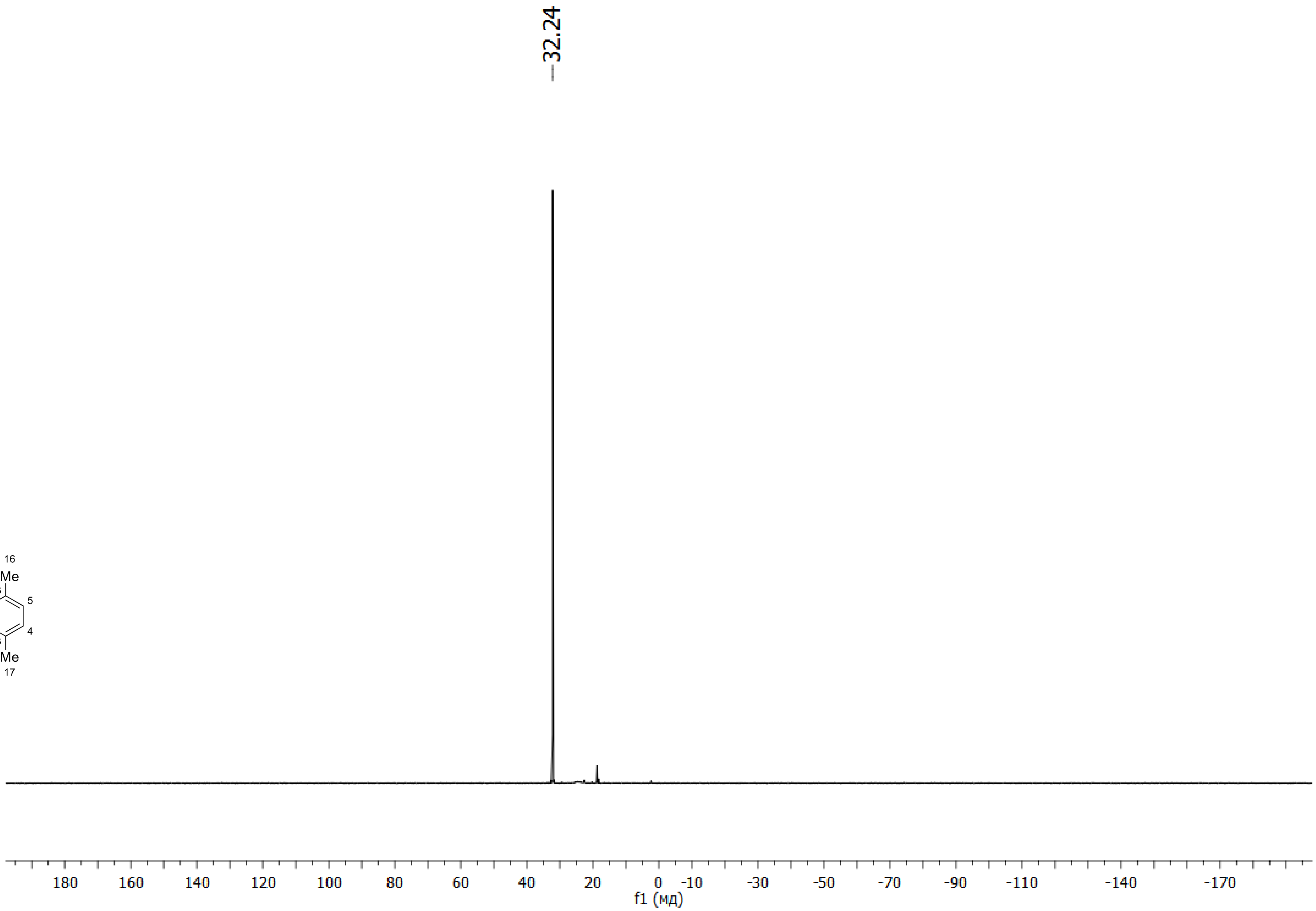

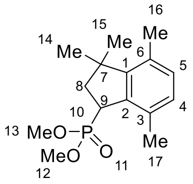

Figure S90. ^{31}P NMR spectrum of the compound **12d** (162 MHz, $CDCl_3$).

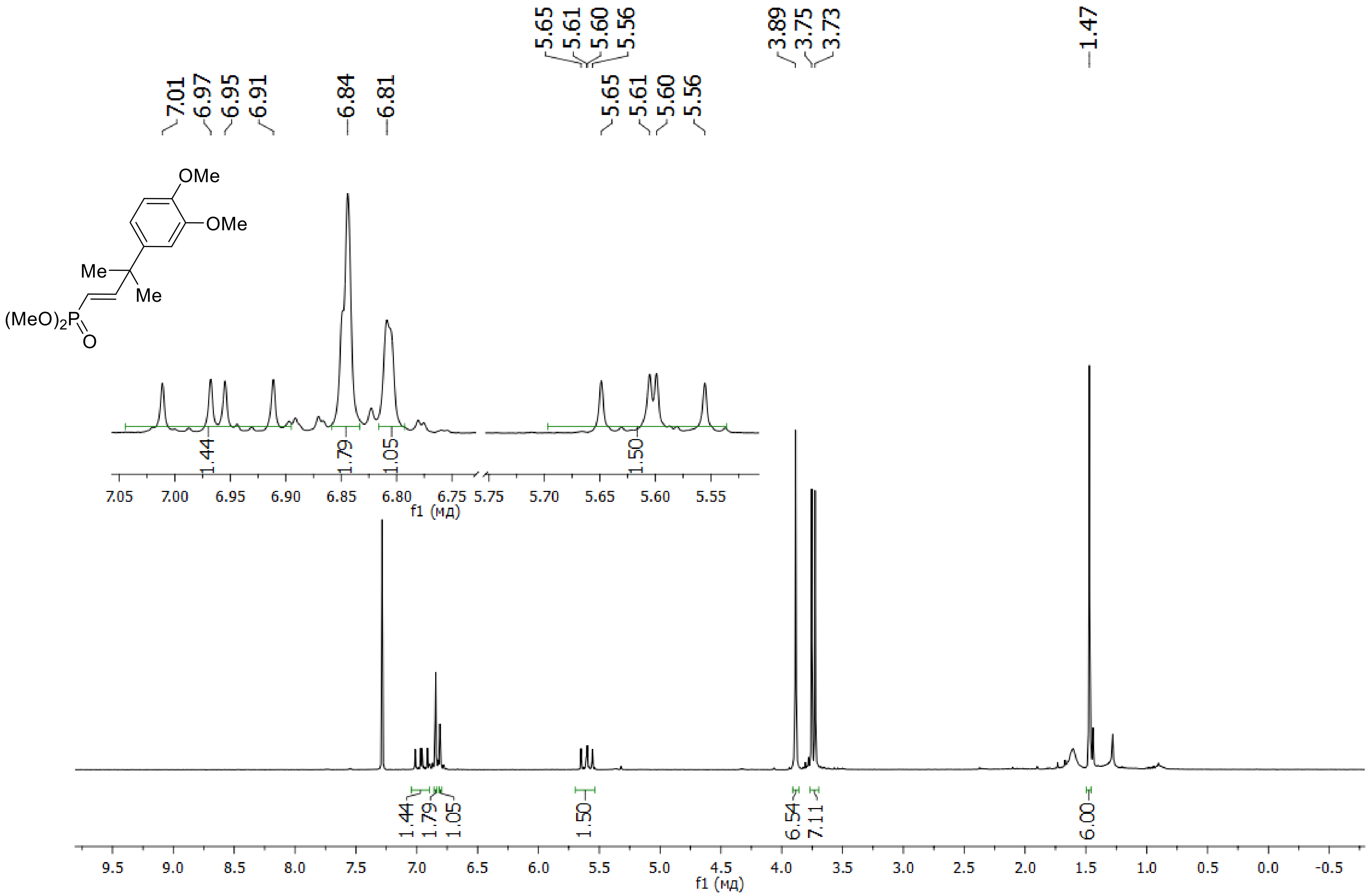

Figure S91. ^{1}H NMR spectrum of the compound **11g** (400 MHz, $CDCl_3$)

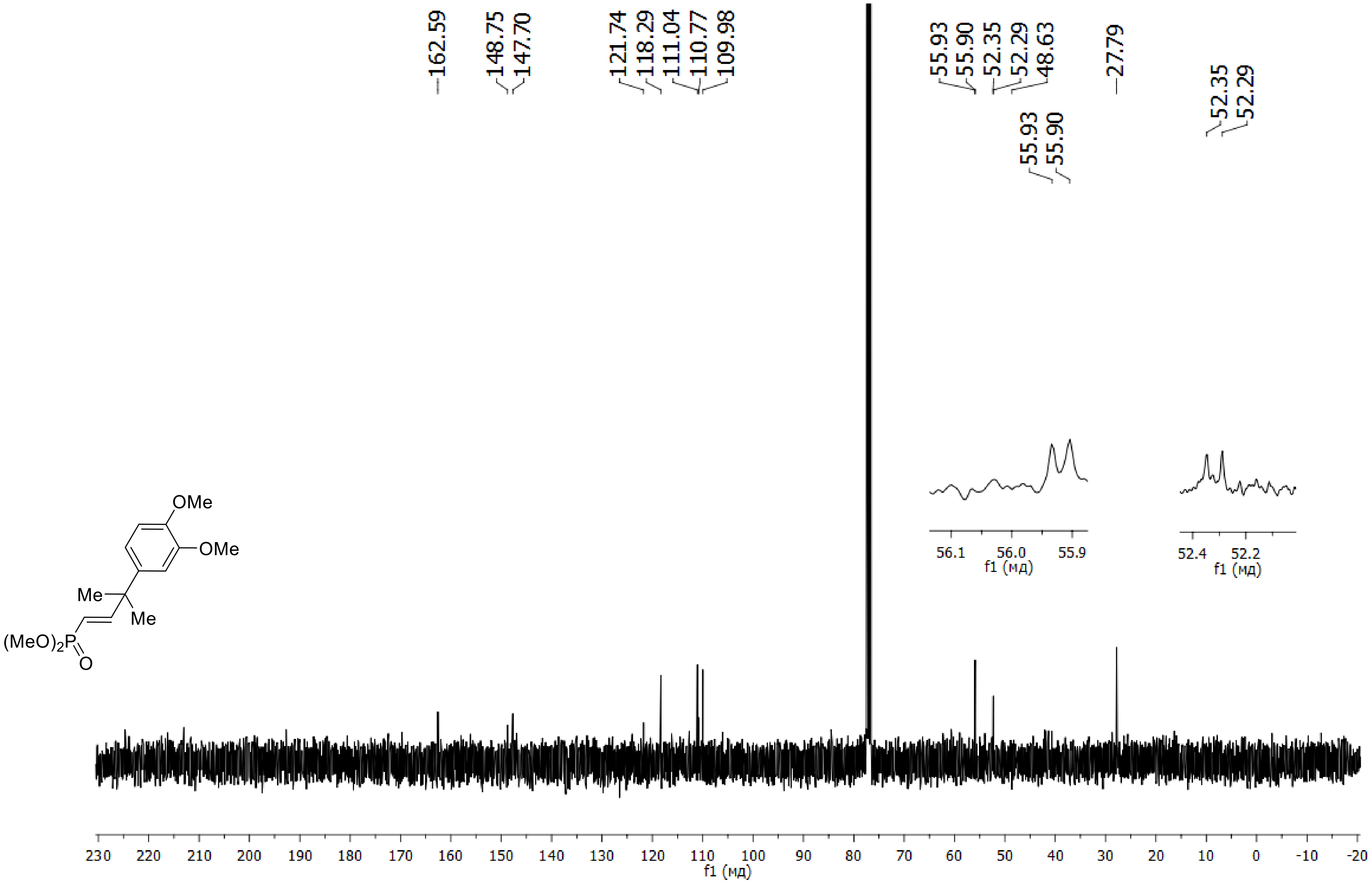

Figure S92. ^{13}C NMR spectrum of the compound **11g** (101 MHz, $CDCl_3$).

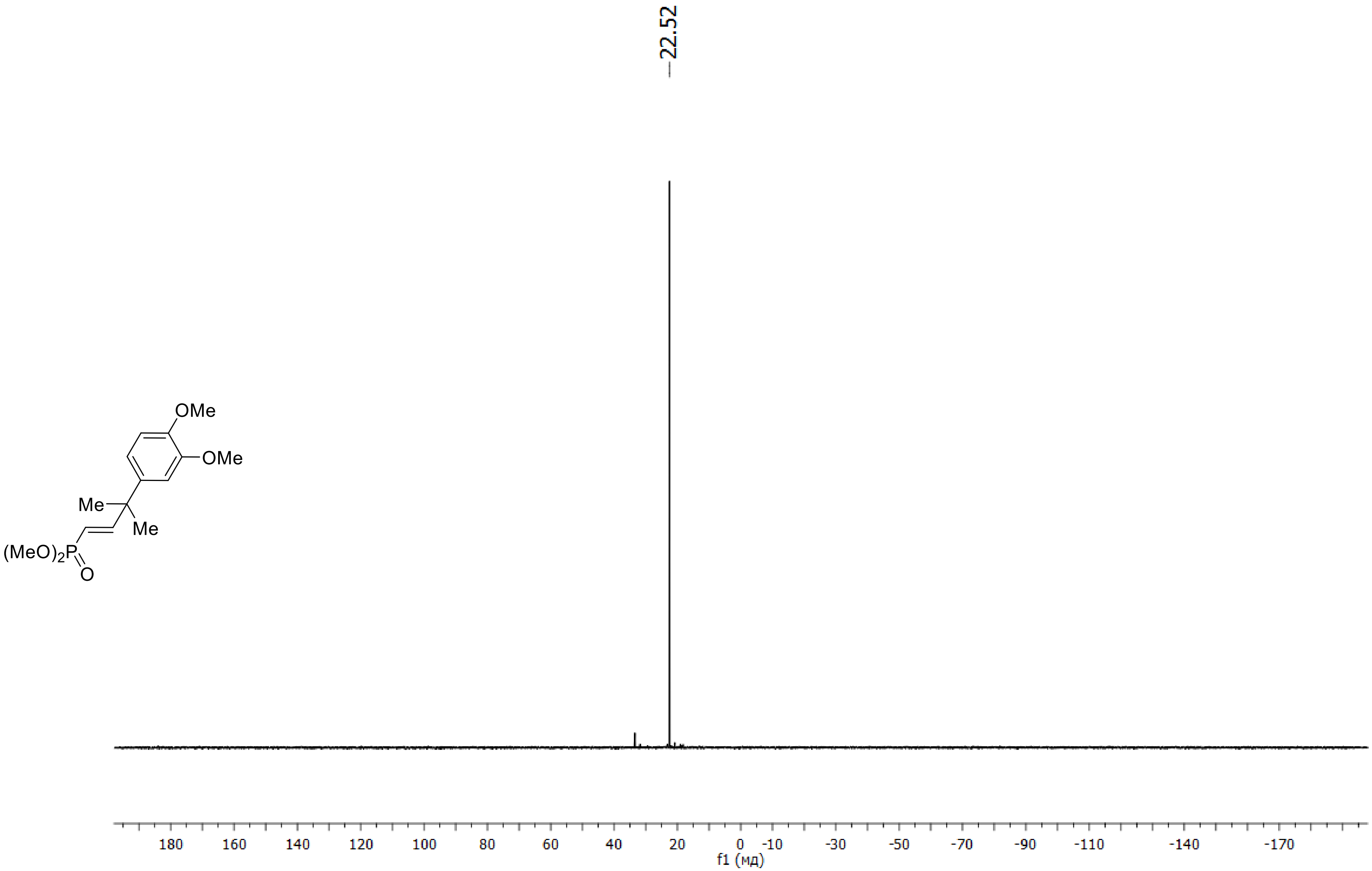

Figure S93. ^{31}P NMR spectrum of the compound **11g** (162 MHz, $CDCl_3$).

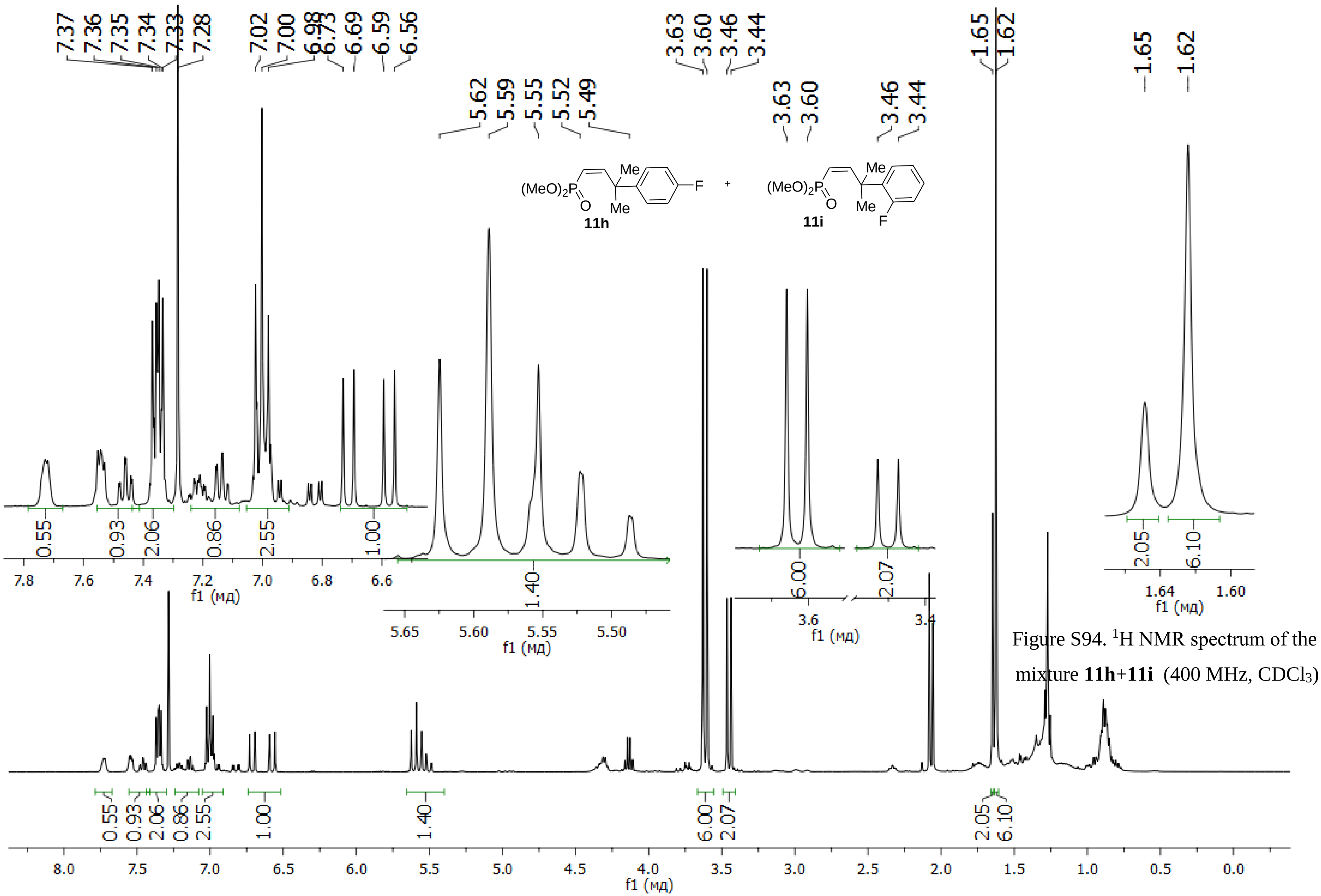

Figure S94. ^{1}H NMR spectrum of the mixture **11h**+**11i** (400 MHz, $CDCl_3$)

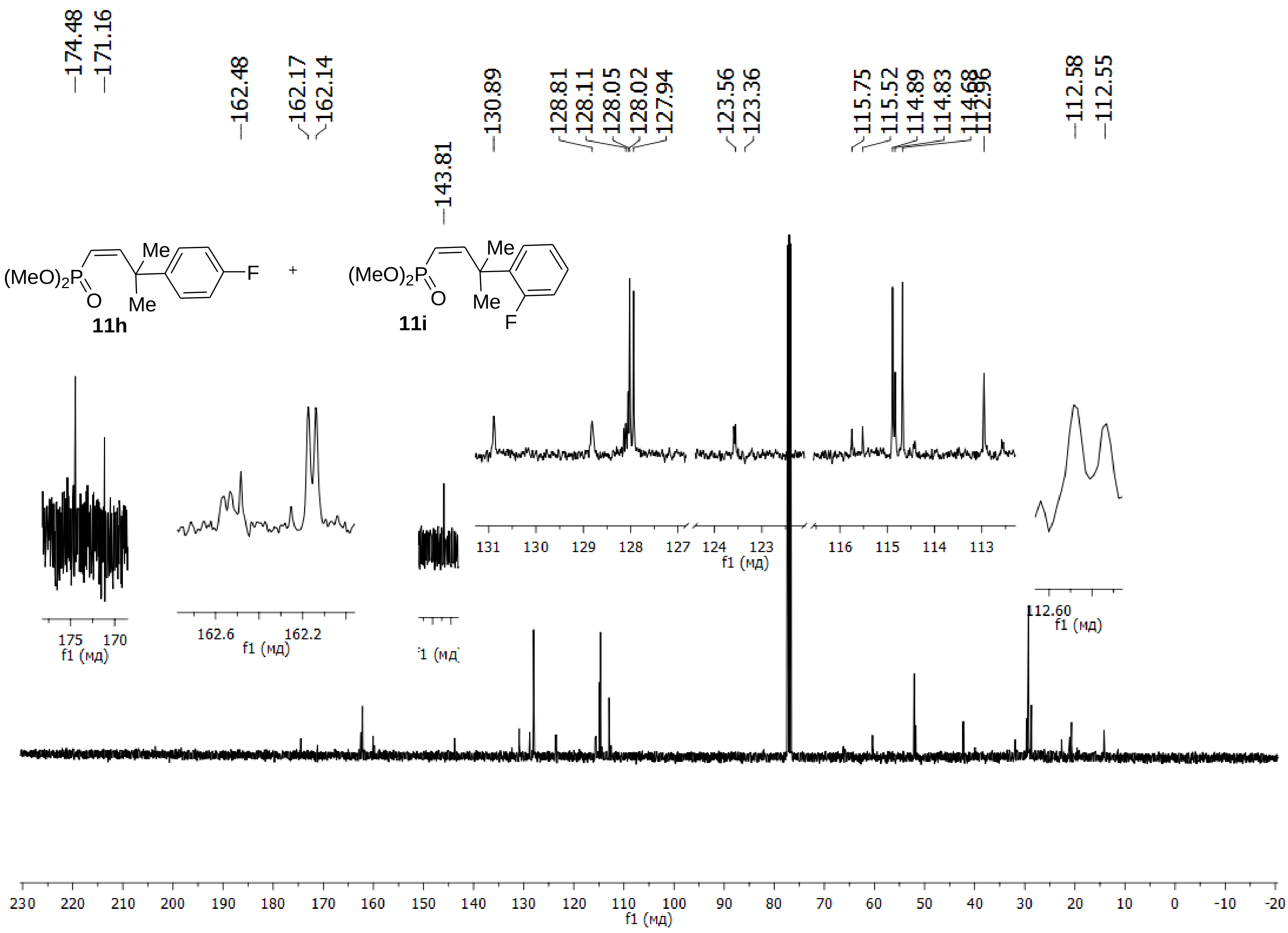

Figure S95. ^{13}C NMR spectrum of the mixture **11h+11i** (101 MHz, $CDCl_3$).

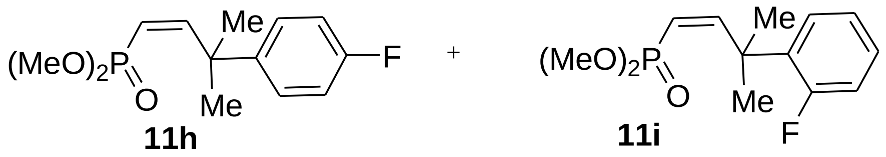

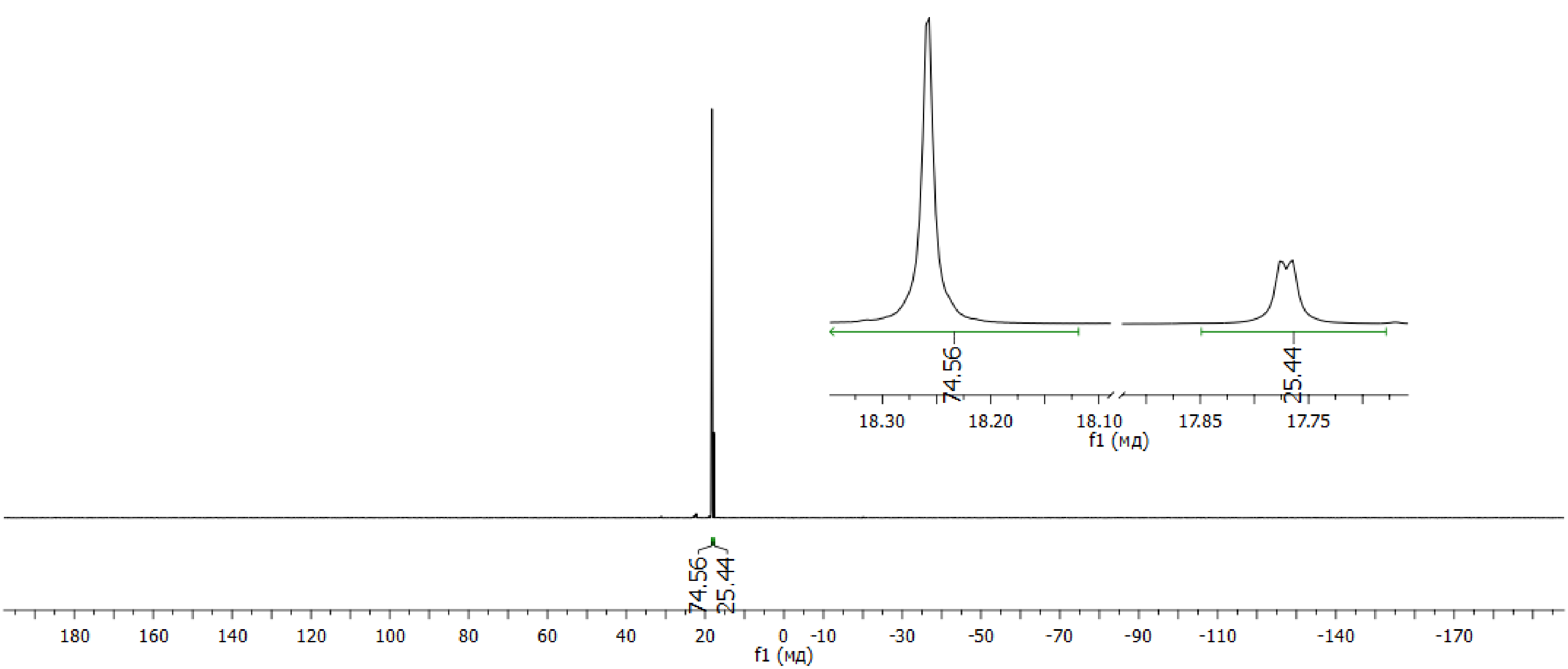

Figure S96. ^{31}P NMR spectrum of the mixture **11h+11i** (162 MHz, $CDCl_3$).

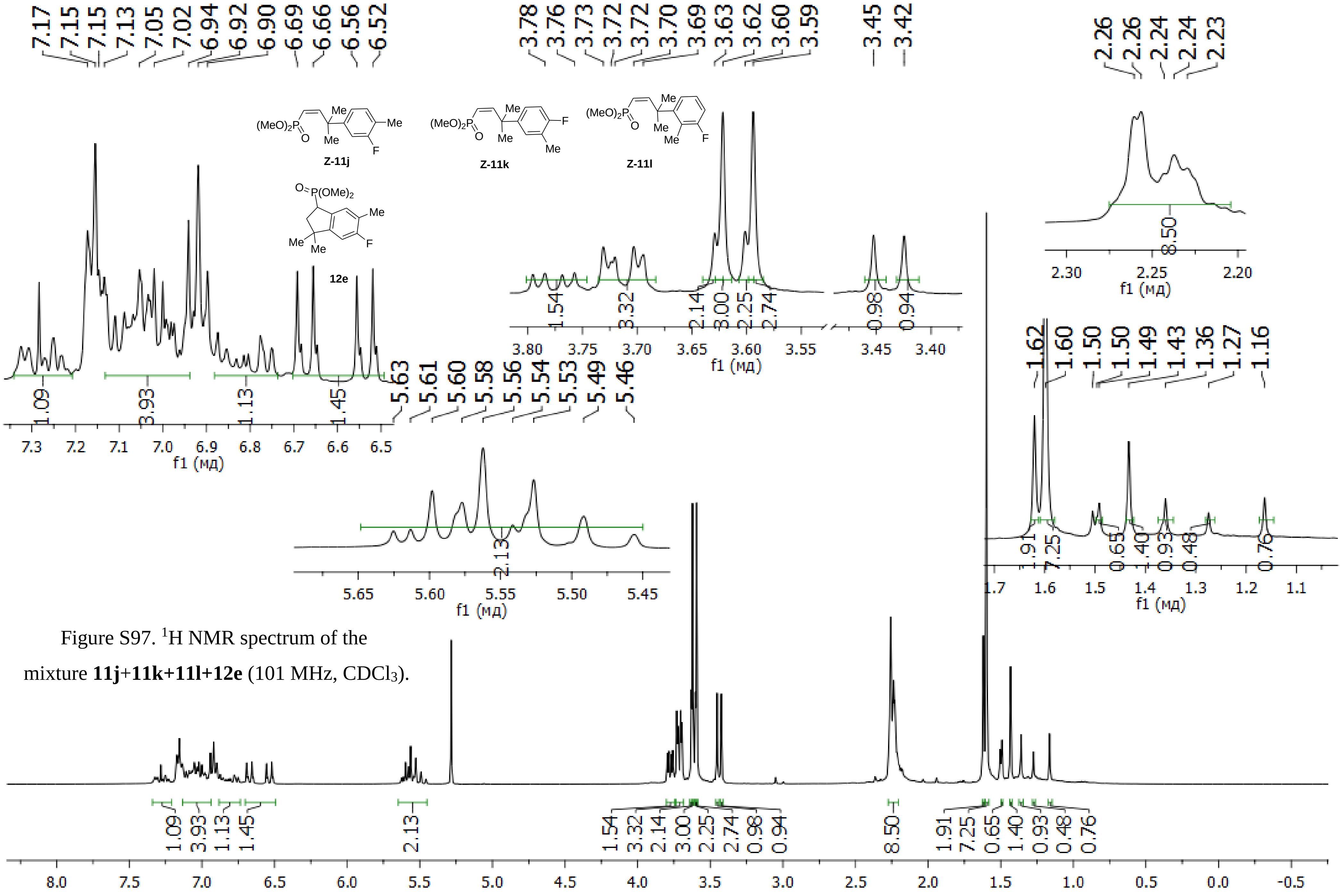

Figure S97. ^{1}H NMR spectrum of the mixture **11j**+**11k**+**11l**+**12e** (101 MHz, $CDCl_3$).

Figure S51. ^{1}H NMR spectrum of the mixture **11j**+**11k**+**11l**+**12e** (400 MHz, $CDCl_3$)

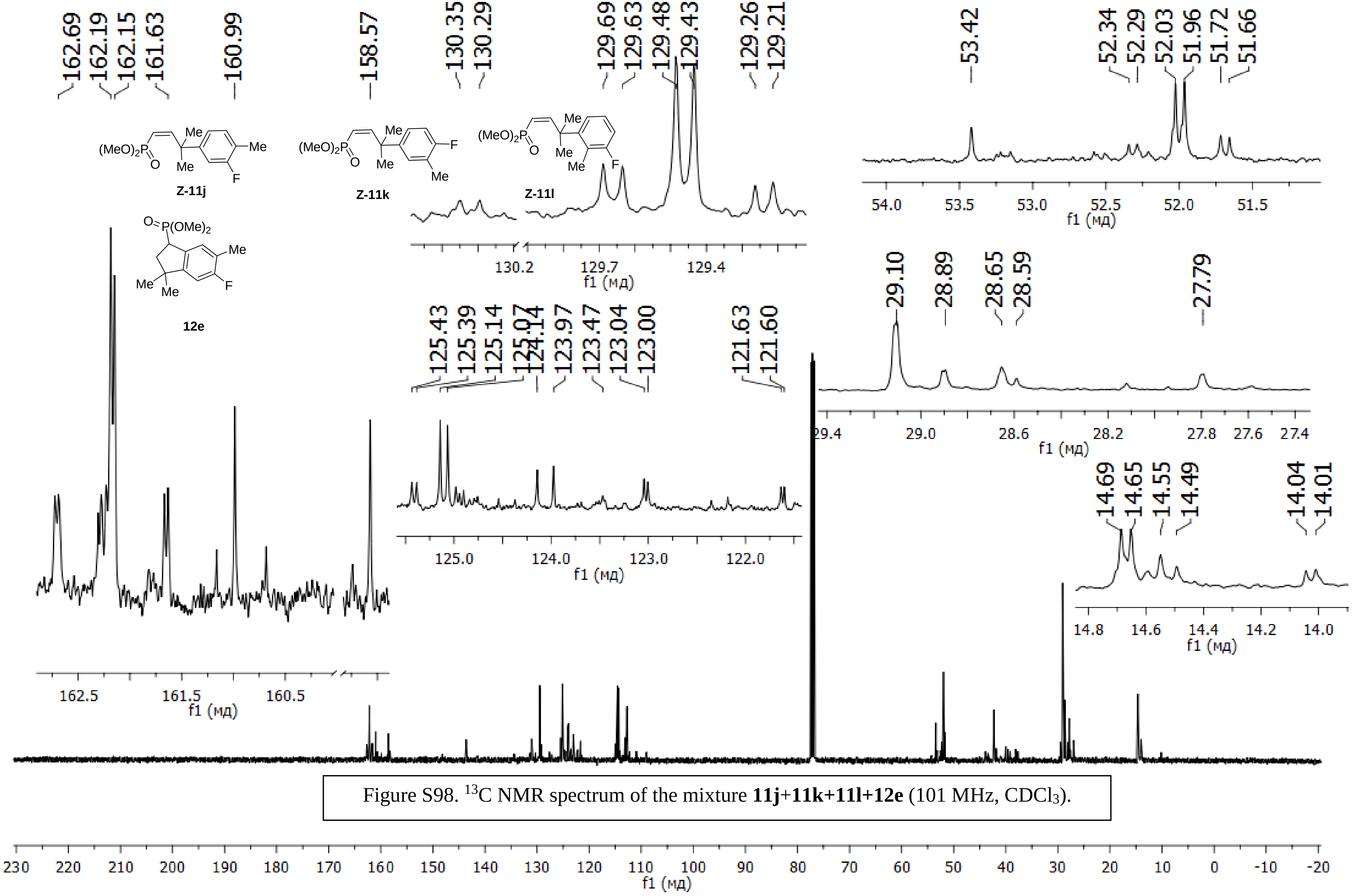

Figure S98. ^{13}C NMR spectrum of the mixture **11j**+**11k**+**11l**+**12e** (101 MHz, $CDCl_3$).

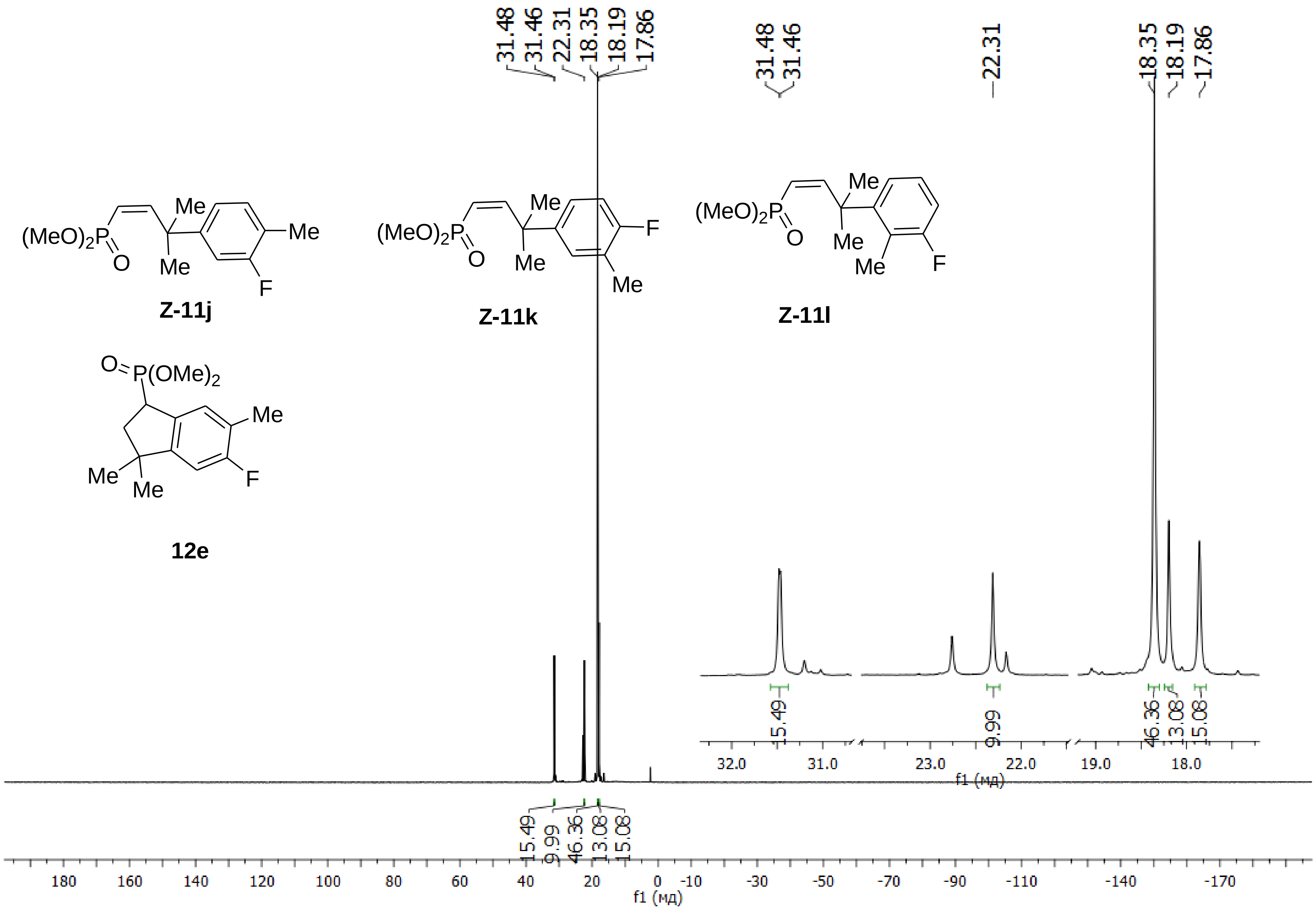

Figure S99. ^{31}P NMR spectrum of the mixture **11j+11k+11l+12e** (162 MHz, $CDCl_3$).

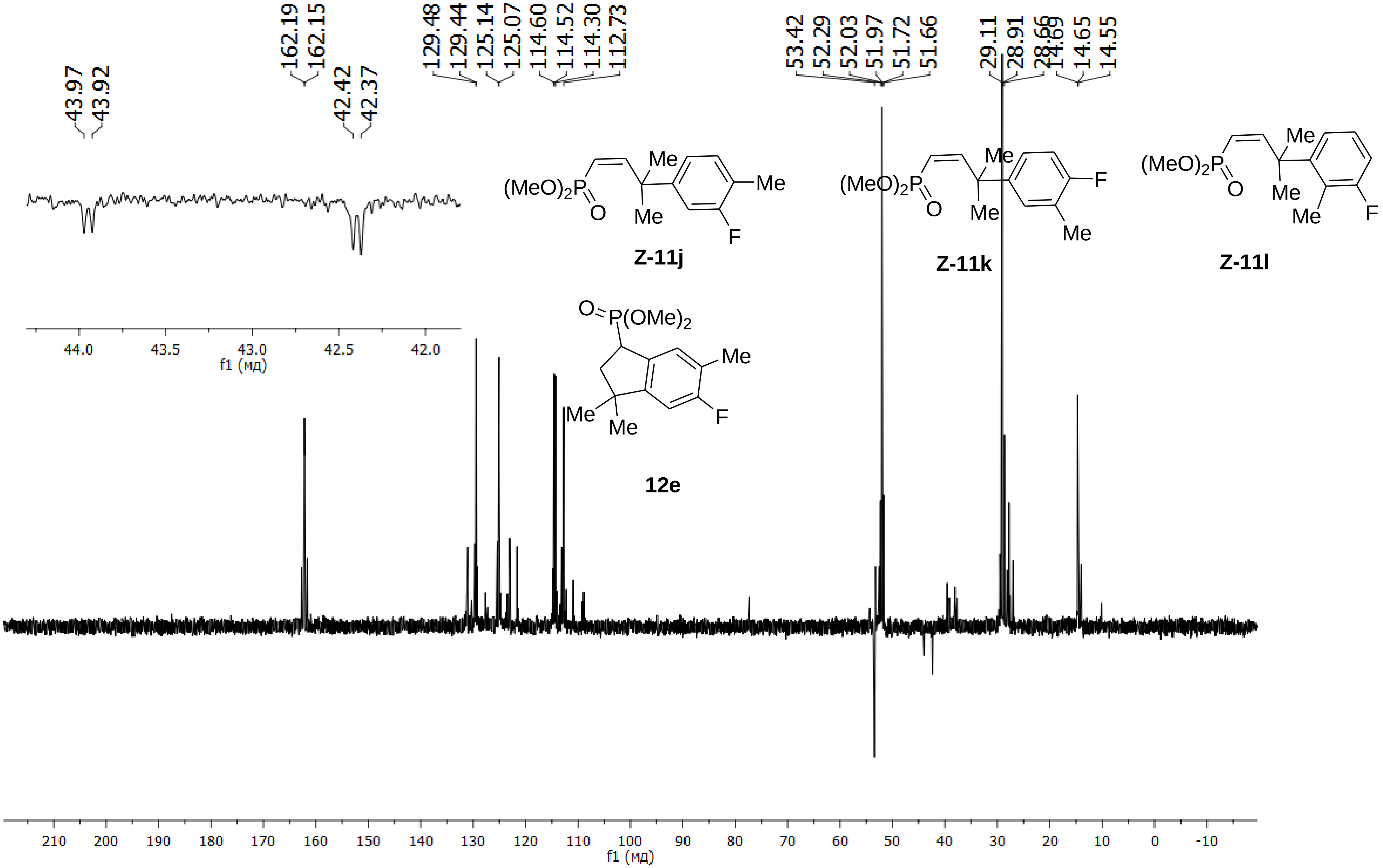

Figure S100. DEPT NMR spectrum of the mixture **11j+11k+11l+12e** (101 MHz, $CDCl_3$).

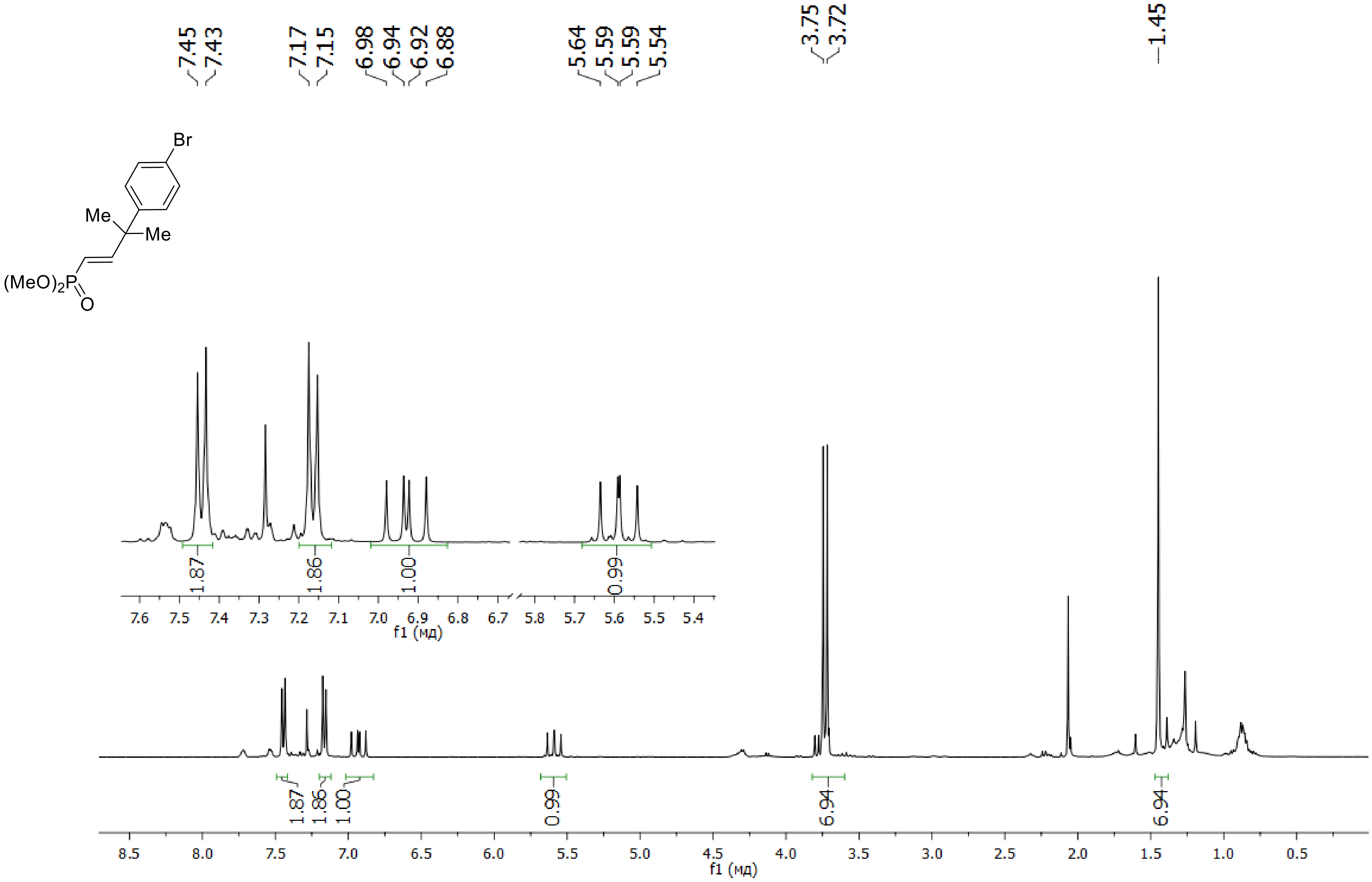

Figure S101. ^{1}H NMR spectrum of the compound *E*-**11m** (400 MHz, $CDCl_3$)

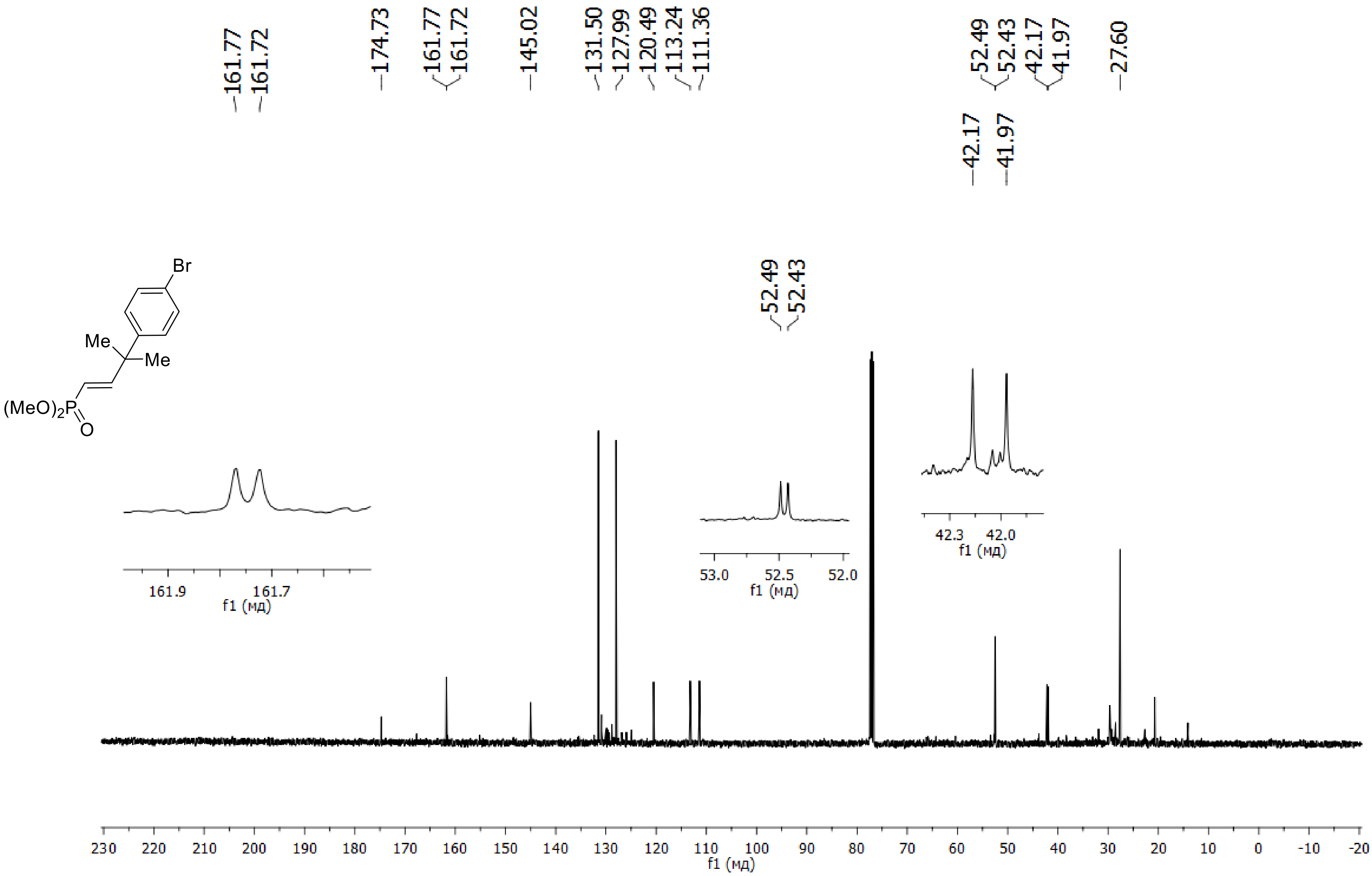

Figure S102. ^{13}C NMR spectrum of the compound *E*-**11m** (101 MHz, $CDCl_3$)

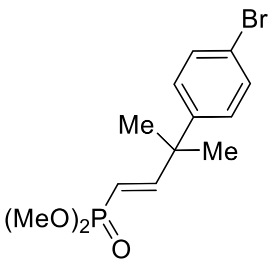

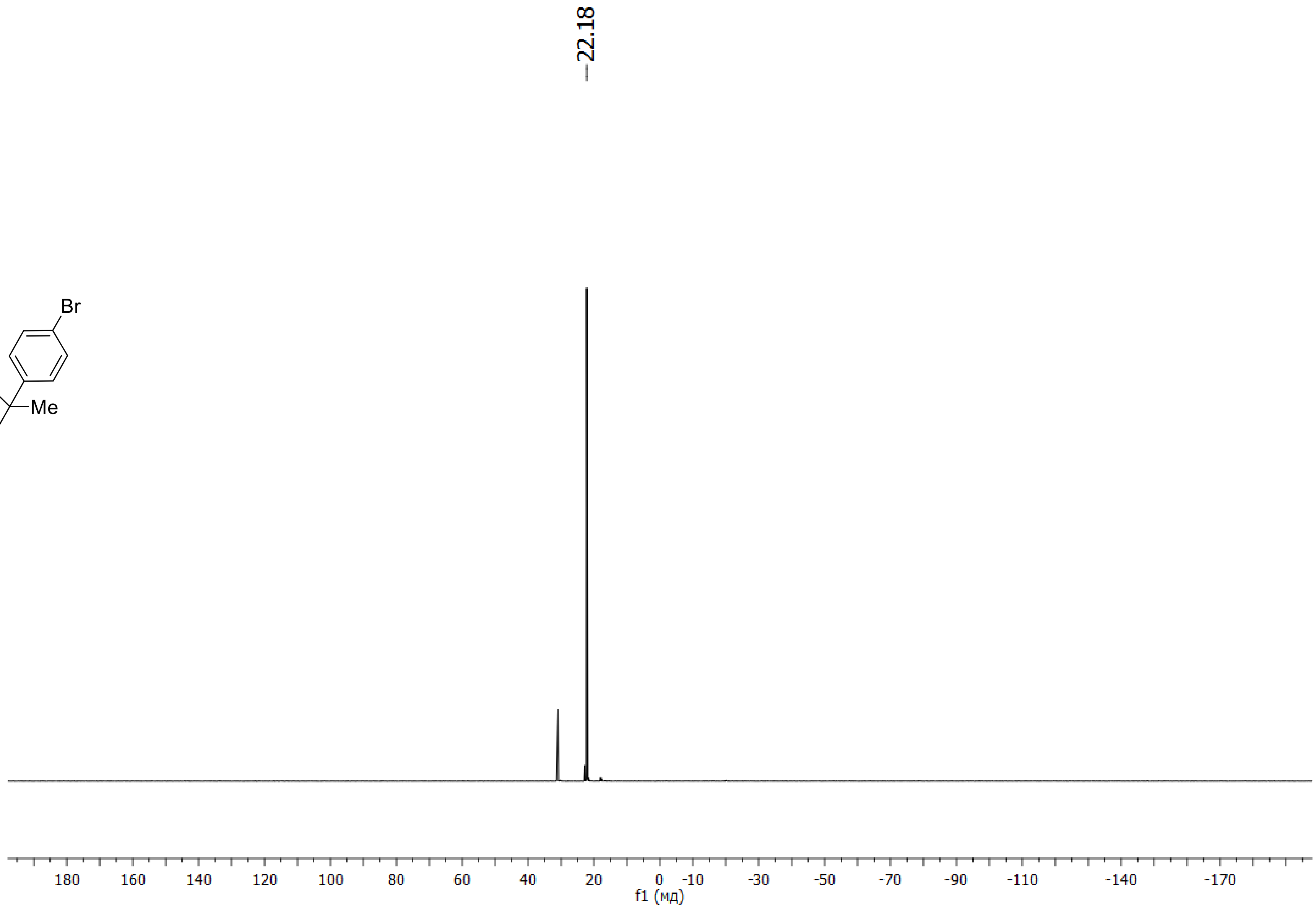

Figure S103. ^{31}P NMR spectrum of the compound *E*-**11m** (162 MHz, $CDCl_3$)

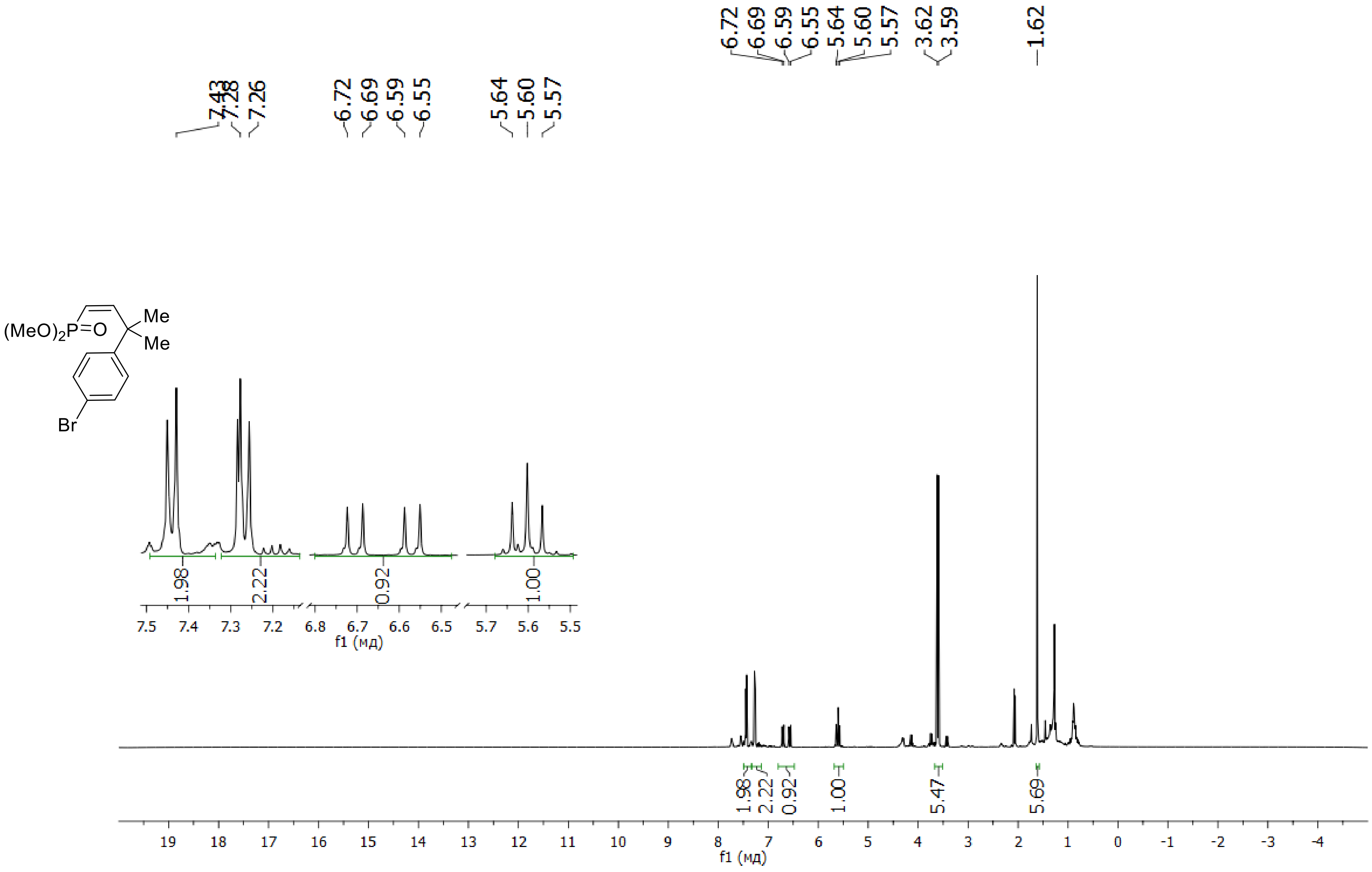

Figure S104. ^{1}H NMR spectrum of the compound *Z*-**11m** (400 MHz, $CDCl_3$)

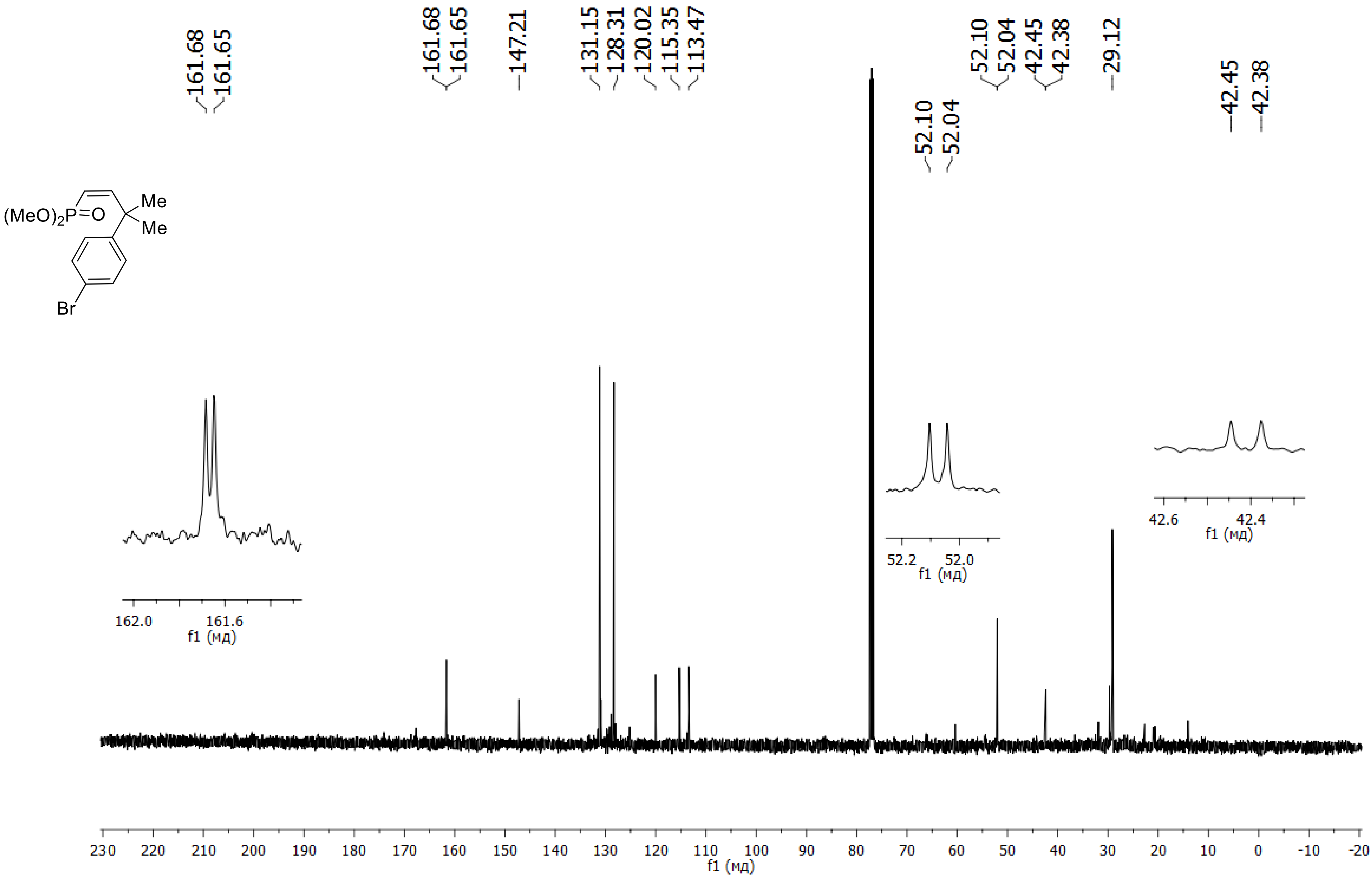

Figure S105. ^{13}C NMR spectrum of the compound *Z*-**11m** (101 MHz, $CDCl_3$)

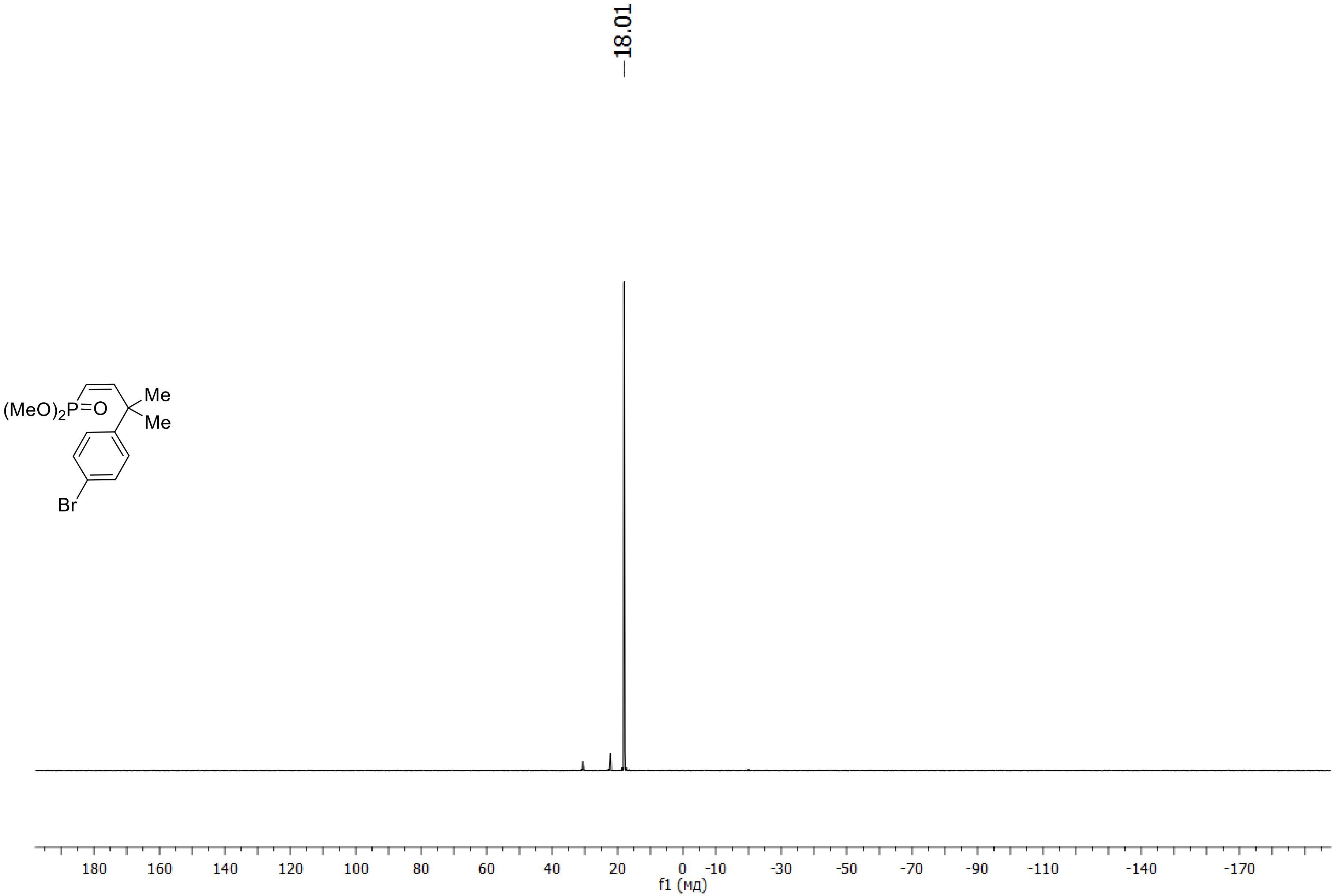

Figure S106. ^{31}P NMR spectrum of the compound *E*-**11m** (162 MHz, $CDCl_3$)

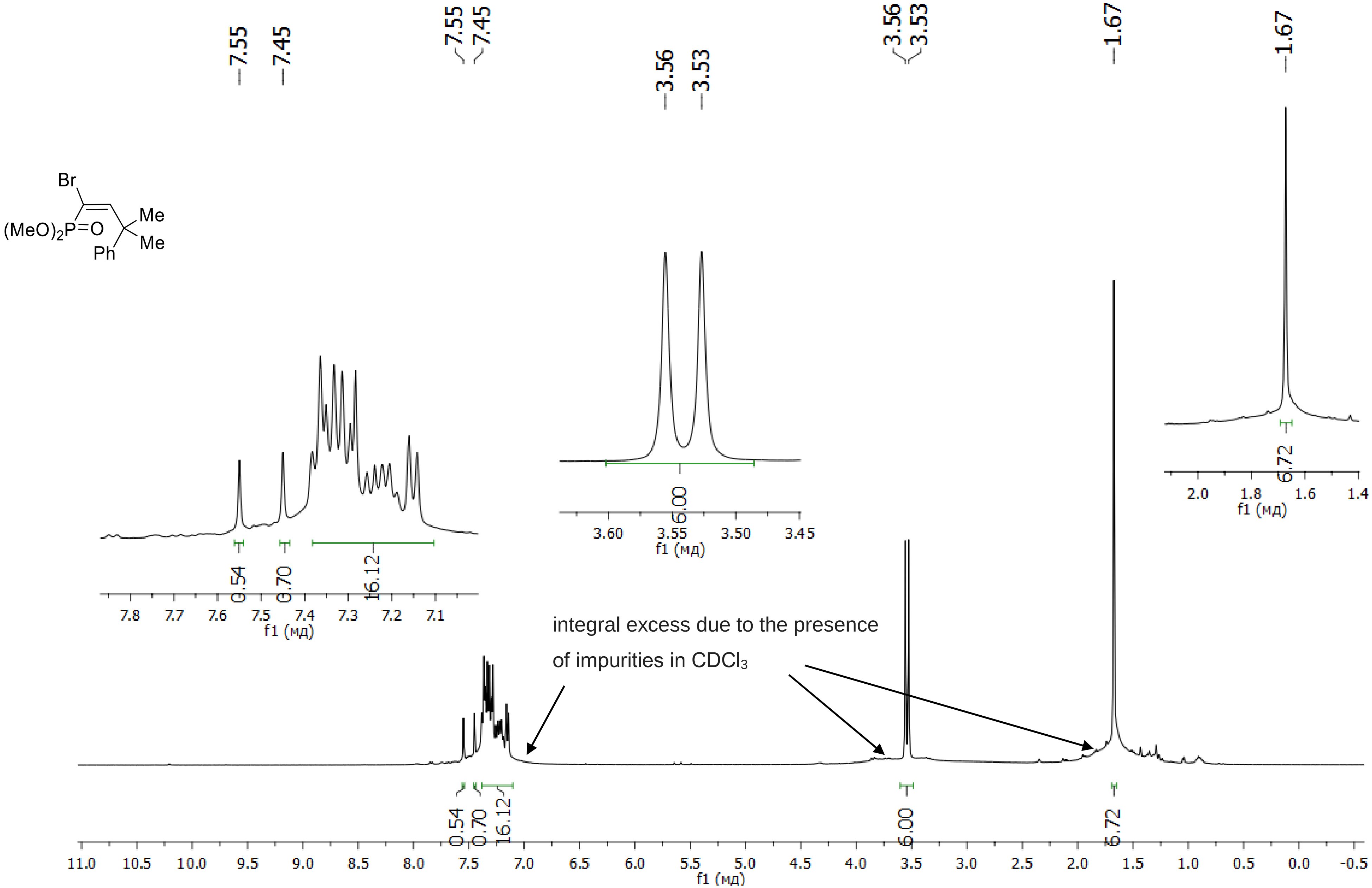

Figure S107. ^{1}H NMR spectrum of the compound **11n** (400 MHz, $CDCl_3$)

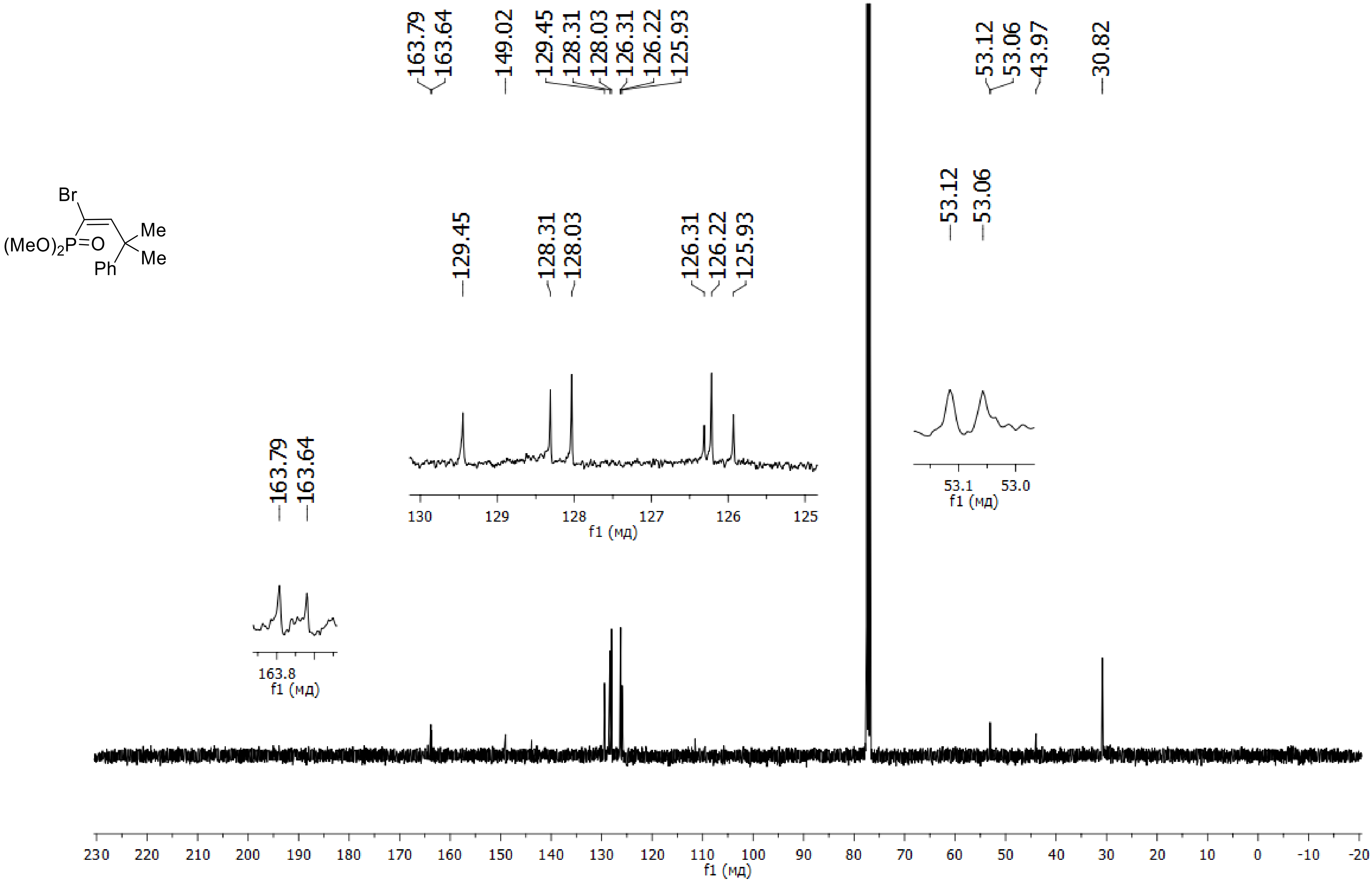

Figure S108. ^{13}C NMR spectrum of the compound **11n** (101 MHz, $CDCl_3$)

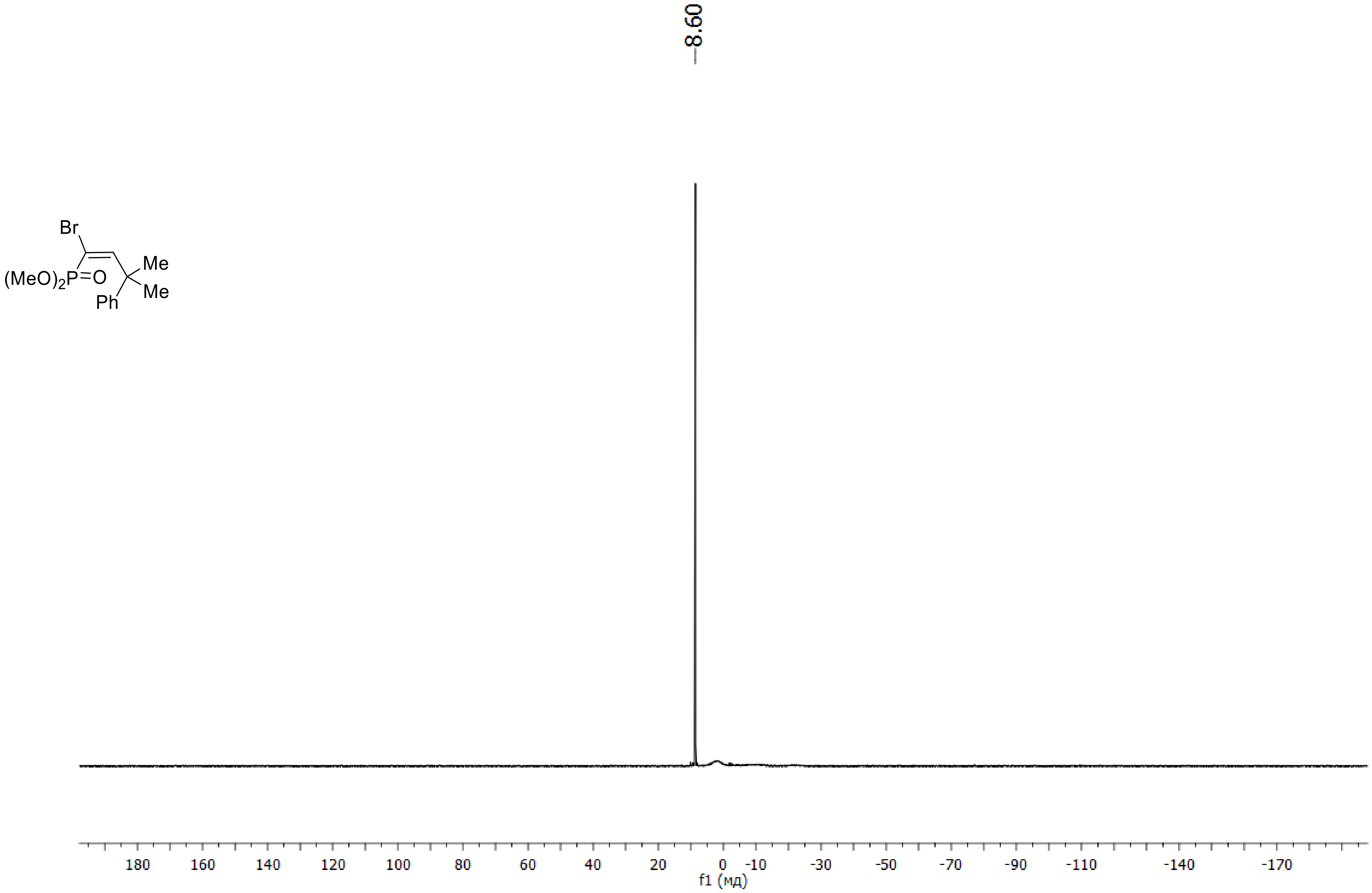

Figure S109. ^{31}P NMR spectrum of the compound **11n** (162 MHz, $CDCl_3$)

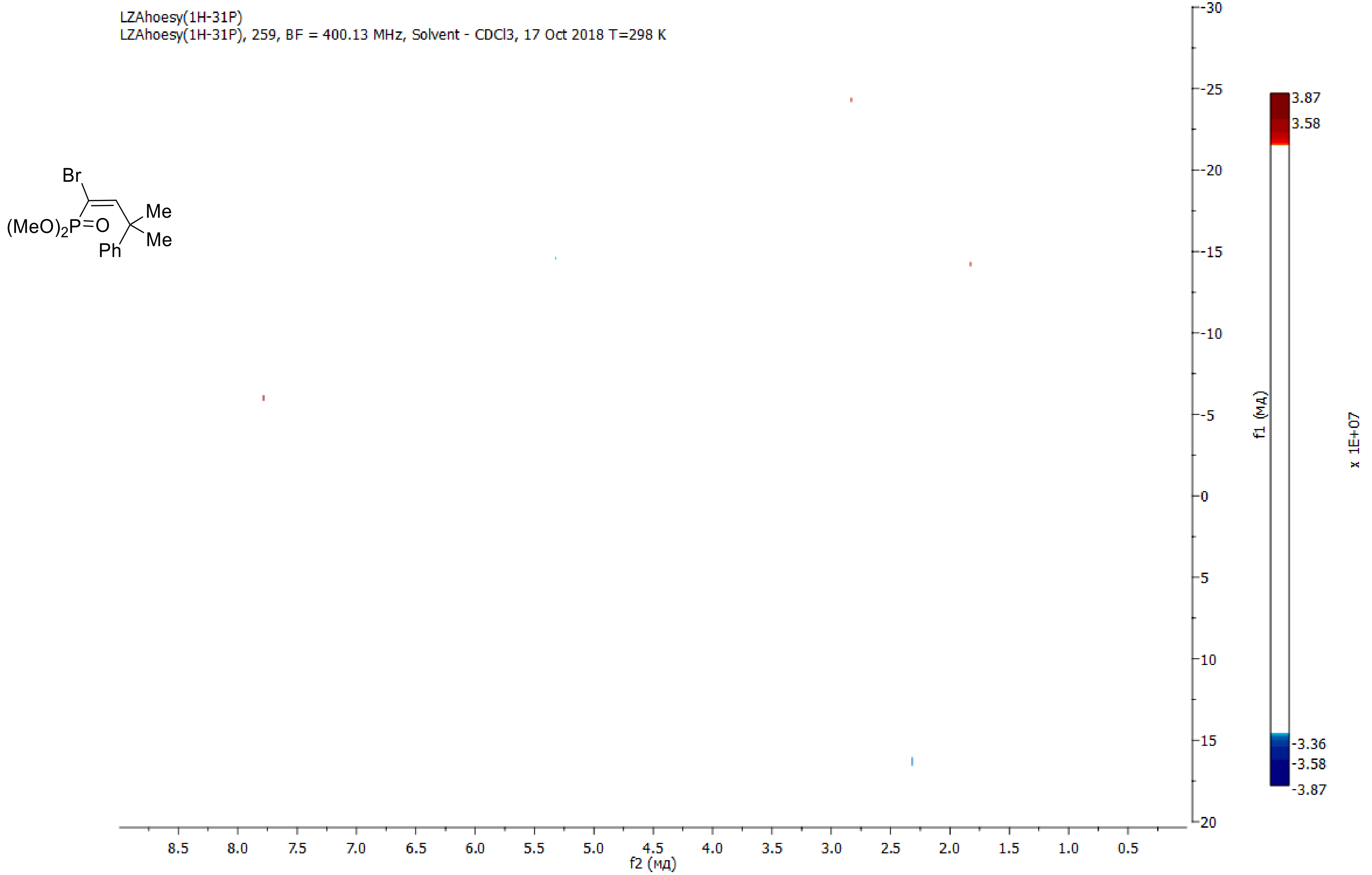

Figure S110. P-H HOESY NMR spectrum of the compound **11n** (400 MHz, $CDCl_3$)

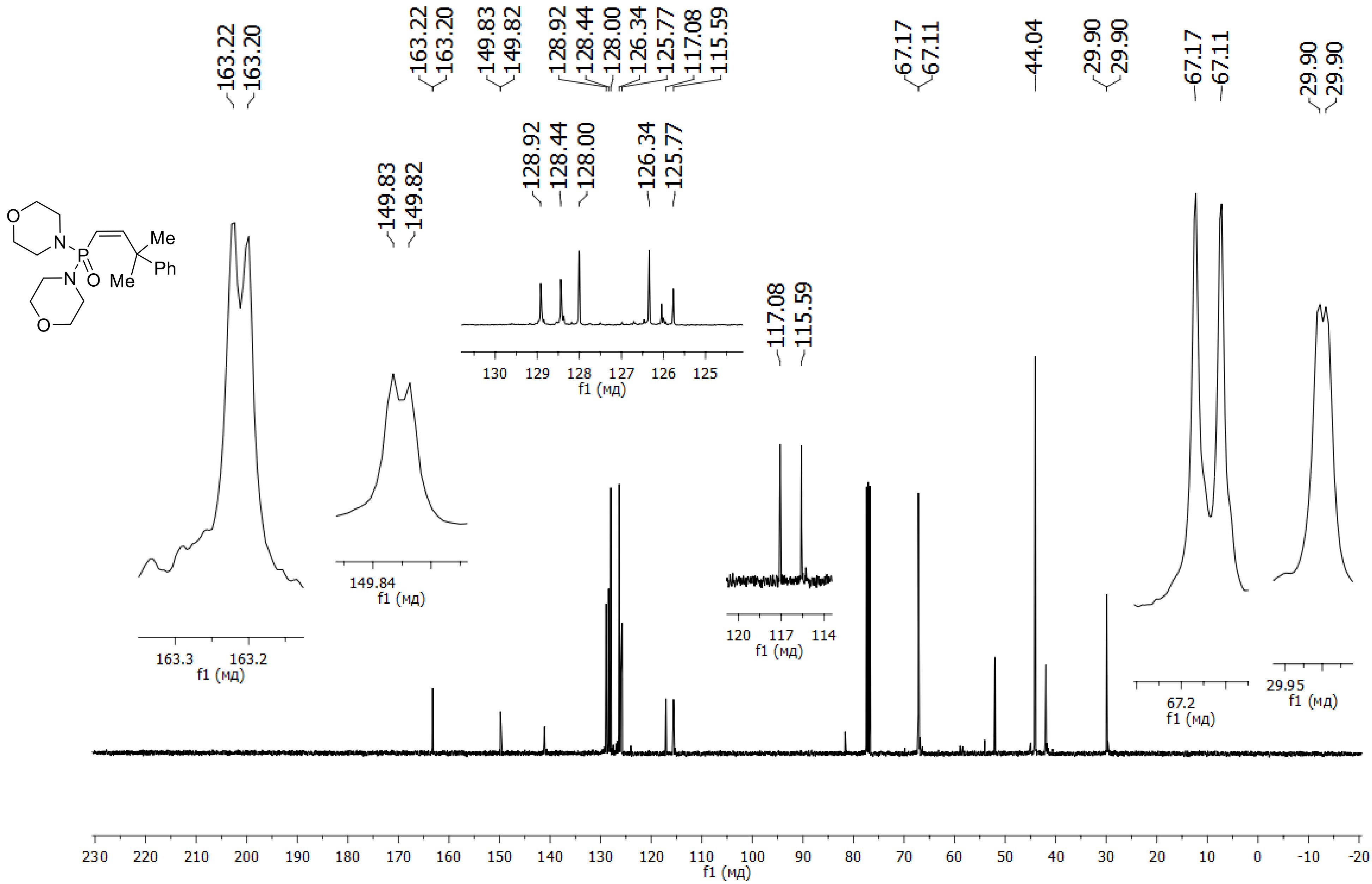

Figure S111. ^{1}H NMR spectrum of the compound **11o** (400 MHz, $CDCl_3$)

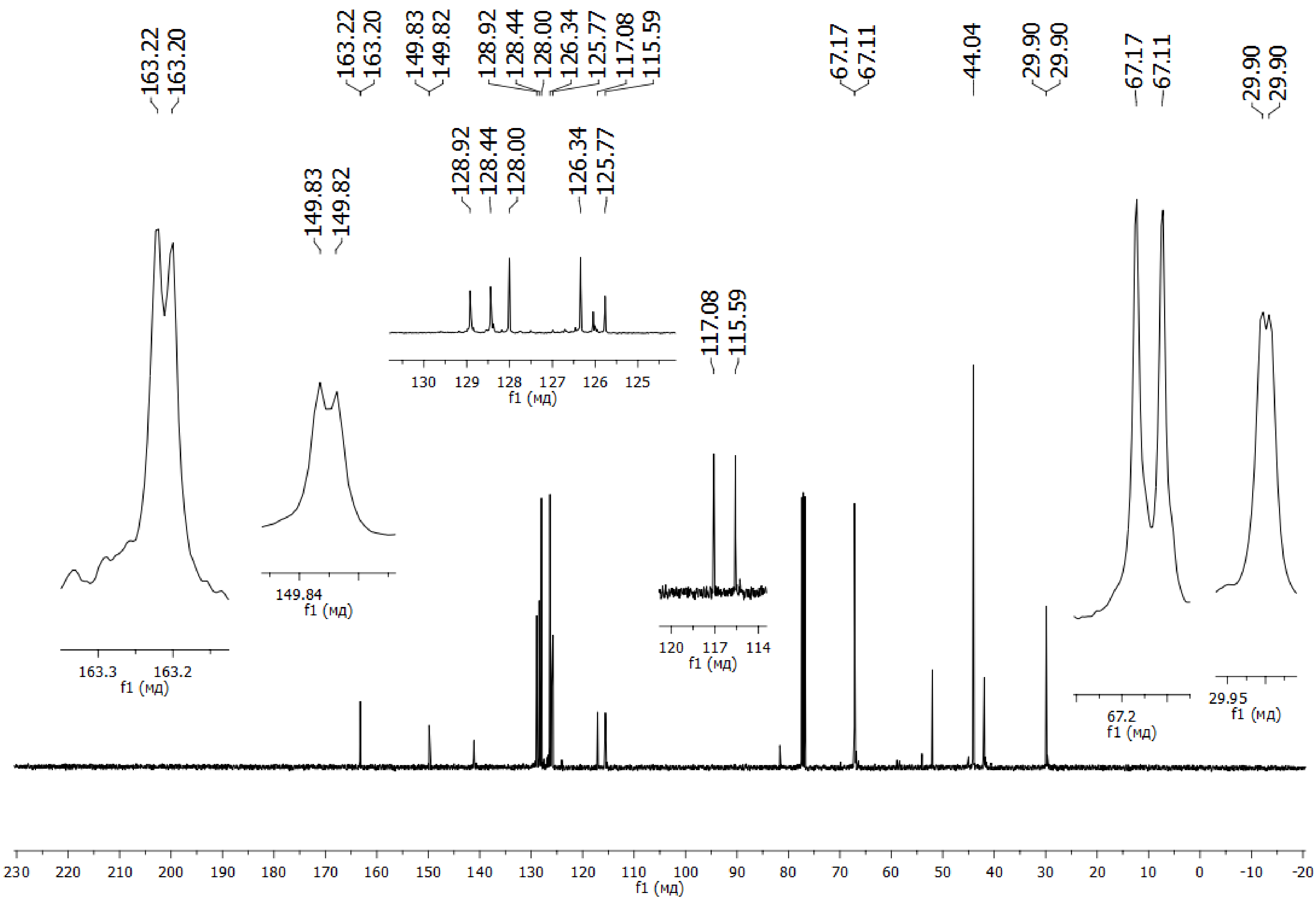

Figure S112. ^{13}C NMR spectrum of the compound **11o** (101 MHz, $CDCl_3$)

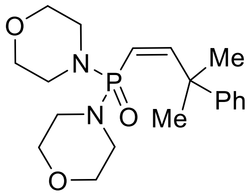

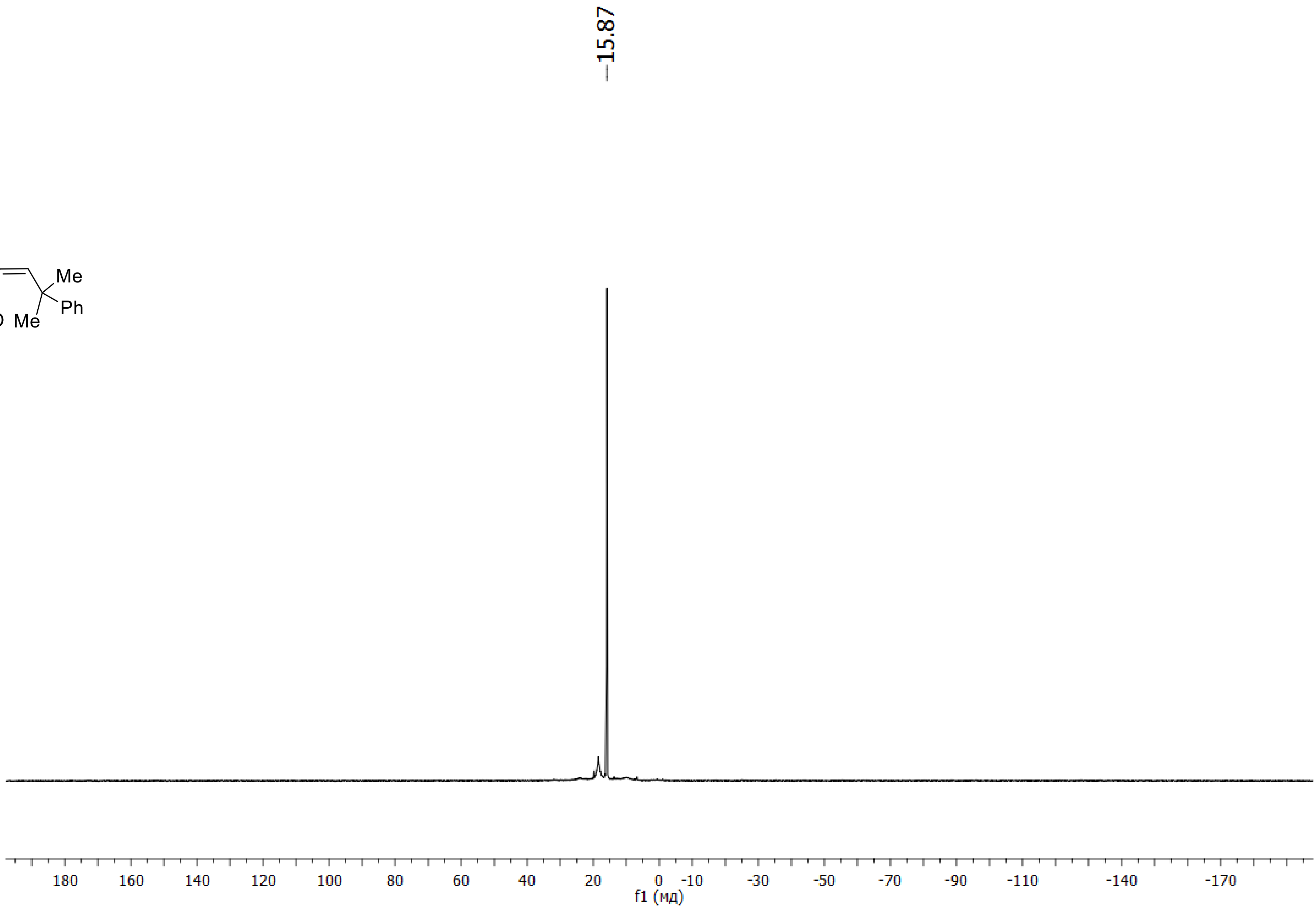

Figure S113. ^{31}P NMR spectrum of the compound **11o** (162 MHz, $CDCl_3$)

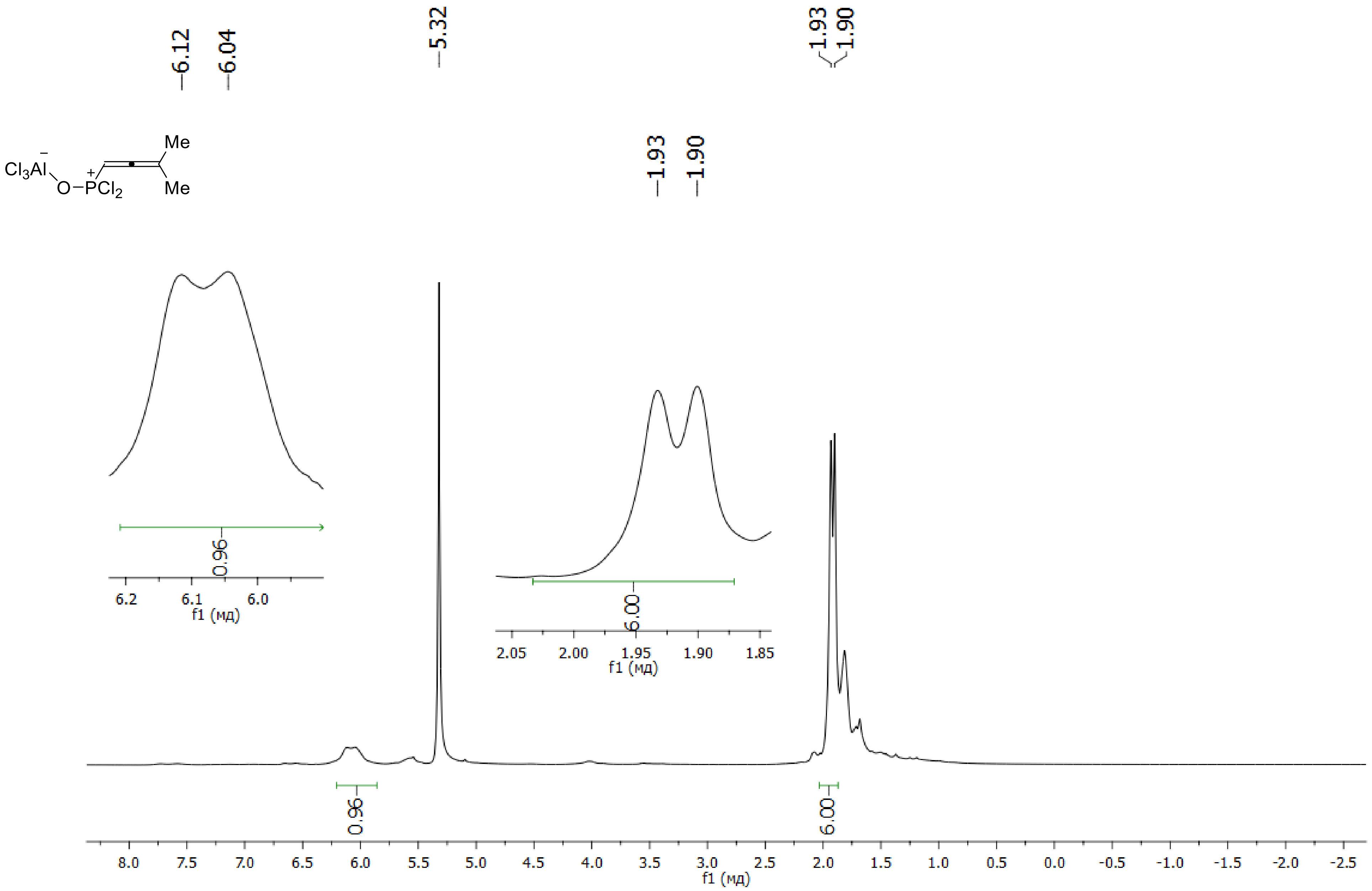

Figure S114. ^{1}H NMR spectrum of the compound **13** (400 MHz, CD_2Cl_2)

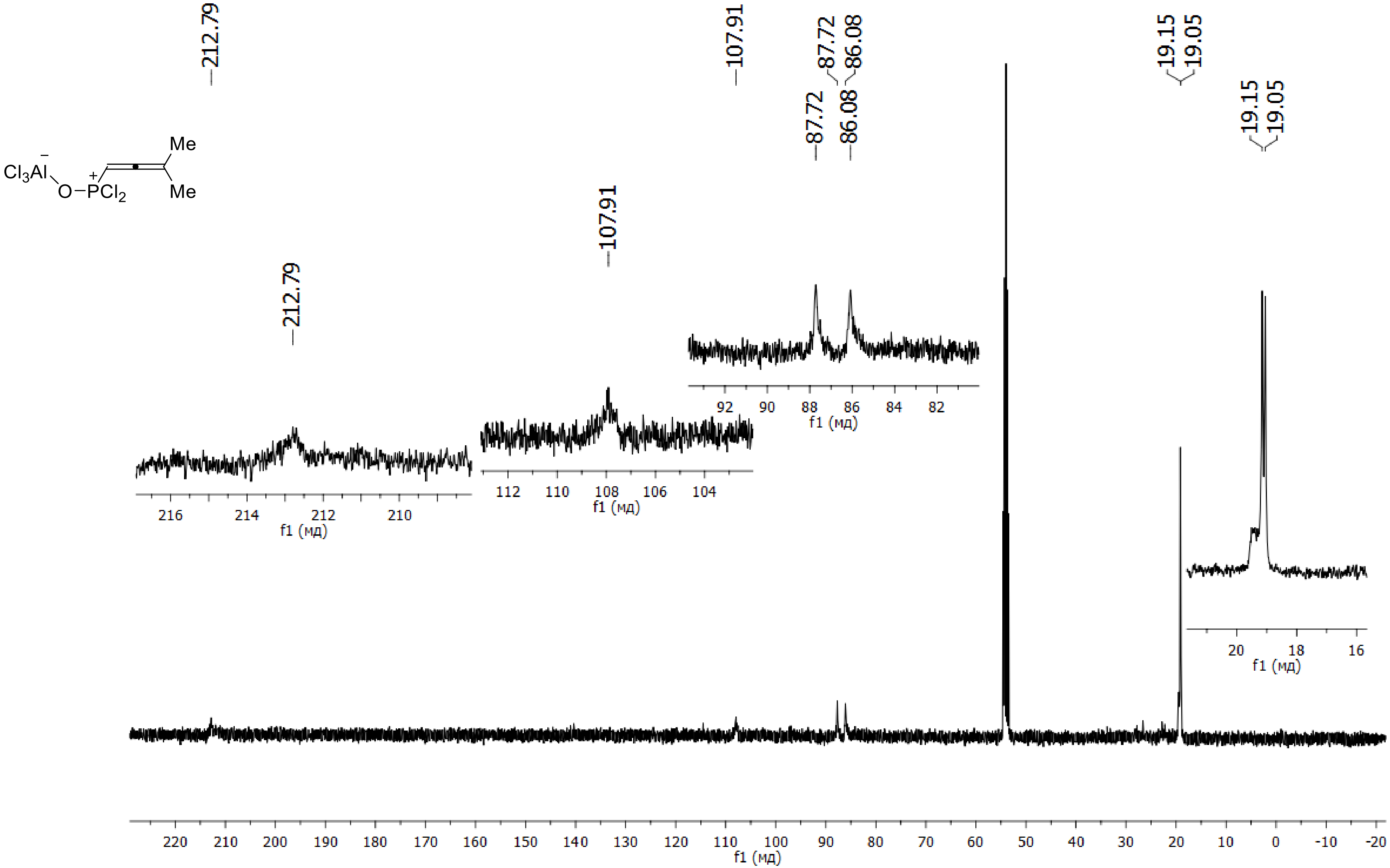

Figure S115. ^{13}C NMR spectrum of the compound **13** (101 MHz, , CD_2Cl_2)

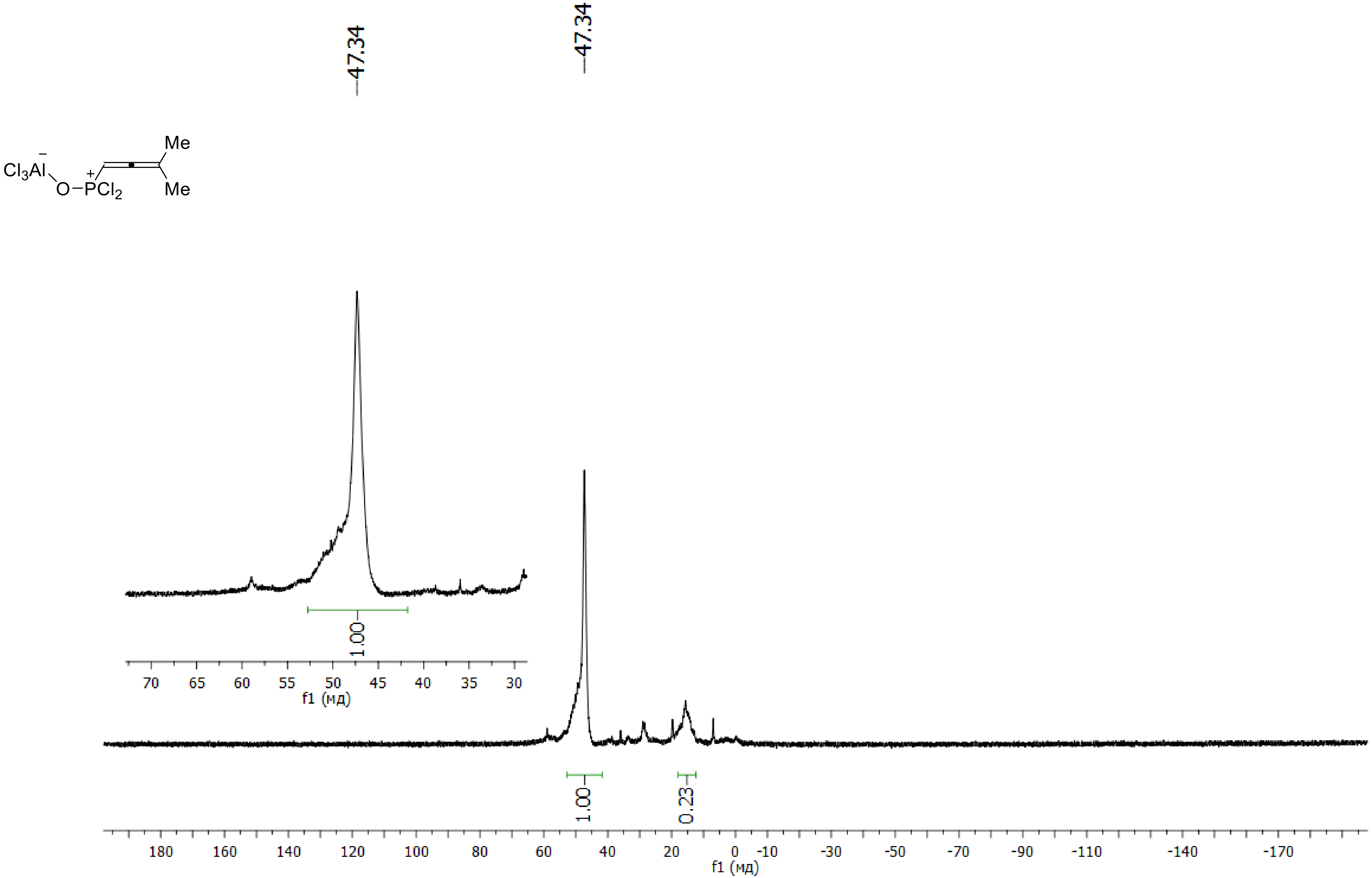

Figure S116. ^{31}P NMR spectrum of the compound **13** (162 MHz, CD_2Cl_2)

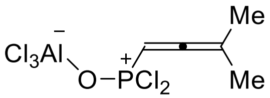

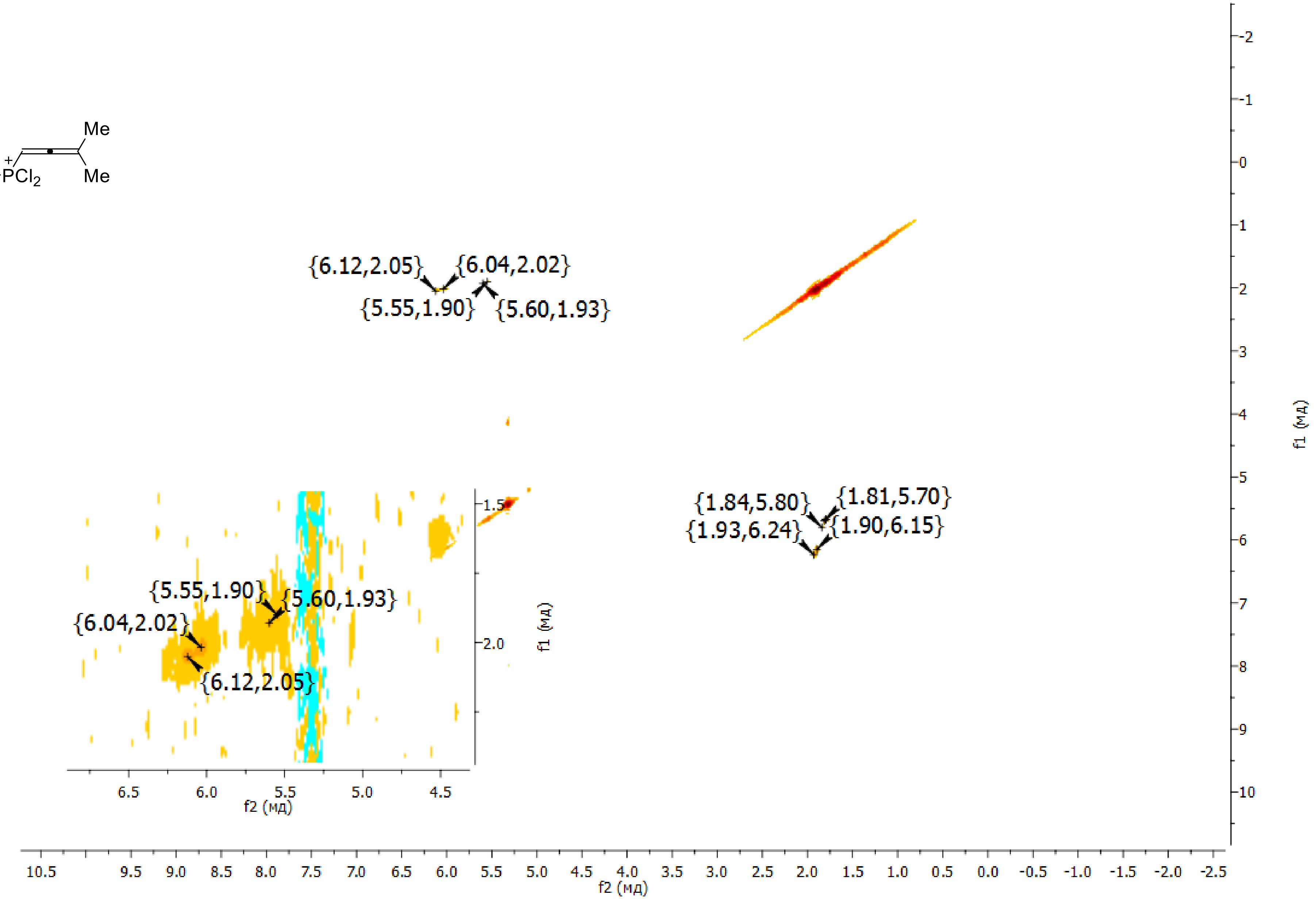

Figure S117. H-H COSY NMR spectrum of the compound **13** (400 MHz, CD_2Cl_2)

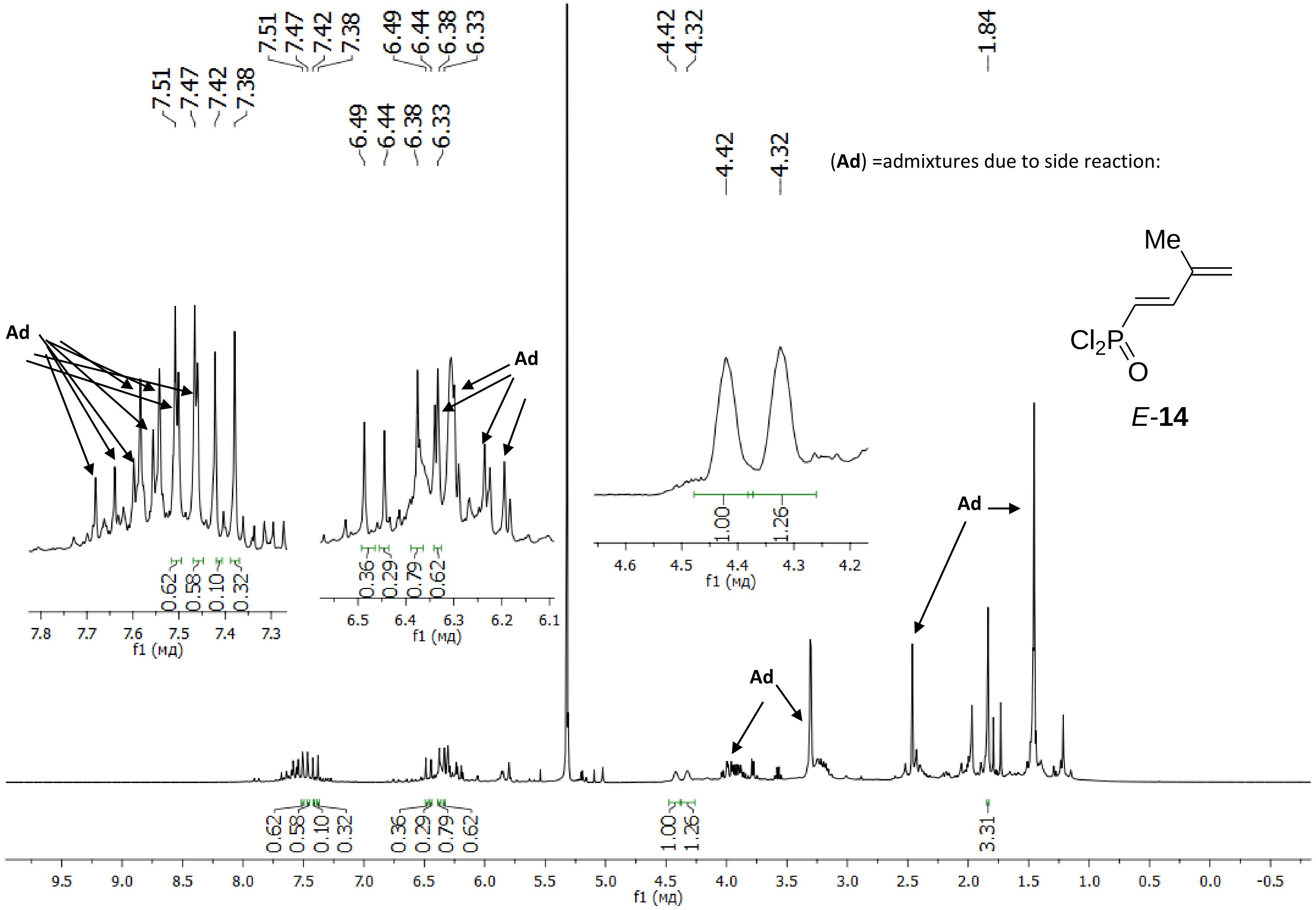

Figure S118. ^{1}H NMR spectrum of the compound **14** (400 MHz, CD_2Cl_2)

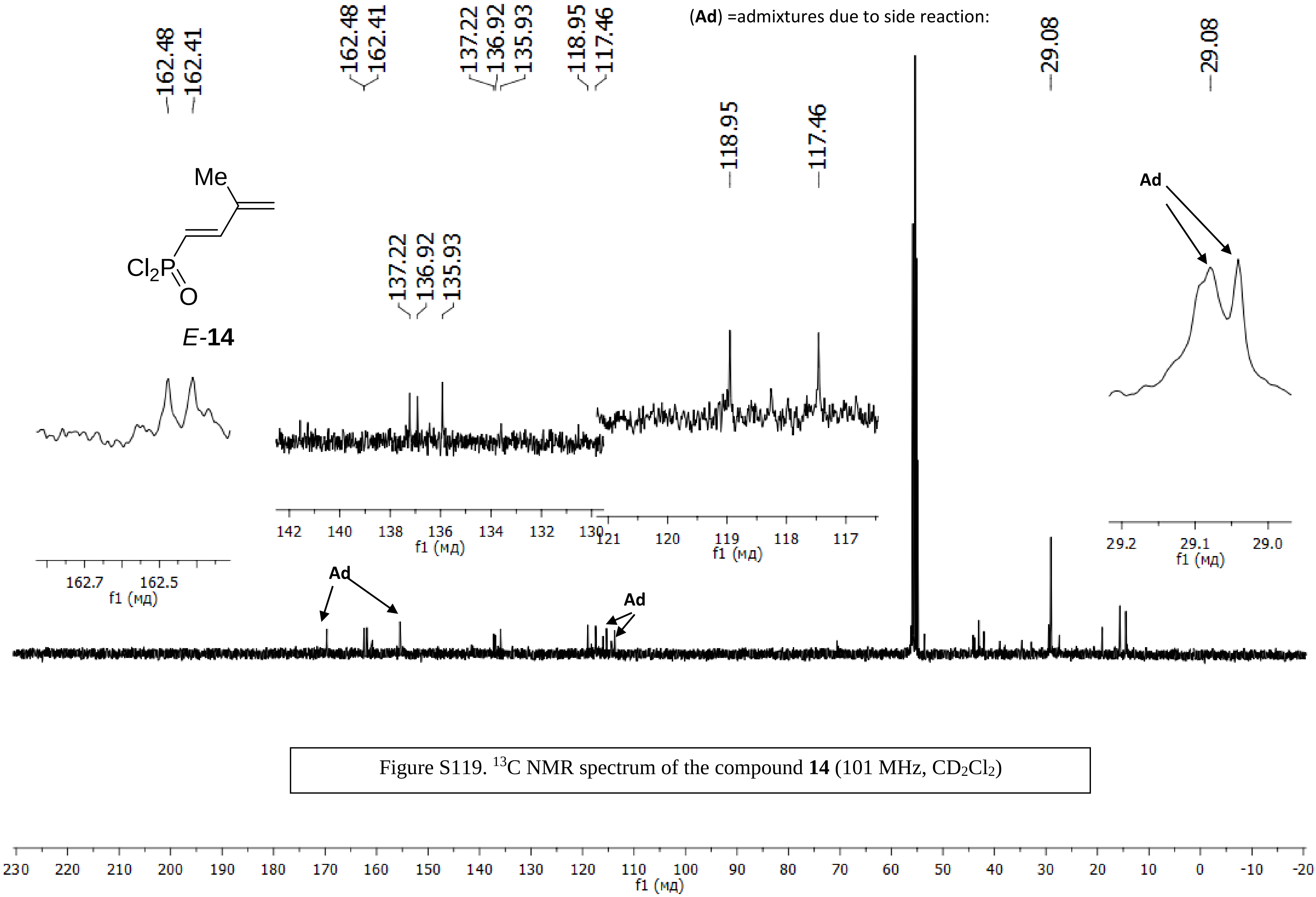

Figure S119. ^{13}C NMR spectrum of the compound **14** (101 MHz, CD_2Cl_2)

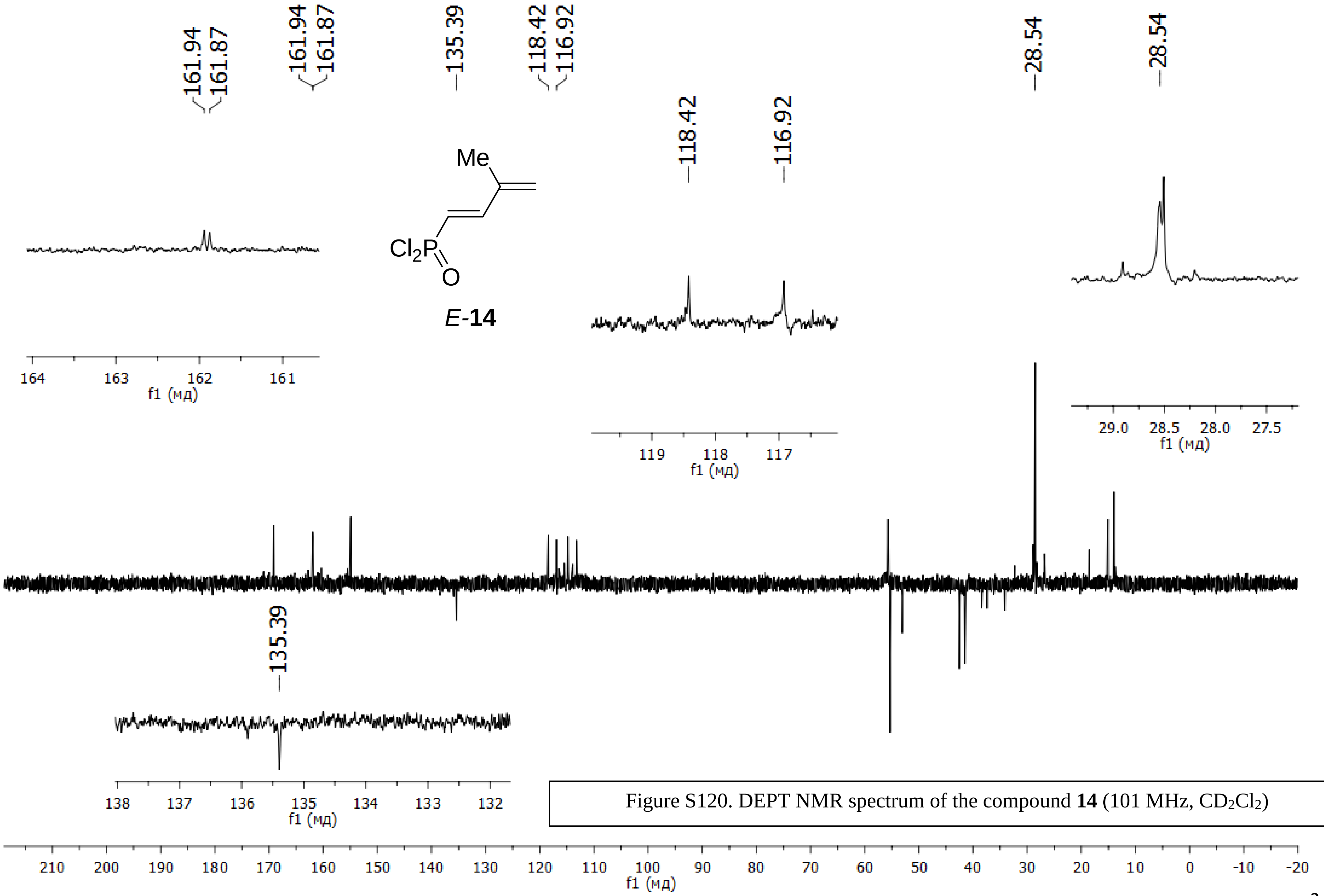

Figure S120. DEPT NMR spectrum of the compound **14** (101 MHz, CD_2Cl_2)

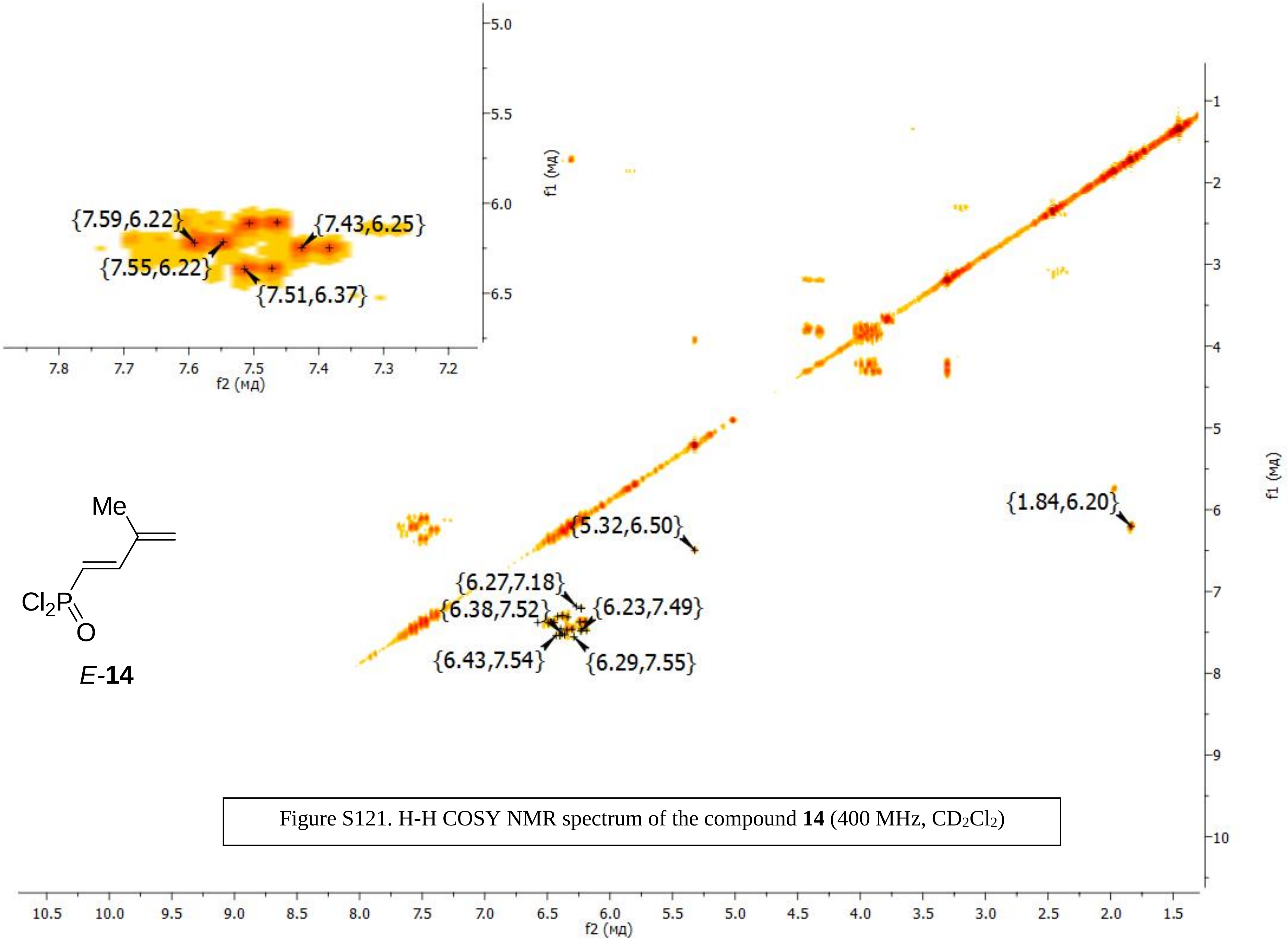

E-**14**

Figure S121. H-H COSY NMR spectrum of the compound **14** (400 MHz, CD_2Cl_2)

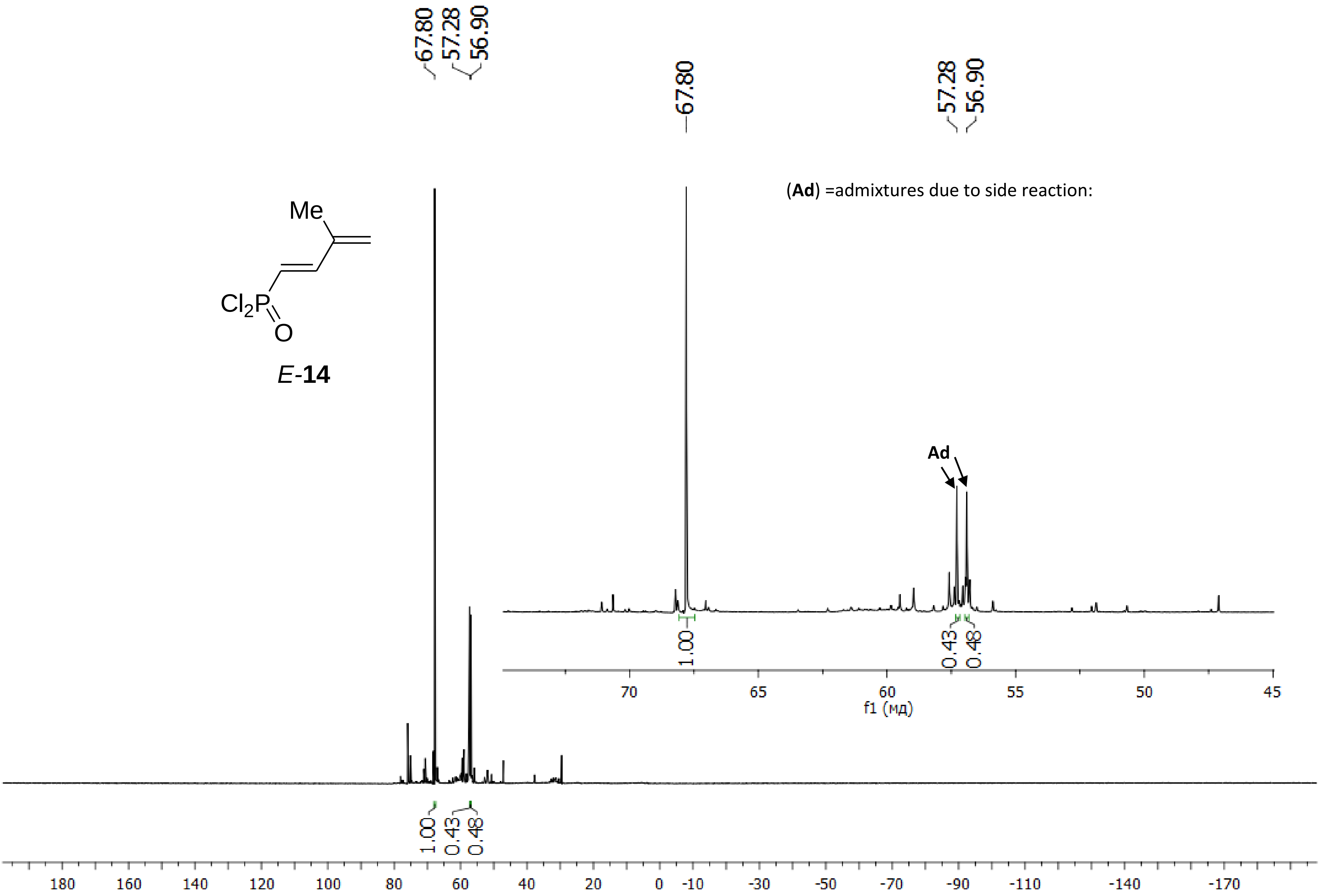

Figure S122. ^{31}P NMR spectrum of the compound **14** (162 MHz, CD_2Cl_2)

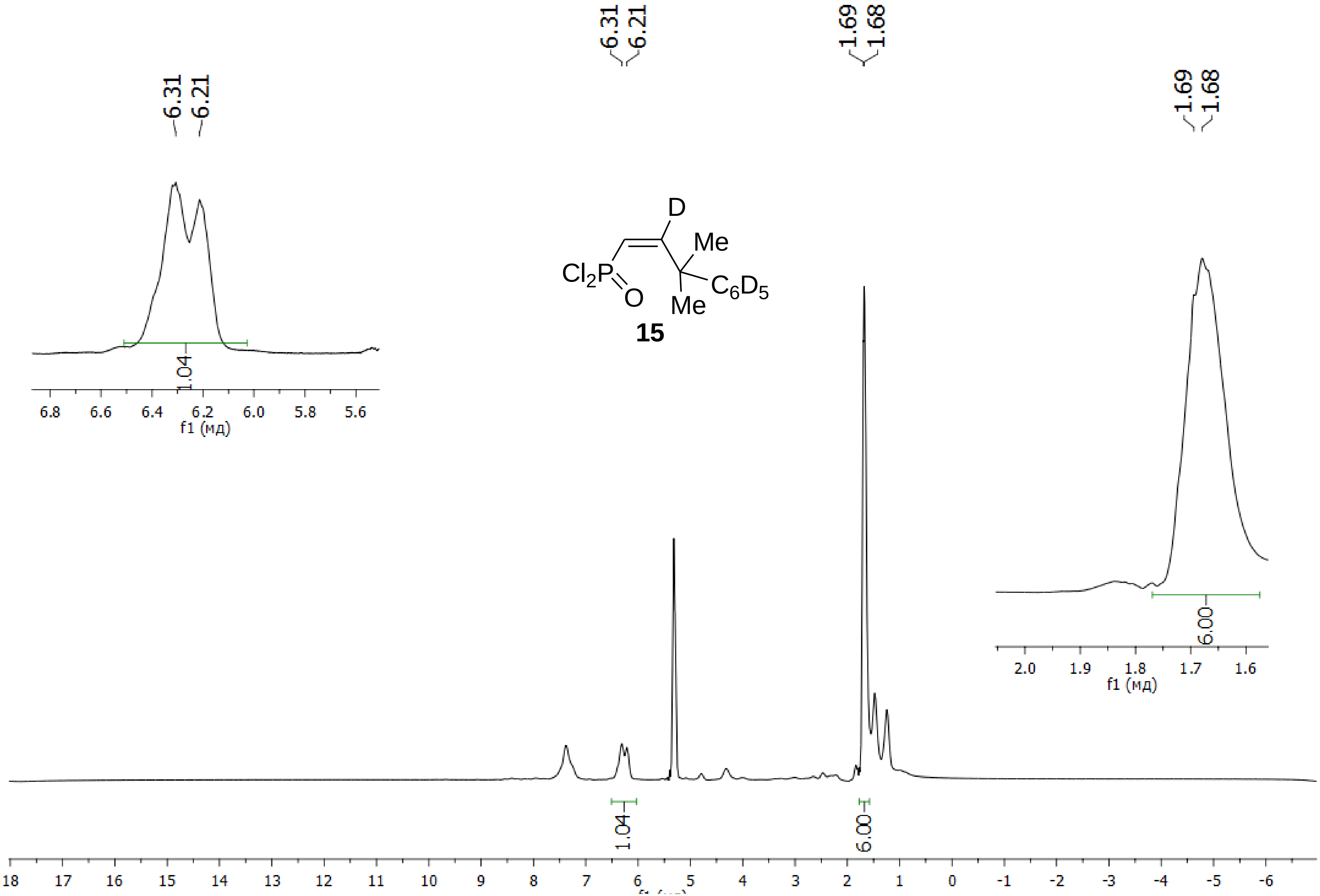

Figure S123. ^{1}H NMR spectrum of the compound **15** (400 MHz, CD_2Cl_2)

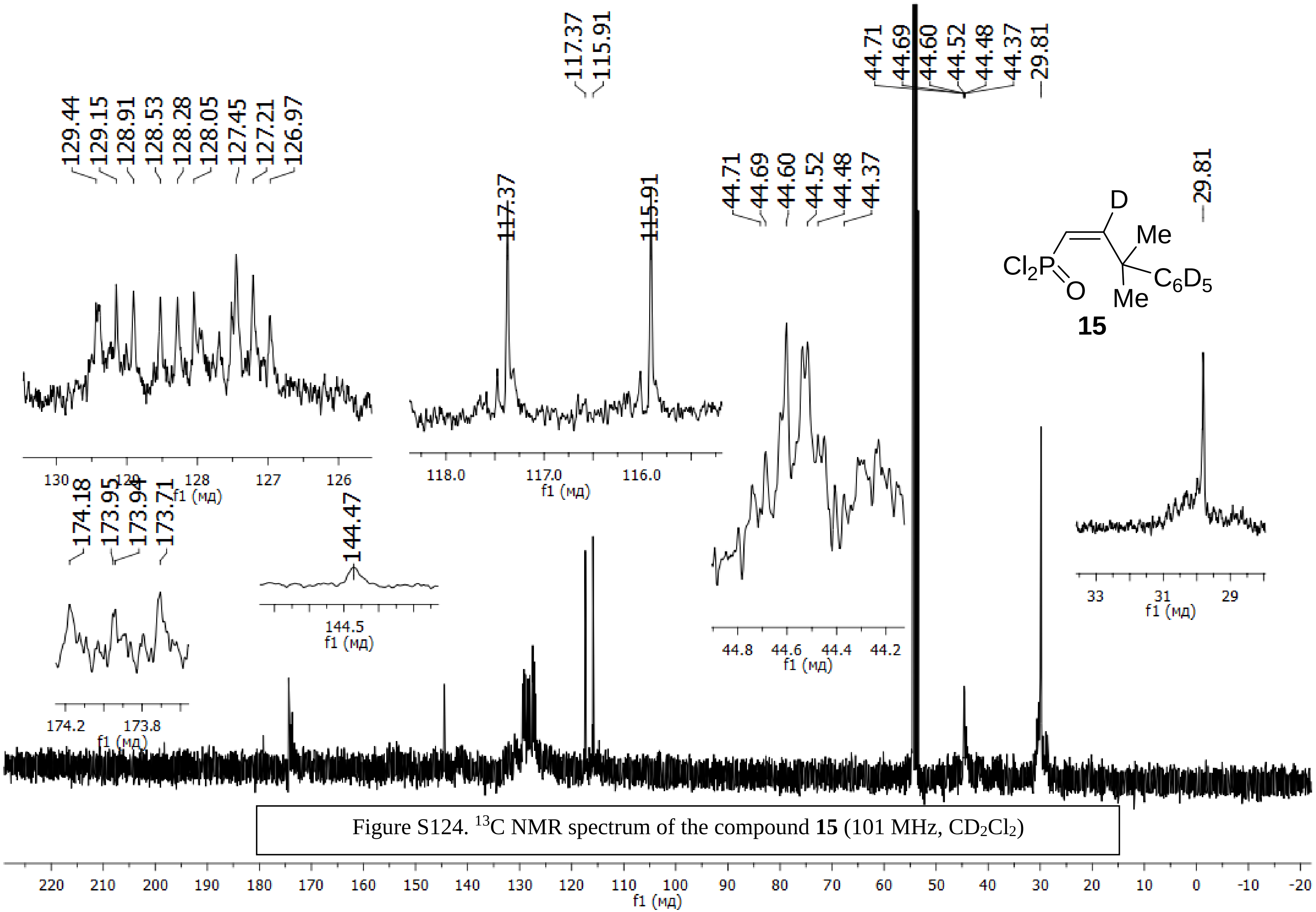

Figure S124. ^{13}C NMR spectrum of the compound **15** (101 MHz, CD_2Cl_2)

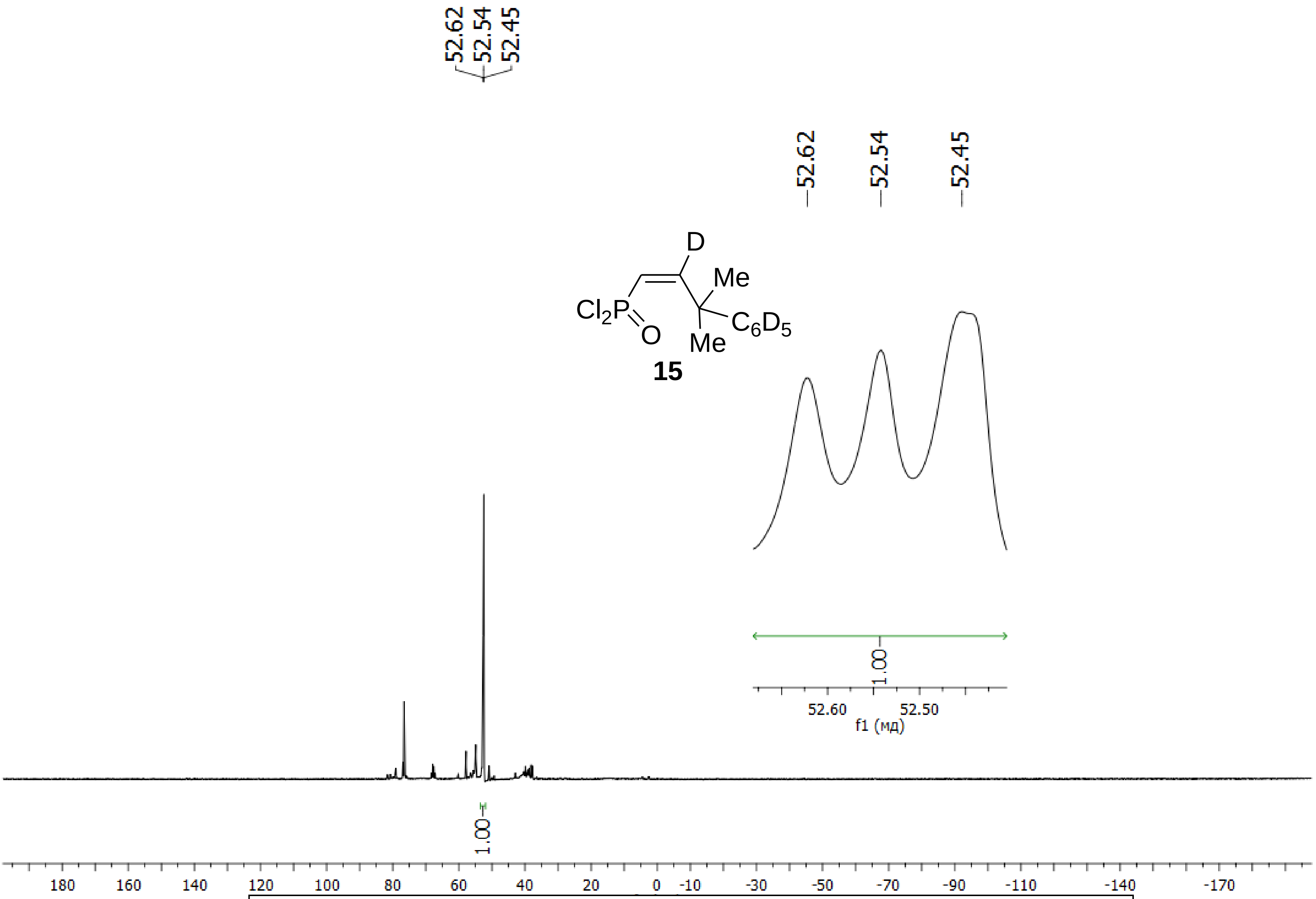

Figure S125. ^{31}P NMR spectrum of the compound **15** (101 MHz, CD_2Cl_2)

Figure S80. ^{31}P NMR spectrum of the compound **15** (162 MHz, CD_2Cl_2).

III. References

1. M. J. Frisch, *et all.*; *Gaussian 09, Revision C.01*, 2010, Gaussian, Inc., Wallingford CT.
2. Dolomanov, O. V.; Bourhis, L. J.; Gildea, R. J.; Howard, J. A. K.; Puschmann, H. OLEX2: a complete structure solution, refinement and analysis program *J. Appl. Cryst*. **2009**, *42*, 339-341.
3. Sheldrick, G. M. A short history of SHELX *Acta Cryst.* **2008**, *A64*, 112-122

4. R. S. Macomber, E. R. Kennedy, *J. Org. Chem.*, 1976, **41**, 3191–3197.

5. G. M. Kosolapoff, US Patent, 1949, 2486657.

6. R. S. Macomber, G. A. Krudy *J. Org. Chem*, 1981, **46**, 4038-4041.

7. K. S. Madden, S. David, J. P. Knowles, A. Whiting, *Chem. Commun.*, 2015, **51**, 11409–11412.

8. R. Skouta, C.-J. Li *Can. J. Chem.* **2008**, *86*, 616–620.

IV. DFT-calculations

1a

Sum of electronic and thermal Free Energies=

-1531.890640

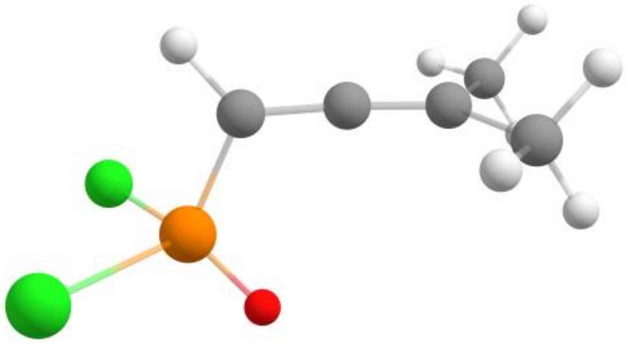

6	2.769745000	-0.000052000	-0.191402000
6	1.548643000	-0.000095000	-0.638174000
6	0.294355000	-0.000111000	-0.994817000
15	-0.948850000	0.000053000	0.287498000
1	-0.049937000	-0.000205000	-2.021336000
6	3.501599000	-1.288610000	0.084336000
1	2.892447000	-2.158530000	-0.145253000
1	3.785335000	-1.330050000	1.137615000
1	4.419058000	-1.334134000	-0.505531000
6	3.501652000	1.288496000	0.084190000
1	3.784976000	1.330206000	1.137578000
1	2.892684000	2.158425000	-0.145854000
1	4.419359000	1.333752000	-0.505299000
17	-2.166725000	-1.595797000	-0.198724000
17	-2.166328000	1.595943000	-0.198669000
8	-0.493154000	-0.000065000	1.678311000

13

Sum of electronic and thermal Free Energies=

-3155.320861

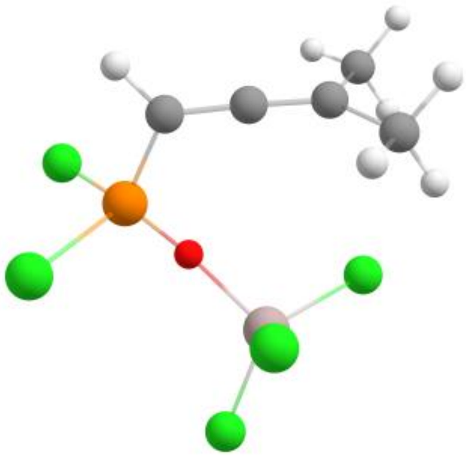

6	-1.655578000	-2.435478000	0.224733000
6	-0.376546000	-2.295046000	0.403382000
6	0.909380000	-2.089059000	0.528101000
15	1.570497000	-0.569803000	-0.077198000
1	1.614198000	-2.791342000	0.955936000
6	-2.190814000	-3.002755000	-1.064633000
1	-1.397051000	-3.283396000	-1.751498000
1	-2.817761000	-2.248853000	-1.541336000
1	-2.808199000	-3.877542000	-0.855968000
6	-2.652878000	-2.034028000	1.277886000
1	-3.272696000	-2.894336000	1.535809000
1	-3.299476000	-1.256511000	0.870725000
1	-2.173960000	-1.648248000	2.171136000
17	2.967651000	-1.019816000	-1.476844000
17	2.637450000	0.319844000	1.390839000
8	0.565362000	0.355361000	-0.697434000
13	-0.830724000	1.444207000	-0.161180000
17	-0.965226000	1.057785000	1.939443000
17	-0.209697000	3.405825000	-0.646609000
17	-2.508260000	0.710944000	-1.252055000

16

Sum of electronic and thermal Free Energies=

-4778.711172

6	0.641638000	1.188708000	0.182502000
6	1.087815000	-0.028422000	-0.016733000
6	1.055435000	-1.318218000	-0.339874000
13	3.486107000	-0.006881000	0.155321000
15	-1.051244000	1.532664000	-0.195422000
8	-1.808099000	0.329620000	-0.674885000
1	1.252611000	2.021201000	0.502143000

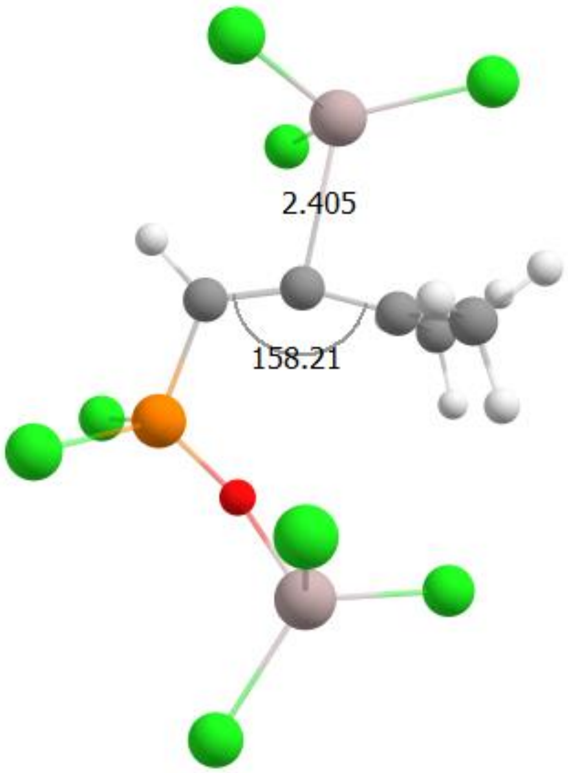

17	-1.041848000	2.935443000	-1.645690000
17	-1.902174000	2.437219000	1.384166000
13	-2.895454000	-0.998795000	0.026503000
17	-4.862542000	-0.255847000	-0.151611000
17	-2.179076000	-1.170121000	2.032175000
17	-2.384598000	-2.680882000	-1.178664000
17	3.520202000	1.033432000	1.985173000
17	4.293354000	-1.950937000	0.157801000
17	3.725939000	1.104581000	-1.620613000
6	0.851626000	-2.371558000	0.702249000
6	1.241797000	-1.752446000	-1.761436000
1	0.756515000	-1.957644000	1.699580000
1	1.676235000	-3.081937000	0.664822000
1	-0.068840000	-2.901233000	0.449695000
1	1.500790000	-0.928655000	-2.418617000
1	0.287902000	-2.183306000	-2.076157000
1	2.002504000	-2.529035000	-1.821551000

17

Sum of electronic and thermal Free Energies=

-5010.523680

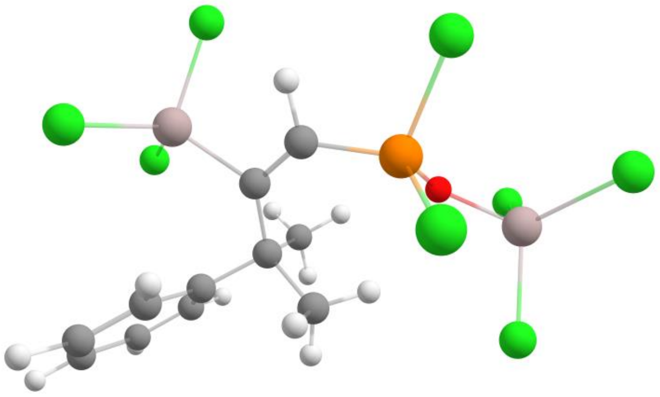

6	0.206423000	1.211199000	-0.329492000
6	1.196359000	0.364868000	0.010605000
6	1.103464000	-1.150342000	0.140554000
13	2.881525000	1.406529000	0.502231000
15	-1.516588000	0.986875000	-0.625641000
8	-2.262682000	0.086750000	0.333334000
1	0.395872000	2.279139000	-0.344654000
17	-1.966085000	0.433909000	-2.544730000
17	-2.290923000	2.867660000	-0.514218000
13	-3.882785000	-0.745373000	0.517891000
17	-5.310065000	0.566789000	-0.379962000
17	-4.113164000	-0.990553000	2.618985000
17	-3.680385000	-2.587900000	-0.543475000
17	2.199423000	3.306817000	1.309844000
17	4.042982000	0.382270000	1.997557000
17	4.003973000	1.827872000	-1.300043000
6	0.672585000	-1.409261000	1.602518000
6	0.092162000	-1.828658000	-0.808023000
1	1.405196000	-1.019275000	2.306064000
1	0.553567000	-2.478893000	1.772288000
1	-0.285876000	-0.934198000	1.795229000
1	0.273086000	-1.546947000	-1.843065000
1	-0.937661000	-1.612145000	-0.551203000
1	0.207283000	-2.909133000	-0.734039000
6	3.093265000	-2.704879000	0.639071000
6	2.464716000	-1.776326000	-0.187805000

	6	4.294331000	-2.012117000	-1.762259000
	6	3.079801000	-1.448012000	-1.396359000
	6	4.303716000	-3.281596000	0.272848000
	6	4.911761000	-2.936277000	-0.928197000
	1	5.862183000	-3.372161000	-1.204466000
	1	4.763382000	-1.713765000	-2.689427000
	1	2.620676000	-0.719084000	-2.048328000
	1	2.658672000	-2.966435000	1.590741000
	1	4.780917000	-3.987515000	0.939156000
**22**	6	-2.151747000	1.509960000	0.491116000
**Sum of electronic and thermal Free Energies=**	6	-1.354274000	2.576365000	0.498874000
**-3155.622691**	6	0.038411000	2.694456000	0.045288000
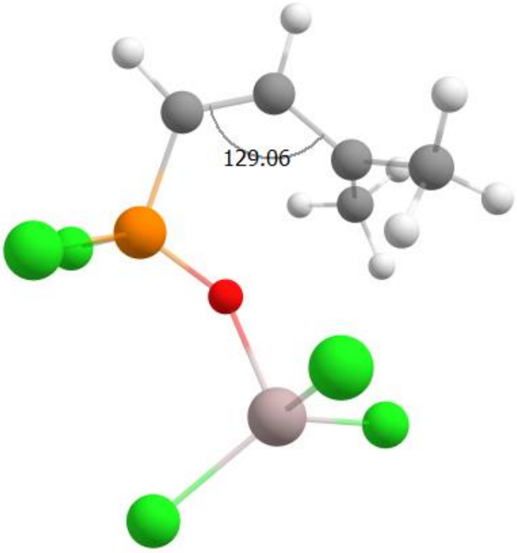	1	-1.740137000	3.506562000	0.915036000
	15	-1.575793000	-0.094930000	-0.033441000
	8	-0.131349000	0.043795000	-0.439003000
	1	-3.164031000	1.573327000	0.864642000
	17	-2.721618000	-0.687554000	-1.554596000
	17	-1.890927000	-1.365547000	1.464187000
	13	1.497438000	-0.847866000	-0.115256000
	17	1.080579000	-2.868636000	-0.445046000
	17	1.737007000	-0.270171000	1.954212000
	17	2.805781000	0.247224000	-1.364807000
	6	1.067714000	2.811910000	1.029232000
	6	0.331606000	2.791533000	-1.382444000
	1	0.741608000	3.219827000	1.984301000
	1	2.004562000	3.221122000	0.661700000
	1	1.295615000	1.722454000	1.290528000
	1	-0.468819000	2.436059000	-2.021658000
	1	1.286229000	2.306786000	-1.609891000
	1	0.491734000	3.864002000	-1.582228000
**23**	6	0.653701000	1.775502000	0.989645000
**Sum of electronic and thermal Free Energies=**	6	1.767769000	1.033525000	0.972024000
**-3387.590576**	6	2.127540000	-0.280798000	0.312300000
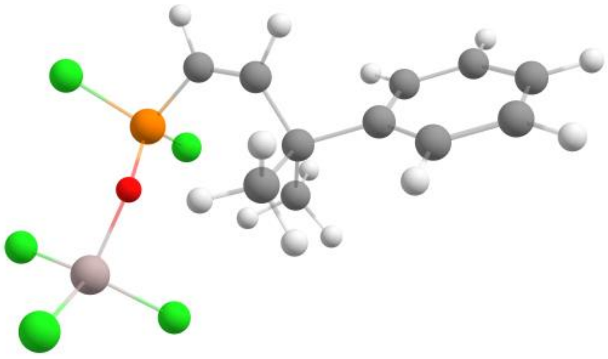	1	2.573809000	1.445320000	1.570244000
	15	-0.936323000	1.419203000	0.306620000
	8	-1.348392000	-0.014494000	0.464438000
	1	0.646984000	2.699952000	1.549807000
	17	-1.061721000	2.010801000	-1.621405000
	17	-2.193927000	2.659574000	1.286958000
	13	-2.662542000	-1.194147000	-0.124893000
	17	-4.413455000	0.009813000	-0.188815000
	17	-2.593860000	-2.709731000	1.353505000
	17	-1.954520000	-1.779534000	-2.045372000
	6	1.638753000	-1.403649000	1.255936000

6	1.490288000	-0.476323000	-1.079014000
1	2.098758000	-1.333969000	2.240696000
1	1.881271000	-2.374809000	0.828160000
1	0.560445000	-1.352772000	1.372120000
1	1.696503000	0.368349000	-1.732965000
1	0.419628000	-0.636654000	-1.028847000
1	1.919941000	-1.364847000	-1.537360000
6	3.651754000	-0.283748000	0.130096000
6	4.252020000	0.783506000	-0.545331000
6	4.467458000	-1.317547000	0.583070000
6	5.622857000	0.820041000	-0.759475000
6	5.843058000	-1.286930000	0.366756000
6	6.426295000	-0.220243000	-0.303210000
1	6.062681000	1.656769000	-1.284041000
1	7.493952000	-0.197770000	-0.469202000
1	4.043072000	-2.158838000	1.108362000
1	6.456118000	-2.101395000	0.726609000
1	3.640102000	1.596768000	-0.914370000

$AlCl_3$

Sum of electronic and thermal Free Energies=

-1623.387700

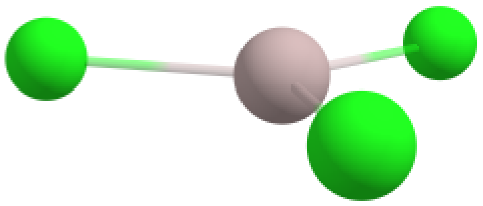

17	1.042591000	1.797439000	-0.000115000
17	1.036373000	-1.801028000	-0.000115000
17	-2.078615000	0.003588000	-0.000115000
13	-0.000456000	0.000001000	0.000452000

$HAlCl_4$

Sum of electronic and thermal Free Energies=

-2084.235525

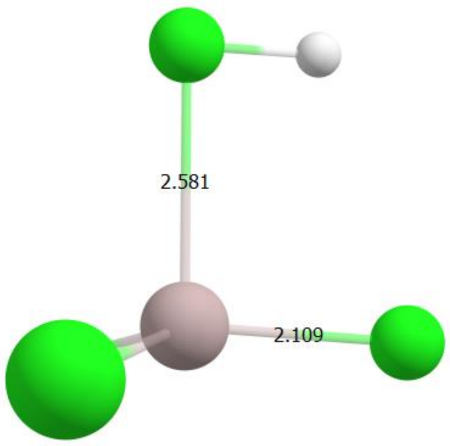

17	-2.585089000	1.827302000	-0.340975000
17	0.441411000	3.158655000	1.009739000
17	0.396133000	0.793087000	-1.711362000
17	-0.466588000	-0.271838000	1.624058000
13	-0.135669000	1.288252000	0.268842000
1	-2.364476000	1.538348000	-1.577541000

$AlCl_4^-$

17	1.246303000	-1.715895000	0.438928000

Sum of electronic and thermal Free Energies=

-2083.811161

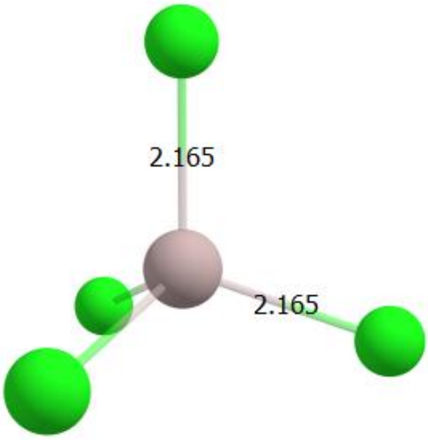

17	1.243352000	1.638360000	-0.678341000
17	-1.066617000	0.594926000	1.787784000
17	-1.422932000	-0.517259000	-1.548395000
13	-0.000139000	-0.000172000	0.000031000

H_2O

Sum of electronic and thermal Free Energies=

-76.458820

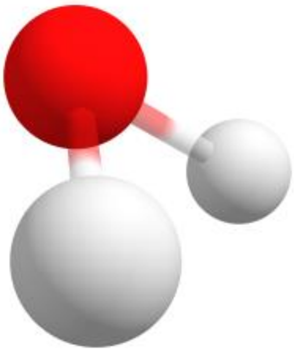

8	0.000000000	0.000000000	0.116803000
1	0.000000000	0.763023000	-0.467210000
1	0.000000000	-0.763023000	-0.467210000

H_3O^+

Sum of electronic and thermal Free Energies=

-76.718509

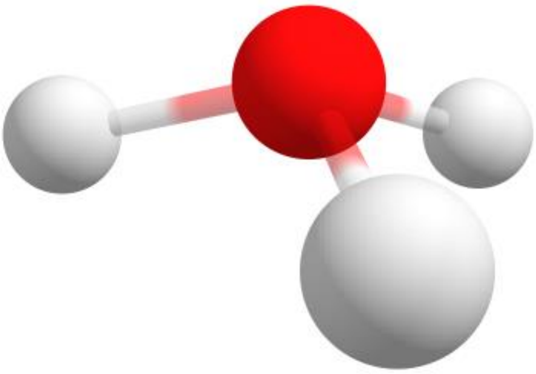

8	0.000000000	0.000000000	0.075720000
1	0.000000000	0.939460000	-0.201920000
1	0.813596000	-0.469730000	-0.201920000
1	-0.813596000	-0.469730000	-0.201920000

Benzene

Sum of electronic and thermal Free Energies=

6	-1.204914000	-0.695640000	0.000000000
6	-1.204914000	0.695640000	0.000000000
6	-0.000217000	1.391518000	0.000000000

-232.266703				
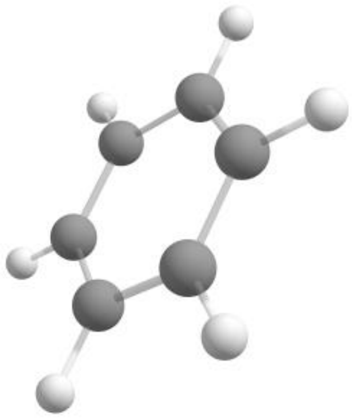	6	1.205045000	0.695530000	0.000000000
	6	1.205045000	-0.695530000	0.000000000
	6	-0.000217000	-1.391518000	0.000000000
	1	0.000479000	-2.472693000	0.000000000
	1	2.141125000	-1.236680000	0.000000000
	1	2.141125000	1.236680000	0.000000000
	1	0.000479000	2.472693000	0.000000000
	1	-2.141087000	1.236776000	0.000000000
	1	-2.141087000	-1.236776000	0.000000000

V. X-ray data for 3a

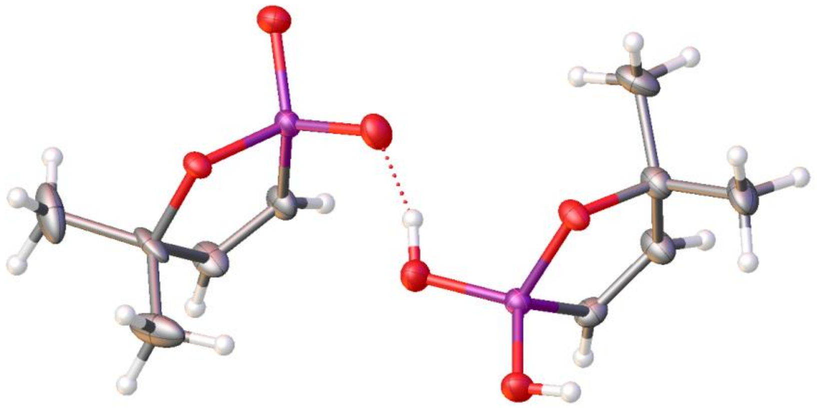

Compound 3a, CCDC number 1870758

Table 1 Crystal data and structure refinement for 7071-9778_lza061.

Identification code	7071-9778_lza061
Empirical formula	$C_5H_9O_3P$
Formula weight	148.09
Temperature/K	99.99(18)
Crystal system	orthorhombic
Space group	$Pmc2_1$
a/Å	9.4877(3)
b/Å	6.7742(2)
c/Å	11.0033(4)
α/°	90
β/°	90
γ/°	90
Volume/Å^3	707.20(4)
Z	4
ρ_{calc}g/cm^3	1.391
μ/mm^{-1}	2.968
F(000)	312.0
Crystal size/mm^3	0.2 × 0.15 × 0.11
Radiation	CuKα (λ = 1.54184)
2Θ range for data collection/°	9.322 to 139.968
Index ranges	$-11 \leq h \leq 11$, $-8 \leq k \leq 7$, $-13 \leq l \leq 13$
Reflections collected	6074
Independent reflections	1390 [R_{int} = 0.0491, R_{sigma} = 0.0270]

Data/restraints/parameters	1390/1/99
Goodness-of-fit on F^2	1.132
Final R indexes [I>=2σ (I)]	R_1 = 0.0568, wR_2 = 0.1512
Final R indexes [all data]	R_1 = 0.0568, wR_2 = 0.1512
Largest diff. peak/hole / e $Å^{-3}$	0.65/-0.58
Flack parameter	0.02(3)
